# The First Simplified Heparan Sulphate—Alginate Hybrid Trisaccharides: Synthesis and Biological Effects on Human Dermal Fibroblasts

**DOI:** 10.3390/ijms26178305

**Published:** 2025-08-27

**Authors:** Katalin Kútvölgyi, Zsófia Peleskei, Fruzsina Demeter, Roland A. Barta, Attila Mándi, Eszter Homoki, Attila Oláh, János Hajkó, Mihály Herczeg, Erika Lisztes

**Affiliations:** 1 Department of Pharmaceutical Chemistry, Faculty of Pharmacy, University of Debrecen, Egyetem tér 1, H-4032 Debrecen, Hungary; kutvolgyi.katalin@pharm.unideb.hu (K.K.); peleskei.zsofia@pharm.unideb.hu (Z.P.); demeter.fruzsina@science.unideb.hu (F.D.); 2 Doctoral School of Pharmacy, Medical Science Doctoral Council, University of Debrecen, Egyetem tér 1, H-4032 Debrecen, Hungary; 3 TAPI Hungary Industries Kft., Pallagi út 13, H-4042 Debrecen, Hungary; janos.hajko@tapi.com; 4 Department of Organic Chemistry, University of Debrecen, P.O. Box 400, H-4002 Debrecen, Hungary; barta.roland@science.unideb.hu (R.A.B.); mandi.attila@science.unideb.hu (A.M.); 5 Department of Physiology, Faculty of Medicine, University of Debrecen, P.O. Box 22, H-4012 Debrecen, Hungary; homokiesztu@gmail.com (E.H.); olah.attila@med.unideb.hu (A.O.); 6 Doctoral School of Molecular Medicine, University of Debrecen, Egyetem tér 1, H-4032 Debrecen, Hungary

**Keywords:** heparan sulphate, alginate, trisaccharides, mannuronic acid, anti-inflammatory, dermal fibroblasts

## Abstract

Glycosaminoglycans (GAGs) are linear, high molecular weight polydisperse heteropolysaccharides consisting of repeating disaccharide units, which always contain a uronic acid building block (e.g., d-glucuronic acid or l-iduronic acid). Their analogues containing d-mannuronic acid were not known until now. Another important class of the linear negatively charged polisaccharides are alginates, which are also present in the cell surface in the cell wall. They are composed of blocks of 1,4-linked β-d-mannuronic acid and its C-5 epimer α-l-guluronic acid in alternating or random order. Both groups of molecules have significant biological activity (e.g., cell growth inhibitory activity, anti-inflammatory effect, etc.). In the course of our research, we combined the structural characteristics of these two groups of molecules and produced a series of heparan sulphate analogue trisaccharides containing d-mannuronic acid, with a simplified structure, in which α- and β-mannosidic bonds are also found. Since trisaccharides may exert diverse biological effects and alginate derivatives can influence wound healing processes, we investigated the effects of the synthesized compounds on primary human dermal fibroblasts. We found that, when applied at 10 μM, none of the compounds influenced viability or spontaneous collagen production; however, some derivatives exhibited anti-inflammatory activity and suppressed the poly(I:C)-induced release of interleukin 6.

## 1. Introduction

**Figure 1:**
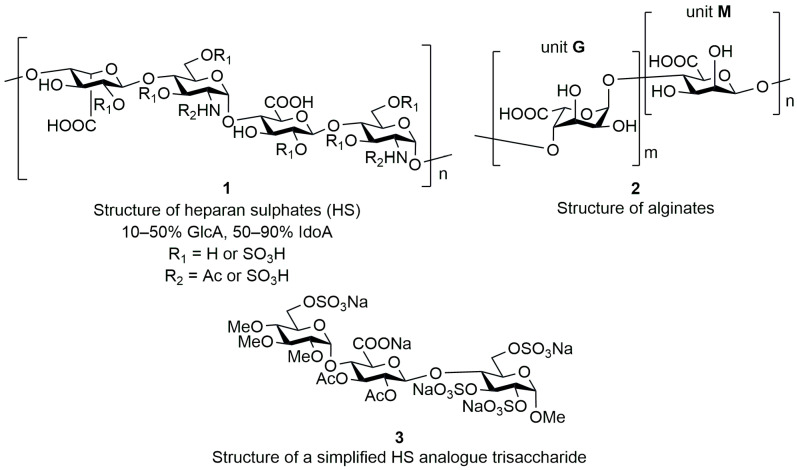
Structure of heparan sulphates (**HS**; **1**), alginates (**2**), and a simplified analogue trisaccharide of heparan sulphates (**3**).

In recent decades, researchers have paid increasing attention to the highly negatively charged polysaccharides and higher oligosaccharides in the cell surface or in the extracellular matrix, as they play various important roles. A remarkable class of these structures is the group of glycosaminoglycans (GAGs), which are linear, high molecular weight polydisperse heteropolysaccharides consisting of repeating disaccharide units [1,2,3]. One component of the repeating disaccharide units is always *N*-acetylglucosamine or *N*-acetylgalactosamine and the other building block is a uronic acid (e.g., d-glucuronic acid or l-iduronic acid). GAGs often contain sulphate ester groups in a variety of patterns [4,5]. Heparan sulphates (**1** HS) (Figure 1) are particularly important GAG molecules. Indeed, they have been used as anticoagulants in the medical practice since the late 1930s [6,7]. However, in addition to inhibiting the blood coagulation, these compounds also have many other biological effects [8,9,10,11,12]. It is well known from the literature that heparin and its derivatives may also have anti-inflammatory [13], cardiovascular and tissue protective [14,15], kidney and nerve protective [16], angiogenic, metastasis, and growth factor inhibitory [17,18,19,20,21,22,23], as well as anti-protozoan and anti-viral activity [24,25,26]. It is also known that an oligosaccharide moiety of at least five sugar units is required for the anticoagulant effect. This unique pentasaccharide moiety extracted from the heparin polymer has a specific factor Xa inhibitory effect [27,28,29,30,31]. However, fragments with a smaller or different structure (e.g., di-, tri-, and tetrasaccharides) have no measurable anticoagulant effect [32,33].

**Figure 2:**
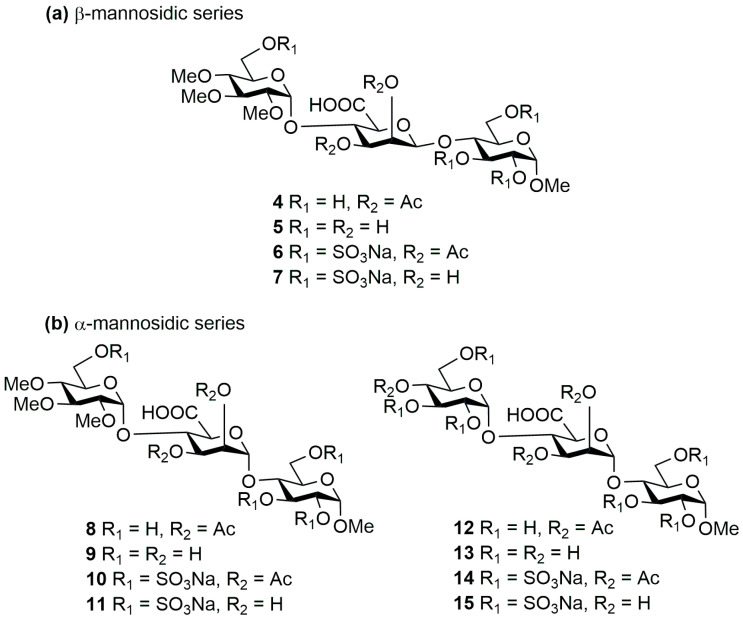
Structure of the planned (**a**) β- and (**b**) α-bond mannuronic acid containing trisaccharides (**4**–**15**).

Another important class of these structures are alginates (Figure 1, **2**). Alginates are linear anionic polysaccharides present in the cell walls of brown seaweeds. They are composed of blocks of 1,4-linked β-d-mannuronic acid (**M**) [34], its C-5 epimer α-l-guluronic acid (**G**), and both **M** and **G** arranged in alternating or random order [34,35]. Alginic acid, a widely used natural polysaccharide, which is generally derived from brown seaweeds such as Kelp, Gulfweed, Ascophyllum, and Macroalgae. It can also be produced by microbial fermentation using specialized bacteria [36]. Alginic acid naturally exists in the cytoplasm and plays an important role in strengthening the cell wall [37]. Modern pharmacological studies have shown that alginic acid has anti-anaphylaxis effect [38], immunomodulatory activities [39,40,41,42,43], antioxidant activities [44,45,46] and anti-inflammatory effects [44,47,48]. It also has been reported that alginic acid extracted from brown algae can lower blood cholesterol levels and may also have antihypertensive potential. Moreover, due to their favourable properties, such as biocompatibility and lack of toxicity, alginates have recently become particularly attractive in wound healing applications [49,50].

**Figure 3:**
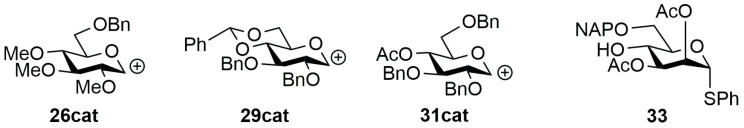
Structure of the carbocations of donors **26**, **29**, and **31**, and theoretical model compound of **25** (**33**) used for the calculations.

In our previous research, we have already prepared several trisaccharide derivatives containing d-glucuronic acid and l-iduronic acid and investigated the possible biological effects of these compounds [51,52]. Among the prepared derivatives with simplified structures (containing d-glucose instead of *N*-acetyl-d-glucosamine unit), we found a compound with a particularly good cell growth inhibitory effect (Figure 1, **3**), which selectively inhibited the growth of tumour cells only, without affecting the viability of healthy cells. There were also some molecules among them that showed promising anti-inflammatory effects [52].

As a continuation of this research, our goal was to synthesize a trisaccharide series that contains d-mannuronic acid instead of d-glucuronic acid, thus retaining the uronic acid function, hopefully combining the favourable biological properties of the two oligosaccharide families (heparan sulphate and alginate). In the course of our work, we also prepared derivatives containing α- and β-mannosidic bonds, thus investigating the significance of the spatial position of the mannosidic bond, and we also synthesized derivatives containing methyl groups or sulphate ester groups at the non-reducing end (Figure 2, **4**–**15**).

Furthermore, we varied the ratio of acetyl-/OH- groups. Thus, with the obtained, variously functionalized compounds, we could investigate the influence of lipid solubility and sulphate degree on the potential biological activity. In this study, we synthesized 11 novel trisaccharides and investigated their biological effects on primary human dermal fibroblasts (FBs). These cells play a critical role in maintaining skin structure and function, serving as the primary dermal mediators of extracellular matrix (ECM) production and dynamic remodelling [53,54], and hence, FBs are essential for proper tissue repair and regeneration. Additionally, through their paracrine signalling (e.g., cytokine and growth factor secretion), FBs actively modulate regenerative processes and fine-tune local inflammatory responses, both of which are crucial for efficient wound healing [55].

## 2. Results and Discussion

### 2.1. Synthesis of the Trisaccharides and the In Silico Investigations

#### 2.1.1. Synthesis of the Protected Trisaccharides

**Scheme 1:**
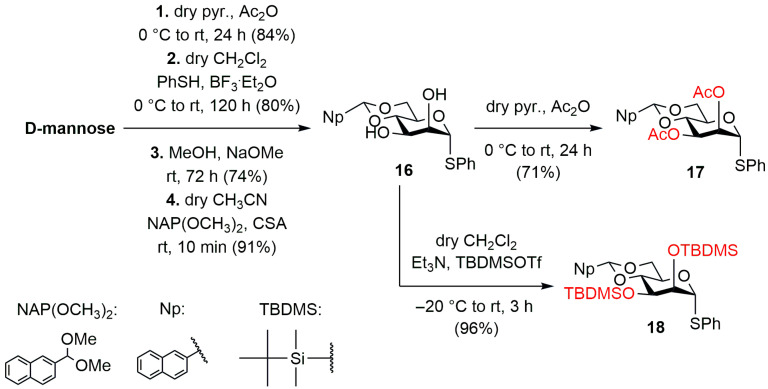
Synthesis of the d-mannose monosaccharide building blocks (**17** and **18**).

Based on our previous experiences, due to the cumbersome work with uronic acids, we planned to build the carboxyl function at the oligosaccharide level, so we started the work by preparing precursor versions of d-mannuronic acid units (Scheme 1). For this, starting from the easily accessible d-mannose, based on literature examples, we prepared derivative **16**, protected with 4,6-*O*-naphthylmethylidene acetal and having an activatable –SPh anomeric group, in 4 steps [56,57,58,59,60]. The formation of the α-mannosidic bond can be implemented more simply, for this was enough to form a participating group at the C-2 position. We chose the already well-proven acetyl group for this purpose, which in our case will control the spatial position of the glycosidic bond to be formed and is resistant to all planned reaction conditions, so it can be used as a functional group in our planned target compounds. For this, the free hydroxyl groups at position C-2 and C-3 of compound **16** were acetylated in dry pyridine using Ac_2_O, and the mannuronic acid precursor molecule **17** was obtained in good yield.

The formation of the β-mannosidic bond was much more challenging, so based on the work of Crich et al. [60,61,62,63], we prepared the disilyl derivative (**18**) of compound **16** under alkaline conditions in dry CH_2_Cl_2_ using *tert*-butyldimethylsilyl trifluoromethanesulphonate (TBDMSOTf). The TBDMSO-group at the C-2 position functions as a non-participating group, and the directing effect of the 4,6-*O*-acetal group in this case allows for the formation of the β-mannosidic bond under special glycosylation conditions. Other protecting groups used during the synthesis were chosen such that the hydroxyl groups bearing sulphate esters in the target compounds were protected with benzyl ethers, and the free hydroxyls in the final state were masked with acetyls. The chain extension C-4 position was protected in 4,6-*O*-acetal form (Ph or Np), which, upon opening, gave 6-*O*-benzyl and 6-*O*-(2-naphthylmethyl) (NAP) groups. Furthermore, this latter group (NAP) protected the –OHs corresponding to the carboxyl groups until the oxidation step.

**Scheme 2:**
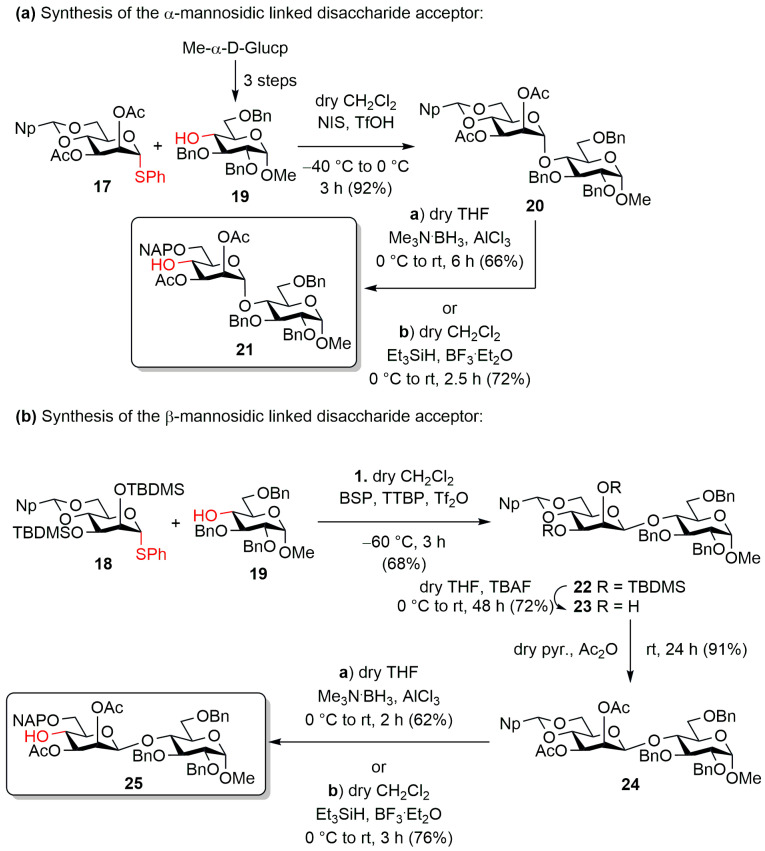
Preparation of the α- (**a**, **21**) and β-linked (**b**, **25**) disaccharide acceptors.

The designed trisaccharides were constructed by stepwise synthesis starting from the reducing end unit (Scheme 2a). For this, the appropriately protected d-glucose building block (**19**) [64] was already available from previous syntheses. The monosaccharide acceptor **19** was glycosylated with the thiomannoside donor **17** in dry CH_2_Cl_2_ using *N*-iodosuccinimide/trifluoromethanesulphonic acid (NIS/TfOH) promoter system. In the reaction, the expected protected disaccharide was formed in excellent yield and stereoselectivity, which was converted into an acceptor (**21**) suitable for chain extension in a reductive ring-opening reaction. The construction of the trisaccharides required an opening method that resulted in the 4-OH/6-ether product. For this, two reaction conditions were tested to find the best yielding conversion option. First, we used the trimethylaminoborane/aluminum trichloride (Me_3_N·BH_3_/AlCl_3_) reagent combination to open the acetal ring in dry THF, which provided the expected C-4′-OH-containing disaccharide in good yield, but the product still contained borane impurities after multiple column chromatography purification. We then switched to the reaction using the triethylsilane/boron trifluoride diethyl etherate (Et_3_SiH/BF_3_·Et_2_O) reagent combination, which provided the expected product (**21**) in better yield and full regioselectivity and had no problems with purification.

To prepare the β-mannosidic bond-containing disaccharide (Scheme 2b), acceptor **19** was also glycosylated with the monosaccharide donor containing silyl protecting groups (**18**). The key step of the synthesis of the disaccharide was the coupling of the armed phenylthio-mannosyl donor **18** to the acceptor **19**, based on a chemoselective pre-activation. In the reaction, we used the activator combination developed by Crich et al. (1-benzenesulfinyl piperidine: BSP, 2,4,6-tri-*tert*-butylpyrimidine: TTBP, triflic anhydride: Tf_2_O) [60,61,62,63], where the thiophenyl-mannoside donor (**18**) was converted in situ to the corresponding anomeric α-triflate followed by the resulting triflate immediately reacting with the protected acceptor (**19**) in an S_N_2 fashion, thus producing the expected β-mannosidic bond containing disaccharide. In the reaction, the triflate group, being located on the α-side of the molecule, only allows the nucleophile to attack from the β-position, and due to the bimolecular nucleophilic substitution mechanism, the departure of the triflate group and the binding of the nucleophile occur simultaneously, which ideally ensures complete β-selectivity. The formation of the β-glycosidic bond was also facilitated by the usage of the 4,6-*O*-acetal protecting group, which can sterically hinder the α-side of the molecule, thus facilitating the attack of the acceptor at the β-position. In the reaction, the expected protected β-mannosidic-containing disaccharide was formed in good yield and with excellent stereoselectivity. The α-linked derivative could not be isolated from the reaction mixture.

Subsequently, the silyl ethers at C-2 and C-3 were removed by fluoride ion-induced cleavage (**23**), and the free hydroxyls were acetylated using Ac_2_O in dry pyridine, thus forming the intended final ester groups on the molecule (**24**). In this case, we also used a reductive ring-opening reaction to release the C-4′ position, in which we also examined the efficiency of the two ring-opening methods used previously. As before, in this case, the Et_3_SiH/BF_3_·Et_2_O reagent combination resulted in a better yield of the reaction.

**Scheme 3:**
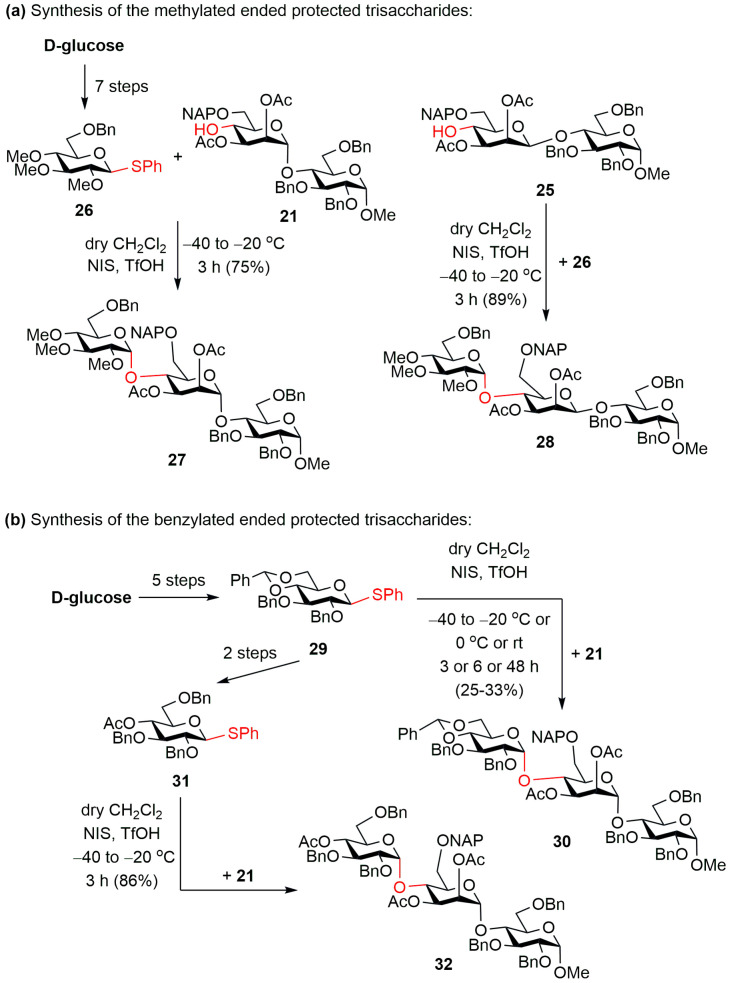
Synthesis of the (**a**) methylated-ended (**27** and **28**) and (**b**) benzylated-ended (**30** and **32**) protected trisaccharides.

The synthesis of the protected trisaccharides was carried out using two types of donors. The first terminal, non-reducing end d-glucose unit contained methyl groups at C-2, 3 and 4 (Scheme 3a). This was intended to ensure better lipid solubility and lower sulphate levels of the target compounds.

This compound (**26**) [65] was already available from our previous syntheses, so the previously prepared mannose-containing disaccharide acceptors (**21** and **25**) were glycosylated with monosaccharide donor **26** using the previously used NIS/TfOH promoter combination. In both cases, the expected protected trisaccharides (**27** and **28**) were formed in good yields and with excellent stereoselectivity.

The other monosaccharide donor contained 4,6-*O*-benzylidene acetal and benzyl ether protecting groups (**29**) [66], which we planned to provide the possibility of synthesizing the final product bearing more free –OH or more sulphate ester groups (Scheme 3b). For this, we glycosylated our α-mannosidic-linked disaccharide acceptor (**21**) with thioglucoside donor **29**. However, the expected protected trisaccharide (**30**) was formed in the reaction only in low yield. We first attempt to improve the yield by changing the reaction conditions (Table 1), changing the coupling reaction temperature and the reaction time, but unfortunately, we could not achieve a significant improvement in the reaction yield.

The low yield of the reaction was probably due to the axial position of the C-2′ group of the acceptor mannose unit, which sterically hindered the free C-4′ hydroxyl. Glycosylation of this hydroxyl with the acetal-containing donor **29**, which has a rather rigid structure and is fixed even in the case of the carbocation formed after activation, was not possible with sufficient efficiency.

To verify our theory, we performed conformational analysis and examined the possible ring conformations of the donor molecules (**26**, **29**), the carbocations that form from them and the d-mannose part of the disaccharide acceptor. We also examined the possibility of a donor with a new protecting group pattern (**31**) being suitable for carrying out a glycosylation reaction with better yield.

#### 2.1.2. In Silico Investigation of the Glycosylation of the α-Mannosidic Bond Containing Disaccharide Acceptor

Since conformational reasons were expected to stay behind the different reactivity, a conformational analysis was performed on donors **26** and **29**, their carbocations (Figure 3, **26cat**, **29cat**), and a theoretical model compound of **25** (Figure 3, **33**) [67,68]. Compound **33** is a model derivative of the disaccharide **25**. This simplification was necessary due to the size and flexibility of **25**, that would have been problematic for the calculations, so we simplified its structure For **26**, **29**, and **33**, an OPLS2005 [69] conformational search was performed in CHCl_3_ with a 21 kJ/mol energy window and the resulting 432, 981, and 1784 conformers were re-optimized first at the ωB97X/6-31G(d) [70] PCM/CH_2_Cl_2_ level followed by an ωB97X/TZVP PCM/CH_2_Cl_2_ level re-optimization. In the case of **26cat** and **29cat**, the OPLS2005 could not handle carbocations (neither did the other available MMFF, MMFFs, AMBER and OPLS force fields), thus a replacement was planned at the molecular mechanics (MM) level followed by a change back at the DFT levels, since the latter ones already can handle carbocations. Since C^+^ → Al and C^+^ → B replacements were also futile, CH^+^ → NH and CH^+^ → C=S changes were performed in parallel, and the conformational analyses were carried out in both roots (291 and 588 initial MM conformers were found for **26cat** and 834 and 876 for **29cat**).

**Figure 4:**
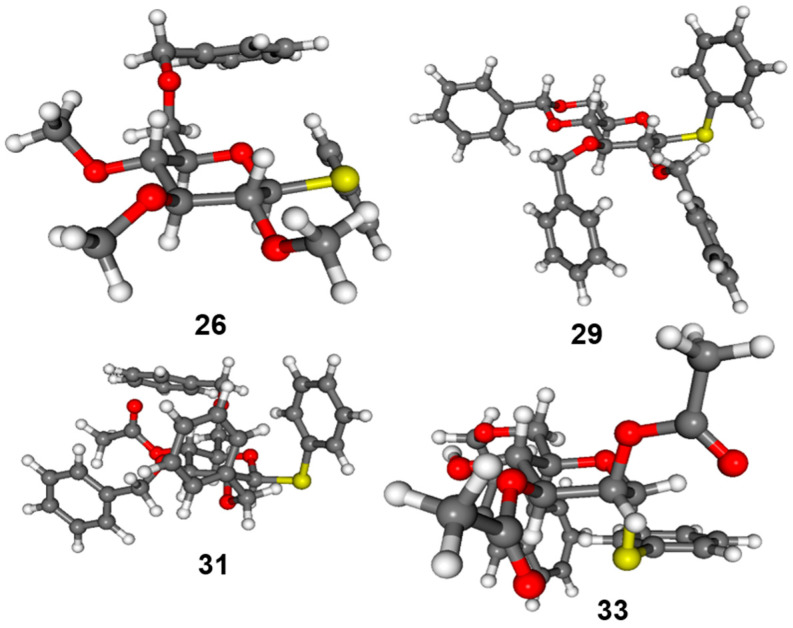
Lowest-energy conformers of donors **26**, **29**, **31**, and acceptor **33**.

In order to obtain a better coverage, the conformers from the two paths were merged after the ωB97X/TZVP PCM/CH_2_Cl_2_ level for further analyses. For the donors and the model compound of the acceptor, an expected ^4^C_1_ major conformation was obtained from the conformational analysis, and in **33**, the C-2 substituent adopted a hindering axial orientation as it was supposed to (Figure 4) [71,72].

**Figure 5:**
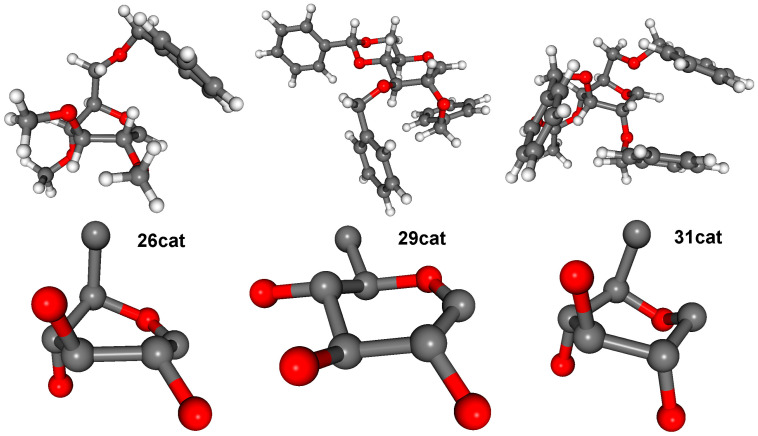
Lowest-energy conformers of cations **26cat**, **29cat**, and **31cat** (**top**) and, for better view, only the sugar ring plus the first non-hydrogen atoms (**bottom**).

**Figure 6:**
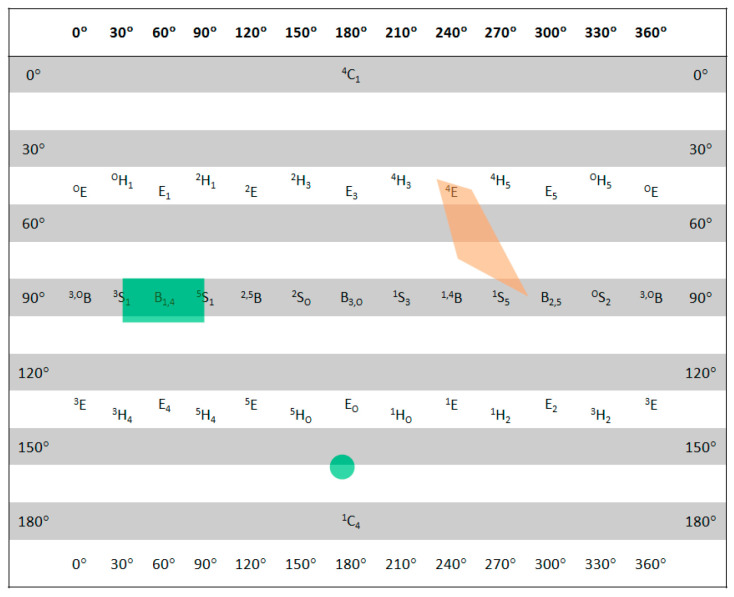
Sugar ring conformations of the low-energy structures of **29cat** (orange) and **31cat** (green) in the lowest 21 kJ/mol energy window plotted on a Cremer-Pople diagram.

The lowest-energy conformer of **26cat** adopted a conformation between ^5^S_1_ skew boat and ^5^H_4_ half chair, while that of **29cat** had a ^4^E envelope conformation (Figure 5). A reclustering of the conformers for only the sugar ring and the first connecting heavy atoms yielded 16 conformer clusters for **26cat** and only 6 for **29cat** in a 21 kJ/mol energy window, in line with the expected rigidity of the carbohydrate ring of **29** and **29cat**. Furthermore, the possible conformers of **29cat** were restricted for the ^4^E, ^1,4^B, ^1^S_5_, ^4^H_5_, and B_2,5_ range of the whole conformational space available for a flexible carbohydrate (Figure 6).

Besides the conformational factors, we also wanted to compare the stability of the carbocations. For this, we compared the energy of the carbocations to the parent donor molecules in the following way: E_carbocation_ − (E_donor_ − E_SPh_^−^). In this comparison, **26cat** showed a c.a. 17 kJ/mol lower relative energy than **29cat** that indicates also a higher stability of **26cat** being an additional factor.

A further possible donor (**31**) was planned based on flexibility and energetic considerations derived from the above calculations. While the parent donor exhibited the expected ^4^C_1_ ring conformation, the lowest-energy conformer of **31cat** showed different ring conformation (between E_O_ and ^4^C_1_) to the lowest-energy conformers of both **26cat** and **29cat**. Surprisingly, **31cat**, in contrast to the very large number of initial MM conformers (3629 and 6534), did not show high flexibility in the ring conformation, the possible range of which was, however, different from **29cat**. Relative stability of **31cat** to **29cat** was even higher than that of **26cat** with almost 40 kJ/mol.

Since preparation of **31** and its successful application was possible, our original assumptions on conformational backgrounds could be verified and refined. Carbocation **26cat** is flexible and energetically more favoured than **29cat**, while the latter can adopt only ring conformations in a narrow range, which are not convenient for the coupling. On the other hand, the sugar ring part of **31cat** is conformationally less flexible than that of **26cat** (Figure 6) but is energetically very favourable and its possible conformational range more adequate to the coupling. It is also worth noting that orientation of the substituents of **31cat** is more similar to **26cat** than those of **29cat** (Figure 5).

Based on the calculations, we were able to prepare donor **31** [73], which seemed promising in contrast to derivative **29**, based on literature examples, in two steps (reductive ring opening reaction and acetylation). Then, we glycosylated the disaccharide acceptor **21** with the new thioglucoside donor **31** under the previously used reaction conditions. The conformational calculation was confirmed. Using the more flexible donor, the expected protected trisaccharide (**32**) was formed in the reaction with excellent yield and stereoselectivity.

#### 2.1.3. Transformation of the Protected Trisaccharides

**Scheme 4:**
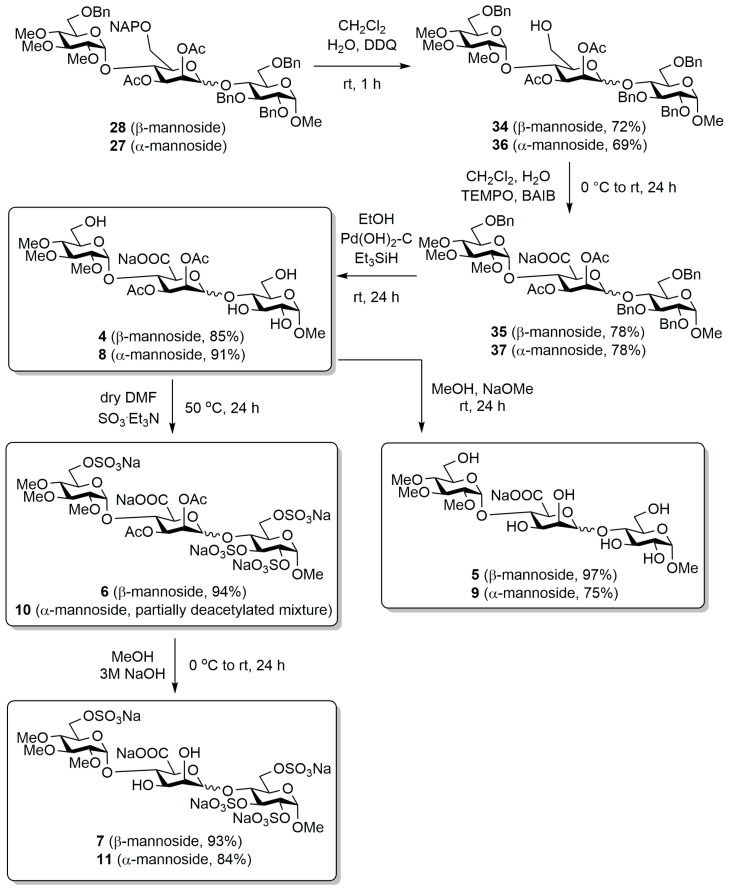
Transformation of the α- and β-mannosidic bond containing trisaccharide bearing trimethylated capping unit.

Among the protected trisaccharides, the α- and β-mannosidic bond containing molecules with the trimethyl closing unit were first converted into the intended final products (Scheme 4).

To this end, we first selectively removed the naphthylmethyl ether protecting groups at the C-6 position of the mannose units using 2,3-dichloro-5,6-dicyano-1,4-benzoquinone (DDQ) reagent under oxidative conditions (**34**, **36**) [74], and then oxidized the released primary hydroxyl using (2,2,6,6-tetramethylpiperidin-1-yl)oxyl (TEMPO) and (diacetoxyiodo)benzene (BAIB) reagents in aqueous CH_2_Cl_2_ to carboxylic acid sodium salt (**35**, **37**) [75,76]. The benzyl groups were removed from the molecules by catalytic transfer hydrogenation using Pearlmann’s catalyst [Pd(OH)_2_-C], and the necessary hydrogen was produced in situ in the reaction mixture by adding triethylsilane (Et_3_SiH) [77,78]. The reaction produced our first target compounds (**4**, **8**), which contains methyl, acetyl, and free hydroxyl groups in addition to the carboxylic function, in good yield. Subsequently, the acetyl groups were removed from compounds **4** and **8** under Zemplén conditions to obtain molecules **5** and **9**, which also contain free –OHs on the mannuronic acid unit. We also prepared the first sulphated derivatives starting from compounds **4** and **8**. For this, we introduced the sulphate ester groups onto the free hydroxyls of the molecules using SO_3_·Et_3_N complex (**6**) [79].

In the case of the β-mannoside derivative (**6**), the expected product was formed in excellent yield; however, in the case of the α-mannosidic-linked compound (**10**), unfortunately, during the processing of the reaction, acetyl group cleavage occurred as a side reaction and a mixture of partially deacetylated compounds was obtained in the reaction. The expected product could not be isolated from this mixture, so unfortunately, we could not prepare this compound in pure form and test its biological activity. Finally, by removing the acetyl groups from the mannuronic acid units of derivatives **6** and the partially deacetylated crude product **10** under alkaline conditions [79], we obtained the target compounds **7** and **11**, containing sulphate ester and –OH groups.

**Scheme 5:**
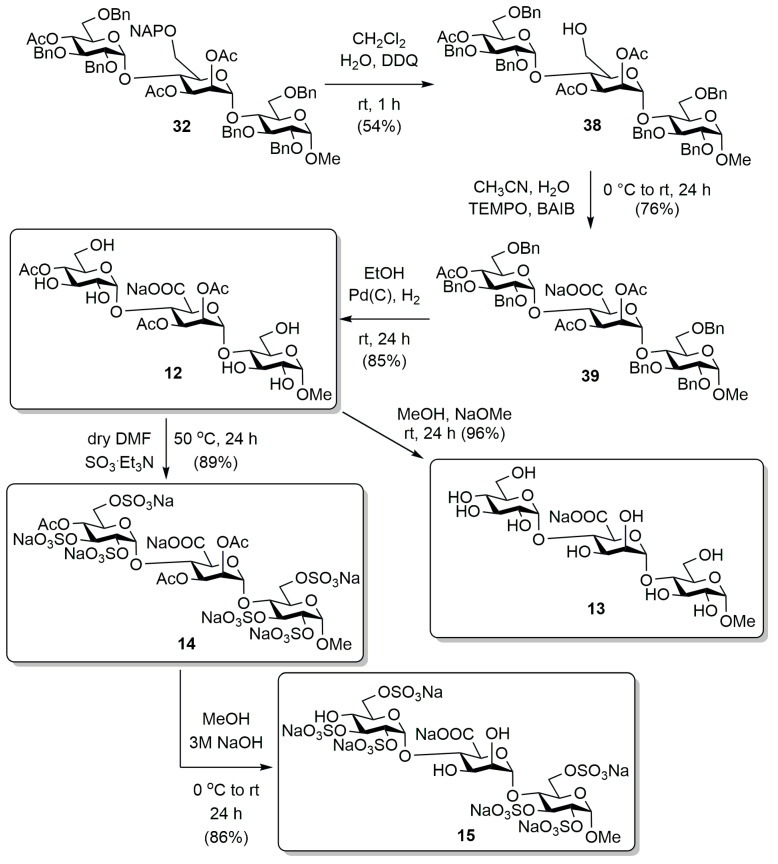
Transformation of the trisulphated-ended α-mannosidic bond containing trisaccharide.

The sulphated terminal unit-bearing trisaccharide, which containing α-mannosidic bond, was also converted to the intended target compounds using the previously presented route (Scheme 5). First, the NAP group was removed from the mannose unit under oxidative conditions (**38**), then the released primary –OH was oxidized to carboxyl function (**39**), and then the catalytic hydrogenation of the benzyl groups led to derivative **12**, bearing acetyl and hydroxyl groups. In this case, however, during the TEMPO/BAIB oxidation, due to solubility problems, CH_3_CN was used as the solvent instead of CH_2_Cl_2_. Furthermore, we had to change the conditions of the catalytic hydrogenation, since the cleavage of the benzyl groups was not complete with the method used so far. However, under pressure (in an autoclave, 10 bar) using a Pd(C) catalyst, the reaction was complete. Next, we removed the acetyl groups from a part of derivative **12** according to the Zemplén method, thus preparing the completely free mannuronic acid containing trisaccharide (**13**), and another part of compound **12** was sulphated using the previously used SO_3_·Et_3_N complex, thus synthesizing the acetyl groups containing sulphated derivative (**14**). Subsequently, we deacetylated a part of this derivative under alkaline conditions to obtain our sulphated target compound containing free –OHs (**15**).

**Figure 7:**
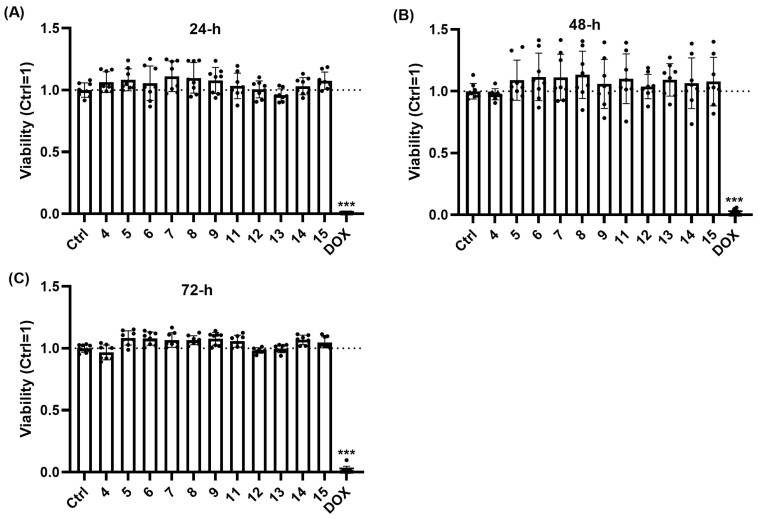
When applied at 10 μM, the tested trisaccharides do not decrease the viability of human dermal FBs. Assessment of FB viability was performed using the EZ4U cell proliferation assay. Trisaccharides were applied at a concentration of 10 µM for 24 (**A**), 48 (**B**), or 72 (**C**) hours. Data are normalized to the vehicle-treated control group and are expressed as the mean ± SD of *N* = 8 biological replicates of two donors. Doxorubicin (a well-known chemotherapeutic agent) was used as a positive control in 10 µg/mL. *** *p* < 0.001 compared to the respective vehicle-treated control group.

By transforming the protecting groups, we finally successfully synthesized eleven α- and β-mannosidic linkage containing heparan sulphate analogue trisaccharides with a diverse end-group pattern and a simplified structure, which contain d-mannuronic acid instead of d-glucuronic acid. After the synthesis, we examined the biological effect of these derivatives on primary human dermal FBs (Figure 7).

### 2.2. Biological Investigations of the Prepared Trisaccharides

To evaluate the effect of synthetized trisaccharides on FBs, we first assessed cellular viability by measuring mitochondrial enzyme activity, as detailed in the Section 3 [80]. Importantly, unlike the well-known chemotherapeutic agent doxorubicin (applied as a positive control [81]), none of the trisaccharides decreased viability of the FBs at 10 µM, even following prolonged 72 h exposure (Figure 7A–C). Thus, we concluded that they can be applied in 10 µM without the risk of cytotoxicity.

**Figure 8:**
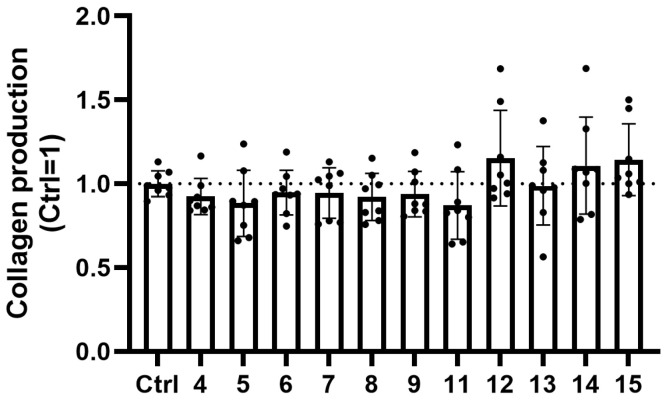
Trisaccharides do not influence collagen production of human dermal FBs over the course of 48 h treatments. Collagen production by human dermal FBs was measured using Sirius Red staining. Trisaccharides were applied at a concentration of 10 µM for 48 h. Data are normalized to the vehicle-treated (H_2_O) control group and are expressed as the mean ± SD of *N* = 8 biological replicates of two donors.

Given that collagen production is a key function of FBs and that it has central importance in ECM dynamics maintaining structural integrity, elasticity, and renewal of the skin, we investigated the effects of trisaccharides on the collagen synthesis using Sirius Red staining [82]. Collagen production was quantified following 48 h trisaccharide treatment. As shown on Figure 8, none of the tested trisaccharides modulated collagen production of human dermal FBs in a significant manner.

**Figure 9:**
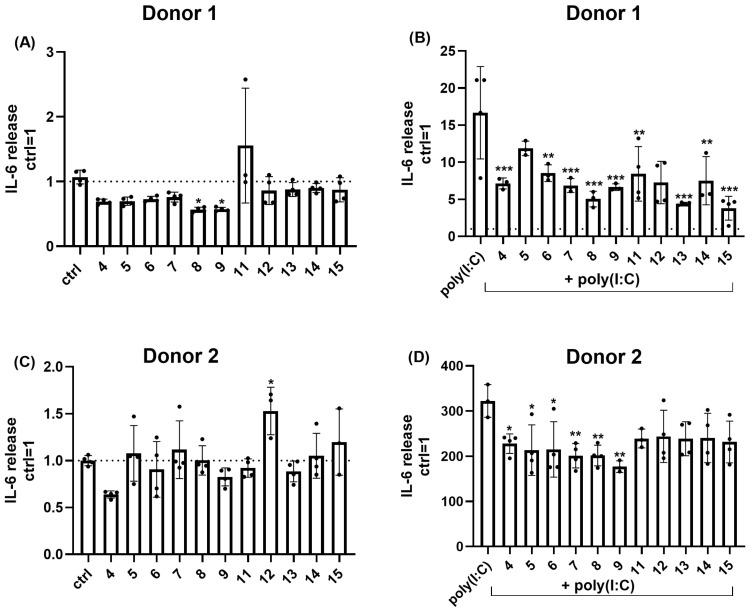
Most of the examined trisaccharides appeared to suppress poly(I:C)-induced IL-6 release of human dermal FBs. (**A**,**C**) Effect of trisaccharides (all applied at 10 µM for 24 h) on spontaneous IL-6 release of human dermal FBs. Data are normalized to the vehicle-treated (H_2_O) control group and are expressed as the mean ± SD of *N* = 3–4 biological replicates of each donor. (**B**,**D**) Effect of trisaccharides (all applied at 10 µM for 24 h) on poly(I:C)-induced IL-6 release of human dermal FBs. Data are normalized to the vehicle-treated (H_2_O) control group and are expressed as the mean ± SD of *N* = 3–4 biological replicates of each donor. Cells were pre-treated with poly(I:C) (or vehicle) for 1 h, and this was followed by a 24 h treatment using 10 µM of the trisaccharides in combination with poly(I:C). *, **, and *** *p* < 0.05, 0.01, and 0.001 compared to the respective control groups.

Because via releasing various inflammatory mediators, FBs play an important role in shaping cutaneous immune processes [83,84]. Next, we examined the release of a key pro-inflammatory cytokine (interleukin [IL]-6). Cells were treated with various trisaccharides (10 µM each) for 24 h and the impact on spontaneous IL-6 release was quantified using the ELISA technique. Interestingly, we found that the effect of the trisaccharides on the spontaneous IL-6 release exhibited significant donor-dependence. Indeed, while compound **8** and **9** decreased IL-6 release in case of Donor 1, this effect could not be reproduced in case of the cells of Donor 2. Likewise, elevation of IL-6 release in response of compound **11** (Donor 1) or compound **12** (Donor 2) could not be detected in the cells of the other donor (Figure 9).

Having investigated the effects on the spontaneous IL-6 release, next we assessed the activity of the compounds under inflammatory conditions. Inflammatory response of the FBs was evoked by the poly(I:C)-induced activation of toll-like receptor 3 (TLR3), a pathogen-associated molecular pattern recognizing receptor widely used to mimic inflammatory conditions in experimental dermatology studies (see, e.g., [85]). As shown on Figure 9, TLR3 activation significantly increased the release of IL-6, and this could be suppressed by multiple compounds (**4**, **6**, **7**, **8**, and **9**) in case of both donors, while anti-inflammatory activity of compound **5**, **11**, **13**, **14**, and **15** appeared to be donor-dependent (Figure 9).

## 3. Materials and Methods

### 3.1. Genereal Information

Optical rotations were measured at room temperature on a Perkin-Elmer 241 automatic polarimeter. TLC analysis was performed on Kieselgel 60 F_254_ (Merck) silica-gel plates with visualization by immersing in a sulphuric acid solution (5% in EtOH) followed by heating. Column chromatography was performed on silica gel 60 (Merck 0.063–0.200 mm). Organic solutions were dried over MgSO_4_ and concentrated under vacuum. ^1^H and *J*-modulated ^13^C NMR spectroscopy (^1^H: 500 and 700 MHz; ^13^C: 125.76 and 176.08 MHz) were performed on Bruker Avance II 500 and Bruker Avance NEO 700 spectrometers at 25 °C (Supplementary Materials: NMR spectra of the synthesized compounds). Chemical shifts are referenced to SiMe_4_ or sodium 3-(trimethylsilyl)-1-propanesulphonate (DSS, d = 0.00 ppm for ^1^H nuclei) and to residual solvent signals (CDCl_3_: d = 77.00 ppm, CD_3_OD: d = 49.15 ppm for ^13^C nuclei). MALDI-TOF MS measurements were carried out with a Bruker Autoflex Speed mass spectrometer equipped with a time-of-flight (TOF) mass analyser. In all cases, 19 kV (ion source voltage 1) and 16.65 kV (ion source voltage 2) were used. For reflectron mode, 21 kV and 9.55 kV were applied as reflector voltage 1 and reflector voltage 2, respectively. A solid phase laser (355 nm, ≥100 μJ/pulse) operating at 500 Hz was applied to produce laser desorption and 3000 shots were summed. 2,5-Dihydroxybenzoic acid (DHB) was used as matrix and F_3_CCOONa as cationising agent in DMF. HRMS measurements were carried out on a maXis II UHR ESI-QTOF MS instrument (Bruker, Bremen, Germany) in positive ionization mode. The following parameters were applied for the electrospray ion source: capillary voltage: 3.6 kV; end plate offset: 500 V; nebulizer pressure: 0.5 bar; dry gas temperature: 200 °C; dry gas flow rate: 4.0 L/min. Constant background correction was applied for each spectrum, and the background was recorded before each sample by injecting the blank sample matrix (solvent). Na-formate calibrant was injected after each sample, which enabled internal calibration during data evaluation. Mass spectra were recorded by otofControl version 4.1 (build: 3.5, Bruker) and processed by Compass DataAnalysis version 4.4 (build: 200.55.2969).

Mixed torsional/low-mode conformational searches were carried out by means of the MacroModel 10.8.011 software [86] using the OPLS2005 force field [69] with an implicit solvent model for CHCl_3_ applying a 21 kJ/mol energy window (Supplementary Table S1). Geometry re-optimizations of the resultant conformers (ωB97X/6-31G(d) and then ωB97X/TZVP levels, both with PCM solvent model for CH_2_Cl_2_), were performed with the Gaussian 16 package [87]. Boltzmann distributions were estimated from the ωB97X energies. Visualization of the results was performed by the MOLEKEL 5.4 software package [88]. Puckering values were generated based on the model proposed by Cremer and Pople using the Cremer–Pople Parameter Calculator [71,72].

### 3.2. Experiments on Primary Human Dermal FBs

#### 3.2.1. Isolation and Culture of Human Dermal FBs

Primary human dermal fibroblasts (FBs) were isolated from skin specimens of dermatologically healthy individuals undergoing surgical interventions following written informed consent adhering to Helsinki guidelines, and after obtaining Institutional Research Ethics Committee’s and National Public Health Centre’s permission (document ID: NNGYK/GYSZ/54991-2/2024). Full-thickness skins were placed into the transport medium containing Dulbecco’s modified essential medium (DMEM containing high glucose, GlutaMAX™ Supplement, pyruvate) supplemented with 5% penicillin-streptomycin (both from Thermo Fisher Scientific, Waltham, MA, USA). After removal of lipid layer, skin specimens were sliced into 3–5 mm^2^ squares. Epidermis was removed following an overnight incubation using 2.4 IU mL^−1^ dispase at 4 °C (Thermo Fisher Scientific, Waltham, MA, USA). After removal of epidermal sheet, dermal skin pieces were collected into the aforementioned DMEM that was supplemented by 0.25% Collagenase I, 0.05% DNase I, and 1% penicillin-streptomycin (all from Thermo Fisher Scientific, Waltham, MA, USA) for overnight incubation at 37 °C. Digestion was terminated by adding 5 × volume of normal culture medium (the aforementioned DMEM supplemented with 10% FBS, 1% penicillin-streptomycin, and 0.5% amphotericin B), and the suspension was strained using a 70 µm cell strainer (Corning, New York, NY, USA). The cell suspension was subsequently seeded into and cultured in d = 10 cm tissue culture dishes (TPP Techno Plastic Products AG, Trasadingen, Switzerland) in the same medium at 37 °C in a humidified, 5% CO_2_-containing atmosphere. Medium was changed every other day. When cultures reached 80–90% confluence, they were either sub-cultured (and used for the appropriate assays) or cryopreserved under liquid nitrogen for later use. Only FBs below passage number 6 were used for the experiments.

#### 3.2.2. Viability

Cellular viability was assessed by measuring mitochondrial enzyme activity. Mitochondrial fitness was investigated via the conversion of a non-toxic tetrazolium compound into a soluble formazan dye by mitochondrial dehydrogenases using EZ4U (Biomedica, Wien, Austria) assay following the manufacturer’s protocol [80]. Briefly, cells were plated in 96-well cell culture plates, cultured for 24 h and treated with various compounds for 24, 48 and 72 h. Following the treatment, 20 µL of the tetrazolium substrate containing working solution was added to 200 µL phenol red-free culturing medium in each well and was incubated for additional two hours. After incubation, the absorbance of the formazan product was measured at 450 nm (reference wavelength: 620 nm) using a FlexStation 3 microplate reader (Molecular Devices, Sunnyvale, CA, USA). Absorbance values measured at 620 nm were subtracted from the value measured at 450 nm. These corrected values were then normalized to the mean value of the vehicle-treated control group (positive control: Doxorubicin [10 µg/mL; Merck KGaA, Darmstadt, Germany]).

#### 3.2.3. Collagen Production

Collagen production was assessed by Sirius Red staining [82]. Briefly, cells were plated in 96-well cell culture plates. On the next day, the medium was changed to a serum-free culture medium for 24 h. After this serum starvation, the cells were treated with the test agents for 48 h in serum-free culture medium. After the removal of the culture medium, the cells were washed with 200 µL/well of phosphate-buffered saline (PBS) and fixed with 50 µL/well of Kahle’s solution (26% ethanol, 3.7% formaldehyde, and 2% glacial acetic acid) at room temperature for 15 min. After another washing step (200 µL/well PBS), cells were stained by adding 50 µL of 0.1% Sirius Red (Direct Red 80) solution dissolved in 1% acetic acid. Next, the plates were incubated at room temperature for 1 h, after which the wells were washed with 400 µL/well 0.1 M HCl. Finally, 100 µL 0.1 M NaOH solution was added to each well to eluate the bound dye from the cell-associated collagens. All chemicals were obtained from Merck KGAA (Darmstadt, Germany). Absorbance of the eluted product was measured at 540 nm using a FlexStation 3 microplate reader (Molecular Devices). Data were normalized to the mean of the vehicle-treated control group.

#### 3.2.4. Measurement of IL-6 Release

Supernatants were collected from FBs cultures exposed to various treatments for 24 h and analysed for human IL-6 using a commercially available ELISA kit (BD Biosciences, Franklin Lakes, NJ, USA) according to the manufacturer’s protocol. In brief, plates were coated using a specific capture antibody diluted in coating buffer (0.1 M Na_2_CO_3_; pH was adjusted to 9.5 using 10 M NaOH) and incubated overnight at 4 °C. On the other day, the plates were incubated with assay diluent (10% FBS in PBS) at room temperature (RT) for 1 h, while dilutions of the standards and the samples were prepared in assay diluent. The standards and the samples were then pipetted into the appropriate wells, and were incubated for 2 h at RT. After the incubation, detection antibody and SAv-HRP reagent were added to each well, and the plates were incubated for 1 h at RT. Between each step, the plates were washed with wash buffer (0.05% Tween-20 in PBS). After washing, substrate solution (tetramethylbenzidine and hydrogen peroxide in citrate-buffer, pH 5.0) was added to every well for 30 min in the dark, followed by stop solution (1M H_2_SO_4_). Absorbance was measured at 405 nm within 30 min using a FlexStation 3 microplate reader (Molecular Devices). The concentration of IL-6 was calculated with the help of a calibration curve created by serial dilution of IL-6 standards, using third order polynomial (cubic) interpolation in GraphPad Prism 10.3.1 (GraphPad Prism, LLC, San Diego, CA, USA). Finally, data were normalized to the vehicle-treated control group. To mimic inflammatory conditions, the TLR3 activator polyinosinic:polycytidylic acid (poly(I:C), InvivoGen, San Diego, CA, USA) was applied for 1 h as pre-treatment, then in combination with trisaccharides for 24 h at 10 µg/mL.

#### 3.2.5. Statistical Analysis

During the experiments, treated and non-treated samples originated from the same primary cultures were compared; therefore, no randomization was applied. Data were analysed and graphs were plotted by GraphPad Prism 10.3.1 (GraphPad Prism Software Boston, MA, USA). Depending on the sample size, Gaussian distribution was tested by Anderson–Darling or Shapiro–Wilk tests. In case of Gaussian distribution, one-way ANOVA followed by Dunnett’s multiple comparisons test (comparison to a single control group) were used, whereas in case of non-Gaussian distribution, Kruskal–Wallis test followed by Dunn’s multiple comparisons test were used, and *p* < 0.05 values were regarded as significant differences.

### 3.3. General Methods

General method A for glycosylation reactions using NIS/TfOH activator combinations (**20**, **27**, **28**, **30** and **32**)

To a solution of the appropriate 4-OH mono- and disaccharide acceptor (**19** [64], **21** and **25**) (2.293 mmol) and monosaccharide donor (**17**, **26** [65], **29** [66] and **31** [73]) (3.439 mmol, 1.5 equiv.) in dry CH_2_Cl_2_ (50.0 mL), 4 Å MS (50 piece) were added and the reaction mixture was stirred at room temperature under argon. After 30 min, the stirred mixture was cooled to −40 °C under argon and at this temperature, NIS (5.159 mmol, 1.5 equiv. to the donor) and TfOH (1.548 mmol, 0.3 equiv. to NIS), dissolved in dry THF (2.0 mL), were added. The temperature was allowed to warm up to −20 °C (**27**, **28** and **32**) or to 0 °C (**20**) or room temperature (**30**), and the reaction mixture was stirred for 3 h (**20**, **27**, **28** and **32**) or 48 h (**30**). Then, the reaction mixture was quenched with Et_3_N (330 µL), diluted with CH_2_Cl_2_ (330 mL), filtered, and the mixture was washed with saturated aqueous solution of Na_2_S_2_O_3_ (2 × 75 mL), saturated aqueous solution of NaHCO_3_ (75 mL), and H_2_O (2 × 75 mL) until neutral pH. The organic layer was dried over MgSO_4_ and concentrated.

General method B for regioselective ring opening reactions using Me_3_N·BH_3_/AlCl_3_ (**21**, **25**)

To a solution of the appropriate 4,6-*O*-acetals (**20** and **24**) (0.715 mmol) in dry THF (5.0 mL), 4 Å MS (12 pieces) and Me_3_N·BH_3_ (4.292 mmol, 6.0 equiv.) were added, and the reaction mixture was stirred for 30 min at room temperature. After 30 min, the reaction mixture was cooled to 0 °C, AlCl_3_ (4.292 mmol, 6.0 equiv.) was added, and the mixture was stirred at room temperature for 2 h (**24**) or 6 h (**20**). After that, the reaction mixture was diluted with CH_2_Cl_2_ (150 mL) and washed with H_2_O (4 × 20 mL). The organic layer was dried over MgSO_4_, filtered and concentrated.

General method C for regioselective ring opening reaction using BF_3_·Et_2_O/Et_3_SiH (**21**, **25** and **31**)

The appropriate 4,6-*O*-acetals (**20**, **24** and **29**) (0.735 mmol) was dissolved in anhydrous CH_2_Cl_2_ (8.0 mL), then 4 Å MS (~20 pieces) were added, and the solution was stirred at room temperature for 30 min. After 30 min, the mixture was cooled to 0 °C, then Et_3_SiH (1.41 mL, 8.820 mmol, 12.0 equiv.) and BF_3_·Et_2_O (181 μL, 1.469 mmol, 2.0 equiv.) were added. The reaction mixture was stirred at 0 °C for 2.5 h (**21** and **31**) or 3 h (**25**). Then, the mixture was diluted with CH_2_Cl_2_ (75 mL), washed with water (25 mL), saturated aqueous solution of NaHCO_3_ (2 × 25 mL), and water (2 × 25 mL) until neutral pH. The organic layer was dried over MgSO_4_, filtered and concentrated.

General method D for removal of the NAP-ether using DDQ (**34**, **36** and **38**)

The NAP-ether containing compounds (**27**, **28** and **32**) (0.358 mmol) was dissolved in the mixture of CH_2_Cl_2_ (6.0 mL) and water (600 μL), then DDQ was added (0.537 mmol, 1.5 equiv./OH) and the reaction mixture was stirred for 1 h at room temperature. After 1 h, the reaction mixture was diluted with CH_2_Cl_2_ (50 mL) and washed with saturated aqueous solution of NaHCO_3_ (2 × 15 mL) as well as with water (2 × 15 mL) until neutral pH. The organic layer was dried over MgSO_4_, filtered and concentrated.

General method E for oxidation of the C-6′-OH with TEMPO/BAIB (**35**, **37** and **39**)

Trisaccharide **34**, **36** and **38** (0.259 mmol) was dissolved in CH_2_Cl_2_ (5.0 mL) (**34**, **36**) or in CH_3_CN (5.0 mL), (**38**) and H_2_O (2.5 mL), TEMPO (7.0 mg, 0.047 mmol, 0.18 equiv./OH), and BAIB (250 mg, 0.777 mmol, 3.0 equiv./OH) were added at room temperature. The solution was stirred vigorously for 24 h at room temperature. After 24 h, the reaction mixture was quenched by the addition of 10% aqueous solution of Na_2_S_2_O_3_ (20 mL) and extracted with CH_2_Cl_2_ (3 × 50 mL), and the combined organic layers were dried over MgSO_4_, filtered and all volatiles were evaporated.

General method F for removal of benzyl groups by catalytic hydrogenation (**4** and **8**)

To a solution of the oxidized products (**35** and **37**) (0.192 mmol), abs. EtOH (9.0 mL) were added 20% Pd(OH)_2_-C (81.0 mg) and Et_3_SiH (460 µL, 2.280 mmol, 15.0 equiv.), and the reaction mixture was stirred at room temperature for 24 h. After 24 h, the reaction mixture was filtered through a pad of Celite, washed with MeOH, and concentrated under reduced pressure.

General method G for Zemplén deacetylation (**5**, **9** and **13**)

To a stirred solution of the appropriate trisaccharides (**4**, **8** and **12**) (0.058 mmol) in MeOH (3.0 mL), NaOMe (20 mg) was added and the reaction mixture was stirred for 24 h at room temperature. After 24 h, the mixture was neutralized with Amberlite IR 120 H^+^ resin, filtered, washed with MeOH and concentrated.

General method H for introduction of the sulphate ester groups (**6**, **10** and **14**)

SO_3_·Et_3_N complex (1.320 mmol, 5.0 equiv./-OH) was added to a solution of the appropriate trisaccharides poliols (**4**, **8**, **12**) (0.066 mmol) in dry DMF (3.6 mL). The reaction mixture was stirred at 50 °C for 24 h. Subsequently, the reaction was quenched with NaHCO_3_ solution (6.625 mmol, 5.0 equiv. to the complex), and the mixture was concentrated under reduced pressure.

General method I for the alkaline hydrolysis of acetyl groups (**7**, **11** and **15**)

To a solution of the appropriate sulphated trisaccharides (**6**, **10** and **14**) (0.041 mmol), in MeOH (2.00 mL), a solution of NaOH (3M, 900 μL) was added at 0 °C. The reaction mixture was allowed to warm up to room temperature and stirred for 24 h. Subsequently, the reaction mixture was neutralized with AcOH solution (60%), and the mixture was concentrated under reduced pressure.

### 3.4. Synthesis of the Mannuronic Acid Containing Trisaccharides

Methyl (2,3,4-tri-*O*-methyl-α-d-glucopyranosyl)-(1→4)-(sodium 2,3-di-*O*-acetyl-β-d-mannopyranosyl-uronate)-(1→4)-α-d-glucopyranoside (**4**)

Compound **35** (200 mg, 0.192 mmol) was converted to **4** according to general method F. The crude product was purified by silica gel chromatography (1:1 CH_2_Cl_2_/MeOH) to give compound **4** (110 mg, 85%) as a white foam. *R*_f_ 0.37 (1:1 CH_2_Cl_2_/MeOH); ${\left[\alpha \right]}_{D}^{25}$ + 124.0 (*c* 0.10, H_2_O); ^1^H NMR (700 MHz, D_2_O) *δ* = 5.30 (d, *J* = 3.6 Hz, 1H, H-1′), 5.25–5.23 (m, 2H, H-1″, H-3′), 5.18 (t, *J* = 3.7 Hz, 1H, H-2′), 4.72 (d, *J* = 3.8 Hz, 1H, H-1), 4.28 (t, *J* = 7.2 Hz, 1H, H-4′), 4.20 (d, *J* = 6.8 Hz, 1H, H-5′), 3.84 (dd, *J* = 3.9 Hz, *J* = 12.3 Hz, 1H, H-6a), 3.76 (d, *J* = 12.8 Hz, 1H, H-6b), 3.75–3.70 (m, 2H, H-3, H-6″a), 3.67–3.64 (m, 3H, H-5, H-5″, H-6″b), 3.57 (t, *J* = 9.5 Hz, 1H, H-4), 3.53 (s, 3H, OC*H*_3_), 3.50–3.48 (m, 2H, H-2, H-3″), 3.47 (s, 3H, OC*H*_3_), 3.41 (s, 3H, OC*H*_3_), 3.33 (s, 3H, C-1-OC*H*_3_), 3.24 (dd, *J* = 3.7 Hz, *J* = 10.0 Hz, 1H, H-2″), 3.21 (t, *J* = 9.7 Hz, 1H, H-4″), 2.08 (s, 3H, Ac-C*H*_3_), 2.03 (s, 3H, Ac-C*H*_3_) ppm; ^13^C NMR (176 MHz, D_2_O) *δ* = 175.4 (1C, *C*OONa), 173.9, 173.7 (2C, 2 × C_q_ Ac), 100.1 (1C, C-1), 99.4 (1C, C-1′), 97.2 (1C, C-1″), 82.9 (1C, C-3″), 81.1 (1C, C-2″), 79.3 (1C, C-4″), 78.8 (1C, C-4), 75.8 (1C, C-5′), 74.0 (1C, C-3), 72.9 (1C, C-4′), 72.2 (2C, C-2, C-3′), 71.8 (1C, C-5″), 71.2 (1C, C-5), 70.4 (1C, C-2′), 61.4 (1C, C-6), 61.1 (1C, O*C*H_3_), 60.6 (1C, C-6″), 60.5, 59.6 (2C, 2 × O*C*H_3_), 56.0 (1C, C-1-O*C*H_3_), 21.3, 21.2 (2C, 2 × Ac-*C*H_3_) ppm; UHR ESI-QTOF (positive ion): *m*/*z* calcd. for [M + H]^+^ C_26_H_42_NaO_19_ 681.2212, found: 681.2209.

Methyl (2,3,4-tri-*O*-methyl-α-d-glucopyranosyl)-(1→4)-(sodium β-d-mannopyranosyl-uronate)-(1→4)-α-d-glucopyranoside (**5**)

Compound **4** (40 mg, 0.058 mmol) was converted to **5** according to general method G. The crude product was purified by silica gel chromatography (1:1 CH_2_Cl_2_/MeOH) to give compound **5** (34 mg, 97%) as a white foam. *R*_f_ 0.18 (1:1 CH_2_Cl_2_/MeOH); ${\left[\alpha \right]}_{D}^{25}$ + 149.2 (*c* 0.13, H_2_O); ^1^H NMR (700 MHz, D_2_O) *δ* = 5.43 (d, *J* = 3.6 Hz, 1H, H-1″), 5.20 (d, *J* = 3.7 Hz, 1H, H-1′), 4.69 (d, *J* = 3.8 Hz, 1H, H-1), 4.17 (d, *J* = 7.4 Hz, 1H, H-5′), 4.06 (t, *J* = 7.5 Hz, 1H, H-4′), 3.96 (dd, *J* = 3.1 Hz, *J* = 7.7 Hz, 1H, H-3′), 3.82 (t, *J* = 3.4 Hz, 1H, H-2′), 3.74–3.72 (m, 2H, H-6a,b), 3.71 (t, *J* = 9.4 Hz, 1H, H-3), 3.67 (dd, *J* = 1.9 Hz, *J* = 12.4 Hz, 1H, H-6″a), 3.63 (dd, *J* = 3.4 Hz, *J* = 12.5 Hz, 1H, H-6″b), 3.61 (dd, *J* = 3.2 Hz, *J* = 6.9 Hz, 1H, H-5), 3.56 (dt, *J* = 3.0 Hz, *J* = 10.2 Hz, 1H, H-5″), 3.53 (t, *J* = 9.6 Hz, 1H, H-4), 3.50 (s, 3H, OC*H*_3_), 3.47 (dd, *J* = 3.7 Hz, *J* = 9.9 Hz, 1H, H-2), 3.44 (s, 3H, OC*H*_3_), 3.43 (t, *J* = 9.0 Hz, 1H, H-3″), 3.41 (s, 3H, OC*H*_3_), 3.30 (s, 3H, C-1-OC*H*_3_), 3.22 (dd, *J* = 3.7 Hz, *J* = 10.0 Hz, 1H, H-2″), 3.19 (t, *J* = 9.7 Hz, 1H, H-4″) ppm; ^13^C NMR (176 MHz, D_2_O) *δ* = 175.2 (1C, *C*OONa), 102.1 (1C, C-1′), 100.1 (1C, C-1), 97.0 (1C, C-1″), 82.5 (1C, C-3″), 81.3 (1C, C-2″), 79.4 (1C, C-4″), 78.5 (1C, C-4), 75.4 (1C, C-4′), 74.6 (1C, C-5′), 74.3 (1C, C-3), 72.1 (1C, C-2), 71.8 (1C, C-5″), 71.4 (1C, C-3′), 71.2 (1C, C-5), 71.0 (1C, C-2′), 61.5 (1C, C-6), 61.0 (1C, O*C*H_3_), 60.6 (1C, C-6″), 60.6, 58.9 (2C, 2 × O*C*H_3_), 56.0 (1C, C-1-O*C*H_3_) ppm; UHR ESI-QTOF (positive ion): *m*/*z* calcd. for [M + H]^+^ C_22_H_38_NaO_17_ 597.2001, found: 597.2002.

Penta-sodium [methyl (2,3,4-tri-*O*-methyl-6-*O*-sulphonato-α-d-glucopyranosyl)-(1→4)-(2,3-di-*O*-acetyl-β-d-mannopyranosyl-uronate)-(1→4)-2,3,6-tri-*O*-sulphonato-α-d-glucopyranoside (**6**)

Compound **4** (45 mg, 0.066 mmol) was converted to **6** according to general method H. The crude product was purified by Sephadex G-25 gel chromatography in H_2_O and transformed to Na^+^ salt using Dowex Na^+^ ion exchange resin to give **6** (68 mg, 94%) as a white solid. *R*_f_ 0.28 (7:6:1 CH_2_Cl_2_/MeOH/H_2_O); ${\left[\alpha \right]}_{D}^{25}$ + 98.6 (*c* 0.07, H_2_O); ^1^H NMR (700 MHz, D_2_O) *δ* = 5.52 (t, *J* = 2.6 Hz, 1H, H-2′), 5.32 (d, *J* = 2.0 Hz, 1H, H-1′), 5.25–5.24 (m, 2H, H-1″, H-3′), 5.12 (d, *J* = 3.5 Hz, 1H, H-1), 4.71–4.68 (m, 1H, H-3), 4.37 (dd, *J* = 1.8 Hz, *J* = 11.3 Hz, 1H, H-6a), 4.33 (dd, *J* = 3.6 Hz, *J* = 9.9 Hz, 1H, H-2), 4.26–4.24 (m, 1H, H-6″a), 4.23–4.20 (m, 2H, H-4′, H-6b), 4.13–4.11 (m, 2H, H-5′, H-6″b), 4.06–4.04 (m, 1H, H-5), 3.93–3.90 (m, 2H, H-4, H-5″), 3.59 (s, 3H, OC*H*_3_), 3.54 (s, 3H, OC*H*_3_), 3.53–3.52 (m, 1H, H-3″), 3.45 (s, 6H, 2 × OC*H*_3_), 3.30–3.27 (m, 2H, H-2″, H-4″), 2.14, 2.07 (2 × s, 6H, 2 × Ac-C*H*_3_) ppm; ^13^C NMR (176 MHz, D_2_O) *δ* = 175.1 (1C, *C*OONa), 173.5, 173.4 (2C, 2 × C_q_ Ac), 99.9 (1C, C-1′), 97.8 (1C, C-1), 97.7 (1C, C-1″), 82.9 (1C, C-3″), 80.9 (1C, C-2″), 79.3 (1C, C-3), 78.9 (1C, C-4″), 76.6 (1C, C-4), 75.8 (1C, C-2), 75.2 (1C, C-5′), 72.8 (1C, C-3′), 72.6 (1C, C-4′), 70.7 (1C, C-2′), 69.7 (1C, C-5″), 69.0 (1C, C-5), 68.0 (1C, C-6), 66.9 (1C, C-6″), 61.0, 60.8, 59.9 (3C, 3 × O*C*H_3_), 56.2 (1C, C-1-O*C*H_3_), 21.4, 21.1 (2C, 2 × Ac-*C*H_3_) ppm; UHR ESI-QTOF (positive ion): *m*/*z* calcd. for [M + 2Na]^2+^ C_26_H_37_Na_7_O_31_S_4_ 566.9737, found: 566.9737.

Penta-sodium [methyl (2,3,4-tri-*O*-methyl-6-*O*-sulphonato-α-d-glucopyranosyl)-(1→4)-(β-d-mannopyranosyl-uronate)-(1→4)-2,3,6-tri-*O*-sulphonato-α-d-glucopyranoside (**7**)

Compound **6** (45 mg, 0.041 mmol) was converted to **7** according to general method I. The crude product was purified by Sephadex G-25 gel chromatography in H_2_O and transformed to Na^+^ salt using Dowex Na^+^ ion exchange resin to produce **7** (38 mg, 93%) as a white solid. *R*_f_ 0.61 (6:6:1 CH_2_Cl_2_/MeOH/H_2_O); ${\left[\alpha \right]}_{D}^{25}$ + 28.2 (*c* 0.16, H_2_O); ^1^H NMR (700 MHz, D_2_O) *δ* = 5.50 (d, *J* = 3.6 Hz, 1H, H-1″), 5.11 (d, *J* = 2.1 Hz, 1H, H-1′), 5.07 (d, *J* = 3.4 Hz, 1H, H-1), 4.59 (d, *J* = 9.3 Hz, 1H, H-3), 4.30 (dd, *J* = 2.3 Hz, *J* = 11.4 Hz, 1H, H-6″a), 4.28 (dd, *J* = 3.6 Hz, *J* = 10.0 Hz, 1H, H-2), 4.20 (dd, *J* = 2.8 Hz, *J* = 10.4 Hz, 2H, H-6a, H-6″b), 4.08–4.07 (m, 2H, H-2′, H-6b), 3.97–3.95 (m, 4H, H-3′, H-4′, H-5′, H-5), 3.84 (d, *J* = 10.1 Hz, 1H, H-5″), 3.81 (t, *J* = 9.3 Hz, 1H, H-4), 3.53 (s, 3H, OC*H*_3_), 3.51–3.48 (m, 4H, H-3″, OC*H*_3_), 3.45 (s, 3H, OC*H*_3_), 3.39 (s, 3H, C-1-OC*H*_3_), 3.25 (t, *J* = 9.8 Hz, 1H, H-4″), 3.24 (dd, *J* = 4.0 Hz, *J* = 9.8 Hz, 1H, H-2″) ppm; ^13^C NMR (176 MHz, D_2_O) *δ* = 182.3 (1C, *C*OONa), 103.2 (1C, C-1′), 97.9 (1C, C-1), 96.9 (1C, C-1″), 82.3 (1C, C-3″), 81.1 (1C, C-2″), 79.0 (2C, C-3, C-4″), 77.7 (1C, C-4), 75.9 (1C, C-2), 75.3, 74.7 (2C, C-3′, C-4′), 71.7 (1C, C-5′), 71.3 (1C, C-2′), 69.6 (1C, C-5″), 69.5 (1C, C-5), 68.0 (1C, C-6″), 67.0 (1C, C-6), 61.0, 60.8, 58.9 (3C, 3 × O*C*H_3_), 56.2 (1C, C-1-O*C*H_3_) ppm; UHR ESI-QTOF (positive ion): *m*/*z* calcd. for [M + 2Na]^2+^ C_22_H_33_Na_7_O_29_S_4_ 524.9632, found: 524.9628.

Methyl (2,3,4-tri-*O*-methyl-α-d-glucopyranosyl)-(1→4)-(sodium 2,3-di-*O*-acetyl-α-d-mannopyranosyl-uronate)-(1→4)-α-d-glucopyranoside (**8**)

Compound **37** (407 mg, 0.391 mmol) was converted to **8** according to general method F. The crude product was purified by silica gel chromatography (1:1 CH_2_Cl_2_/MeOH) to produce compound **8** (241 mg, 91%) as a white foam. *R*_f_ 0.41 (1:1 CH_2_Cl_2_/MeOH); ${\left[\alpha \right]}_{D}^{25}$ + 116.0 (*c* 0.10, H_2_O); ^1^H NMR (700 MHz, D_2_O) *δ* = 5.34 (d, *J* = 4.1 Hz, 1H, H-1′), 5.28–5.26 (m, 2H, H-1″, H-3′), 5.19 (t, *J* = 3.5 Hz, 1H, H-2′), 4.73 (d, *J* = 3.7 Hz, 1H, H-1), 4.32–4.30 (m, 2H, H-4′, H-5′), 3.83 (dd, *J* = 4.4 Hz, *J* = 12.4 Hz, 1H, H-6a), 3.78 (dd, *J* = 1.8 Hz, *J* = 12.3 Hz, 1H, H-6b), 3.75 (t, *J* = 9.4 Hz, 1H, H-3), 3.72 (dd, *J* = 2.0 Hz, *J* = 12.5 Hz, 1H, H-6″a), 3.69–3.67 (m, 2H, H-5, H-6″b), 3.63–3.58 (m, 2H, H-4, H-5″), 3.55 (s, 3H, OC*H*_3_), 3.51–3.48 (m, 2H, H-2, H-3″), 3.48 (s, 3H, OC*H*_3_), 3.43 (s, 3H, OC*H*_3_), 3.34 (s, 3H, C-1-OC*H*_3_), 3.26 (dd, *J* = 3.8 Hz, *J* = 10.0 Hz, 1H, H-2″), 3.23 (t, *J* = 9.7 Hz, 1H, H-4″), 2.09 (s, 3H, Ac-C*H*_3_), 2.05 (s, 3H, Ac-C*H*_3_) ppm; ^13^C NMR (176 MHz, D_2_O) *δ* = 173.7 (1C, *C*OONa), 172.9, 172.7 (2C, 2 × C_q_ Ac), 99.1 (1C, C-1), 98.3 (1C, C-1′), 96.4 (1C, C-1″), 82.0 (1C, C-3″), 80.1 (1C, C-2″), 78.3 (1C, C-4″), 77.7 (1C, C-4), 74.2 (1C, C-4′), 73.0 (1C, C-3), 71.8 (1C, C-5′), 71.3 (1C, C-2), 71.0 (2C, C-3′, C-5″), 70.1 (1C, C-5), 69.3 (1C, C-2′), 60.4 (1C, C-6), 60.1 (1C, O*C*H_3_), 59.7 (1C, O*C*H_3_), 59.6 (1C, C-6″), 58.7 (1C, O*C*H_3_), 55.0 (1C, C-1-O*C*H_3_), 20.4, 20.2 (2C, 2 × Ac-*C*H_3_) ppm; UHR ESI-QTOF (positive ion): *m*/*z* calcd. for [M + H]^+^ C_26_H_42_NaO_19_ 681.2212, found: 681.2211.

Methyl (2,3,4-tri-*O*-methyl-α-d-glucopyranosyl)-(1→4)-(sodium α-d-mannopyranosyl-uronate)-(1→4)-α-d-glucopyranoside (**9**)

Compound **8** (42 mg, 0.062 mmol) was converted to **9** according to general method G. The crude product was purified by silica gel chromatography (1:1 CH_2_Cl_2_/MeOH) to produce compound **9** (28 mg, 75%) as a white foam. *R*_f_ 0.35 (1:1 CH_2_Cl_2_/MeOH); ${\left[\alpha \right]}_{D}^{25}$ + 255.4 (*c* 0.11, H_2_O); ^1^H NMR (700 MHz, D_2_O) *δ* = 5.45 (d, *J* = 3.6 Hz, 1H, H-1″), 5.28 (d, *J* = 4.3 Hz, 1H, H-1′), 4.72 (d, *J* = 3.7 Hz, 1H, H-1), 4.40 (d, *J* = 6.5 Hz, 1H, H-5′), 4.14 (t, *J* = 6.9 Hz, 1H, H-4′), 4.04 (dd, *J* = 3.1 Hz, *J* = 7.1 Hz, 1H, H-3′), 3.85–3.84 (m, 1H, H-2′), 3.80–3.73 (m, 3H, H-3, H-6a,b), 3.71–3.64 (m, 3H, H-5, H-6″a,b), 3.58 (t, *J* = 9.8 Hz, 1H, H-4), 3.52 (s, 3H, OC*H*_3_), 3.51–3.49 (m, 2H, H-2, H-5″), 3.47 (s, 3H, OC*H*_3_), 3.43 (s, 3H, OC*H*_3_), 3.42–3.39 (m, 1H, H-3″), 3.33 (s, 3H, C-1-OC*H*_3_), 3.27–3.22 (m, 2H, H-2″, H-4″) ppm; ^13^C NMR (176 MHz, D_2_O) *δ* = 173.7 (1C, *C*OONa), 101.6 (1C, C-1′), 100.1 (1C, C-1), 97.2 (1C, C-1″), 82.7 (1C, C-3″), 81.1 (1C, C-2″), 79.4 (1C, C-4″), 78.2 (1C, C-4), 75.6 (1C, C-4′), 74.2 (1C, C-3), 73.6 (1C, C-5′), 72.1 (2C, C-2, C-5″), 71.1 (2C, C-3′, C-5), 70.6 (1C, C-2′), 61.5 (1C, C-6), 61.1 (1C, O*C*H_3_), 60.8 (1C, O*C*H_3_), 60.6 (1C, C-6″), 59.0 (1C, O*C*H_3_), 56.1 (1C, C-1-O*C*H_3_) ppm; UHR ESI-QTOF (positive ion): *m*/*z* calcd. for [M + H]^+^ C_22_H_38_NaO_17_ 597.2001, found: 597.1999.

Penta-sodium [methyl (2,3,4-tri-*O*-methyl-6-*O*-sulphonato-α-d-glucopyranosyl)-(1→4)-(α-d-mannopyranosyl-uronate)-(1→4)-2,3,6-tri-*O*-sulphonato-α-d-glucopyranoside (**11**)

Compound **8** (60 mg, 0.088 mmol) was converted to **10** according to general method H. The crude product was purified by Sephadex G-25 gel chromatography in H_2_O and transformed to Na^+^ salt using Dowex Na^+^ ion exchange resin to produce **10** (56 mg, mixture of partially deacetylated compounds) as a white solid. UHR ESI-QTOF (positive ion): *m*/*z* calcd for [M + 2Na]^2+^ C_26_H_37_Na_7_O_31_S_4_ 566.9737, found: 566.9732. The partially deacetylated mixture of compound **10** (56 mg, 0.051 mmol) was converted to **11** according to general method I. The crude product was purified by Sephadex G-25 gel chromatography in H_2_O and transformed to Na^+^ salt using Dowex Na^+^ ion exchange resin to produce **11** (44 mg, 84% for two steps) as a white solid. *R*_f_ 0.74 (6:7:1 CH_2_Cl_2_/MeOH/H_2_O); ${\left[\alpha \right]}_{D}^{25}$ + 55.6 (*c* 0.16, H_2_O); ^1^H NMR (700 MHz, D_2_O) *δ* = 5.54 (d, *J* = 3.5 Hz, 1H, H-1″), 5.16 (s, 1H, H-1′), 5.11 (d, *J* = 3.3 Hz, 1H, H-1), 4.63 (t, *J* = 9.3 Hz, 1H, H-3), 4.34–4.31 (m, 2H, H-2, H-6a), 4.24–4.23 (m, 2H, H-6b, H-6″a), 4.13–4.11 (m, 2H, H-2′, H-6″b), 4.01–3.99 (m, 4H, H-3′, H-4′, H-5′, H-5), 3.89–3.88 (m, 1H, H-5″), 3.85 (t, *J* = 9.3 Hz, 1H, H-4), 3.57 (s, 3H, OC*H*_3_), 3.53 (s, 3H, OC*H*_3_), 3.55–3.52 (m, 1H, H-3″), 3.49 (s, 3H, OC*H*_3_), 3.43 (s, 3H, C-1-OC*H*_3_), 3.30–3.27 (m, 2H, H-2″, H-4″) ppm; ^13^C NMR (176 MHz, D_2_O) *δ* = 176.2 (1C, *C*OONa), 103.2 (1C, C-1′), 97.9 (1C, C-1), 96.9 (1C, C-1″), 82.3 (1C, C-3″), 81.1 (1C, C-2″), 78.9 (2C, C-3, C-4″), 77.7 (1C, C-4), 75.9 (1C, C-2), 75.3 (1C, C-4′), 74.7 (1C, C-5′), 71.7 (1C, C-3′), 71.3 (1C, C-2′), 69.5 (2C, C-5, C-5″), 68.0 (1C, C-6), 67.0 (1C, C-6″), 61.0, 60.8, 58.9 (3C, 3 × O*C*H_3_), 56.2 (1C, C-1-O*C*H_3_) ppm; UHR ESI-QTOF (positive ion): *m*/*z* calcd. for [M + 2Na]^2+^ C_22_H_33_Na_7_O_29_S_4_ 524.9632, found: 524.9630.

Methyl (4-*O*-acetyl-α-d-glucopyranosyl)-(1→4)-(sodium 2,3-di-*O*-acetyl-α-d-mannopyranosyl-uronate)-(1→4)-α-d-glucopyranoside (**12**)

A mixture of compound **39** (264 mg, 0.216 mmol), which was dissolved in EtOH/AcOH (96%, 29:1, 14.0 mL), and Pd/C (10%, 173 mg) was stirred in an autoclave under a H_2_ atmosphere (10 bar) for 24 h. After 24 h, the catalyst was filtered through a pad of Celite^®^, washed with MeOH, and the filtrate was concentrated. The crude product was purified by silica gel chromatography (1:1 CH_2_Cl_2_/MeOH) to give compound **12** (125 mg, 85%) as a white solid. *R*_f_ 0.44 (1:1 CH_2_Cl_2_/MeOH); ${\left[\alpha \right]}_{D}^{25}$ + 172.8 (*c* 0.09, H_2_O); ^1^H NMR (700 MHz, D_2_O) *δ* = 5.30 (d, *J* = 3.7 Hz, 1H, H-1′), 5.25 (dd, *J* = 3.2 Hz, *J* = 7.3 Hz, 1H, H-3′), 5.21 (t, *J* = 3.4 Hz, 1H, H-2′), 5.15 (d, *J* = 3.9 Hz, 1H, H-1″), 4.76 (d, *J* = 9.8 Hz, 1H, H-4″), 4.72 (d, *J* = 3.9 Hz, 1H, H-1), 4.28–4.23 (m, 2H, H-4′, H-5′), 3.86 (dt, *J* = 3.2 Hz, *J* = 10.7 Hz, 1H, H-5″), 3.83–3.77 (m, 3H, H-3″, H-6a,b), 3.74 (t, *J* = 9.5 Hz, 1H, H-3), 3.66 (ddd, *J* = 2.2 Hz, *J* = 4.2 Hz, *J* = 9.9 Hz, 1H, H-5), 3.62–3.56 (m, 2H, H-4, H-6″a), 3.51–3.47 (m, 3H, H-2, H-2″, H-6″b), 3.34 (s, 3H, C-1-OC*H*_3_), 2.09, 2.08, 2.02 (3 × s, 9H, 3 × Ac-C*H*_3_) ppm; ^13^C NMR (176 MHz, D_2_O) *δ* = 175.3 (1C, *C*OONa), 174.1, 173.8, 173.7 (3C, 3 × C_q_ Ac), 100.1 (1C, C-1), 100.0 (1C, C-1′), 99.5 (1C, C-1″), 78.6 (1C, C-4), 75.4 (1C, C-5′), 74.0 (1C, C-3), 73.7 (1C, C-4′), 72.3 (2C, C-2, C-3′), 72.0 (1C, C-2″), 71.7 (2C, C-3″, C-4″), 71.2 (1C, C-5), 70.7 (1C, C-5″), 70.5 (1C, C-2′), 61.4 (1C, C-6), 60.7 (1C, C-6″), 55.9 (1C, C-1-O*C*H_3_), 21.3, 21.2 (3C, 3 × Ac-*C*H_3_) ppm; UHR ESI-QTOF (positive ion): *m*/*z* calcd. for [M + H]^+^ C_25_H_38_NaO_20_ 681.1849, found: 681.1848.

Methyl (α-d-glucopyranosyl)-(1→4)-(sodium α-d-mannopyranosyl-uronate)-(1→4)-α-d-glucopyranoside (**13**)

Compound **12** (35 mg, 0.051 mmol) was converted to **13** according to general method G. The crude product was purified by silica gel chromatography (1:1 CH_2_Cl_2_/MeOH) to give compound **13** (27 mg, 96%) as a white foam. *R*_f_ 0.38 (1:1 CH_2_Cl_2_/MeOH); ${\left[\alpha \right]}_{D}^{25}$ + 205.8 (*c* 0.12, H_2_O); ^1^H NMR (700 MHz, D_2_O) *δ* = 5.28 (d, *J* = 2.9 Hz, 1H, H-1′), 5.20 (s, 1H, H-1″), 4.71 (s, 1H, H-1), 4.45 (d, *J* = 4.7 Hz, 1H, H-5′), 4.14 (t, *J* = 5.0 Hz, 1H, H-4′), 4.08–4.06 (m, 1H, H-3′), 3.87–3.85 (m, 1H, H-2′), 3.81–3.75 (m, 3H, H-3, H-6a,b), 3.71–3.69 (m, 2H, H-6″a,b), 3.65–3.59 (m, 4H, H-3″, H-4, H-5, H-5″), 3.52–3.50 (m, 1H, H-2), 3.48–3.46 (m, 1H, H-2″), 3.36 (t, *J* = 9.3 Hz, 1H, H-4″), 3.33 (s, 3H, C-1-OC*H*_3_) ppm; ^13^C NMR (176 MHz, D_2_O) *δ* = 172.6 (1C, *C*OONa), 100.5 (1C, C-1′), 99.2 (1C, C-1), 99.1 (1C, C-1″), 77.5 (1C, C-3″), 75.7 (1C, C-4′), 73.1 (1C, C-3), 72.9 (1C, C-5′), 72.7 (1C, C-5), 72.3 (1C, C-5″), 71.4 (1C, C-2″), 71.1 (1C, C-2), 70.1 (1C, C-4), 69.7 (1C, C-3′), 69.3 (1C, C-2′), 69.1 (1C, C-4″), 60.6 (1C, C-6), 60.1 (1C, C-6″), 55.0 (1C, C-1-O*C*H_3_) ppm; UHR ESI-QTOF (positive ion): *m*/*z* calcd. for [M + H]^+^ C_19_H_32_NaO_17_ 555.1532, found: 555.1532.

Hepta-sodium [methyl (4-*O*-acetyl-2,3,6-tri-*O*-sulphonato-α-d-glucopyranosyl)-(1→4)-(2,3-di-*O*-acetyl-α-d-mannopyranosyl-uronate)-(1→4)-2,3,6-tri-*O*-sulphonato-α-d-glucopyranoside (**14**)

Compound **12** (52 mg, 0.076 mmol) was converted to **14** according to general method H. The crude product was purified by Sephadex G-25 gel chromatography in H_2_O and transformed to Na^+^ salt using Dowex Na^+^ ion exchange resin to give **14** (96 mg, 89%) as a white solid. *R*_f_ 0.63 (6:7:1 CH_2_Cl_2_/MeOH/H_2_O); ${\left[\alpha \right]}_{D}^{25}$ + 94.2 (*c* 0.12, H_2_O); ^1^H NMR (700 MHz, D_2_O) *δ* = 5.53 (t, *J* = 2.7 Hz, 1H, H-3′), 5.51 (d, *J* = 3.7 Hz, 1H, H-1″), 5.30 (d, *J* = 2.3 Hz, 1H, H-1′), 5.27 (dd, *J* = 3.1 Hz, *J* = 8.9 Hz, 1H, H-2′), 5.11 (d, *J* = 3.5 Hz, 1H, H-1), 5.09 (t, *J* = 9.8 Hz, 1H, H-4″), 4.68 (t, *J* = 9.4 Hz, 1H, H-3), 4.59 (t, *J* = 9.7 Hz, 1H, H-3″), 4.37 (dd, *J* = 1.8 Hz, *J* = 11.5 Hz, 1H, H-6a), 4.31 (dd, *J* = 3.5 Hz, *J* = 9.9 Hz, 1H, H-2), 4.29–4.23 (m, 3H, H-4′, H-5′, H-6a), 4.20–4.18 (m, 2H, H-2″, H-5″), 4.15 (dd, *J* = 2.0 Hz, *J* = 11.4 Hz, 1H, H-6″a), 4.09 (dd, *J* = 1.6 Hz, *J* = 11.6 Hz, 1H, H-6″b), 4.04 (ddd, *J* = 1.9 Hz, *J* = 5.6 Hz, *J* = 9.7 Hz, 1H, H-5), 3.92 (t, *J* = 9.2 Hz, 1H, H-4), 3.43 (s, 3H, C-1-OC*H*_3_), 2.11, 2.06, 2.02 (3 × s, 9H, 3 × Ac-C*H*_3_) ppm; ^13^C NMR (176 MHz, D_2_O) *δ* = 174.3 (1C, *C*OONa), 173.1, 172.7 (3C, 3 × C_q_ Ac), 99.1 (1C, C-1′), 96.9 (1C, C-1), 96.7 (1C, C-1″), 78.5 (1C, C-3), 75.9 (2C, C-3″, C-4), 75.0 (1C, C-2), 74.6 (1C, C-2″), 74.4 (1C, C-4′), 71.8 (2C, C-2′, C-5′), 69.7 (1C, C-3′), 68.2 (1C, C-5), 67.8 (1C, C-4″), 67.7 (1C, C-5″), 67.2 (1C, C-6), 65.5 (1C, C-6″), 55.4 (1C, C-1-O*C*H_3_), 20.9, 20.6, 20.3 (3C, 3 × Ac-*C*H_3_) ppm; UHR ESI-QTOF (positive ion): *m*/*z* calcd. for [M + 2Na]^2+^ C_25_H_31_Na_9_O_38_S_6_ 668.8943, found: 668.8943.

Hepta-sodium [methyl (2,3,6-tri-*O*-sulphonato-α-d-glucopyranosyl)-(1→4)-(α-d-mannopyranosyl-uronate)-(1→4)-2,3,6-tri-*O*-sulphonato-α-d-glucopyranoside (**15**)

Compound **14** (48 mg, 0.037 mmol) was converted to **15** according to general method I. The crude product was purified by Sephadex G-25 gel chromatography in H_2_O and transformed to Na^+^ salt using Dowex Na^+^ ion exchange resin to give **15** (37 mg, 86%) as a white solid. *R*_f_ 0.41 (6:7:1 CH_2_Cl_2_/MeOH/H_2_O); ${\left[\alpha \right]}_{D}^{25}$ + 64.0 (*c* 0.10, H_2_O); ^1^H NMR (700 MHz, D_2_O) *δ* = 5.60 (d, *J* = 3.7 Hz, 1H, H-1″), 5.16 (d, *J* = 2.1 Hz, 1H, H-1′), 5.09 (d, *J* = 3.5 Hz, 1H, H-1), 4.61 (t, *J* = 9.3 Hz, 1H, H-3), 4.52 (t, *J* = 9.5 Hz, 1H, H-3″), 4.38 (dd, *J* = 2.1 Hz, *J* = 11.8 Hz, 1H, H-6a), 4.33–4.29 (m, 2H, H-2, H-6″a), 4.23–4.21 (m, 2H, H-2″, H-6b), 4.16–4.14 (m, 1H, H-6″b), 4.08–4.07 (m, 4H, H-2′, H-3′, H-4′, H-5′), 4.01–3.98 (m, 1H, H-5), 3.97–3.95 (m, 1H, H-5″), 3.86 (t, *J* = 9.3 Hz, 1H, H-4), 3.71 (t, *J* = 9.6 Hz, 1H, H-4″), 3.40 (s, 3H, C-1-OC*H*_3_) ppm; ^13^C NMR (176 MHz, D_2_O) *δ* = 175.1 (1C, *C*OONa), 101.8 (1C, C-1′), 97.0 (2C, C-1, C-1″), 78.4 (1C, C-3″), 78.0 (1C, C-3), 76.7 (1C, C-4), 75.6 (1C, C-4′), 75.1 (1C, C-2″), 75.0 (1C, C-2), 74.7 (1C, C-5′), 70.1 (2C, C-2′, C-3′), 69.7 (1C, C-5″), 68.6 (1C, C-5), 67.9 (1C, C-4″), 67.2 (1C, C-6), 66.1 (1C, C-6″), 55.3 (1C, C-1-O*C*H_3_) ppm; UHR ESI-QTOF (positive ion): *m*/*z* calcd. for [M + 2Na]^2+^ C_19_H_25_Na_9_O_35_S_6_ 605.8784, found: 605.8784.

Phenyl 2,3-di-*O*-acetyl-4,6-*O*-(2-naphthyl)methylidene-1-thio-α-d-mannopyranoside (**17**)

To a stirred solution of compound **16** [58] (13.2 g, 0.032 mol) in dry pyridine (200 mL), Ac_2_O (80.0 mL, 0.845 mol, 26.3 equiv.) was added at 0 °C and the reaction mixture was stirred for 24 h at room temperature. After 24 h, all volatiles were removed by reduced pressure and the residue was dissolved in CH_2_Cl_2_ (500 mL), washed with H_2_O (2 × 250 mL), 1M aqueous solution of H_2_SO_4_ (2 × 250 mL), H_2_O (2 × 250 mL), saturated aqueous solution of NaHCO_3_ (2 × 250 mL), and H_2_O (2 × 250 mL) until neutral pH. The organic layer was dried over MgSO_4_, filtered, and concentrated under reduced pressure. The crude product was purified by silica gel chromatography (6:4 *n*-hexane/EtOAc) to give **17** (11,24 g, 71%) as a colourless syrup. *R*_f_ 0.52 (6:4 *n*-hexane/EtOAc); ${\left[\alpha \right]}_{D}^{25}$ + 96.0 (*c* 0.30, CHCl_3_); ^1^H NMR (500 MHz, CDCl_3_) *δ* = 7.95–7.23 (m, 12H, arom.), 5.75 (s, 1H, H_ac_), 5.64 (dd, *J* = 1.3 Hz, *J* = 3.4 Hz, 1H, H-2), 5.47–5.45 (m, 2H, H-1, H-3), 4.53 (dt, *J* = 5.0 Hz, *J* = 9.9, Hz, 1H, H-5), 4.30 (dd, *J* = 4.9 Hz, *J* = 10.4 Hz, 1H, H-6a), 4.20 (t, *J* = 10.0 Hz, 1H, H-4), 3.93 (t, *J* = 10.3 Hz, 1H, H-6b), 2.17, 2.03 (2 × s, 6H, 2 × Ac-C*H*_3_) ppm; ^13^C NMR (125 MHz, CDCl_3_) *δ* = 169.9, 169.8 (2C, 2 × Ac-*C*=O), 134.5, 133.8, 133.0, 132.9 (4C, 4 × C_q_ arom.), 132.4–123.9 (12C, arom.), 102.3, (1C, C_ac_), 87.0 (1C, C-1), 76.4 (1C, C-4), 71.6 (1C, C-2), 68.7 (1C, C-3), 68.6 (1C, C-6), 65.3 (1C, C-5), 21.0, 20.9 (2C, 2 × Ac-*C*H_3_) ppm; UHR ESI-QTOF (positive ion): *m*/*z* calcd. for [M + Na]^+^ C_27_H_26_NaO_7_S 517.1291, found: 517.1291.

Phenyl 2,3-di-*O*-(*tert*-buthyl-dimethyl-silyl)-4,6-*O*-(2-naphthyl)methylidene-1-thio-α-d-mannopyranoside (**18**)

To a cooled (−20 °C) solution of **16** [58] (2.0 g, 4.877 mmol) in dry CH_2_Cl_2_ (40.7 mL), Et_3_N (5.64 mL, 40.48 mmol, 8.3 equiv.) and TBDMSOTf (5.6 mL, 24.38 mmol, 5.0 equiv.) were added. The mixture was allowed to warm up to room temperature and stirred for 3 h. After 3 h, CH_2_Cl_2_ (113 mL) and water (56 mL) were added and stirred for 15 min. After separation of the phases, the organic layer was dried over MgSO_4_ and filtered, and the filtrate was evaporated. The crude product was purified by silica gel chromatography (98:2 *n*-hexane/acetone) to produce **18** (3.01 g, 96%) as a colourless syrup. *R*_f_ 0.52 (1:1 CH_2_Cl_2_/*n*-hexane); ${\left[\alpha \right]}_{D}^{25}$ + 116.7 (*c* 0.15, CHCl_3_); ^1^H NMR (500 MHz, CDCl_3_) *δ* = 7.95–7.18 (m, 12H, arom.), 5.66 (s, 1H, H_ac_), 5.32 (s, 1H, H-1), 4.29 (dt, *J* = 4.9 Hz, *J* = 9.4 Hz, 1H, H-5), 4.21 (dd, *J* = 4.6 Hz, *J* = 10.0 Hz, 1H, H-6a), 4.14 (bs, 1H, H-2), 4.10 (dd, *J* = 2.6 Hz, *J* = 9.1 Hz, 1H, H-3), 4.05 (t, *J* = 9.1 Hz, 1H, H-4), 3.82 (t, *J* = 10.1 Hz, 1H, H-6b), 0.90, 0.86 (2 × s, 18H, 6 × *t*-Bu-C*H*_3_), 0.11, 0.07, 0.05, 0.04 (4 × s, 12H, 4 × Si-C*H*_3_) ppm; ^13^C NMR (125 MHz, CDCl_3_) *δ* = 135.2, 134.2, 133.7, 133.1 (4C, 4 × C_q_ arom.), 131.9–124.2 (12C, arom.), 102.3, (1C, C_ac_), 90.5 (1C, C-1), 79.5 (1C, C-4), 75.3 (1C, C-2), 70.8 (1C, C-3), 68.9 (1C, C-6), 66.0 (1C, C-5), 26.2 (5C, 5 × Si-*C*H_3_), 25.9 (5C, 5 × Si-*C*H_3_), 18.6, 18.2 (2C, 2 × C_q_ Si-*t*-Bu) ppm; UHR ESI-QTOF (positive ion): *m*/*z* calcd. for [M + Na]^+^ C_35_H_50_NaO_5_SSi_2_ 661.2810, found: 661.2809.

Methyl [2,3-di-*O*-acetyl-4,6-*O*-(2-naphthyl)methylidene-α-d-mannopyranosyl]-(1→4)-2,3,6-tri-*O*-benzyl-α-d-glucopyranoside (**20**)

Compound **19** [64] (5.09 g, 10.96 mmol) was glycosylated with **17** (8.13 g, 16.435 mmol, 1.5 equiv.) according to general method A. The crude product was purified by silica gel chromatography (98:2 CH_2_Cl_2_/EtOAc) to produce **20** (8.59 g, 92%) as a colourless syrup. *R*_f_ 0.32 (98:2 CH_2_Cl_2_/EtOAc); ${\left[\alpha \right]}_{D}^{25}$ + 32.5 (*c* 0.36, CHCl_3_); ^1^H NMR (500 MHz, CDCl_3_) *δ* = 7.91–7.22 (m, 22H, arom.), 5.68 (s, 1H, H_ac_), 5.42–5.41 (m, 2H, H-1′, H-2′), 5.37 (dd, *J* = 2.9 Hz, *J* = 9.9 Hz, 1H, H-3′), 5.06 (d, *J* = 11.2 Hz, 1H, Bn-C*H*_2_a), 4.72–4.56 (m, 6H, 2 × Bn-C*H*_2_, Bn-C*H*_2_b, H-1), 4.13 (dd, *J* = 4.4 Hz, *J* = 10.3 Hz, 1H, H-5′), 4.06–3.98 (m, 3H, H-3, H-4′, H-5), 3.95 (t, *J* = 9.0 Hz, 1H, H-4), 3.84–3.76 (m, 3H, H-6′a,b, H-6a), 3.71–3.70 (m, 1H, H-6b), 3.56 (dd, *J* = 3.4 Hz, *J* = 9.5 Hz, 1H, H-2), 3.39 (s, 3H, C-1OC*H_3_*), 1.99, 1.96 (2 × s, 6H, 2 × Ac-C*H*_3_) ppm; ^13^C NMR (125 MHz, CDCl_3_) *δ* = 170.0, 169.5 (2C, 2 × C_q_ Ac), 138.6, 138.1, 138.0, 134.6, 133.8, 132.9 (6C, 6 × C_q_ arom.), 128.6–123.9 (22C, arom.), 102.1, (1C, C_ac_), 99.7 (1C, C-1′), 97.9 (1C, C-1), 81.9 (1C, C-3), 80.3 (1C, C-2), 76.1 (1C, C-4′), 75.2, (1C, Bn-*C*H_2_), 74.7 (1C, C-4), 73.7, 73.3 (2C, 2 × Bn-*C*H_2_), 70.2 (1C, C-2′), 69.4 (1C, C-5′), 69.0, 68.7 (2C, C-6, C-6′), 68.5 (1C, C-3′), 64.9 (1C, C-5), 55.4 (1C, C-1-O*C*H_3_), 20.9, 20.7 (2C, 2 × Ac-*C*H_3_) ppm; UHR ESI-QTOF (positive ion): m/z calcd. for [C_49_H_52_O_13_ + Na]^+^: 871.3300; found: 871.3297.

Methyl [2,3-di-*O*-acetyl-4,6-*O*-(2-naphthyl)methylidene-α-d-mannopyranosyl]-(1→4)-2,3,6-tri-*O*-benzyl-α-d-glucopyranoside (**21**)

Reaction 1.: Compound **20** (623 mg, 0.735 mmol) was converted to **21** by general method B. The crude product was purified by silica gel chromatography (6:4 *n*-hexane/EtOAc) to produce compound **21** (463 mg, 66%) as a white foam.

Reaction 2.: Compound **20** (607 mg, 0.715 mmol) was converted to **21** by general method C. The crude product was purified by silica gel chromatography (55:45 *n*-hexane/EtOAc, then 6:4 hexane/acetone) to produce compound **21** (439 mg, 72%) as a white foam. *R*_f_ 0.44 (6:4 *n*-hexane/EtOAc); ${\left[\alpha \right]}_{D}^{25}$ + 32.4 (*c* 0.33, CHCl_3_); ^1^H NMR (500 MHz, CDCl_3_) *δ* = 7.81–7.22 (m, 22H, arom.), 5.39 (s, 1H, H-1′), 5.28 (bs, 1H, H-2′), 5.12 (dd, *J* = 3.1 Hz, *J* = 9.9 Hz, 1H, H-3′), 5.04 (d, *J* = 11.1 Hz, 1H, Bn-C*H*_2_a), 4.71–4.45 (m, 8H, NAP-C*H*_2_, 2 × Bn-C*H*_2_, Bn-C*H*_2_b, H-1), 3.99–3.96 (m, 2H, H-3, H-4′), 3.86 (t, *J* = 9.1 Hz, 1H, H-4), 3.80–3.74 (m, 3H, H-5, H-5′, H-6a), 3.68–3.64 (m, 2H, H-6b, H-6′a), 3.57–3.52 (m, 2H, H-2, H-6′b), 3.38 (s, 3H, C-1OC*H_3_*), 2.60 (s, 1H, H-4-O*H*), 2.02, 1.91 (2 × s, 6H, 2 × Ac-C*H*_3_) ppm; ^13^C NMR (125 MHz, CDCl_3_) *δ* = 170.7, 169.6 (2C, 2 × C_q_ Ac), 138.6, 138.3, 138.0, 135.2, 133.3, 133.1 (6C, 6 × C_q_ arom.), 128.5–125.7 (22C, arom.), 98.9 (1C, C-1′), 97.9 (1C, C-1), 81.8 (1C, C-3), 80.2 (1C, C-2), 75.2, (1C, Bn-*C*H_2_), 75.1 (1C, C-4′), 73.8, 73.3 (3C, NAP-*C*H_2_, 2 × Bn-*C*H_2_), 71.8, 71.7 (2C, C-3′, C-4), 70.3 (1C, C-6′), 69.7 (1C, C-5′), 69.5 (1C, C-2′), 69.1 (1C, C-6), 67.2 (1C, C-5), 55.4 (1C, C-1-O*C*H_3_), 21.0, 20.7 (2C, 2 × Ac-*C*H_3_) ppm; UHR ESI-QTOF (positive ion): *m*/*z* calcd. for [C_49_H_54_O_13_ + Na]^+^: 873.3457; found: 873.3458.

Methyl [2,3-di-*O*-(*tert*-buthyl-dimethyl-silyl)-4,6-*O*-(2-naphthyl)methylidene-β-d-mannopyranosyl]-(1→4)-2,3,6-tri-*O*-benzyl-α-d-glucopyranoside (**22**)

A mixture of the thiomannosyl donor **18** (193 mg, 0.302 mmol), BSP (76 mg, 0.362 mmol, 1.2 equiv.), TTBP (150 mg, 0.604 mmol, 2 equiv.), and 4 Å molecular sieves (419 mg) in dry CH_2_Cl_2_ (4.2 mL) was stirred under an atmosphere of Argon for 1 h. The reaction was cooled to −60 °C and Tf_2_O (61 µL, 0.362 mmol, 1.2 equiv.) was added. After 30 min of stirring at −60 °C, a solution of the acceptor **19** [64] (88 mg, 0.189 mmol, 1.0 equiv.) in dry CH_2_Cl_2_ (1.4 mL) was added. The reaction mixture was stirred for further 3 h, at −60 °C. After 3 h, the reaction mixture was quenched by the addition of pyridine (745 μL). The mixture was filtered, and the filtrate was washed with saturated aqueous solution of NaHCO_3_ (25 mL) and brine (25 mL). The organic phase was dried (MgSO_4_) and filtered, and the filtrate was concentrated under reduced pressure. The crude product was purified by silica gel chromatography (CH_2_Cl_2_) to produce **22** (131 mg, 68%) as a white foam. *R*_f_ 0.41 (8:2 *n*-hexane/acetone); ${\left[\alpha \right]}_{D}^{25}$ – 1.82 (*c* 0.11, CHCl_3_); ^1^H NMR (500 MHz, CDCl_3_) *δ* = 7.49–6.71 (m, 22H, arom.), 5.21 (s, 1H, H_ac_), 4.51 (s, 1H, H-1′), 4.29 (d, *J* = 11.6 Hz, 1H, Bn-C*H*_2_a), 4.15 (d, *J* = 12.0 Hz, 3H, Bn-C*H*_2_, H-1), 4.05 (dd, *J* = 18.0 Hz, *J* = 11.8 Hz, 3H, Bn-C*H*_2_b, Bn-C*H*_2_), 3.65 (ddd, *J* = 3.0 Hz, *J* = 9.8 Hz, *J* = 16.5, 2H, H-6′a, H-3′), 3.53 (t, *J* = 9.3 Hz, 1H, H-4′), 3.47–3.37 (m, 3H, H-2′, H-3, H-5′), 3.36–3.26 (m, 5H, H-6′a, H-6a,b, H-5, H-4), 3.10 (dd, *J* = 3.5 Hz, *J* = 9.5, 1H, H-2), 2.91 (s, 3H, OC*H*_3_), 0.38 (s, 18H, 6 × C*H*_3_ Si-*t*-Bu), −0.43 (s, 3H, Si-C*H*_3_), −0.44 (s, 3H, Si-C*H*_3_), −0.53 (s, 3H, Si-C*H*_3_), −0.65 (s, 3H, Si-C*H*_3_) ppm; ^13^C NMR (125 MHz, CDCl_3_) *δ* = 139.0, 138.2, 138.1, 135.3, 133.7, 133.0 (6C, C_q_ arom.), 128.5–124.2 (22C, arom.), 104.3 (1C, C-1′), 102.3 (1C, C_ac_), 97.9 (1C, C-1), 81.3 (1C, C-2′), 80.2 (1C, C-2), 79.6 (1C, C-4′), 78.4 (1C, C-4), 74.9 (1C, Bn-*C*H_2_), 74.2 (1C, C-3), 73.8, 73.3 (2C, 2 × Bn-*C*H_2_), 70.0 (1C, C-3′), 69.7 (1C, C-5), 69.2 (2C, C-6, C-6′), 65.6 (1C, C-5′), 55.4 (1C, O*C*H_3_), 26.2 (5C, 5 × Si-*C*H_3_), 25.9 (5C, 5 × Si-*C*H_3_), 18.6 (2C, 2 × C_q_ Si-*t*-Bu) ppm; MALDI-TOF-MS (positive ion): *m*/*z* calcd. for [M + Na]^+^ C_57_H_76_NaO_11_SSi_2_ 1015.4824, found: 1015.4819.

Methyl [4,6-*O*-(2-naphthyl)methylidene-β-d-mannopyranosyl]-(1→4)-2,3,6-tri-*O*-benzyl-α-d-glucopyranoside (**23**)

Compound **22** (1.473 g, 1.483 mmol) was dissolved in dry THF (30 mL), and the reaction mixture was cooled to 0 °C. After, 1M solution of TBAF in dry THF (5.936 mL, 5.936 mmol, 2 equiv./OH) was added and the reaction mixture was stirred for 48 h at room temperature. After 48 h, the reaction mixture was diluted with EtOAc (500 mL) and washed with water (150 mL) and brine (150 mL). The organic layer was dried over MgSO_4_ and filtered, and the filtrate was concentrated under reduced pressure. The crude product was purified by silica gel chromatography (6:4 *n*-hexane/acetone) to give **23** (813 mg, 72%) as a white crystal. *R*_f_ 0.21 (65:35 *n*-hexane/acetone); ${\left[\alpha \right]}_{D}^{25}$ + 38.9 (*c* 0.18, CHCl_3_); M.p.: 203–205 °C; ^1^H NMR (500 MHz, CDCl_3_) *δ* = 7.94–7.22 (m, 22H, arom.), 5.64 (s, 1H, H_ac_), 5.33 (s, 1H, H-1′), 5.06 (d, *J* = 11.1 Hz, 1H, Bn-C*H*_2_a), 4.72 (d, *J* = 12.0 Hz, 1H, Bn-C*H*_2_a), 4.66–4.53 (m, 4H, Bn-C*H*_2_b, H-1, BnC*H*_2_), 4.54 (d, *J* = 11.9 Hz, 1H, Bn-C*H*_2_b), 4.11 (dd, *J* = 4.5 Hz, *J* = 10.1 Hz, 1H, H-6′a), 3.96 (dd, *J* = 3.4 Hz, *J* = 9.5 Hz, 1H, H-3′), 3.94–3.89 (m, 2H, H-3, H-4′), 3.87–3.81 (m, 2H, H-2′, H-5′), 3.80 (t, *J* = 7.2 Hz, 1H, H-4), 3.76–3.69 (m, 4H, H-5, H-6a,b, H-6′b), 3.54 (dd, *J* = 3.5 Hz, *J* = 9.6 Hz, 1H, H-2), 3.39 (s, 3H, OC*H*_3_), 2.68–2.24 (m, 2H, 2 × OH) ppm; ^13^C NMR (125 MHz, CDCl_3_) *δ* = 138.6, 138.1, 138.0, 134.7, 133.8, 133.0 (6C, C_q_ arom.), 128.7–123.9 (22C, arom.), 102.3 (1C, C_ac_), 102.0 (1C, C-1′), 97.9 (1C, C-1), 82.1 (1C, C-3), 80.3 (1C, C-2), 78.9 (1C, C-4′), 76.3 (1C, C-4), 75.8, 73.8, 73.3 (3C, 3 × Bn*C*H_2_), 71.4 (1C, C-2′), 69.7 (1C, C-5), 69.2 (1C, C-6), 68.8 (1C, C-6′), 68.5 (1C, C-3′), 64.2 (1C, C-5′), 55.4 (1C, O*C*H_3_) ppm; UHR ESI-QTOF (positive ion): *m*/*z* calcd. for [M + Na]^+^ C_45_H_48_NaO_11_ 787.3089, found: 787.3083.

Methyl [2,3-di-*O*-acetyl-4,6-*O*-(2-naphthyl)methylidene-β-d-mannopyranosyl]-(1→4)-2,3,6-tri-*O*-benzyl-α-d-glucopyranoside (**24**)

To a solution of **23** (614 mg, 0.803 mmol) in dry pyridine (4.0 mL), Ac_2_O (2.0 mL) was added at 0 °C and the reaction mixture was stirred at room temperature for 24 h. After 24 h, the reaction mixture was concentrated under reduced pressure. The crude product was purified by silica gel chromatography (6:4 *n*-hexane/acetone) to produce **24** (620 mg, 91%) as a white foam. *R*_f_ 0.52 (6:4 *n*-hexane/acetone); ${\left[\alpha \right]}_{D}^{25}$ + 44.5 (*c* 0.11, CHCl_3_); ^1^H NMR (500 MHz, CDCl_3_) *δ* = 7.91–7.21 (m, 22H, arom.), 5.68 (s, 1H, H_ac_), 5.43 (s, 2H, H-1′, H-2′), 5.38 (d, *J* = 10.0 Hz, 1H, H-3′), 5.07 (d, *J* = 11.2 Hz, 1H, Bn-C*H*_2_a), 4.71–4.56 (m, 6H, Bn-C*H*_2_b, H-1, 2 × BnC*H*_2_), 4.14 (dd, *J* = 4.1 Hz, *J* = 10.2 Hz, 1H, H-6′a), 4.07–3.93 (m, 4H, H-3, H-4, H-4′, H-5′), 3.83–3.76 (m, 3H, H-5, H-6′b, H-6a), 3.70 (d, *J* = 9.2 Hz, 1H, H-6b), 3.56 (dd, *J* = 3.4 Hz, *J* = 9.4 Hz, 1H, H-2), 3.39 (s, 3H, OC*H*_3_), 1.98, 1.96 (2 × s, 6H, 2 × Ac-C*H*_3_) ppm; ^13^C NMR (125 MHz, CDCl_3_) *δ* = 170.0, 169.4 (2C, 2 × C_q_ Ac), 138.6, 138.1, 138.0, 134.6, 133.7, 132.9 (6C, C_q_ arom.), 128.5–123.8 (22C, arom.), 102.1 (1C, C_ac_), 99.6 (1C, C-1′), 97.9 (1C, C-1), 81.9 (1C, C-3), 80.2 (1C, C-2), 76.0 (1C, C-4′), 75.2 (1C, Bn-*C*H_2_), 74.7 (1C, C-4), 73.6, 73.3 (2C, 2 × Bn*C*H_2_), 70.1 (1C, C-2′), 69.4 (1C, C-5), 68.9 (1C, C-6), 68.7 (1C, C-6′), 68.5 (1C, C-3′), 64.9 (1C, C-5′), 55.4 (1C, O*C*H_3_), 20.9, 20.7 (2C, 2 × Ac-*C*H_3_) ppm; UHR ESI-QTOF (positive ion): *m*/*z* calcd. for [M + Na]^+^ C_49_H_52_NaO_13_ 871.3300, found: 871.3308.

Methyl [2,3-di-*O*-acetyl-6-*O*-(2-naphthyl)methyl-β-d-mannopyranosyl]-(1→4)-2,3,6-tri-*O*-benzyl-α-d-glucopyranoside (**25**)

Reaction 1.: Compound **24** (150 mg, 0.177 mmol) was converted to **25** by general method B. The crude product was purified by silica gel chromatography (55:45 *n*-hexane/EtOAc, then 6:4 *n*-hexane/acetone) to give compound **25** (94 mg, 62%) as a white foam.

Reaction 2.: Compound **24** (153 mg, 0.180 mmol) was converted to **25** by general method C. The crude product was purified by silica gel chromatography (65:35 *n*-hexane/acetone) to produce compound **25** (117 mg, 76%) as a white foam. *R*_f_ 0.46 (65:35 *n*-hexane/acetone); ${\left[\alpha \right]}_{D}^{25}$ + 44.5 (*c* 0.11, CHCl_3_); ^1^H NMR (500 MHz, CDCl_3_) *δ* = 7.82–7.07 (m, 22H, arom.), 5.39 (d, *J* = 1.9 Hz, 1H, H-1′), 5.30 (dd, *J* = 1.8 Hz, *J* = 3.4 Hz, 1H, H-2′), 5.13 (dd, *J* = 3.3 Hz, *J* = 9.9 Hz, 1H, H-3′), 5.03 (d, *J* = 11.1 Hz, 1H, Bn-C*H*_2_a), 4.74–4.40 (m, 8H, Bn-C*H*_2_b, 2 × Bn-C*H*_2_, NAP-C*H*_2_, H-1), 3.98 (t, *J* = 9.1 Hz, 2H, H-3, H-4′), 3.86 (t, *J* = 9.2 Hz, 1H, H-4), 3.82–3.72 (m, 3H, H-5′, H-5, H-6a), 3.71–3.61 (m, 2H, H-6b, H-6′a), 3.54 (ddd, *J* = 4.0 Hz, *J* = 9.9 Hz, *J* = 17.8 Hz, 2H, H-6′b, H-2), 3.37 (s, 3H, OC*H*_3_), 2.72 (s, 1H, H-4′-O*H*), 2.00 (s, 3H, Ac-C*H*_3_), 1.90 (s, 3H, Ac-C*H*_3_) ppm; ^13^C NMR (125 MHz, CDCl_3_) *δ* = 170.7, 169.6 (2C, 2 × C_q_ Ac), 138.5, 138.3, 138.0, 135.2, 133.2, 133.0 (6C, C_q_ arom.), 128.5–125.6 (22C, arom.), 98.9 (1C, C-1′), 97.8 (1C, C-1), 81.8 (1C, C-3), 80.2 (1C, C-2), 75.2 (1C, C-4), 75.1, 73.8, 73.2, 73.2 (4C, 3 × Bn-*C*H_2_, NAP-*C*H_2_), 71.8 (2C, C-5′, C-3′), 70.2 (1C, C-6′), 69.7 (1C, C-2′), 69.5 (1C, C-5), 69.1 (1C, C-6), 67.0 (1C, C-4′), 55.3 (1C, C-1-O*C*H_3_), 20.9, 20.7 (2C, 2 × Ac-*C*H_3_) ppm; MALDI-TOF-MS (positive ion): *m*/*z* calcd. for [M + Na]^+^ C_49_H_54_NaO_13_ 873.3462, found: 873.3461.

Methyl (6-*O*-benzyl-2,3,4-tri-*O*-methyl-α-d-glucopyranosyl)-(1→4)-[2,3-di-*O*-acetyl-6-*O*-(2-naphthyl)methyl-α-d-mannopyranosyl]-(1→4)-2,3,6-tri-*O*-benzyl-α-d-glucopyranoside (**27**)

Disaccharide acceptor **21** (1.042 g, 1.225 mmol) was glycosylated with **26** [65] (0.842 g, 2.082 mmol, 1.7 equiv.) according to general method A. The crude product was purified by silica gel chromatography (7:3 *n*-hexane/acetone) to give **27** (1.055 g, 75%) as a colourless syrup. *R*_f_ 0.49 (7:3 *n*-hexane/acetone); ${\left[\alpha \right]}_{D}^{25}$ + 40.7 (*c* 0.70, CHCl_3_); ^1^H NMR (500 MHz, CDCl_3_) *δ* = 7.80–7.13 (m, 27H, arom.), 5.43 (d, *J* = 9.3 Hz, 1H, H-3′), 5.34 (s, 1H, H-1′), 5.30–5.28 (m, 2H, H-1″, H-2′), 5.03 (d, *J* = 11.0 Hz, 1H, Bn-C*H*_2_a), 4.75 (d, *J* = 11.0 Hz, 1H, Bn-C*H*_2_b), 4.71–4.43 (m, 7H, H-1, NAP-C*H*_2_, 2 × Bn-C*H*_2_), 4.32 (d, *J* = 12.1 Hz, 1H, Bn-C*H*_2_a), 4.21 (t, *J* = 9.3 Hz, 1H, H-4′), 4.10 (d, *J* = 12.1 Hz, 1H, Bn-C*H*_2_b), 3.99–3.93 (m, 2H, H-3, H-5′), 3.83 (t, *J* = 9.0 Hz, 1H, H-4), 3.78–3.75 (m, 3H, H-5, H-6a, H-6′a), 3.71 (d, *J* = 9.7 Hz, 1H, H-6b), 3.59 (s, 4H, H-6′b, OC*H*_3_), 3.53 (t, *J* = 8.1 Hz, 2H, H-2, H-5″), 3.44 (s, 3H, OC*H*_3_), 3.43–3.38 (m, 1H, H-3″), 3.39 (s, 6H, 2 × OC*H*_3_), 3.36–3.34 (m, 1H, H-6″a), 3.22–3.18 (m, 2H, H-4″, H-6″b), 3.11 (dd, *J* = 3.3 Hz, *J* = 9.6 Hz, 1H, H-2″), 1.96 (s, 3H, Ac-C*H*_3_), 1.93 (s, 3H, Ac-C*H*_3_) ppm; ^13^C NMR (125 MHz, CDCl_3_) *δ* = 169.6 (2C, 2 × C_q_ Ac), 138.6, 138.3, 138.0, 136.0, 133.3, 133.0 (7C, C_q_ arom.), 128.5–125.8 (27C, arom.), 99.2 (1C, C-1′), 97.8 (1C, C-1), 97.1 (1C, C-1″), 83.4 (1C, C-3″), 81.5 (2C, C-2″, C-3), 80.2 (1C, C-2), 79.2 (1C, C-4″), 76.8 (1C, C-4), 75.2, 73.6, 73.3, 73.2 (5C, 4 × Bn-*C*H_2_, NAP-*C*H_2_), 72.1 (1C, C-3′), 72.0 (1C, C-5′), 71.0 (1C, C-5″), 70.2 (2C, C-2′, C-4′), 69.5 (1C, C-5), 69.2 (2C, C-6, C-6′), 68.1 (1C, C-6″), 60.7, 60.4, 59.2 (3C, 3 × O*C*H_3_), 55.3 (1C, C-1-O*C*H_3_), 21.0, 20.7 (2C, 2 × Ac-*C*H_3_) ppm; UHR ESI-QTOF (positive ion): *m*/*z* calcd. for [M + Na]^+^ C_65_H_76_NaO_18_ 1167.4924, found: 1167.4922.

Methyl (6-*O*-benzyl-2,3,4-tri-*O*-methyl-α-d-glucopyranosyl)-(1→4)-[2,3-di-*O*-acetyl-6-*O*-(2-naphthyl)methyl-β-d-mannopyranosyl]-(1→4)-2,3,6-tri-*O*-benzyl-α-d-glucopyranoside (**28**)

Disaccharide acceptor **25** (350 mg, 0.411 mmol) was glycosylated with **26** [65] (283 mg, 0.699 mmol, 1.7 equiv.) according to general method A. The crude product was purified by silica gel chromatography (7:3 *n*-hexane/acetone) to give **28** (420 mg, 89%) as a colourless syrup. *R*_f_ 0.49 (7:3 *n*-hexane/acetone); ${\left[\alpha \right]}_{D}^{25}$ + 78.0 (*c* 0.10, CHCl_3_); ^1^H NMR (700 MHz, CDCl_3_) *δ* = 7.81–7.13 (m, 27H, arom.), 5.43 (dd, *J* = 3.1 Hz, *J* = 9.0 Hz, 1H, H-3′), 5.34 (s, 1H, H-1′), 5.30–5.29 (m, 1H, H-2′), 5.28 (d, *J* = 3.7 Hz, 1H, H-1″), 5.03 (d, *J* = 11.0 Hz, 1H, Bn-C*H*_2_a), 4.75 (d, *J* = 10.9 Hz, 1H, Bn-C*H*_2_b), 4.70–4.44 (m, 7H, H-1, NAP-C*H*_2_, 2 × Bn-C*H*_2_), 4.32 (d, *J* = 12.1 Hz, 1H, Bn-C*H*_2_a), 4.21 (t, *J* = 9.3 Hz, 1H, H-4′), 4.10 (d, *J* = 12.1 Hz, 1H, Bn-C*H*_2_b), 3.97 (t, *J* = 9.1 Hz, 1H, H-3), 3.95–3.94 (m, 1H, H-5′), 3.83 (t, *J* = 9.2 Hz, 1H, H-4), 3.78–3.76 (m, 3H, H-5, H-6′ab), 3.71–3.70 (m, 1H, H-6a), 3.59 (s, 4H, H-6b, OC*H*_3_), 3.54–3.50 (m, 2H, H-2, H-5″), 3.43 (s, 3H, OC*H*_3_), 3.42–3.41 (m, 1H, H-3″), 3.39 (s, 6H, 2 × OC*H*_3_), 3.37–3.34 (m, 1H, H-6″a), 3.22–3.19 (m, 2H, H-4″, H-6″b), 3.11 (dd, *J* = 3.8 Hz, *J* = 9.8 Hz, 1H, H-2″), 1.96 (s, 3H, Ac-C*H*_3_), 1.93 (s, 3H, Ac-C*H*_3_) ppm; ^13^C NMR (176 MHz, CDCl_3_) *δ* = 169.6, 169.5 (2C, 2 × C_q_ Ac), 138.6, 138.3, 138.0, 137.9, 136.0, 133.3, 132.9 (7C, C_q_ arom.), 128.5–125.8 (27C, arom.), 99.2 (1C, C-1′), 97.8 (1C, C-1), 97.1 (1C, C-1″), 83.3 (1C, C-3″), 81.5 (2C, C-2″, C-3), 80.1 (1C, C-2), 79.2 (1C, C-4″), 76.8 (1C, C-4), 75.2, 73.6, 73.3, 73.2 (5C, 4 × Bn-*C*H_2_, NAP-*C*H_2_), 72.0 (1C, C-3′), 72.0 (1C, C-5′), 71.0 (1C, C-5″), 70.2 (2C, C-2′, C-4′), 69.5 (1C, C-5), 69.2 (2C, C-6, C-6′), 68.1 (1C, C-6″), 60.7, 60.4, 59.2 (3C, 3 × O*C*H_3_), 55.3 (1C, C-1-O*C*H_3_), 21.0, 20.7 (2C, 2 × Ac-*C*H_3_) ppm; UHR ESI-QTOF (positive ion): *m*/*z* calcd. for [M + Na]^+^ C_65_H_76_NaO_18_ 1167.4924, found: 1164.4922.

Methyl (2,3-di-*O*-benzyl-4,6-*O*-benzylidene-α-d-glucopyranosyl)-(1→4)-[2,3-di-*O*-acetyl-6-*O*-(2-naphthyl)methyl-α-d-mannopyranosyl]-(1→4)-2,3,6-tri-*O*-benzyl-α-d-glucopyranoside (**30**)

Reaction 1.: Compound **21** (420 mg, 0.493 mmol) was glycosylated with **29** [66] (453 mg, 0.839 mmol, 1.7 equiv.) according to general method A. The crude product was purified by silica gel chromatography (65:35 *n*-hexane/EtOAc) to give **30** (120 mg, 25%) as a colourless syrup.

Reaction 2.: Compound **21** (490 mg, 0.576 mmol) was glycosylated with **29** [66] (529 mg, 0.979 mmol, 1.7 equiv.) according to general method A, but the temperature was allowed to warm up to 0 °C and mixture was stirred under Argon for 6 h. The crude product was purified by silica gel chromatography (65:35 *n*-hexane/EtOAc) to give **30** (246 mg, 33%) as a colourless syrup.

Reaction 3.: Compound **21** (668 mg, 0.785 mmol) was glycosylated with **29** [66] (722 mg, 1.335 mmol, 1.7 equiv.) according to general method A, but the temperature was allowed to warm up to room temperature and the mixture was stirred under Argon for 48 h. The crude product was purified by silica gel chromatography (6:4 *n*-hexane/EtOAc) to give **30** (282 mg, 28%) as a colourless syrup. *R*_f_ 0.50 (6:4 *n*-hexane/EtOAc); ${\left[\alpha \right]}_{D}^{25}$ + 64.2 (*c* 0.24, CHCl_3_); ^1^H NMR (700 MHz, CDCl_3_) *δ* = 7.79–7.17 (m, 37H, arom.), 5.49 (s, 1H, H_ac_), 5.44 (dd, *J* = 3.1 Hz, *J* = 8.5 Hz, 1H, H-3′), 5.41 (d, *J* = 2.1 Hz, 1H, H-1′), 5.33 (t, *J* = 2.8 Hz, 1H, H-2′), 5.18 (d, *J* = 3.7 Hz, 1H, H-1″), 5.04–4.41 (m, 13H, H-1, NAP-C*H*_2_, 5 × Bn-C*H*_2_), 4.31 (t, *J* = 9.0 Hz, 1H, H-4′), 4.04 (t, *J* = 4.9 Hz, *J* = 10.2 Hz, 1H, H-6a″), 3.98 (t, *J* = 9.1 Hz, 1H, H-3), 3.93 (t, *J* = 9.4 Hz, 1H, H-3″), 3.90 (d, *J* = 9.6 Hz, 1H, H-5′), 3.86 (t, *J* = 9.4 Hz, 1H, H-4), 3.82–3.78 (m, 3H, H-5, H-5″, H-6′a), 3.71 (dd, *J* = 4.3 Hz, *J* = 10.6 Hz, 1H, H-6a), 3.67 (d, *J* = 10.8 Hz, 1H, H-6b), 3.54–3.50 (m, 3H, H-2, H-4″, H-6′b), 3.48–3.45 (m, 2H, H-2″, H-6″b), 3.39 (s, 3H, C-1-OC*H*_3_), 1.95 (s, 3H, Ac-C*H*_3_), 1.87 (s, 3H, Ac-C*H*_3_) ppm; ^13^C NMR (176 MHz, CDCl_3_) *δ* = 169.9, 169.7 (2C, 2 × C_q_ Ac), 138.8, 138.7, 138.3, 138.1, 138.0, 137.6, 135.8, 133.4, 133.0 (9C, C_q_ arom.), 129.0–125.9 (37C, arom.), 101.3 (1C, C_ac_), 99.0 (1C, C-1′), 97.9 (1C, C-1), 97.7 (1C, C-1″), 82.2 (1C, C-4″), 81.7 (1C, C-3), 80.3 (1C, C-2), 79.4 (1C, C-2″), 78.5 (1C, C-3″), 75.9 (1C, C-4), 75.4, 75.3, 73.7, 73.4, 73.3 (6C, 5 × Bn-*C*H_2_, NAP-*C*H_2_), 71.9 (1C, C-5′), 71.7 (1C, C-3′), 70.6 (1C, C-4′), 70.3 (1C, C-2′), 69.5 (1C, C-5), 69.2 (1C, C-6), 69.0 (1C, C-6″), 68.7 (1C, C-6′), 63.5 (1C, C-5″), 55.4 (1C, C-1-O*C*H_3_), 21.1, 20.9 (2C, 2 × Ac-*C*H_3_) ppm; UHR ESI-QTOF (positive ion): *m*/*z* calcd. for [M + Na]^+^ C_76_H_80_NaO_18_ 1303.5237, found: 1303.5271.

Methyl (4-*O*-acetyl-2,3,6-tri-*O*-benzyl-α-d-glucopyranosyl)-(1→4)-[2,3-di-*O*-acetyl-6-*O*-(2-naphthyl)methyl-α-d-mannopyranosyl]-(1→4)-2,3,6-tri-*O*-benzyl-α-d-glucopyranoside (**32**)

Disaccharide acceptor **21** (2.000 g, 2.350 mmol) was glycosylated with **31** [73] (2.061 g, 3.525 mmol, 1.5 equiv.) according to general method A. The crude product was purified by silica gel chromatography (6:4 *n*-hexane/acetone) to give **32** (2.70 g, 86%) as a colourless syrup. *R*_f_ 0.48 (6:4 *n*-hexane/acetone); ${\left[\alpha \right]}_{D}^{25}$ + 62.9 (*c* 0.27, CHCl_3_); ^1^H NMR (500 MHz, CDCl_3_) *δ* = 7.80–7.09 (m, 37H, arom.), 5.49 (dd, *J* = 3.2 Hz, *J* = 9.0 Hz, 1H, H-3′), 5.40 (d, *J* = 2.0 Hz, 1H, H-1′), 5.34–5.33 (m, 1H, H-2′), 5.18 (d, *J* = 3.5 Hz, 1H, H-1″), 5.05 (d, *J* = 11.1 Hz, 1H, Bn-C*H*_2_a), 4.99 (t, *J* = 9.8 Hz, 1H, H-4″), 4.80–4.41 (m, 12H, H-1, NAP-C*H*_2_, 4 × Bn-C*H*_2_, Bn-C*H*_2_b), 4.31–4.27 (m, 2H, H-4′, Bn-C*H*_2_a), 4.11 (d, *J* = 12.0 Hz, 1H, Bn-C*H*_2_b), 3.99 (t, *J* = 9.1 Hz, 1H, H-3), 3.96–3.90 (m, 2H, H-5′, H-6′a), 3.88–3.84 (m, 1H, H-4), 3.81–3.78 (m, 3H, H-3″, H-5, H-5″), 3.73–3.69 (m, 1H, H-6a), 3.71–3.69 (m, 1H, H-6b), 3.63–3.61 (m, 1H, H-6′b), 3.53 (dd, *J* = 3.4 Hz, *J* = 9.6 Hz, 1H, H-2), 3.49 (dd, *J* = 3.5 Hz, *J* = 9.8 Hz, 1H, H-2″), 3.39 (s, 3H, C-1-OC*H*_3_), 3.18 (dd, *J* = 2.4 Hz, *J* = 10.6 Hz, 1H, H-6″b), 3.14 (dd, *J* = 4.4 Hz, *J* = 10.7 Hz, 1H, H-6″b), 1.98, 1.85, 1.73 (3 × s, 9H, 3 × Ac-C*H*_3_) ppm; ^13^C NMR (125 MHz, CDCl_3_) *δ* = 170.0, 169.7 (3C, 3 × C_q_ Ac), 138.6, 138.3, 138.0, 137.8, 135.9, 133.3, 133.0 (9C, C_q_ arom.), 128.5–125.9 (37C, arom.), 99.1 (1C, C-1′), 97.9 (1C, C-1), 97.4 (1C, C-1″), 81.6 (1C, C-3), 80.2 (1C, C-2), 80.0 (1C, C-2″), 78.9 (1C, C-3″), 76.2 (1C, C-4), 75.3, 73.6, 73.5, 73.4, 73.3 (7C, 6 × Bn-*C*H_2_, NAP-*C*H_2_), 72.4 (1C, C-5′), 71.6 (1C, C-4′), 71.3 (1C, C-3′), 70.4 (1C, C-2′), 70.3 (1C, C-4″), 69.6 (2C, C-5, C-5″), 69.2 (1C, C-6), 69.0 (1C, C-6′), 68.6 (1C, C-6″), 55.4 (1C, C-1-O*C*H_3_), 21.1, 20.8 (3C, 3 × Ac-*C*H_3_) ppm; UHR ESI-QTOF (positive ion): *m*/*z* calcd. for [M + 2Na]^2+^ C_78_H_84_Na_2_O_19_ 685.2696, found: 685.2695.

Methyl (6-*O*-benzyl-2,3,4-tri-*O*-methyl-α-d-glucopyranosyl)-(1→4)-(2,3-di-*O*-acetyl-β-d-mannopyranosyl)-(1→4)-2,3,6-tri-*O*-benzyl-α-d-glucopyranoside (**34**)

Compound **28** (410 mg, 0.358 mmol) was converted to **34** by general method D. The crude product was purified by silica gel chromatography (6:4 *n*-hexane/acetone) to produce compound **34** (260 mg, 72%) as a white foam. *R*_f_ 0.50 (6:4 *n*-hexane/acetone); ${\left[\alpha \right]}_{D}^{25}$ + 79.0 (*c* 0.10, CHCl_3_); ^1^H NMR (700 MHz, CDCl_3_) *δ* = 7.33–7.22 (m, 20H, arom.), 5.38 (dd, *J* = 3.2 Hz, *J* = 9.1 Hz, 1H, H-3′), 5.34 (d, *J* = 1.8 Hz, 1H, H-1′), 5.26 (t, *J* = 2.4 Hz, 1H, H-2′), 5.23 (d, *J* = 4.0 Hz, 1H, H-1″), 5.03 (d, *J* = 11.1 Hz, 1H, Bn-C*H*_2_a), 4.71–4.69 (m, 2H, Bn-C*H*_2_a, Bn-C*H*_2_b), 4.61–4.51 (m, 6H, H-1, Bn-C*H*_2_b, 2 × Bn-C*H*_2_), 4.12 (t, *J* = 9.3 Hz, 1H, H-4′), 3.96 (t, *J* = 9.2 Hz, 1H, H-3), 3.82 (t, *J* = 8.9 Hz, 1H, H-4), 3.74–3.72 (m, 2H, H-5, H-5′), 3.71–3.68 (m, 1H, H-6′a), 3.66–3.62 (m, 4H, H-5″, H-6a,b, H-6″a), 3.60–3.58 (m, 5H, OC*H*_3_, H-6′b, H-6″b), 3.51 (dd, *J* = 3.4 Hz, *J* = 9.6 Hz, 1H, H-2), 3.45 (2 × s, 6H, 2 × OC*H*_3_), 3.44 (t, *J* = 9.3 Hz, 1H, H-3″), 3.39 (s, 3H, C-1-OC*H*_3_), 3.13 (dd, *J* = 4.0 Hz, *J* = 9.8 Hz, 1H, H-2″), 3.08 (t, *J* = 9.5 Hz, 1H, H-4″), 2.65–2.64 (m, 1H, H-6′-OH), 1.96 (s, 3H, Ac-C*H*_3_), 1.89 (s, 3H, Ac-C*H*_3_) ppm; ^13^C NMR (176 MHz, CDCl_3_) *δ* = 169.6, 169.5 (2C, 2 × C_q_ Ac), 138.5, 138.1, 138.0, 137.5 (4C, C_q_ arom.), 128.5–127.2 (20C, arom.), 99.1 (1C, C-1′), 97.7 (1C, C-1), 97.6 (1C, C-1″), 83.6 (1C, C-3″), 81.6 (1C, C-3), 81.2 (1C, C-2″), 80.1 (1C, C-2), 79.9 (1C, C-4″), 76.0 (1C, C-4), 75.1, 73.6, 73.5, 73.2 (4C, 4 × Bn-*C*H_2_), 72.2 (1C, C-5′), 72.0 (1C, C-3′), 71.3 (1C, C-5″), 70.1 (2C, C-2′, C-4′), 69.5 (1C, C-5), 68.8, 69.7 (2C, C-6, C-6″), 61.1 (1C, C-6′), 60.7, 60.5, 59.5 (3C, 3 × O*C*H_3_), 55.3 (1C, C-1-O*C*H_3_), 20.9, 20.7 (2C, 2 × Ac-*C*H_3_) ppm; UHR ESI-QTOF (positive ion): *m*/*z* calcd. for [M + 2Na]^2+^ C_54_H_68_NaO_18_ 525.2095, found: 525.2095.

Methyl (6-*O*-benzyl-2,3,4-tri-*O*-methyl-α-d-glucopyranosyl)-(1→4)-(sodium 2,3-di-*O*-acetyl-β-d-mannopyranosyl-uronate)-(1→4)-2,3,6-tri-*O*-benzyl-α-d-glucopyranoside (**35**)

Compound **34** (260 mg, 0.259 mmol) was converted to **35** according to general method E. The crude product was purified by silica gel chromatography (95:5 CH_2_Cl_2_/MeOH) to produce compound **35** (209 mg, 78%) as a white foam. *R*_f_ 0.38 (95:5 CH_2_Cl_2_/MeOH); ${\left[\alpha \right]}_{D}^{25}$ + 1.43 (*c* 0.15, CHCl_3_); ^1^H NMR (700 MHz, CD_3_OD) *δ* = 7.33–7.22 (m, 20H, arom.), 5.46 (d, *J* = 3.4 Hz, 1H, H-1′), 5.36 (dd, *J* = 3.2 Hz, *J* = 7.1 Hz, 1H, H-3′), 5.23 (t, *J* = 3.4 Hz, 1H, H-2′), 5.19 (d, *J* = 3.8 Hz, 1H, H-1″), 5.01 (d, *J* = 10.9 Hz, 1H, Bn-C*H*_2_a), 4.73–4.47 (m, 8H, H-1, Bn-C*H*_2_a, 3 × Bn-C*H*_2_), 4.34–4.30 (m, 2H, H-4′, H-5′), 3.95 (t, *J* = 9.2 Hz, 1H, H-3), 3.90 (t, *J* = 9.2 Hz, 1H, H-4), 3.85 (dd, *J* = 4.1 Hz, *J* = 12.0 Hz, 1H, H-6″a), 3.75–3.72 (m, 3H, H-5, H-5″, H-6″b), 3.68 (dd, *J* = 1.7 Hz, *J* = 10.7 Hz, 1H, H-6a), 3.63 (dd, *J* = 4.0 Hz, *J* = 10.5 Hz, 1H, H-6b), 3.58 (s, 3H, OC*H*_3_), 3.50 (dd, *J* = 3.5 Hz, *J* = 9.5 Hz, 1H, H-2), 3.46–3.44 (m, 4H, H-3″, OC*H*_3_), 3.43 (s, 3H, OC*H*_3_), 3.38 (s, 3H, C-1-OC*H*_3_), 3.17 (d, *J* = 9.5 Hz, 1H, H-4″), 3.14 (dd, *J* = 3.9 Hz, *J* = 9.8 Hz, 1H, H-2″), 1.93 (s, 3H, Ac-C*H*_3_), 1.89 (s, 3H, Ac-C*H*_3_) ppm; ^13^C NMR (176 MHz, CD_3_OD) *δ* = 169.6, 169.5 (3C, *C*OONa, 2 × C_q_ Ac), 138.7, 138.1, 137.9, 137.8 (4C, C_q_ arom.), 128.5–127.4 (20C, arom.), 99.1 (1C, C-1′), 97.9 (1C, C-1), 97.4 (1C, C-1″), 83.2 (1C, C-3″), 81.4 (2C, C-2″, C-3), 80.2 (1C, C-2), 79.6 (1C, C-4″), 76.3 (1C, C-4), 75.2, 73.9, 73.7, 73.3 (4C, 4 × Bn-*C*H_2_), 72.6 (2C, C-4′, C-5′), 71.0 (1C, C-5″), 70.5 (1C, C-3′), 69.6 (1C, C-5), 69.5 (1C, C-2′), 68.9 (1C, C-6″), 68.7 (1C, C-6), 60.8, 60.5, 59.3 (3C, 3 × O*C*H_3_), 55.4 (1C, C-1-O*C*H_3_), 20.9, 20.8 (2C, 2 × Ac-*C*H_3_) ppm; UHR ESI-QTOF (positive ion): *m*/*z* calcd. for [M + H]^+^ C_54_H_65_NaO_19_ 1041.4091, found: 1041.4090.

Methyl (6-*O*-benzyl-2,3,4-tri-*O*-methyl-α-d-glucopyranosyl)-(1→4)-[2,3-di-*O*-acetyl-α-d-mannopyranosyl]-(1→4)-2,3,6-tri-*O*-benzyl-α-d-glucopyranoside (**36**)

Compound **27** (1.20 g, 1.047 mmol) was converted to **36** by general method D. The crude product was purified by silica gel chromatography (6:4 *n*-hexane/acetone) to produce compound **36** (723 mg, 69%) as a white foam. *R*_f_ 0.41 (6:4 *n*-hexane/acetone); ${\left[\alpha \right]}_{D}^{25}$ + 81.9 (*c* 0.26, CHCl_3_); ^1^H NMR (500 MHz, CDCl_3_) *δ* = 7.33–7.21 (m, 20H, arom.), 5.38 (dd, *J* = 3.3 Hz, *J* = 9.1 Hz, 1H, H-3′), 5.33 (d, *J* = 1.9 Hz, 1H, H-1′), 5.25 (t, *J* = 2.6 Hz, 1H, H-2′), 5.23 (d, *J* = 4.0 Hz, 1H, H-1″), 5.03 (d, *J* = 11.1 Hz, 1H, Bn-C*H*_2_a), 4.72–4.50 (m, 8H, H-1, Bn-C*H*_2_b, 3 × Bn-C*H*_2_), 4.12 (t, *J* = 9.3 Hz, 1H, H-4′), 3.96 (t, *J* = 9.1 Hz, 1H, H-3), 3.82 (t, *J* = 8.9 Hz, 1H, H-4), 3.75–3.72 (m, 2H, H-5, H-5′), 3.67–3.64 (m, 4H, H-5″, H-6a, H-6′a, H-6″a), 3.60 (s, 3H, OC*H*_3_), 3.59–3.57 (m, 3H, H-6b, H-6′b, H-6″b), 3.51 (dd, *J* = 3.5 Hz, *J* = 9.6 Hz, 1H, H-2), 3.45 (2 × s, 6H, 2 × OC*H*_3_), 3.43 (t, *J* = 9.4 Hz, 1H, H-3″), 3.39 (s, 3H, C-1-OC*H*_3_), 3.13 (dd, *J* = 4.0 Hz, *J* = 9.8 Hz, 1H, H-2″), 3.08 (t, *J* = 9.4 Hz, 1H, H-4″), 2.63 (t, *J* = 6.3 Hz, 1H, H-6′-OH), 1.96 (s, 3H, Ac-C*H*_3_), 1.89 (s, 3H, Ac-C*H*_3_) ppm; ^13^C NMR (125 MHz, CDCl_3_) *δ* = 169.6, 169.5 (2C, 2 × C_q_ Ac), 138.6, 138.1, 138.0, 137.6 (4C, C_q_ arom.), 128.5–127.2 (20C, arom.), 99.1 (1C, C-1′), 97.8 (1C, C-1), 97.6 (1C, C-1″), 83.6 (1C, C-3″), 81.6 (1C, C-3), 81.3 (1C, C-2″), 80.1 (1C, C-2), 79.9 (1C, C-4″), 76.0 (1C, C-4), 75.1, 73.6, 73.5, 73.2 (4C, 4 × Bn-*C*H_2_), 72.2 (1C, C-5′), 72.0 (1C, C-3′), 71.4 (1C, C-5″), 70.1 (2C, C-2′, C-4′), 69.6 (1C, C-5), 68.8 (2C, C-6, C-6″), 61.2 (1C, C-6′), 60.7, 60.5, 59.6 (3C, 3 × O*C*H_3_), 55.3 (1C, C-1-O*C*H_3_), 20.9, 20.7 (2C, 2 × Ac-*C*H_3_) ppm; UHR ESI-QTOF (positive ion): *m*/*z* calcd. for [M + 2Na]^2+^ C_54_H_68_Na_2_O_18_ 525.2095, found: 525.2095.

Methyl (6-*O*-benzyl-2,3,4-tri-*O*-methyl-α-d-glucopyranosyl)-(1→4)-(sodium 2,3-di-*O*-acetyl-α-d-mannopyranosyl-uronate)-(1→4)-2,3,6-tri-*O*-benzyl-α-d-glucopyranoside (**37**)

Compound **36** (653 mg, 0.649 mmol) was converted to **37** according to general method E. The crude product was purified by silica gel chromatography (95:5 CH_2_Cl_2_/MeOH) to produce compound **37** (527 mg, 78%) as a white foam. *R*_f_ 0.83 (1:1 CH_2_Cl_2_/MeOH); ${\left[\alpha \right]}_{D}^{25}$ + 61.4 (*c* 0.28, CHCl_3_); ^1^H NMR (700 MHz, CDCl_3_) *δ* = 7.33–7.22 (m, 20H, arom.), 5.45 (d, *J* = 3.0 Hz, 1H, H-1′), 5.36 (dd, *J* = 3.1 Hz, *J* = 7.4 Hz, 1H, H-3′), 5.24 (t, *J* = 3.3 Hz, 1H, H-2′), 5.19 (d, *J* = 3.7 Hz, 1H, H-1″), 5.01 (d, *J* = 11.0 Hz, 1H, Bn-C*H*_2_a), 4.72–4.46 (m, 8H, H-1, Bn-C*H*_2_a, 3 × Bn-C*H*_2_), 4.33 (t, *J* = 8.1 Hz, 1H, H-4′), 4.31 (t, *J* = 7.8 Hz, 1H, H-5′), 3.95 (t, *J* = 9.2 Hz, 1H, H-3), 3.90 (t, *J* = 9.4 Hz, 1H, H-4), 3.84 (dd, *J* = 2.8 Hz, *J* = 11.1 Hz, 1H, H-6a), 3.74–3.70 (m, 3H, H-5, H-5″, H-6b), 3.68–3.67 (m, 1H, H-6″a), 3.63 (dd, *J* = 4.0 Hz, *J* = 10.6 Hz, 1H, H-6″b), 3.58 (s, 3H, OC*H*_3_), 3.50 (dd, *J* = 3.4 Hz, *J* = 9.5 Hz, 1H, H-2), 3.46–3.44 (m, 4H, H-3″, OC*H*_3_), 3.42 (s, 3H, OC*H*_3_), 3.38 (s, 3H, C-1-OC*H*_3_), 3.17 (d, *J* = 10.2 Hz, 1H, H-4″), 3.13 (dd, *J* = 3.8 Hz, *J* = 9.8 Hz, 1H, H-2″), 1.93 (s, 3H, Ac-C*H*_3_), 1.88 (s, 3H, Ac-C*H*_3_) ppm; ^13^C NMR (176 MHz, CDCl_3_) *δ* = 169.6 (3C, *C*OONa, 2 × C_q_ Ac), 138.6, 138.0, 137.8 (4C, C_q_ arom.), 128.6–127.4 (20C, arom.), 99.2 (1C, C-1′), 97.9 (1C, C-1), 97.4 (1C, C-1″), 83.2 (1C, C-3″), 81.5 (2C, C-2″, C-3), 80.2 (1C, C-2), 79.6 (1C, C-4″), 76.3 (1C, C-4), 75.2, 73.9, 73.7, 73.3 (4C, 4 × Bn-*C*H_2_), 72.6 (2C, C-4′, C-5′), 71.0 (1C, C-5″), 70.6 (1C, C-3′), 69.6 (1C, C-5), 69.5 (1C, C-2′), 68.9 (1C, C-6″), 68.7 (1C, C-6), 60.8, 60.5, 59.3 (3C, 3 × O*C*H_3_), 55.4 (1C, C-1-O*C*H_3_), 20.9, 20.7 (2C, 2 × Ac-*C*H_3_) ppm; UHR ESI-QTOF (positive ion): *m*/*z* calcd. for [M + H]^+^ C_54_H_65_NaO_19_ 1041.4091, found: 1041.4095.

Methyl (4-*O*-acetyl-2,3,6-tri-*O*-benzyl-α-d-glucopyranosyl)-(1→4)-(2,3-di-*O*-acetyl-α-d-mannopyranosyl)-(1→4)-2,3,6-tri-*O*-benzyl-α-d-glucopyranoside (**38**)

Compound **32** (2.02 g, 1.527 mmol) was converted to **38** by general method D. The crude product was purified by silica gel chromatography (6:4 *n*-hexane/acetone) to produce compound **38** (985 mg, 54%) as a white foam. *R*_f_ 0.64 (1:1 *n*-hexane/acetone); ${\left[\alpha \right]}_{D}^{25}$ + 68.8 (*c* 0.49, CHCl_3_); ^1^H NMR (700 MHz, CDCl_3_) *δ* = 7.32–7.22 (m, 30H, arom.), 5.42 (dd, *J* = 3.2 Hz, *J* = 8.5 Hz, 1H, H-3′), 5.37 (d, *J* = 2.5 Hz, 1H, H-1′), 5.31 (t, *J* = 3.0 Hz, 1H, H-2′), 5.32 (d, *J* = 3.0 Hz, 1H, H-1″), 5.04 (d, *J* = 11.0 Hz, 1H, Bn-C*H*_2_a), 4.97 (t, *J* = 10.1 Hz, 1H, H-4″), 4.80–4.64 (m, 5H, Bn-C*H*_2_a, 2 × Bn-C*H*_2_b, Bn-C*H*_2_), 4.61 (d, *J* = 3.6 Hz, 1H, H-1), 4.60–4.46 (m, 6H, 3 × Bn-C*H*_2_), 4.15 (t, *J* = 9.0 Hz, 1H, H-4′), 3.98 (t, *J* = 9.1 Hz, 1H, H-3), 3.85 (t, *J* = 9.5 Hz, 1H, H-4), 3.84–3.80 (m, 3H, H-3″, H-5′, H-5″), 3.79–3.75 (m, 3H, H-5, H-6a, H-6′a), 3.68–3.66 (m, 2H, H-6b, H-6′b), 3.57 (dd, *J* = 3.8 Hz, *J* = 9.7 Hz, 1H, H-2″), 3.53 (dd, *J* = 3.4 Hz, *J* = 9.7 Hz, 1H, H-2), 3.44 (dd, *J* = 2.4 Hz, *J* = 10.5 Hz, 1H, H-6″a), 3.42–3.41 (m, 1H, H-6″b), 3.40 (s, 3H, C-1-OC*H*_3_), 2.63 (t, *J* = 6.6 Hz, 1H, H-6′-OH), 1.93, 1.85, 1.82 (3 × s, 9H, 3 × Ac-C*H*_3_) ppm; ^13^C NMR (176 MHz, CDCl_3_) *δ* = 169.9, 169.7, 169.6 (3C, 3 × C_q_ Ac), 138.6, 138.5, 138.3, 138.1, 137.7, 137.5 (6C, C_q_ arom.), 128.6–127.4 (30C, arom.), 99.1 (1C, C-1′), 97.9 (1C, C-1), 97.3 (1C, C-1″), 81.7 (1C, C-3), 80.3 (1C, C-2), 79.6 (1C, C-2″), 79.0 (1C, C-3″), 75.8 (1C, C-4), 75.4, 75.2, 73.7, 73.6, 73.3 (6C, 6 × Bn-*C*H_2_), 72.5 (1C, C-5′), 71.9 (1C, C-3′), 70.5 (1C, C-4′), 70.3 (1C, C-4″), 70.2 (2C, C-2′, C-5″), 69.8 (1C, C-5), 68.9 (1C, C-6), 68.7 (1C, C-6″), 61.6 (1C, C-6′), 55.5 (1C, C-1-O*C*H_3_), 21.1, 20.9, 20.8 (3C, 3 × Ac-*C*H_3_) ppm; UHR ESI-QTOF (positive ion): *m*/*z* calcd. for [M + 2Na]^2+^ C_67_H_76_Na_2_O_19_ 615.2383, found: 615.2385.

Methyl (4-*O*-aceyl-2,3,6-tri-*O*-benzyl-α-d-glucopyranosyl)-(1→4)-(sodium 2,3-di-*O*-acetyl-α-d-mannopyranosyl-uronate)-(1→4)-2,3,6-tri-*O*-benzyl-α-d-glucopyranoside (**39**)

Compound **38** (940 mg, 0.793 mmol) was converted to **39** according to general method E, using CH_3_CN instead of CH_2_Cl_2_ as a solvent. The crude product was purified by silica gel chromatography (95:5 CH_2_Cl_2_/MeOH) to produce compound **39** (735 mg, 76%) as a white foam. *R*_f_ 0.36 (4:6 *n*-hexane/acetone); ${\left[\alpha \right]}_{D}^{25}$ + 13.6 (*c* 0.14, CHCl_3_); ^1^H NMR (700 MHz, CD_3_OD) *δ* = 7.37–7.19 (m, 30H, arom.), 5.73 (d, *J* = 6.0 Hz, 1H, H-1′), 5.38 (dd, *J* = 3.2 Hz, *J* = 5.6 Hz, 1H, H-3′), 5.23 (dd, *J* = 3.1 Hz, *J* = 6.3 Hz, 1H, H-2′), 5.14 (d, *J* = 3.5 Hz, 1H, H-1″), 4.98 (t, *J* = 9.8 Hz, 1H, H-4″), 4.91–4.74 (m, 6H, 3 × Bn-C*H*_2_), 4.71 (d, *J* = 3.4 Hz, 1H, H-1), 4.65 (d, *J* = 4.0 Hz, 1H, H-5′), 4.61–4.37 (m, 6H, 3 × Bn-C*H*_2_), 4.34 (t, *J* = 4.6 Hz, 1H, H-4′), 3.97 (ddd, *J* = 2.7 Hz, *J* = 4.6 Hz, *J* = 10.1 Hz, 1H, H-5″), 3.94 (t, *J* = 9.2 Hz, 1H, H-4), 3.91–3.86 (m, 3H, H-3, H-3″, H-6a), 3.74 (ddd, *J* = 1.6 Hz, *J* = 5.5 Hz, *J* = 9.8 Hz, 1H, H-5), 3.69 (dd, *J* = 5.5 Hz, *J* = 10.9 Hz, 1H, H-6b), 3.56 (dd, *J* = 3.6 Hz, *J* = 9.8 Hz, 1H, H-2″), 3.54 (dd, *J* = 3.5 Hz, *J* = 9.6 Hz, 1H, H-2), 3.46 (dd, *J* = 2.5 Hz, *J* = 10.8 Hz, 1H, H-6″a), 3.41 (dd, *J* = 4.8 Hz, *J* = 10.8 Hz, 1H, H-6″b), 3.36 (s, 3H, C-1-OC*H*_3_), 1.96, 1.79, 1.73 (3 × s, 9H, 3 × Ac-C*H*_3_) ppm; ^13^C NMR (176 MHz, CD_3_OD) *δ* = 171.5, 171.3, 171.2 (4C, *C*OONa, 3 × C_q_ Ac), 140.2, 139.8, 139.7, 139.6, 139.5, 139.3 (6C, C_q_ arom.), 129.5–128.2 (30C, arom.), 99.4 (1C, C-1″), 98.8 (1C, C-1), 97.9 (1C, C-1′), 82.6 (1C, C-3), 81.5 (1C, C-2), 81.0 (1C, C-2″), 79.7 (1C, C-3″), 76.6 (1C, C-4), 76.1, 75.8 (2C, 2 × Bn-*C*H_2_), 75.7 (1C, C-4′), 75.1 (1C, C-5′), 74.6, 74.4, 74.1, 73.9 (4C, 4 × Bn-*C*H_2_), 71.5 (1C, C-4″), 71.4 (1C, C-3′), 71.0 (2C, C-5, C-5″), 70.9 (1C, C-6), 70.2 (1C, C-2′), 70.0 (1C, C-6″), 55.6 (1C, C-1-O*C*H_3_), 21.0, 20.8 (3C, 3 × Ac-*C*H_3_) ppm; UHR ESI-QTOF (positive ion): *m*/*z* calcd. for [M + H]^+^ C_67_H_74_NaO_20_ 1221.4666, found: 1221.4634.

## 4. Conclusions

Continuing our previous work, we successfully synthesized non-glycosaminoglycan-type heparan sulphate/heparosan analogue trisaccharides containing α- or β-mannosidic-linked d-mannuronic acid units instead of a d-glucuronic acid building block. Of the eleven derivatives prepared, four contain β-mannosidic linked mannuronic acid units and seven contain α-mannosidic linked mannuronic acid units. The total synthesis of these non-glycosaminoglycan-type derivatives required 20–24 steps.

In order to increase the low yield of one of the glycosylation steps, in silico conformational analysis methods were used to designed a new donor, because of the analysis of key donors and carbocations revealed conformational and energetic reasons behind the different reactivity of **26**, **29** and **31**. Based on these results, the synthesis of the trisaccharide in question was optimized using a new monosaccharide donor (**31**).

In order to obtain a primary insight to their putative biological effects, we decided to perform different screening assays using a single concentration of the compounds. At a concentration of 10 µM, the synthesized trisaccharides showed no effect on either the viability of primary human dermal FBs or their collagen production. However, further studies may be required for a more thorough investigation of the effect of these compounds on collagen production (i.e., examination of dose-dependency and the expression of different collagen subtypes) *in vivo*.

Of great importance, we could also demonstrate that some of the trisaccharides were able to significantly suppress TLR3 activation-induced IL-6 release, demonstrating promising anti-inflammatory properties. Interestingly, this effect exhibited a marked donor-dependence. Indeed, while in case of Donor 1, almost all test agents (except for **5** and **12**) were able to significantly suppress IL-6 release, in case of Donor 2, only **4**, **5**, **6**, **7**, **8**, and **9** could decrease the release of this cytokine. Of course, it would be premature to draw bold conclusions, but these data argue that some of the trisaccharides (**4**, **5**, **6**, **7**, **8**, and **9**) might exert donor-independent anti-inflammatory activity, without the risk of impairing wound healing or triggering pathological fibrosis. Further studies (assessing multiple concentrations of each molecule) are therefore invited to explore putative additional beneficial effects of these compounds (among others on the release of key itch mediators, e.g., IL-31).

Finally, from the perspective of rational drug design, it is important to find connections between the structure and the biological activity of these compounds. Here, it is noteworthy that some of the compounds lacking sulphate ester group, but having methyl groups (i.e., **4**, **8**, **9**) were more effective, in case of Donor 2, than the more polaric -OH and -OAc group-containing derivatives (**13**, **14**, **15**). Indeed, these latter compounds only decreased IL-6 release in case of Donor 1, but not in case of Donor 2, indicating that certain structures (e.g., trisaccharides with methyl groups, but without sulphate ester group) may exert more universal, possibly donor-independent, anti-inflammatory effects.

Obviously, the molecular mechanism of the beneficial IL-6 suppressing effect is unclear at this point. However, the fact that some compounds were efficient in both donors, while others only in one of them, argues that the different trisaccharides might have targeted independent anti-inflammatory pathways in the FBs. If proven true, this hypothesis may pave the way of the application of rational combinations of these compounds to further increase their efficiency by taking advantage of targeting independent anti-inflammatory pathways simultaneously. Thus, systematic exploration of the details of these anti-inflammatory effects is warranted.

## Figures and Tables

**Table 1:** Optimization of the glycosylation reaction of the monosaccharide donor **29** and disaccharide acceptor **21**.

	Promoter	Temperature (°C)	Reaction Time (h)	Yield ^1^ (%)
Reaction I.	NIS/TfOH	−40 to −20	3	25
Reaction II.	NIS/TfOH	−40 to 0	6	33
Reaction III.	NIS/TfOH	−40 to rt.	48	28

^1^ Isolated yield.

## Data Availability

Data are contained within the article and Supplementary Materials.

## Supplementary Materials

The following supporting information can be downloaded at https://www.mdpi.com/article/10.3390/ijms26178305/s1.
