# Supplementary material for: The First Simplified Heparan Sulphate—Alginate Hybrid Trisaccharides: Synthesis and Biological Effects on Human Dermal Fibroblasts
Source: Int J Mol Sci. 2025 Aug 27;26(17):8305. doi: 10.3390/ijms26178305 (PMC12428572; doi:10.3390/ijms26178305)
Supplement: Supplementary file 1 [file ijms-26-08305-s001.zip › ijms-3792876-supplementary.pdf]

Supporting Information

The first simplified heparan sulphate—alginate hybrid trisaccharides: Synthesis and biological effects on human dermal fibroblasts

Katalin Kútvölgyi [1,2,3], Zsófia Peleskei [1,2], Fruzsina Demeter [1], Roland A. Barta [4], Attila Mándi [4], Eszter Homoki [5,6], Attila Oláh [5], János Hajkó [3], Mihály Herczeg [1,*] and Erika Lisztes [5,*]

[1] Department of Pharmaceutical Chemistry, Faculty of Pharmacy, University of Debrecen, H-4032, Debrecen, Egyetem tér 1., Hungary; [2] Doctoral School of Pharmacy, Medical Science Doctoral Council, University of Debrecen, H-4032, Debrecen, Egyetem tér 1., Hungary; [3] TAPI Hungary Industries Kft., H-4042, Debrecen, Pallagi út 13., [4] Department of Organic Chemistry, University of Debrecen, P.O. Box 400, H-4002, Debrecen, Hungary; [5] Department of Physiology, Faculty of Medicine, University of Debrecen, P.O. Box: 22, H-4012, Debrecen, Hungary; [6] Doctoral School of Molecular Medicine, University of Debrecen, Egyetem tér 1, H-4032, Debrecen, Hungary

Abstract Glycosaminoglycans (GAGs) are linear, high molecular weight polydisperse heteropolysaccharides consisting of repeating disaccharide units which always contain a uronic acid building block (e.g. D-glucuronic acid or L-iduronic acid). Their analogues containing D-mannuronic acid were not known until now. Another important class of the linear negatively charged polisaccharides are alginates which are also present in the cell surface of in the cell wall. They are composed of blocks of 1,4-linked β-D-mannuronic acid and its C-5 epimer α-L-guluronic acid in alternating or random order. Both groups of molecules have significant biological activity (e.g.: cell growth inhibitory activity, anti-inflammatory effect etc.). In the course of our research, we combined the structural characteristics of these two groups of molecules and produced a series of heparan sulphate analogue trisaccharides containing D-mannuronic acid, with a simplified structure, in which α- and β-mannosidic bonds are also found. Since trisaccharides may exert diverse biological effects and alginate derivatives can influence wound healing processes, we investigated the effects of the synthesized compounds on primary human dermal fibroblasts. We found that, when applied at 10 μM, none of the compounds influenced viability or spontaneous collagen production; however, some derivatives exhibited anti-inflammatory activity, and suppressed the poly(I:C)-induced release of interleukin 6.

Table of Contents

In silico studies 3
References.. 19
NMR spectra of the synthesized compounds... 20

In silico studies

Computational Section

Mixed torsional/low-mode conformational searches were carried out by means of the MacroModel 10.8.011 software [1] using the OPLS2005 force field [2] with an implicit solvent model for $CHCl_3$ applying a 21 kJ/mol energy window. Geometry re-optimizations of the resultant conformers (ωB97X/6-31G(d) and than ωB97X/TZVP levels both with PCM solvent model for CH_2Cl_2) were performed with the Gaussian 16 package [3]. Boltzmann distributions were estimated from the ωB97X energies. Visualization of the results was performed by the MOLEKEL software package [4]. Puckering values were generated based on the model proposed by Cremer and Pople using the Cremer–Pople Parameter Calculator [5,6].

Table S1. Cartesian coordinates, energies and Cremer-Pople parameters of the low-energy conformers calculated at the ωB97X/TZVP PCM/CH_2Cl_2 level.

33, Conf A
Charge: 0

C	1.999646	-1.629989	0.450693
C	2.810805	-0.830599	-0.552123
C	2.994923	0.615644	-0.111142
C	1.642967	1.244152	0.233322
O	0.943586	0.460680	1.160201
C	0.695554	-0.879742	0.761734
C	-0.078596	-1.497767	1.915346
O	1.732190	-2.891611	-0.127764
S	0.726988	1.572002	-1.330650
C	-0.698843	2.410476	-0.654844
C	-0.637218	3.769305	-0.361910
C	-1.754781	4.417938	0.143730
C	-2.936580	3.716240	0.344211
C	-3.000216	2.362351	0.043958
C	-1.881533	1.706822	-0.452629
O	3.813697	0.597567	1.064956
C	4.493942	1.722007	1.361505
O	4.417776	2.726999	0.700505
C	5.324483	1.542262	2.593990
O	-0.399959	-2.846594	1.599781
C	-1.771881	-3.196860	1.757968
C	-2.679848	-2.439727	0.821178
C	-3.744807	-1.727191	1.292799
C	-4.602867	-1.013419	0.414178
C	-4.331963	-1.033166	-0.973868
C	-3.217679	-1.780593	-1.443428
C	-2.419773	-2.462911	-0.575090
C	-5.711600	-0.267933	0.889294
C	-6.506894	0.425884	0.021600
C	-6.232850	0.410424	-1.364897
C	-5.172591	-0.301713	-1.850033
O	4.075036	-1.478489	-0.710469
C	4.714013	-1.311238	-1.882797
O	4.284414	-0.621162	-2.773518
C	5.996949	-2.083140	-1.914849
H	2.578935	-1.738869	1.376932
H	2.287280	-0.834141	-1.511025
H	3.491922	1.193871	-0.890493
H	1.803587	2.204740	0.719567
H	0.070503	-0.894772	-0.140346
H	-0.981243	-0.904059	2.081480
H	0.527036	-1.474314	2.827044
H	1.018707	-3.294074	0.388293
H	0.282942	4.316123	-0.534407
H	-1.703894	5.476141	0.372571
H	-3.810478	4.226910	0.732261
H	-3.922604	1.810627	0.193368
H	-1.929777	0.648138	-0.686414
H	5.890974	2.448931	2.787773
H	4.673598	1.322368	3.441004
H	5.999744	0.696381	2.464466
H	-2.082764	-3.035209	2.795654
H	-1.814809	-4.267735	1.553466
H	-3.949244	-1.694731	2.359400
H	-3.012452	-1.799185	-2.508718
H	-1.568675	-3.026341	-0.943782
H	-5.916776	-0.256662	1.954725
H	-7.352595	0.992939	0.393207
H	-6.869840	0.967545	-2.042094
H	-4.958359	-0.316049	-2.913573
H	6.489766	-1.933246	-2.871537
H	5.790626	-3.142544	-1.760873
H	6.645570	-1.749037	-1.104458

ωB97X Energy = -1970.66678275 a.u.
Cremer-Pople parameters:
φ (°), θ (°), Q: 237.162, 0.345, 0.537

26, Conf A
Charge: 0

C	-2.122007	-1.508759	0.336271
C	-3.157738	-0.393719	0.267217
C	-2.638700	0.834615	-0.476960
C	-1.246353	1.215939	0.037254
O	-0.378255	0.108519	-0.018170
C	-0.788316	-0.952435	0.835020
C	0.327279	-1.968816	0.884721
O	0.474792	-2.589566	-0.376547
C	2.915637	-2.522105	-0.515150
C	3.993124	-2.806386	0.314725
C	5.150056	-2.036982	0.262563
C	5.233546	-0.968894	-0.618165
C	4.157237	-0.674735	-1.448521
C	3.007663	-1.448196	-1.398348
C	1.658180	-3.362771	-0.474965
C	-5.411836	-1.143008	0.466991
O	-4.326992	-0.837970	-0.392300
C	-4.117146	2.423510	-1.456747
O	-3.539078	1.900922	-0.273184
C	-2.585935	-3.820988	0.675024
O	-2.589259	-2.511863	1.218974
S	-0.555800	2.548003	-0.987602
C	0.910697	2.936055	-0.041107
C	0.941484	4.076411	0.754606
C	2.088542	4.389497	1.472546
C	3.202150	3.563894	1.401256
C	3.171409	2.425169	0.605854
C	2.031605	2.114152	-0.120757
H	-1.977154	-1.917893	-0.669061

H	-3.400797	-0.083436	1.292829
H	-2.563005	0.584658	-1.542766
H	-1.326052	1.583896	1.068695
H	-0.928392	-0.571728	1.857897
H	0.099482	-2.718985	1.653528
H	1.248631	-1.448880	1.171785
H	3.927207	-3.634500	1.013773
H	5.982851	-2.269473	0.916636
H	6.133111	-0.365035	-0.658845
H	4.217231	0.159820	-2.138821
H	2.164200	-1.213029	-2.039781
H	1.557435	-3.932018	-1.400902
H	1.716762	-4.078406	0.355715
H	-6.240831	-1.454420	-0.167507
H	-5.158022	-1.953186	1.156443
H	-5.715278	-0.260777	1.041569
H	-4.803654	3.212279	-1.152612
H	-3.355172	2.851912	-2.117445
H	-4.672273	1.649899	-1.996143
H	-1.578925	-4.124748	0.370167
H	-2.944350	-4.490894	1.455538
H	-3.254955	-3.886647	-0.189740
H	0.067864	4.715472	0.811324
H	2.109144	5.279964	2.090257
H	4.095274	3.808786	1.964728
H	4.037258	1.774041	0.544701
H	2.002081	1.226245	-0.740492

ωB97X Energy = -1629.55566642 a.u.
Cremer-Pople parameters:
φ (°), θ (°), Q: 340.648, 7.357, 0.562

29, Conf A
Charge: 0

C	-1.623204	0.817518	-0.007517
C	-0.420791	-0.100497	0.074803
C	0.853827	0.701142	-0.216817
C	0.709276	1.557727	-1.486476
O	-0.471777	2.341071	-1.466469
C	-1.627295	1.536241	-1.350074
C	-3.952246	0.947868	0.061898
O	-2.831819	0.087437	0.132819
C	-2.868044	2.406077	-1.431194
O	-4.010636	1.595171	-1.188316
O	-0.272087	-0.679271	1.355261
C	-0.234386	-3.048907	0.734348
C	-0.932883	-3.759955	-0.234388
C	-0.303317	-4.757254	-0.971304
C	1.036045	-5.042248	-0.750473
C	1.743658	-4.332583	0.214077
C	1.107498	-3.350159	0.954835
C	-0.902490	-1.951453	1.526934
C	3.923359	-1.265332	0.329025
C	4.329038	-1.503165	-0.980154
C	5.305635	-2.451767	-1.250935
C	5.893154	-3.167523	-0.215661
C	5.497336	-2.929322	1.093702
C	4.514790	-1.985803	1.361788
C	2.872621	-0.233613	0.640637
O	1.924312	-0.191642	-0.414226
C	-5.199412	0.133086	0.250497
C	-5.510512	-0.882381	-0.648978
C	-6.658845	-1.637548	-0.473006
C	-7.503187	-1.382581	0.602443
C	-7.195197	-0.371040	1.499452
C	-6.043098	0.385875	1.322487
S	2.134059	2.628096	-1.819578
C	2.314143	3.559977	-0.303901
C	1.285585	4.357417	0.193026
C	1.482229	5.092873	1.353046
C	2.708669	5.062637	2.004413
C	3.740554	4.286607	1.496257
C	3.542876	3.529837	0.349134
H	-1.555069	1.568968	0.793868
H	-0.510999	-0.882992	-0.689905
H	1.043679	1.362306	0.637457
H	0.695604	0.884040	-2.353711
H	-1.655658	0.791540	-2.157632
H	-3.858009	1.708886	0.852843
H	-2.801542	3.212239	-0.688475
H	-2.988372	2.843738	-2.420574
H	-1.977879	-3.528368	-0.418066
H	-0.859024	-5.304841	-1.724204
H	1.530980	-5.812970	-1.330535
H	2.794754	-4.541290	0.386895
H	1.661301	-2.797090	1.706385
H	-1.962007	-1.885219	1.266465
H	-0.823677	-2.154227	2.596166
H	3.864424	-0.950335	-1.788288
H	5.609127	-2.633613	-2.275939
H	6.654643	-3.909164	-0.428676
H	5.947685	-3.485363	1.908318
H	4.198799	-1.814109	2.386559
H	2.373910	-0.480248	1.583659
H	3.337908	0.755035	0.753950
H	-4.849664	-1.076357	-1.486291
H	-6.898788	-2.427764	-1.175200
H	-8.401304	-1.974218	0.738688
H	-7.850609	-0.168845	2.338690
H	-5.801402	1.177409	2.024379
H	0.337034	4.394152	-0.327567
H	0.675984	5.704660	1.741326
H	2.860563	5.647166	2.904403
H	4.701812	4.261279	1.996373
H	4.344605	2.913413	-0.042198

ωB97X Energy = -2051.17633683 a.u.
Cremer-Pople parameters:
φ (°), θ (°), Q: 295.888, 7.708, 0.568

31, Conf A
Charge: 0

C	0.839813	-0.032332	-0.459463
C	-0.341585	0.855389	-0.817681
C	-1.612846	0.029108	-1.047227
C	-1.313125	-1.139899	-2.000607
O	-0.213033	-1.899596	-1.542473
C	0.993776	-1.156136	-1.481474
C	2.122452	-2.107403	-1.159892
C	-4.059382	1.303921	0.308990
C	-4.119051	2.673506	0.555691
C	-4.228405	3.155028	1.851177
C	-4.281235	2.267554	2.919784
C	-4.232977	0.901214	2.684497
C	-4.124923	0.422878	1.384445
C	-3.911005	0.795324	-1.102964
O	-2.575549	0.895814	-1.608608
O	2.037130	0.748766	-0.515896
O	2.275372	0.773761	1.724962
C	2.668097	1.078470	0.628032
C	3.933124	1.825835	0.334437
O	1.959186	-2.613735	0.146748

C	4.333006	-2.894350	0.672513
C	4.492963	-1.809924	1.533131
C	5.733837	-1.210633	1.685451
C	6.833374	-1.693365	0.983514
C	6.682429	-2.773964	0.127365
C	5.434874	-3.367671	-0.029295
C	2.972918	-3.535374	0.514062
O	-0.549956	1.781728	0.225635
C	0.503439	3.902487	-0.388903
C	1.256111	4.323593	0.706559
C	2.430423	5.037608	0.525267
C	2.872389	5.335531	-0.759015
C	2.133973	4.916487	-1.856112
C	0.953509	4.205983	-1.669299
C	-0.776625	3.133544	-0.171159
S	-2.697521	-2.261850	-2.335279
C	-3.265873	-2.770863	-0.719103
C	-4.640731	-2.919050	-0.549723
C	-5.150491	-3.355125	0.664941
C	-4.294854	-3.621837	1.724878
C	-2.924808	-3.471436	1.555694
C	-2.403700	-3.065086	0.334654
H	0.716881	-0.447291	0.541189
H	-0.101785	1.378594	-1.753180
H	-1.939996	-0.358968	-0.077325
H	-1.096710	-0.721460	-2.993756
H	1.202966	-0.714140	-2.467127
H	2.113872	-2.925623	-1.891432
H	3.073393	-1.569847	-1.257023
H	-4.079378	3.367522	-0.278462
H	-4.275958	4.223460	2.029860
H	-4.366522	2.642398	3.933474
H	-4.280100	0.203920	3.513429
H	-4.096877	-0.648927	1.206617
H	-4.264433	-0.235734	-1.174098
H	-4.504745	1.403811	-1.785957
H	4.684559	1.109282	-0.006745
H	4.288174	2.312410	1.239687
H	3.771423	2.560399	-0.453930
H	3.635497	-1.426632	2.076708
H	5.845758	-0.366329	2.357054
H	7.804236	-1.226451	1.104991
H	7.534425	-3.153518	-0.425340
H	5.317886	-4.207245	-0.707735
H	3.028053	-4.346810	-0.222980
H	2.643803	-3.962406	1.462749
H	0.910647	4.093881	1.709738
H	3.002342	5.365883	1.385971
H	3.789007	5.896544	-0.902175
H	2.471832	5.148534	-2.859843
H	0.370887	3.893883	-2.530788
H	-1.345059	3.578768	0.647028
H	-1.401812	3.159142	-1.067816
H	-5.313245	-2.687323	-1.368637
H	-6.221438	-3.470912	0.784901
H	-4.693514	-3.949277	2.677917
H	-2.249352	-3.684387	2.376265
H	-1.332920	-2.973535	0.199274

ωB97X Energy = -2205.06312071 a.u.
Cremer-Pople parameters:
φ (°), θ (°), Q: 332.167, 5.213, 0.561

26cat, Conf A
Charge: +1

C	2.208685	-0.801997	0.030573
C	1.695996	0.617225	0.350663
C	0.485226	1.002051	-0.529656
O	0.385888	-1.191873	-1.517462
C	1.076994	-1.752204	-0.311622
C	0.060722	-1.985502	0.789272
O	-1.021046	-2.712571	0.260548
C	-2.665659	-0.920705	0.383402
C	-2.757981	0.101391	1.321157
C	-3.045127	1.404365	0.923681
C	-3.239365	1.693670	-0.418533
C	-3.155151	0.675960	-1.364082
C	-2.871386	-0.622171	-0.964184
C	-2.291378	-2.325773	0.791193
C	2.367830	1.023340	2.585925
O	1.294884	0.758181	1.690499
C	0.371462	3.334494	-0.206780
O	0.555423	2.258104	-1.124231
C	4.402048	-0.433576	-0.802999
O	3.051178	-0.772033	-1.094081
H	2.721639	-1.208293	0.910007
H	2.495103	1.326658	0.107104
H	-0.410635	0.921817	0.122125
H	1.461186	-2.690424	-0.702630
H	0.569335	-2.548994	1.580324
H	-0.262958	-1.039298	1.227937
H	-2.603650	-0.118995	2.372672
H	-3.115255	2.190958	1.666067
H	-3.463590	2.707051	-0.730591
H	-3.322518	0.894941	-2.412508
H	-2.811482	-1.416832	-1.701253
H	-2.997101	-3.049446	0.384572
H	-2.291846	-2.420061	1.882134
H	2.872871	1.955592	2.313474
H	3.098089	0.208206	2.598683
H	1.931406	1.123284	3.576634
H	1.174738	3.365873	0.534538
H	-0.592422	3.240897	0.303253
H	0.388964	4.246872	-0.796939
H	4.842810	-1.169573	-0.124248
H	4.485542	0.563636	-0.359729
H	4.937464	-0.445171	-1.749159
C	0.191804	0.033577	-1.608154
H	-0.246625	0.377961	-2.543804

ωB97X Energy = -999.477105130 a.u.
Cremer-Pople parameters:
φ (°), θ (°), Q: 93.973, 106.895, 0.575

26cat, Conf B
Charge: +1

C	-1.430104	0.264699	-1.318551
C	-1.758053	0.670241	0.135210
C	-2.110651	-0.542311	1.014347
O	-1.850824	-2.096703	-0.818578
C	-0.909720	-1.153064	-1.497942
C	0.495425	-1.495542	-1.064924
O	0.601434	-1.407350	0.331669
C	2.923369	-0.639016	0.437795
C	4.175522	-1.002301	-0.041941
C	5.115611	-0.030048	-0.363262
C	4.802576	1.313178	-0.217009
C	3.547058	1.682371	0.253249
C	2.613801	0.711725	0.583431
C	1.910268	-1.691326	0.820696
C	-0.661720	2.743414	0.437635
O	-0.718957	1.364635	0.774681

C	-2.876258	0.482991	3.000456
O	-3.179500	-0.355244	1.887220
C	-2.928465	1.623258	-2.568540
O	-2.600097	0.321113	-2.104399
H	-0.653178	0.932217	-1.711197
H	-2.662038	1.290175	0.097359
H	-1.175835	-0.808149	1.545185
H	-1.029969	-1.424049	-2.544106
H	0.737173	-2.509238	-1.406727
H	1.168310	-0.795100	-1.575335
H	4.419952	-2.052542	-0.167253
H	6.090620	-0.324113	-0.734636
H	5.532947	2.072593	-0.471684
H	3.297475	2.731602	0.367201
H	1.628012	0.991730	0.941358
H	1.799944	-1.742510	1.904561
H	2.232702	-2.676560	0.464135
H	-1.583674	3.254461	0.734554
H	-0.495387	2.895890	-0.633999
H	0.177967	3.166018	0.986009
H	-3.751254	0.463735	3.645060
H	-2.680062	1.509899	2.682192
H	-2.007555	0.099928	3.544241
H	-3.130202	2.313916	-1.743093
H	-3.828610	1.523591	-3.170224
H	-2.119392	2.026042	-3.185753
C	-2.391053	-1.778519	0.256981
H	-3.090024	-2.509650	0.661604

ωB97X Energy = -999.476663736 a.u.
Cremer-Pople parameters:
φ (°), θ (°), Q: 100.323, 103.279, 0.515

26cat, Conf C
Charge: +1

C	-0.724616	0.186403	-0.724646
C	-2.179894	0.530136	-0.426535
C	-2.751514	-0.534793	0.509538
O	-1.554213	-2.195398	-0.793365
C	-0.577606	-1.200705	-1.349226
C	0.782396	-1.815301	-1.140997
O	0.967486	-2.002643	0.240134
C	3.044177	-0.798522	0.643706
C	3.952360	-0.451467	-0.350642
C	4.563906	0.797171	-0.344311
C	4.270560	1.707775	0.659929
C	3.366322	1.368062	1.660391
C	2.757821	0.122913	1.650313
C	2.337759	-2.130202	0.630447
C	-2.748353	2.833336	-0.609932
O	-2.279479	1.770671	0.215530
C	-4.522249	0.199190	1.917033
O	-4.126909	-0.441689	0.700396
C	0.746094	2.018244	-1.138343
O	-0.177564	1.069890	-1.670309
H	-0.156326	0.219819	0.211808
H	-2.759306	0.525671	-1.357570
H	-2.198619	-0.489290	1.466051
H	-0.834283	-1.167584	-2.407220
H	0.863080	-2.764155	-1.681599
H	1.505446	-1.109176	-1.567468
H	4.191039	-1.164716	-1.133614
H	5.271637	1.055563	-1.123475
H	4.749561	2.680035	0.668482
H	3.141342	2.074363	2.451257
H	2.055462	-0.143324	2.434251
H	2.308750	-2.559735	1.631472
H	2.848205	-2.836675	-0.032719
H	-2.081076	2.997384	-1.460016
H	-3.758814	2.622777	-0.972846
H	-2.768297	3.724123	0.014285
H	-4.185497	-0.378731	2.782135
H	-5.608822	0.229793	1.903905
H	-4.121655	1.213171	1.962986
H	0.269252	2.650984	-0.385086
H	1.073014	2.631330	-1.975490
H	1.612527	1.514914	-0.696764
C	-2.473031	-1.899620	-0.012368
H	-3.115989	-2.728903	0.282023

ωB97X Energy = -999.475800958 a.u.
Cremer-Pople parameters:
φ (°), θ (°), Q: 192.260, 50.258, 0.480

26cat, Conf D
Charge: +1

C	2.542947	-0.550527	0.493194
C	1.516480	0.565715	0.698323
C	0.828605	0.908087	-0.655893
O	1.695018	-1.129237	-1.702084
C	2.021897	-1.691940	-0.362643
C	0.790897	-2.455662	0.108498
O	-0.343402	-1.698641	-0.222656
C	-2.559208	-0.970101	0.217569
C	-3.283232	-0.325656	1.213944
C	-4.273982	0.591259	0.885397
C	-4.540676	0.879361	-0.445176
C	-3.818443	0.241167	-1.446568
C	-2.836933	-0.681946	-1.117163
C	-1.489414	-1.965414	0.576051
C	-0.146814	1.187885	2.274146
O	0.640235	0.153448	1.707980
C	0.246986	3.210329	-0.610286
O	1.088353	2.192490	-1.142837
C	4.616116	0.616544	0.615677
O	3.662424	-0.053056	-0.200101
H	2.813902	-0.965530	1.470053
H	2.051052	1.474392	1.001469
H	-0.254300	0.717020	-0.545291
H	2.840137	-2.373016	-0.580832
H	0.758335	-3.437500	-0.373955
H	0.883571	-2.598813	1.190751
H	-3.071206	-0.539411	2.256820
H	-4.832709	1.085660	1.671762
H	-5.309752	1.598229	-0.703116
H	-4.025676	0.459241	-2.488097
H	-2.277699	-1.182791	-1.900164
H	-1.829123	-2.992104	0.391858
H	-1.227261	-1.871740	1.635345
H	0.485312	2.011169	2.623899
H	-0.673547	0.754093	3.121681
H	-0.887010	1.570902	1.562495
H	0.482655	4.121195	-1.154536
H	0.441154	3.369315	0.454251
H	-0.807623	2.957557	-0.759191
H	5.430360	0.914958	-0.040208
H	4.997638	-0.056091	1.389293
H	4.194456	1.509293	1.086316
C	1.194287	0.006784	-1.783960
H	1.019221	0.358633	-2.799692

ωB97X Energy = -999.475613070 a.u.
Cremer-Pople parameters:

φ (°), θ (°), Q: 84.729, 115.984, 0.525

26cat, Conf E
Charge: +1

C	0.533041	0.298082	1.064145
C	1.887143	0.713805	0.501334
C	2.367201	-0.397113	-0.449717
O	1.680277	-1.969009	1.251372
C	0.516409	-1.117850	1.656403
C	-0.703936	-1.907744	1.252948
O	-0.650817	-2.072101	-0.145084
C	-2.660585	-0.874383	-0.822156
C	-3.771092	-0.635462	-0.020476
C	-4.403041	0.603055	-0.040372
C	-3.925193	1.612272	-0.862753
C	-2.816712	1.381391	-1.670435
C	-2.190663	0.145291	-1.649432
C	-1.931496	-2.193086	-0.775956
C	2.798956	2.832703	-0.086073
O	1.717976	1.910001	-0.197917
C	3.805347	-0.507797	-2.330649
O	3.622139	-0.105610	-0.973604
C	-0.808959	2.153431	1.719842
O	0.112501	1.136122	2.108995
H	-0.182427	0.336591	0.235002
H	2.609920	0.833566	1.318003
H	1.600514	-0.522894	-1.233213
H	0.625303	-1.065802	2.737051
H	-0.733376	-2.875926	1.761663
H	-1.571731	-1.318353	1.571030
H	-4.150936	-1.424667	0.621227
H	-5.269178	0.777187	0.587483
H	-4.417532	2.577733	-0.880091
H	-2.445038	2.165650	-2.319765
H	-1.327900	-0.036149	-2.283545
H	-1.716446	-2.553527	-1.781543
H	-2.528951	-2.950475	-0.258533
H	2.512710	3.711941	-0.659249
H	2.953409	3.117535	0.959024
H	3.719717	2.411769	-0.494495
H	4.806529	-0.193151	-2.613289
H	3.068713	-0.018977	-2.974210
H	3.724453	-1.593625	-2.439366
H	-0.350234	2.851053	1.016787
H	-1.088902	2.678134	2.630915
H	-1.703739	1.712255	1.265941
C	2.434382	-1.675575	0.308778
H	3.223690	-2.401207	0.110473

ωB97X Energy = -999.475603525 a.u.
Cremer-Pople parameters:
φ (°), θ (°), Q: 167.213, 62.353, 0.530

26cat, Conf F
Charge: +1

C	-0.832197	1.155533	-0.589646
C	-1.809642	0.629956	0.481708
C	-2.357041	-0.771258	0.195445
O	-2.038571	-0.406248	-2.164690
C	-0.779321	0.361200	-1.904490
C	0.367958	-0.611901	-2.012542
O	0.292932	-1.507347	-0.929125
C	2.524364	-1.197009	-0.000570
C	3.789771	-1.026715	-0.547995
C	4.695244	-0.143649	0.029848
C	4.334464	0.582552	1.154440
C	3.066981	0.421843	1.704517
C	2.168957	-0.465761	1.132787
C	1.531260	-2.151346	-0.616834
C	-0.964518	1.821705	2.346129
O	-1.227431	0.556339	1.754152
C	-3.263352	-1.857680	2.103363
O	-3.499125	-1.075666	0.930393
C	-0.119215	3.298070	-1.314112
O	-1.186729	2.497148	-0.828433
H	0.176651	1.099344	-0.160293
H	-2.663518	1.319591	0.490436
H	-1.537547	-1.492638	0.354599
H	-0.762720	1.042183	-2.752521
H	0.336306	-1.145670	-2.968615
H	1.284795	-0.010411	-1.981980
H	4.072458	-1.587778	-1.433268
H	5.680784	-0.020605	-0.404246
H	5.038145	1.273419	1.604329
H	2.782327	0.985063	2.586307
H	1.179268	-0.587944	1.563929
H	1.266272	-2.943511	0.083768
H	1.947397	-2.615713	-1.517126
H	-0.614863	1.625174	3.357217
H	-1.873414	2.429906	2.381958
H	-0.189075	2.367575	1.798695
H	-2.810128	-2.817451	1.839116
H	-4.237580	-2.030468	2.553969
H	-2.620137	-1.320129	2.801433
H	0.232343	2.963748	-2.296373
H	0.721908	3.287853	-0.612113
H	-0.502490	4.311482	-1.406188
C	-2.696257	-0.908612	-1.235722
H	-3.580527	-1.468401	-1.539374

ωB97X Energy = -999.475550371 a.u.
Cremer-Pople parameters:
φ (°), θ (°), Q: 130.833, 83.913, 0.496

26cat, Conf G
Charge: +1

C	-2.197864	-0.737993	-0.350137
C	-1.977930	0.721933	0.080438
C	-0.740573	0.849522	0.989478
O	-1.382882	-1.248284	1.991810
C	-1.722618	-1.805285	0.647561
C	-0.502456	-2.580875	0.208875
O	0.533141	-1.645279	0.057880
C	2.720561	-1.025711	-0.572564
C	3.869792	-0.998505	0.207063
C	4.741065	0.082515	0.138593
C	4.460987	1.147645	-0.704166
C	3.310754	1.128299	-1.485645
C	2.448007	0.045317	-1.422179
C	1.748158	-2.167194	-0.479028
C	-2.358679	2.804422	-1.020984
O	-1.804907	1.492161	-1.075048
C	0.726521	2.577791	1.651933
O	-0.623378	2.143005	1.489593
C	-4.116700	-0.433639	-1.734836
O	-3.555771	-1.029103	-0.571909
H	-1.624958	-0.870380	-1.277309
H	-2.859055	1.054301	0.643636
H	0.136277	0.537328	0.402500
H	-2.548985	-2.474543	0.869984
H	-0.246609	-3.345760	0.950607

H	-0.750891	-3.079160	-0.736248
H	4.087640	-1.827125	0.872982
H	5.637378	0.092253	0.748000
H	5.138766	1.991889	-0.756967
H	3.091466	1.956184	-2.150168
H	1.552579	0.028466	-2.036152
H	2.133972	-2.957634	0.173472
H	1.545011	-2.598148	-1.466421
H	-3.434917	2.760805	-0.821691
H	-2.194727	3.246532	-2.001286
H	-1.870487	3.409197	-0.255493
H	0.682383	3.608003	1.995600
H	1.261893	2.525658	0.698666
H	1.251619	1.970906	2.396481
H	-3.477523	-0.606146	-2.606229
H	-4.264606	0.642066	-1.609367
H	-5.081822	-0.911456	-1.889330
C	-0.885929	-0.112467	2.109763
H	-0.632599	0.172119	3.131149

ωB97X Energy = -999.475520735 a.u.
Cremer-Pople parameters:
φ (°), θ (°), Q: 144.945, 76.761, 0.550

26cat, Conf H
Charge: +1

C	2.172460	-0.971510	0.642727
C	2.035252	0.541331	0.774737
C	1.670610	1.158505	-0.605564
O	0.854089	-0.992041	-1.407017
C	0.954093	-1.591651	-0.032851
C	-0.384991	-1.455887	0.647557
O	-1.287722	-2.281358	-0.044991
C	-3.219883	-0.785865	0.081764
C	-3.758246	0.018170	1.078800
C	-4.303044	1.259212	0.768653
C	-4.303498	1.707912	-0.543360
C	-3.761380	0.911200	-1.546379
C	-3.225332	-0.328634	-1.234932
C	-2.631482	-2.138461	0.406806
C	1.118639	2.094625	2.315185
O	1.061059	0.794134	1.747283
C	2.773480	3.250363	-0.710783
O	2.662592	1.922472	-1.215278
C	3.858399	-2.489999	-0.105581
O	3.320804	-1.172464	-0.139126
H	2.269598	-1.410502	1.641304
H	3.012499	0.941904	1.064687
H	0.737477	1.742623	-0.467977
H	1.125789	-2.638748	-0.273757
H	-0.265801	-1.776619	1.690768
H	-0.717521	-0.413571	0.661427
H	-3.753286	-0.327300	2.107920
H	-4.723265	1.875880	1.554894
H	-4.727192	2.675333	-0.787503
H	-3.765965	1.255789	-2.574336
H	-2.807056	-0.952940	-2.018100
H	-3.184284	-2.929587	-0.100469
H	-2.684445	-2.323212	1.486247
H	2.102320	2.279835	2.757294
H	0.358541	2.132309	3.091557
H	0.903095	2.870463	1.572611
H	3.537174	3.742546	-1.306847
H	3.082730	3.251922	0.338361
H	1.824342	3.784297	-0.817687
H	4.812909	-2.447173	-0.624124
H	3.211551	-3.208203	-0.617238
H	4.018198	-2.812323	0.927133
C	1.254189	0.163539	-1.629531
H	1.250418	0.464235	-2.676617

ωB97X Energy = -999.473516387 a.u.
Cremer-Pople parameters:
φ (°), θ (°), Q: 73.951, 115.589, 0.541

26cat, Conf I
Charge: +1

C	1.546293	-0.222160	1.328995
C	1.682203	0.809542	0.205951
C	1.973552	0.110909	-1.148658
O	1.683060	-2.112599	-0.203618
C	0.893117	-1.532730	0.922962
C	-0.565848	-1.567532	0.520318
O	-0.726007	-0.967074	-0.739156
C	-3.105559	-0.392072	-0.542917
C	-2.841163	0.844073	0.040371
C	-3.850438	1.539376	0.691170
C	-5.133613	1.010774	0.758097
C	-5.400658	-0.222266	0.180029
C	-4.387855	-0.923108	-0.461783
C	-2.025552	-1.137689	-1.292262
C	0.659925	2.759887	-0.646866
O	0.532949	1.603277	0.169391
C	4.266143	0.703695	-1.456724
O	2.934118	0.700389	-1.969524
C	3.391966	0.290717	2.744321
O	2.833245	-0.588670	1.775894
H	0.941048	0.211912	2.132347
H	2.550102	1.434229	0.444424
H	1.019153	0.137986	-1.700830
H	1.059726	-2.252010	1.721364
H	-0.896275	-2.612191	0.487002
H	-1.136633	-1.053842	1.303279
H	-1.833895	1.247057	-0.003838
H	-3.635637	2.499473	1.146969
H	-5.921497	1.556243	1.264724
H	-6.397556	-0.644535	0.234929
H	-4.598768	-1.892355	-0.903110
H	-1.947539	-0.765634	-2.315037
H	-2.268513	-2.206068	-1.338866
H	1.512496	3.367663	-0.325593
H	-0.258674	3.330682	-0.528122
H	0.785133	2.497606	-1.702137
H	4.899865	1.065777	-2.261817
H	4.591312	-0.304047	-1.175215
H	4.369025	1.367856	-0.595836
H	3.508039	1.306536	2.353676
H	4.372787	-0.105413	2.995897
H	2.766872	0.318578	3.641452
C	2.180867	-1.358394	-1.060848
H	2.760237	-1.866214	-1.832489

ωB97X Energy = -999.473400726 a.u.
Cremer-Pople parameters:
φ (°), θ (°), Q: 92.555, 108.189, 0.521

26cat, Conf J
Charge: +1

C	0.769341	-0.287416	-0.251883
C	1.877024	-0.725187	0.719788
C	3.199642	-0.022345	0.381470
O	1.902329	1.943304	0.000294

C	1.000455	1.104228	-0.857845
C	-0.261130	1.905465	-1.029141
O	-0.836219	2.123828	0.235965
C	-2.919972	0.874690	0.103729
C	-2.897464	-0.001438	1.188894
C	-3.457071	-1.265436	1.087958
C	-4.049059	-1.669862	-0.104078
C	-4.080790	-0.804484	-1.187259
C	-3.517575	0.462797	-1.082279
C	-2.262563	2.226351	0.225729
C	0.721287	-1.129133	2.765815
O	1.617096	-0.300019	2.036992
C	4.097000	-1.431281	-1.294900
O	3.605592	-0.134398	-0.953084
C	-0.224188	-2.232215	-1.162744
O	0.664924	-1.140665	-1.365541
H	-0.175444	-0.261790	0.301793
H	1.999381	-1.811853	0.659927
H	3.982088	-0.322327	1.083327
H	1.540497	0.999104	-1.796449
H	-0.050040	2.854816	-1.531715
H	-0.905682	1.304478	-1.682489
H	-2.444771	0.319004	2.122685
H	-3.440731	-1.934633	1.940778
H	-4.491343	-2.656263	-0.183291
H	-4.546737	-1.112699	-2.116062
H	-3.554860	1.140636	-1.929578
H	-2.585295	2.891346	-0.582128
H	-2.520320	2.692859	1.176362
H	1.124730	-2.142575	2.850318
H	0.623790	-0.691670	3.756408
H	-0.265814	-1.169147	2.293863
H	4.523341	-1.342016	-2.290597
H	4.871928	-1.744683	-0.589978
H	3.284669	-2.161575	-1.311890
H	-0.243059	-2.797411	-2.091827
H	0.119820	-2.887156	-0.354885
H	-1.232850	-1.872014	-0.931953
C	2.885926	1.437000	0.562585
H	3.471743	2.118757	1.175332

ωB97X Energy = -999.472113073 a.u.
Cremer-Pople parameters:
φ (°), θ (°), Q: 313.948, 102.043, 0.670

26cat, Conf K
Charge: +1

C	-1.522443	1.054775	0.851355
C	-2.189636	-0.316631	0.989845
C	-2.732153	-0.811019	-0.365372
O	-1.588218	0.954668	-1.571746
C	-0.662502	1.137676	-0.405686
C	0.488955	0.169669	-0.510569
O	1.225147	0.520096	-1.653300
C	3.381770	-0.322129	-0.857187
C	4.003341	0.886288	-0.548158
C	4.992188	0.939607	0.421379
C	5.375137	-0.217992	1.090446
C	4.761949	-1.424230	0.787926
C	3.765127	-1.473063	-0.179766
C	2.307739	-0.369004	-1.916486
C	-1.146165	-1.304965	2.876637
O	-1.309780	-1.302310	1.462905
C	-4.421306	-2.334875	-1.038888
O	-4.071399	-1.179934	-0.276482
C	-2.216150	3.341188	0.938725
O	-2.581543	1.972997	0.794876
H	-0.870055	1.242127	1.711681
H	-3.053910	-0.197232	1.649917
H	-2.092813	-1.668156	-0.647001
H	-0.295575	2.146559	-0.580180
H	1.082378	0.270893	0.407800
H	0.149409	-0.871859	-0.560941
H	3.707108	1.789047	-1.072880
H	5.471732	1.883732	0.653326
H	6.151577	-0.177022	1.845672
H	5.055438	-2.329402	1.306993
H	3.282543	-2.417680	-0.410667
H	1.928996	-1.392643	-2.022337
H	2.702657	-0.049555	-2.881064
H	-2.110581	-1.442333	3.373884
H	-0.682075	-0.381439	3.234824
H	-0.493215	-2.142037	3.111343
H	-5.474991	-2.520493	-0.849127
H	-3.830008	-3.196562	-0.717276
H	-4.273966	-2.167858	-2.109807
H	-3.141384	3.894151	1.079975
H	-1.709971	3.723940	0.047981
H	-1.572873	3.477132	1.812913
C	-2.535973	0.152109	-1.484853
H	-3.223346	0.158147	-2.331152

ωB97X Energy = -999.472020356 a.u.
Cremer-Pople parameters:
φ (°), θ (°), Q: 87.705, 108.900, 0.580

26cat, Conf L
Charge: +1

C	-2.019540	-0.728645	-0.594843
C	-2.024249	0.722699	-0.105542
C	-0.814686	0.927642	0.830739
O	-1.420675	-1.176985	1.843999
C	-1.672791	-1.772074	0.492831
C	-0.424369	-2.565243	0.180312
O	0.622244	-1.631373	0.111069
C	2.829131	-1.005413	-0.456486
C	2.571008	0.057728	-1.320577
C	3.436541	1.138493	-1.381891
C	4.575730	1.163073	-0.584337
C	4.841568	0.105668	0.272449
C	3.967002	-0.972863	0.339102
C	1.859877	-2.150067	-0.374765
C	-2.652156	2.768962	-1.146677
O	-1.928443	1.543687	-1.227308
C	0.543508	2.727117	1.533248
O	-0.776649	2.230232	1.314631
C	-4.417808	-0.909588	-0.590867
O	-3.187112	-1.115495	-1.269663
H	-1.232947	-0.777093	-1.350207
H	-2.935717	0.946189	0.462587
H	0.096088	0.651378	0.277052
H	-2.514305	-2.434009	0.679580
H	-0.238692	-3.320458	0.951494
H	-0.588228	-3.072682	-0.778254
H	1.686312	0.034829	-1.950168
H	3.228853	1.959998	-2.057973
H	5.256086	2.005315	-0.635851
H	5.729234	0.119340	0.894325
H	4.173723	-1.795551	1.015841
H	1.693869	-2.597649	-1.361835
H	2.228557	-2.927905	0.302211
H	-2.264919	3.404487	-0.348534

H	-3.717446	2.576987	-0.982277
H	-2.523927	3.263937	-2.106718
H	0.438054	3.760930	1.851479
H	1.127344	2.679906	0.608889
H	1.055820	2.158392	2.315439
H	-5.168707	-1.473690	-1.139008
H	-4.387584	-1.277250	0.440890
H	-4.702793	0.146040	-0.586987
C	-0.958926	-0.026446	1.962115
H	-0.752638	0.281771	2.987340

ωB97X Energy = -999.471814811 a.u.
Cremer-Pople parameters:
φ (°), θ (°), Q: 154.279, 71.633, 0.579

26cat, Conf M
Charge: +1

C	1.982240	0.346373	1.312442
C	2.798678	0.760574	0.086725
C	2.865285	-0.415760	-0.908755
O	0.744777	-1.279973	-0.036116
C	0.596111	-0.129931	0.926982
C	-0.380321	0.857364	0.336141
O	-1.649989	0.258125	0.377272
C	-3.979356	0.377247	-0.156278
C	-4.057677	-0.987356	-0.418242
C	-5.287798	-1.628862	-0.432868
C	-6.453333	-0.911701	-0.194200
C	-6.381126	0.449419	0.065797
C	-5.148523	1.088550	0.089606
C	-2.655746	1.091797	-0.177841
C	3.042433	2.693384	-1.260490
O	2.178236	1.883841	-0.473669
C	4.768211	-1.356818	-1.996080
O	4.037318	-1.170875	-0.782094
C	3.787347	-0.471375	2.648661
O	2.572582	-0.751625	1.961010
H	1.872011	1.207326	1.981248
H	3.827190	0.985188	0.385496
H	2.747997	-0.002859	-1.922500
H	0.138738	-0.630217	1.777862
H	-0.345091	1.766170	0.950706
H	-0.099615	1.138446	-0.684004
H	-3.148556	-1.548512	-0.600942
H	-5.337504	-2.693261	-0.632838
H	-7.413966	-1.413607	-0.206902
H	-7.285214	1.015273	0.259471
H	-5.096681	2.151177	0.305148
H	-2.723288	2.026796	0.391649
H	-2.382286	1.350231	-1.210167
H	3.876320	3.063014	-0.656967
H	2.450167	3.531875	-1.618443
H	3.436854	2.143834	-2.121968
H	5.648239	-1.939574	-1.737394
H	5.074320	-0.392133	-2.410433
H	4.174366	-1.903706	-2.733221
H	3.683365	0.425665	3.266239
H	4.622470	-0.349064	1.954543
H	3.983320	-1.328135	3.288960
C	1.744580	-1.395645	-0.765134
H	1.787411	-2.323437	-1.335563

ωB97X Energy = -999.471286562 a.u.
Cremer-Pople parameters:
φ (°), θ (°), Q: 62.869, 122.354, 0.509

26cat, Conf N
Charge: +1

C	2.276472	0.499130	-0.748120
C	1.654373	-0.850138	-0.299667
C	0.869880	-0.790670	1.018049
O	1.805094	1.374537	1.553312
C	1.908091	1.721895	0.099174
C	0.630945	2.437202	-0.274083
O	-0.433378	1.534251	-0.140792
C	-2.738838	0.996037	-0.364564
C	-3.925298	1.307162	0.287961
C	-4.900075	0.335112	0.478427
C	-4.687664	-0.958971	0.027187
C	-3.499648	-1.277487	-0.620970
C	-2.532030	-0.304532	-0.821918
C	-1.687732	2.048830	-0.582409
C	1.435081	-1.963688	-2.371304
O	0.772566	-1.367401	-1.263385
C	-0.097291	-2.933086	1.344691
O	0.879368	-1.973435	1.753856
C	4.449972	0.229127	0.237770
O	3.670871	0.404182	-0.935708
H	1.875315	0.706894	-1.741424
H	2.478609	-1.556214	-0.140099
H	-0.154837	-0.461016	0.765703
H	2.729706	2.435178	0.087247
H	0.492984	3.312319	0.371776
H	0.745815	2.786997	-1.307658
H	-4.090489	2.315859	0.652626
H	-5.822771	0.589682	0.987099
H	-5.444814	-1.719456	0.179920
H	-3.329645	-2.287660	-0.976532
H	-1.603618	-0.557350	-1.325386
H	-1.933700	2.958434	-0.023481
H	-1.608486	2.309946	-1.645024
H	2.067795	-2.793473	-2.039850
H	2.051356	-1.238390	-2.911971
H	0.659811	-2.337905	-3.035829
H	-0.068280	-3.729921	2.083739
H	0.135919	-3.333257	0.356568
H	-1.094330	-2.481197	1.329403
H	4.398145	1.102781	0.896331
H	5.479292	0.104656	-0.088854
H	4.154442	-0.663449	0.800666
C	1.347534	0.275764	1.920951
H	1.302989	0.129344	2.999708

ωB97X Energy = -999.470899976 a.u.
Cremer-Pople parameters:
φ (°), θ (°), Q: 116.502, 94.271, 0.468

26cat, Conf O
Charge: +1

C	0.896263	0.399200	-0.037678
C	2.370991	0.815517	0.023121
C	3.328589	-0.373954	-0.169791
O	1.523729	-1.920882	-0.486602
C	0.695708	-1.047491	0.402613
C	-0.715884	-1.549467	0.281044
O	-1.159373	-1.325786	-1.033360
C	-3.448205	-0.661691	-0.496697
C	-3.319181	0.708098	-0.717344
C	-4.143674	1.607395	-0.060555
C	-5.114322	1.145354	0.822055
C	-5.250906	-0.216320	1.045023
C	-4.416076	-1.115206	0.390540

C	-2.538729	-1.627389	-1.217964
C	3.651816	2.671110	-0.708821
O	2.571394	1.786707	-0.971115
C	5.117129	-1.095505	1.272538
O	3.704860	-0.959548	1.065911
C	-0.196707	2.471081	0.359628
O	0.081669	1.152339	0.814097
H	0.560387	0.489331	-1.079645
H	2.575980	1.214996	1.025503
H	4.190740	-0.071933	-0.765548
H	1.081471	-1.223725	1.407914
H	-1.313703	-0.997012	1.013993
H	-0.749951	-2.616256	0.534337
H	-2.563398	1.067047	-1.409020
H	-4.035349	2.671302	-0.239664
H	-5.761742	1.848029	1.334001
H	-6.003563	-0.582066	1.734103
H	-4.519110	-2.180204	0.574446
H	-2.697976	-1.573646	-2.295474
H	-2.744706	-2.654400	-0.893682
H	3.691561	3.374029	-1.537618
H	4.609078	2.141538	-0.649112
H	3.486209	3.216499	0.225913
H	5.566525	-1.732705	0.508264
H	5.233595	-1.559334	2.248281
H	5.593417	-0.112601	1.269759
H	0.688814	3.109583	0.415881
H	-0.561359	2.455278	-0.672342
H	-0.972057	2.865419	1.013321
C	2.688458	-1.584088	-0.768739
H	3.235281	-2.291681	-1.388885

ωB97X Energy = -999.469499928 a.u.
Cremer-Pople parameters:
φ (°), θ (°), Q: 280.907, 77.280, 0.594

26cat, Conf P
Charge: +1

C	2.250246	-0.757990	0.356286
C	1.714893	0.698862	0.377255
C	0.484923	0.953293	-0.519986
O	0.545525	-1.302858	-1.361063
C	1.193743	-1.762800	-0.090705
C	0.110123	-1.980466	0.952847
O	-0.947045	-2.707350	0.382009
C	-2.611487	-0.933595	0.388726
C	-2.756240	0.116818	1.287131
C	-3.041891	1.401987	0.833706
C	-3.182634	1.644717	-0.524328
C	-3.045876	0.598205	-1.431171
C	-2.762220	-0.681869	-0.976155
C	-2.240004	-2.320329	0.855898
C	2.400881	1.458880	2.516417
O	1.319409	1.080326	1.673370
C	0.303405	3.308445	-0.404166
O	0.507568	2.156082	-1.221496
C	3.483188	-0.659230	-1.709772
O	3.478388	-0.894027	-0.309395
H	2.470132	-1.018838	1.393245
H	2.519555	1.349006	0.011955
H	-0.404685	0.893565	0.140560
H	1.640382	-2.703456	-0.402968
H	0.570754	-2.533499	1.779122
H	-0.228108	-1.020342	1.354926
H	-2.645799	-0.066282	2.351179
H	-3.152940	2.211575	1.545715
H	-3.405854	2.644290	-0.878718
H	-3.172086	0.780330	-2.492354
H	-2.666009	-1.500454	-1.682776
H	-2.922810	-3.064139	0.446854
H	-2.278342	-2.381375	1.948379
H	2.931805	2.319007	2.095656
H	3.109104	0.637665	2.663892
H	1.968171	1.730905	3.475973
H	1.125901	3.443467	0.302168
H	-0.638799	3.223299	0.145440
H	0.261246	4.157762	-1.080857
H	3.061691	0.322004	-1.963601
H	2.948049	-1.442220	-2.255649
H	4.524750	-0.670354	-2.020712
C	0.254651	-0.104482	-1.527294
H	-0.181068	0.148019	-2.492966

ωB97X Energy = -999.469404543 a.u.
Cremer-Pople parameters:
φ (°), θ (°), Q: 104.180, 103.031, 0.515

29cat, Conf A
Charge: +1

C	-0.694969	-0.728887	-1.180416
C	0.471821	0.245234	-1.177191
C	1.670810	-0.481421	-1.799922
O	0.833702	-2.644923	-0.978869
C	-0.341402	-1.977954	-0.388651
C	-2.930117	-1.062598	-0.589762
O	-1.828170	-0.161245	-0.569200
C	-1.518323	-2.936819	-0.397434
O	-2.618316	-2.227540	0.142691
O	0.230861	1.376033	-1.963836
C	0.784160	3.091243	-0.317974
C	1.935343	3.685532	-0.832565
C	2.923974	4.155354	0.017404
C	2.772612	4.036395	1.395006
C	1.627512	3.452964	1.916712
C	0.637069	2.983216	1.061030
C	-0.254710	2.528652	-1.252849
C	3.297908	-0.906951	0.527311
C	4.107774	-2.005448	0.220271
C	4.030412	-3.168565	0.970771
C	3.151223	-3.244196	2.043545
C	2.363070	-2.149826	2.376201
C	2.438444	-0.986574	1.623793
C	3.366845	0.341189	-0.311121
O	2.920101	0.119335	-1.663880
C	-4.115227	-0.389251	0.033305
C	-4.064535	0.021751	1.361784
C	-5.160288	0.646895	1.934207
C	-6.310417	0.863227	1.182905
C	-6.361925	0.453125	-0.140640
C	-5.263139	-0.173327	-0.715438
H	-0.932066	-1.007694	-2.218732
H	0.689951	0.522625	-0.140922
H	1.485851	-0.504012	-2.887308
H	-0.047552	-1.733627	0.632862
H	-3.131690	-1.336655	-1.635562
H	-1.340291	-3.801944	0.237334
H	-1.726011	-3.271113	-1.421291
H	2.056371	3.773537	-1.907412
H	3.814146	4.618786	-0.392118
H	3.545704	4.404574	2.059526
H	1.501793	3.364243	2.989649
H	-0.261127	2.530236	1.470418

H	-1.169436	2.273116	-0.712636
H	-0.501166	3.242588	-2.037840
H	4.807904	-1.938374	-0.606546
H	4.663002	-4.013059	0.725340
H	3.091629	-4.152266	2.631632
H	1.693042	-2.200844	3.226276
H	1.838796	-0.123841	1.901095
H	4.394080	0.682012	-0.429751
H	2.791415	1.150852	0.145238
H	-3.166869	-0.151312	1.944549
H	-5.120532	0.966536	2.969013
H	-7.166868	1.352092	1.632737
H	-7.256965	0.619570	-0.728404
H	-5.302743	-0.494962	-1.750833
C	1.744758	-1.950285	-1.488693
H	2.592092	-2.520399	-1.861105

ωB97X Energy = -1421.09135183 a.u.
Cremer-Pople parameters:
φ (°), θ (°), Q: 237.156, 43.693, 0.501

29cat, Conf B
Charge: +1

C	-1.216368	-0.203077	-0.757682
C	-0.116403	0.766066	-0.352599
C	1.212837	0.005620	-0.089466
O	0.309824	-2.052770	-0.949715
C	-0.934504	-1.622521	-0.275360
C	-3.501254	-0.669662	-0.653616
O	-2.457286	0.201170	-0.237304
C	-2.067671	-2.535860	-0.704485
O	-3.259343	-1.978060	-0.182773
O	0.009846	1.723293	-1.367559
C	1.771528	3.142562	-0.406152
C	2.018380	3.298678	0.952925
C	3.322533	3.322587	1.435494
C	4.388506	3.184983	0.558809
C	4.150093	3.036538	-0.803962
C	2.849327	3.020635	-1.282649
C	0.360346	3.043321	-0.928946
C	3.366417	-1.535442	1.043776
C	3.015109	-2.886349	1.070813
C	3.697690	-3.803598	0.285799
C	4.740803	-3.379151	-0.528434
C	5.106543	-2.039525	-0.549556
C	4.421822	-1.120956	0.234590
C	2.556319	-0.533616	1.817934
O	1.230720	-0.379143	1.269124
C	-4.797349	-0.174777	-0.086082
C	-5.830923	0.194553	-0.934678
C	-7.032986	0.652843	-0.409874
C	-7.199100	0.741104	0.963911
C	-6.163606	0.370816	1.815211
C	-4.965025	-0.086587	1.292479
H	-1.252647	-0.223004	-1.857863
H	-0.382587	1.220649	0.608521
H	2.060767	0.640105	-0.349637
H	-0.745399	-1.668487	0.796291
H	-3.526943	-0.681579	-1.753281
H	-1.964309	-3.536527	-0.291274
H	-2.105072	-2.595578	-1.799260
H	1.186286	3.403411	1.642268
H	3.503434	3.448699	2.496739
H	5.405647	3.202793	0.932944
H	4.981708	2.942398	-1.492849
H	2.663541	2.906711	-2.346068

H	-0.354036	3.368856	-0.165945
H	0.230186	3.666472	-1.813050
H	2.204117	-3.216717	1.712190
H	3.421005	-4.850819	0.312813
H	5.274389	-4.095666	-1.141680
H	5.925269	-1.709561	-1.177862
H	4.704190	-0.072269	0.215194
H	3.047790	0.444403	1.825417
H	2.384488	-0.855959	2.843635
H	-5.700044	0.124564	-2.009490
H	-7.838232	0.940095	-1.075764
H	-8.136269	1.098441	1.374958
H	-6.293546	0.439342	2.889029
H	-4.155322	-0.377006	1.952238
C	1.286011	-1.271670	-0.894051
H	2.157065	-1.584778	-1.467281

ωB97X Energy = -1421.08774140 a.u.
Cremer-Pople parameters:
φ (°), θ (°), Q: 286.681, 81.468, 0.683

29cat, Conf C
Charge: +1

C	-1.287434	-0.479979	-0.678705
C	-0.189187	0.515370	-0.355122
C	1.174799	-0.205819	-0.228878
O	0.202212	-2.389274	-0.572544
C	-1.034431	-1.818112	0.002591
C	-3.582167	-0.900626	-0.549415
O	-2.531182	0.007050	-0.243838
C	-2.181213	-2.770369	-0.287946
O	-3.371909	-2.124946	0.119039
O	-0.174590	1.492951	-1.358437
C	1.450866	3.071073	-0.388747
C	2.539270	2.988272	-1.257356
C	3.827769	3.202229	-0.793250
C	4.043337	3.513034	0.545986
C	2.966553	3.603713	1.414745
C	1.675645	3.378468	0.948071
C	0.051397	2.830981	-0.901071
C	3.802579	-1.174700	0.820967
C	3.789890	-2.518632	1.196211
C	4.588251	-3.437928	0.533993
C	5.410608	-3.021344	-0.507107
C	5.437479	-1.684807	-0.878268
C	4.635438	-0.763769	-0.215362
C	2.876829	-0.200232	1.492842
O	1.491019	-0.422069	1.137209
C	-4.879651	-0.303727	-0.094456
C	-5.851790	0.033692	-1.025054
C	-7.051076	0.596567	-0.606586
C	-7.276090	0.820909	0.743176
C	-6.302378	0.482348	1.676563
C	-5.106184	-0.078587	1.259787
H	-1.300553	-0.641191	-1.767932
H	-0.393895	0.947911	0.631135
H	1.942358	0.367013	-0.750684
H	-0.849332	-1.710135	1.072043
H	-3.585094	-1.077218	-1.635186
H	-2.095047	-3.692588	0.282123
H	-2.204836	-3.006191	-1.359340
H	2.371416	2.759090	-2.305178
H	4.666491	3.140380	-1.477400
H	5.050009	3.688570	0.907179
H	3.127898	3.849535	2.457901
H	0.835390	3.451990	1.631298

H	-0.681961	3.074569	-0.125616
H	-0.150138	3.454451	-1.771725
H	3.151621	-2.841934	2.012602
H	4.575904	-4.479522	0.832715
H	6.036529	-3.739893	-1.023173
H	6.084185	-1.356742	-1.683469
H	4.661209	0.283415	-0.503208
H	3.138443	0.831802	1.242996
H	2.886113	-0.318051	2.575139
H	-5.674815	-0.143386	-2.080789
H	-7.808280	0.858383	-1.336245
H	-8.211413	1.259506	1.071404
H	-6.478617	0.656764	2.731617
H	-4.345266	-0.346367	1.983914
C	1.180926	-1.625627	-0.720564
H	2.067066	-2.094260	-1.145826

ωB97X Energy = -1421.08731341 a.u.
Cremer-Pople parameters:
φ (°), θ (°), Q: 276.621, 74.831, 0.618

29cat, Conf D
Charge: +1

C	-0.453371	-1.625695	-0.559941
C	0.842367	-0.842710	-0.467840
C	2.026045	-1.826397	-0.363922
O	0.590375	-3.723456	0.122464
C	-0.476220	-2.757827	0.454785
C	-2.780093	-1.475817	-0.364439
O	-1.542279	-0.785880	-0.286220
C	-1.813298	-3.477438	0.394350
O	-2.821792	-2.506760	0.597704
O	0.963665	-0.032348	-1.604433
C	0.316970	2.117402	-0.627841
C	0.552856	2.613464	0.648727
C	-0.436149	3.317765	1.327225
C	-1.670858	3.523562	0.731657
C	-1.914231	3.031142	-0.546704
C	-0.924749	2.336449	-1.221672
C	1.370672	1.323134	-1.356257
C	4.416021	-0.197947	0.527869
C	4.971038	0.068771	-0.719689
C	5.259059	1.375067	-1.096478
C	4.994955	2.420924	-0.224895
C	4.446329	2.161377	1.026609
C	4.158853	0.858648	1.400681
C	4.078648	-1.602617	0.936163
O	2.645480	-1.834350	0.917195
C	-3.874685	-0.485983	-0.098724
C	-4.023014	0.064859	1.169997
C	-5.001509	1.018534	1.399622
C	-5.833294	1.427586	0.362980
C	-5.686441	0.878173	-0.902045
C	-4.706469	-0.079478	-1.132234
H	-0.539757	-2.052758	-1.572179
H	0.821664	-0.252970	0.455401
H	2.729062	-1.634803	-1.176360
H	-0.255916	-2.407129	1.463740
H	-2.874210	-1.916147	-1.368473
H	-1.906833	-4.220075	1.183316
H	-1.926073	-3.966991	-0.580915
H	1.519053	2.449264	1.117099
H	-0.240850	3.700527	2.322476
H	-2.446151	4.066808	1.260098
H	-2.880963	3.187801	-1.012971
H	-1.117906	1.945174	-2.215250

H	1.552011	1.734200	-2.349236
H	2.315888	1.337697	-0.803518
H	5.192364	-0.748787	-1.399089
H	5.693421	1.573087	-2.069205
H	5.222130	3.439922	-0.515504
H	4.249217	2.977518	1.712108
H	3.734953	0.654428	2.378669
H	4.356212	-1.800368	1.969835
H	4.576343	-2.332246	0.292750
H	-3.369777	-0.254425	1.973888
H	-5.116758	1.446682	2.388667
H	-6.597914	2.174226	0.544502
H	-6.334945	1.192599	-1.711393
H	-4.589972	-0.509180	-2.121825
C	1.680774	-3.278942	-0.302365
H	2.430871	-4.037749	-0.514017

ωB97X Energy = -1421.08591864 a.u.
Cremer-Pople parameters:
φ (°), θ (°), Q: 264.433, 67.881, 0.564

29cat, Conf E
Charge: +1

C	-1.161968	-1.406206	-0.383433
C	0.188445	-1.229550	0.291186
C	1.086699	-2.396543	-0.134071
O	-0.862150	-3.841982	-0.490536
C	-1.754080	-2.758613	-0.018833
C	-3.337276	-0.581473	-0.599147
O	-2.073754	-0.423331	0.039025
C	-3.113860	-2.922367	-0.674951
O	-3.901798	-1.826027	-0.251203
O	0.834729	-0.053874	-0.104129
C	1.376040	2.252413	0.203195
C	1.972181	3.168988	1.062371
C	2.622074	4.287873	0.560302
C	2.691416	4.494654	-0.810803
C	2.099948	3.581260	-1.673650
C	1.441282	2.468457	-1.169278
C	0.650349	1.062194	0.768114
C	3.914810	-1.551110	0.366597
C	4.378065	-1.190702	-0.892552
C	4.883772	0.085103	-1.117678
C	4.926026	1.004751	-0.082270
C	4.470282	0.647797	1.182848
C	3.969736	-0.622972	1.405928
C	3.330956	-2.912581	0.613202
O	1.916043	-2.845845	0.908103
C	-4.245026	0.521677	-0.146665
C	-4.639790	0.599387	1.185295
C	-5.469643	1.627983	1.600912
C	-5.907594	2.582111	0.688934
C	-5.514538	2.505028	-0.638601
C	-4.682610	1.473803	-1.056117
H	-1.029751	-1.349310	-1.475357
H	0.058853	-1.269880	1.379550
H	1.657785	-2.065330	-1.012722
H	-1.818181	-2.895425	1.060970
H	-3.181423	-0.546906	-1.687266
H	-3.612462	-3.832315	-0.348797
H	-3.006379	-2.935999	-1.766611
H	1.936702	3.002779	2.134624
H	3.083546	4.994126	1.241166
H	3.205018	5.363842	-1.205594
H	2.150374	3.736741	-2.745550
H	0.984608	1.754462	-1.844574

H	1.049047	0.817584	1.759563
H	-0.418930	1.276046	0.867113
H	4.348875	-1.911088	-1.704437
H	5.241489	0.358005	-2.103732
H	5.309849	2.003623	-0.257003
H	4.505818	1.366743	1.993247
H	3.612968	-0.902029	2.392404
H	3.755410	-3.379625	1.500541
H	3.500396	-3.574716	-0.241057
H	-4.298132	-0.147986	1.892306
H	-5.777730	1.687741	2.638246
H	-6.557080	3.385831	1.016173
H	-5.854903	3.246406	-1.351905
H	-4.374880	1.411877	-2.094683
C	0.369580	-3.649830	-0.531846
H	0.952897	-4.515914	-0.840621

ωB97X Energy = -1421.08524056 a.u.
Cremer-Pople parameters:
φ (°), θ (°), Q: 244.187, 54.596, 0.521

29cat, Conf F
Charge: +1

C	-1.162666	-1.288646	-0.771047
C	0.281810	-1.185752	-0.309489
C	1.056996	-2.421280	-0.798667
O	-0.989312	-3.693042	-1.176234
C	-1.730250	-2.656589	-0.421895
C	-3.316250	-0.400612	-0.529348
O	-1.952668	-0.318826	-0.129958
C	-3.194325	-2.729967	-0.818167
O	-3.848773	-1.656288	-0.170998
O	0.912602	-0.044299	-0.820075
C	1.679845	2.216011	-0.556030
C	2.015070	2.236733	-1.903157
C	2.700273	3.322365	-2.438155
C	3.051097	4.395419	-1.633426
C	2.714014	4.379657	-0.284550
C	2.035835	3.295736	0.249177
C	0.933467	1.068992	0.070011
C	3.489680	-1.703816	0.931795
C	3.213041	-1.417566	2.268047
C	3.559392	-0.188746	2.807415
C	4.189626	0.765220	2.015374
C	4.467477	0.487721	0.685490
C	4.117142	-0.743588	0.146081
C	3.099839	-3.036814	0.356506
O	1.664154	-3.172531	0.231069
C	-4.085169	0.680148	0.168672
C	-4.694326	1.685885	-0.567659
C	-5.402652	2.690805	0.079451
C	-5.500202	2.688031	1.462618
C	-4.889837	1.680147	2.201272
C	-4.183539	0.677813	1.556757
H	-1.202785	-1.151271	-1.863577
H	0.302582	-1.197966	0.786774
H	1.772975	-2.105214	-1.565040
H	-1.588803	-2.901265	0.630927
H	-3.365758	-0.285089	-1.621982
H	-3.658634	-3.652898	-0.478360
H	-3.290480	-2.653473	-1.907950
H	1.745031	1.399143	-2.534122
H	2.959412	3.326612	-3.491056
H	3.585196	5.240720	-2.051925
H	2.985751	5.212917	0.353581
H	1.784634	3.283525	1.305746

H	1.431018	0.774960	1.002788
H	-0.091813	1.371222	0.307713
H	2.728970	-2.165577	2.887964
H	3.346587	0.024228	3.848603
H	4.462600	1.725856	2.436971
H	4.951125	1.232372	0.063704
H	4.337301	-0.958256	-0.894818
H	3.381694	-3.854052	1.018266
H	3.563763	-3.198826	-0.619759
H	-4.618335	1.686851	-1.649985
H	-5.877888	3.474485	-0.498711
H	-6.052848	3.471163	1.968762
H	-4.966671	1.677380	3.282340
H	-3.707872	-0.111040	2.128286
C	0.233631	-3.537904	-1.367507
H	0.721120	-4.343036	-1.912971

ωB97X Energy = -1421.08411602 a.u.
Cremer-Pople parameters:
φ (°), θ (°), Q: 258.408, 63.632, 0.552

31cat, Conf A
Charge: +1

C	-2.395747	-1.742819	-0.242827
C	-1.416553	-0.549949	-0.236956
C	-0.145614	-0.836062	0.583347
O	-0.738524	-3.178522	0.809124
C	-1.669734	-3.080350	-0.277182
C	-0.742119	-3.247731	-1.477981
C	1.020902	1.432850	2.059489
C	1.064145	2.462702	1.127118
C	2.265134	3.097045	0.831602
C	3.432378	2.698802	1.465229
C	3.398370	1.663485	2.393349
C	2.199096	1.034955	2.689084
C	-0.282066	0.746901	2.377969
O	-0.320835	-0.623336	1.960647
O	-3.192624	-1.769699	0.941405
O	-4.474811	-0.132722	0.104453
C	-4.220976	-0.897140	1.002032
C	-4.958793	-1.014291	2.297020
O	0.471132	-2.563979	-0.990028
C	2.844083	-2.073649	-1.107356
C	2.806174	-0.796807	-1.667504
C	3.767570	0.137422	-1.323539
C	4.780127	-0.203126	-0.432895
C	4.826876	-1.474365	0.117755
C	3.853590	-2.408514	-0.212470
C	1.794696	-3.073576	-1.461467
O	-1.027196	-0.259639	-1.564219
C	-1.662951	2.087462	-1.800723
C	-0.551319	2.791348	-2.260511
C	-0.323593	4.095653	-1.849821
C	-1.205085	4.709901	-0.967046
C	-2.313982	4.016754	-0.503024
C	-2.543732	2.710941	-0.921652
C	-1.888928	0.665504	-2.244977
H	-3.043403	-1.662450	-1.118347
H	-1.918657	0.309525	0.213748
H	0.667315	-0.224952	0.176400
H	-2.371860	-3.905123	-0.188791
H	-0.492271	-4.292763	-1.648155
H	-1.050248	-2.736063	-2.384427
H	0.151814	2.774222	0.625887
H	2.283624	3.901195	0.104025
H	4.370686	3.192171	1.237740

H	4.309604	1.350737	2.890567
H	2.171864	0.231215	3.418256
H	-1.113852	1.303270	1.932060
H	-0.447123	0.701673	3.454090
H	-5.819402	-0.351337	2.287700
H	-5.277001	-2.045162	2.451485
H	-4.291260	-0.745860	3.117035
H	2.013837	-0.534864	-2.361764
H	3.730647	1.133123	-1.749274
H	5.532675	0.529781	-0.166466
H	5.615986	-1.740067	0.810835
H	3.885264	-3.401376	0.224026
H	1.648884	-3.179760	-2.534519
H	1.947490	-4.046601	-0.994424
H	0.134682	2.313138	-2.952961
H	0.538860	4.637854	-2.220962
H	-1.029197	5.730391	-0.646660
H	-3.006758	4.494518	0.180110
H	-3.420074	2.172644	-0.571210
H	-2.935783	0.382227	-2.097429
H	-1.643318	0.550673	-3.300489
C	0.258600	-2.300547	0.492977
H	1.176562	-2.535588	1.026614

ωB97X Energy = -1574.99318425 a.u.
Cremer-Pople parameters:
φ (°), θ (°), Q: 171.753, 157.601, 0.559

31cat, Conf B
Charge: +1

C	-0.717835	-2.689097	0.808927
C	-0.381151	-1.871460	-0.447262
C	-1.263285	-0.627561	-0.497665
O	-2.265871	-1.049942	1.695682
C	-1.148657	-1.897456	2.031710
C	-0.048916	-1.094879	2.711950
C	-2.189616	2.019540	-1.476157
C	-1.584732	2.839399	-0.529890
C	-2.340123	3.746940	0.203129
C	-3.708395	3.836894	-0.004189
C	-4.321162	3.020645	-0.949336
C	-3.565003	2.118775	-1.681730
C	-1.378821	1.004130	-2.238882
O	-1.633746	-0.345374	-1.819296
O	-1.817669	-3.617762	0.467119
O	-3.288062	-2.023796	-0.188113
C	-2.930643	-3.230988	-0.042609
C	-3.845099	-4.291378	-0.514300
O	0.252809	0.048875	1.958687
C	1.707086	1.859219	1.457693
C	2.275728	1.758530	0.190623
C	2.430842	2.888005	-0.601469
C	2.019526	4.129372	-0.131625
C	1.453034	4.237374	1.131787
C	1.297111	3.105513	1.921158
C	1.509204	0.628295	2.295867
O	0.959039	-1.475760	-0.467301
C	3.234388	-1.827785	-0.958657
C	3.728436	-1.108562	-2.041585
C	4.956033	-0.465491	-1.956222
C	5.696479	-0.536345	-0.783889
C	5.211216	-1.257283	0.300074
C	3.985479	-1.900992	0.210460
C	1.865891	-2.439711	-1.013635
H	0.095473	-3.362685	1.066712
H	-0.612083	-2.489613	-1.324729
H	-0.705022	0.188498	-0.032730
H	-1.528181	-2.611381	2.765458
H	-0.395613	-0.814226	3.713620
H	0.829717	-1.743850	2.830454
H	-0.513234	2.772836	-0.362768
H	-1.854250	4.381171	0.936450
H	-4.299865	4.544068	0.565974
H	-5.389466	3.093693	-1.118105
H	-4.043036	1.484289	-2.421771
H	-0.309627	1.217324	-2.134982
H	-1.635088	1.013349	-3.297853
H	-3.609428	-4.481923	-1.564648
H	-4.875425	-3.954218	-0.442396
H	-3.680030	-5.205126	0.050639
H	2.597134	0.787596	-0.174283
H	2.879242	2.799739	-1.584900
H	2.144063	5.012378	-0.748010
H	1.135197	5.204751	1.503988
H	0.856231	3.191160	2.909397
H	2.305283	-0.100843	2.098829
H	1.522369	0.880644	3.363130
H	3.146181	-1.047624	-2.955547
H	5.334705	0.091869	-2.805217
H	6.654115	-0.033289	-0.715692
H	5.789920	-1.318703	1.214465
H	3.604431	-2.463135	1.058033
H	1.574445	-2.667621	-2.044506
H	1.833796	-3.366528	-0.429359
C	-2.493092	-0.829353	0.379565
H	-3.227494	-0.038264	0.263049

ωB97X Energy = -1574.98904211 a.u.
Cremer-Pople parameters:
φ (°), θ (°), Q: 82.920, 89.090, 0.662

31cat, Conf C
Charge: +1

C	0.058082	-1.908514	-1.375664
C	-1.169951	-1.031473	-1.090046
C	-1.728927	-1.360405	0.302917
O	0.208531	-2.688531	0.924902
C	0.956470	-2.148063	-0.182275
C	1.677803	-0.888532	0.274083
C	-3.613882	0.628280	1.458690
C	-4.104594	0.232141	2.701657
C	-3.898228	1.020999	3.822216
C	-3.200415	2.218748	3.710588
C	-2.708689	2.619904	2.477473
C	-2.912385	1.824445	1.355997
C	-3.835171	-0.246655	0.251808
O	-3.121294	-1.490420	0.329151
O	-0.405258	-3.210031	-1.898318
O	-1.608090	-3.801386	-0.071754
C	-1.191764	-3.990924	-1.256535
C	-1.658189	-5.203481	-1.959406
O	2.437813	-0.433911	-0.819831
C	4.344283	0.416869	0.458632
C	4.430714	1.089904	1.670968
C	5.464722	0.815499	2.558906
C	6.415350	-0.142445	2.240693
C	6.332614	-0.824081	1.031188
C	5.304669	-0.543127	0.145325
C	3.229306	0.711297	-0.514927
O	-0.845264	0.331396	-1.122748
C	-0.550433	2.374266	-2.293207
C	-1.649173	3.215979	-2.146788

C	-1.470272	4.579753	-1.965696
C	-0.187776	5.112877	-1.932802
C	0.912677	4.280445	-2.085389
C	0.729687	2.916226	-2.265428
C	-0.744563	0.894006	-2.438105
H	0.650011	-1.510418	-2.194330
H	-1.937538	-1.265859	-1.840374
H	-1.385944	-0.584270	0.999496
H	1.698703	-2.903027	-0.446698
H	0.964396	-0.120522	0.595804
H	2.311763	-1.155274	1.126648
H	-4.654826	-0.699585	2.787358
H	-4.287335	0.707243	4.784095
H	-3.042565	2.837935	4.586233
H	-2.162281	3.551653	2.385736
H	-2.514384	2.132631	0.393703
H	-3.558594	0.288415	-0.662463
H	-4.880495	-0.542449	0.171889
H	-1.482950	-6.071559	-1.324880
H	-2.735516	-5.111257	-2.110539
H	-1.153424	-5.307436	-2.914655
H	3.683648	1.835632	1.924641
H	5.523119	1.348357	3.501077
H	7.221455	-0.359447	2.932162
H	7.076100	-1.571281	0.777857
H	5.242276	-1.072264	-0.800194
H	2.587790	1.511280	-0.124506
H	3.636604	1.036143	-1.473683
H	-2.652106	2.801184	-2.181999
H	-2.331674	5.228318	-1.855556
H	-0.046948	6.178703	-1.794719
H	1.914364	4.694214	-2.067860
H	1.588614	2.264368	-2.390836
H	-1.664266	0.671229	-2.990650
H	0.101322	0.451670	-2.974177
C	-1.141873	-2.658175	0.825010
H	-1.581362	-2.955842	1.770922

ωB97X Energy = -1574.98760781 a.u.
Cremer-Pople parameters:
φ (°), θ (°), Q: 76.123, 89.911, 0.662

31cat, Conf D
Charge: +1

C	-0.736932	-2.309031	-1.091613
C	-1.321681	-0.909025	-1.253093
C	-2.153961	-0.536034	-0.010108
O	-1.224916	-2.389681	1.285771
C	-0.189117	-2.582702	0.293810
C	1.025219	-1.732377	0.617183
C	-3.337330	2.088899	0.875244
C	-3.293482	3.134942	-0.045607
C	-2.612414	4.304995	0.250907
C	-1.967713	4.443628	1.475338
C	-2.006540	3.409315	2.398250
C	-2.689182	2.236139	2.097019
C	-4.067903	0.815973	0.534380
O	-3.370635	0.012767	-0.433206
O	-1.758347	-3.315828	-1.428542
O	-3.264691	-2.741476	0.159501
C	-2.895016	-3.418981	-0.847058
C	-3.849379	-4.391450	-1.415637
O	1.631794	-2.275760	1.763399
C	3.792713	-1.182225	1.387601
C	3.922062	0.010836	0.683330
C	4.982369	0.201999	-0.193994
C	5.922065	-0.802501	-0.375709
C	5.799500	-1.998590	0.321899
C	4.740612	-2.185360	1.197627
C	2.625232	-1.414003	2.315984
O	-0.203030	-0.085126	-1.430285
C	0.748963	2.071003	-1.600396
C	0.896024	2.748037	-0.393180
C	2.059439	3.453685	-0.119443
C	3.087247	3.484872	-1.053309
C	2.946448	2.812726	-2.260560
C	1.781236	2.108841	-2.532010
C	-0.484711	1.255356	-1.856584
H	0.027249	-2.475727	-1.844343
H	-1.982491	-0.888216	-2.128835
H	-1.574883	0.175812	0.592323
H	0.095459	-3.633526	0.369007
H	1.710728	-1.739200	-0.238718
H	0.728653	-0.691681	0.790410
H	-3.802412	3.030840	-0.998829
H	-2.588129	5.114205	-0.469670
H	-1.438734	5.360478	1.708653
H	-1.509497	3.514284	3.355612
H	-2.726681	1.432072	2.825959
H	-5.023626	1.033045	0.059683
H	-4.267854	0.235488	1.440259
H	-4.513755	-3.840789	-2.086952
H	-3.319622	-5.149953	-1.984968
H	-4.448220	-4.828567	-0.620540
H	3.187360	0.801377	0.815431
H	5.067706	1.136845	-0.737097
H	6.751293	-0.654751	-1.058198
H	6.534319	-2.783961	0.185864
H	4.647519	-3.118535	1.744487
H	2.952796	-1.908482	3.230350
H	2.164841	-0.455144	2.585019
H	0.088956	2.731899	0.334511
H	2.162469	3.983285	0.820932
H	3.995627	4.037909	-0.842497
H	3.744990	2.839086	-2.993268
H	1.671818	1.584496	-3.475933
H	-1.329529	1.661198	-1.292383
H	-0.748966	1.240200	-2.919178
C	-2.353611	-1.757496	0.888793
H	-2.959730	-1.551982	1.765434

ωB97X Energy = -1574.98695973 a.u.
Cremer-Pople parameters:
φ (°), θ (°), Q: 74.810, 92.221, 0.671

31cat, Conf E
Charge: +1

C	-2.478695	1.455296	-0.848625
C	-1.755192	0.931697	0.406306
C	-1.815597	-0.597103	0.426213
O	-2.890643	-0.762695	-1.755778
C	-2.465657	0.575440	-2.087562
C	-1.187648	0.533583	-2.896298
C	0.016609	-2.421024	1.949772
C	1.260919	-2.146025	1.397003
C	2.038558	-3.167833	0.864186
C	1.572800	-4.473585	0.885169
C	0.328052	-4.757723	1.438129
C	-0.445063	-3.736523	1.966387
C	-0.845761	-1.313564	2.494393
O	-2.032070	-1.112395	1.711332
O	-3.901303	1.652706	-0.481754

O	-4.274063	-0.497335	0.130027
C	-4.625676	0.715317	0.012715
C	-5.963478	1.102446	0.505540
O	-0.124094	-0.021982	-2.169420
C	2.227053	-0.566821	-2.205489
C	3.123962	-1.535512	-2.643533
C	4.316869	-1.750804	-1.965345
C	4.617708	-1.003086	-0.835342
C	3.717107	-0.045596	-0.384143
C	2.528883	0.173738	-1.066594
C	0.994406	-0.267718	-3.012426
O	-0.429565	1.366837	0.489623
C	1.185240	2.960771	1.206654
C	1.939308	2.418842	2.244102
C	3.299915	2.673617	2.331514
C	3.919009	3.473643	1.378808
C	3.174052	4.016714	0.341395
C	1.811761	3.760761	0.257858
C	-0.280147	2.654592	1.102458
H	-2.148600	2.459318	-1.103345
H	-2.319236	1.292812	1.277270
H	-0.904907	-0.990790	-0.040924
H	-3.230054	0.970954	-2.761369
H	-1.401522	-0.067742	-3.789048
H	-0.952914	1.554172	-3.228920
H	1.623784	-1.122834	1.370482
H	3.002363	-2.934548	0.424805
H	2.176570	-5.272894	0.470630
H	-0.036074	-5.778639	1.459056
H	-1.416612	-3.957563	2.397230
H	-0.276548	-0.381214	2.558578
H	-1.218375	-1.560969	3.488037
H	-6.641444	0.255521	0.448804
H	-5.852206	1.396016	1.552788
H	-6.335674	1.955995	-0.055335
H	2.891542	-2.124460	-3.525293
H	5.009501	-2.506124	-2.319070
H	5.548406	-1.169004	-0.304551
H	3.940322	0.540136	0.501544
H	1.820735	0.912280	-0.706444
H	1.177107	0.622395	-3.630152
H	0.770468	-1.099722	-3.691033
H	1.455868	1.797887	2.992107
H	3.878001	2.251765	3.145709
H	4.981829	3.675230	1.447396
H	3.653155	4.642762	-0.402368
H	1.229282	4.188351	-0.552040
H	-0.743117	2.629790	2.095845
H	-0.787060	3.420719	0.504989
C	-2.963605	-1.074362	-0.444586
H	-3.154829	-2.135751	-0.329580

ωB97X Energy = -1574.98670654 a.u.
Cremer-Pople parameters:
φ (°), θ (°), Q: 81.782, 86.759, 0.645

31cat, Conf F
Charge: +1

C	-0.458035	0.628917	-0.810738
C	-0.118196	-0.830304	-1.111831
C	1.244184	-1.167646	-0.486577
O	0.559748	0.256012	1.372534
C	-0.475715	0.960428	0.668854
C	-1.800221	0.596031	1.354510
C	4.186713	-1.879857	-0.215611
C	4.208382	-2.678208	0.922562
C	5.047331	-2.367018	1.986318
C	5.869799	-1.252620	1.916195
C	5.858807	-0.452824	0.778296
C	5.024044	-0.767515	-0.281930
C	3.247179	-2.185645	-1.352673
O	2.204183	-1.211742	-1.510314
O	0.549256	1.461836	-1.508474
O	2.242323	1.057470	-0.061472
C	1.743622	1.629352	-1.084687
C	2.606027	2.559176	-1.837769
O	-2.924180	1.049740	0.634962
C	-2.492130	3.462198	0.596729
C	-2.279826	3.732340	-0.754337
C	-1.376301	4.708943	-1.143862
C	-0.680236	5.435172	-0.183166
C	-0.898637	5.185054	1.163830
C	-1.801498	4.201205	1.550497
C	-3.415713	2.340938	1.003085
O	-1.085489	-1.688370	-0.571358
C	-3.226951	-2.716026	-0.632348
C	-3.163871	-4.086551	-0.862752
C	-4.005007	-4.952384	-0.178422
C	-4.915901	-4.451262	0.742594
C	-4.984304	-3.084146	0.976603
C	-4.142416	-2.219350	0.290930
C	-2.293726	-1.780472	-1.342592
H	-1.388821	0.949823	-1.268323
H	-0.027616	-0.960843	-2.195772
H	1.146041	-2.131676	0.023293
H	-0.285891	2.032079	0.782056
H	-1.866582	-0.489939	1.416261
H	-1.783183	1.004140	2.370585
H	3.564871	-3.550682	0.980041
H	5.057010	-2.996252	2.868647
H	6.524313	-1.007662	2.744651
H	6.507422	0.413537	0.718133
H	5.016612	-0.144731	-1.171196
H	2.794629	-3.173247	-1.219948
H	3.773461	-2.172286	-2.306571
H	3.171900	1.958111	-2.555349
H	2.005106	3.287217	-2.375440
H	3.310089	3.036809	-1.160520
H	-2.829173	3.171990	-1.505092
H	-1.220183	4.912672	-2.197128
H	0.022997	6.202290	-0.486437
H	-0.366030	5.755036	1.916337
H	-1.965273	4.002938	2.605119
H	-3.594158	2.367996	2.083202
H	-4.375630	2.428687	0.493721
H	-2.453696	-4.477194	-1.584773
H	-3.952775	-6.018718	-0.365976
H	-5.575465	-5.126734	1.275384
H	-5.697113	-2.690815	1.692361
H	-4.192126	-1.148255	0.468514
H	-2.045592	-2.150286	-2.343403
H	-2.757384	-0.793058	-1.434183
C	1.609925	-0.137104	0.600773
H	2.411563	-0.475453	1.249885

ωB97X Energy = -1574.98556716 a.u.
Cremer-Pople parameters:
φ (°), θ (°), Q: 39.840, 91.059, 0.685

SPh anion
Charge: -1

C	-1.550660	-1.194653	0.000015

C	-0.163191	-1.193922	-0.000003
C	0.576428	-0.000022	-0.000087
C	-0.163189	1.193919	-0.000010
C	-1.550626	1.194675	0.000021
C	-2.261025	0.000000	-0.000004
H	-2.082230	-2.141411	0.000035
H	0.374644	-2.136246	0.000011
H	0.374696	2.136216	0.000005
H	-2.082218	2.141420	0.000043
H	-3.345354	0.000029	0.000004
S	2.339627	0.000001	0.000020

ωB97X Energy = -629.974281783 a.u.

References

[1] Schrödinger, LLC. MacroModel; Schrödinger, LLC: New York, **2015**; http://www.schrodinger.com/MacroModel.

[2] Kaminski, G. A.; Friesner, R. A.; Tirado-Rives, J.; Jorgensen, W. L., Evaluation and Reparametrization of the OPLS-AA Force Field for Proteins via Comparison with Accurate Quantum Chemical Calculations on Peptides, *J. Phys. Chem. B* **2001**, *105*, (28), 6474-6487.

[3] Frisch, M. J.; Trucks, G. W.; Schlegel, H. B.; Scuseria, G. E.; Robb, M. A.; Cheeseman, J. R.; Scalmani, G.; Barone, V.; Petersson, G. A.; Nakatsuji, H. *et* al., Gaussian 16, Revision C.02, Gaussian, Inc., Wallingford CT, USA, **2019**.

[4] Varetto, U., MOLEKEL 5.4; Swiss National Supercomputing Centre: Manno, Switzerland, **2009**.

[5] Cremer, D.; Pople, J. A., General definition of ring puckering coordinates, *J. Am. Chem. Soc.* **1975**, *97*, (6), 1354-1358.

[6] Fushinobu, S., **2006**, Cremer–Pople Parameter Calculator, http://enzyme13.bt.a.u-tokyo.ac.jp/CP/.

NMR spectra of the synthesized compounds

^{1}H, ^{13}C, COSY, HMBC and HSQC NMR spectra of compound **4**

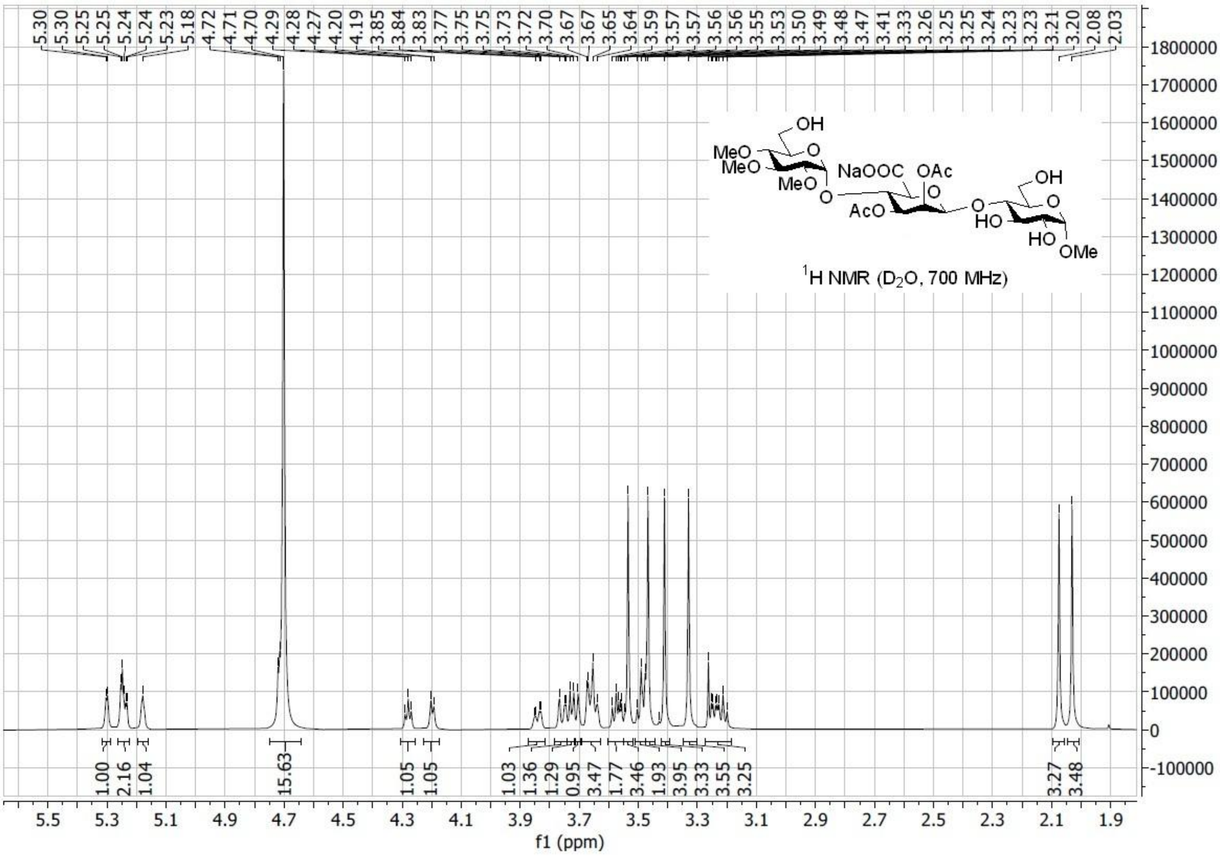

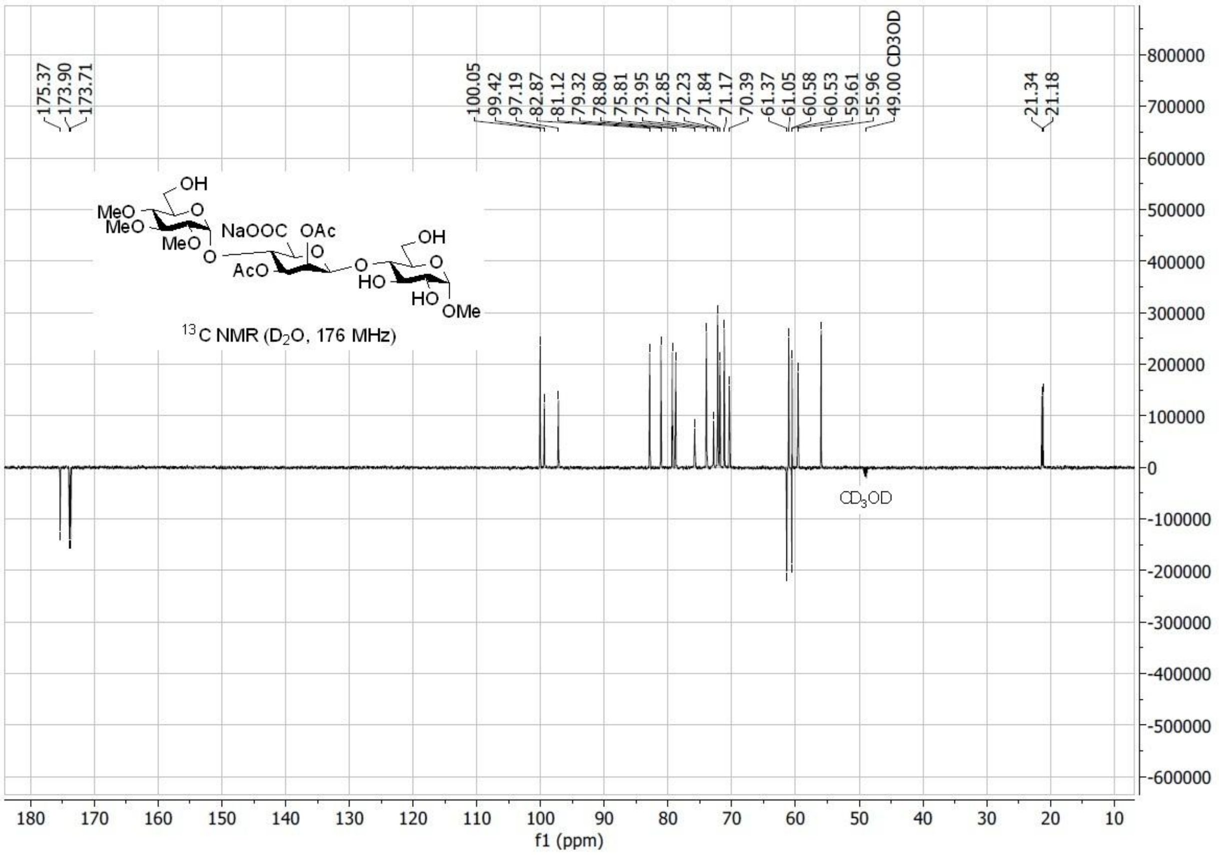

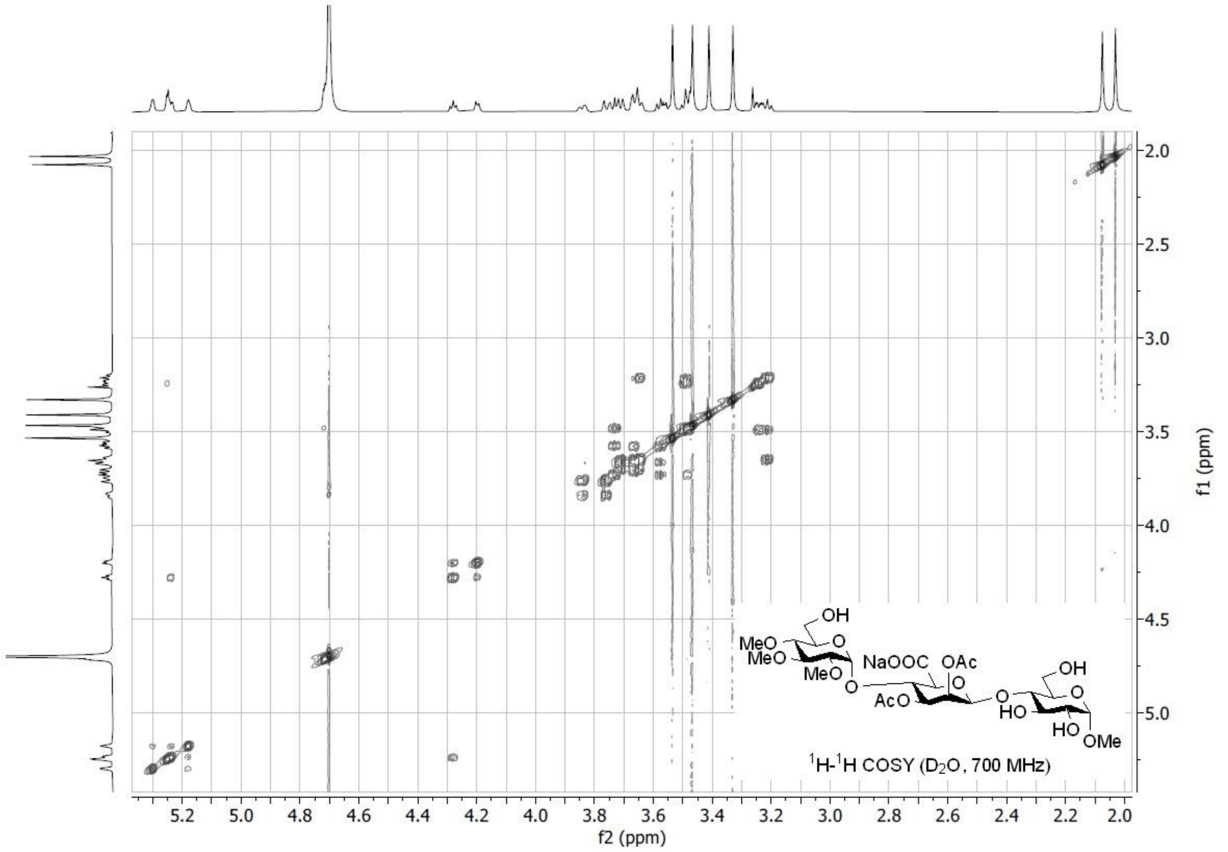
MeO
MeO
MeO
OH
O
O
NaOOC
OAc
AcO
O
O
OH
HO
HO
OMe
^{1}H-^{1}H COSY (D_2O, 700 MHz)
f2 (ppm)
f1 (ppm)

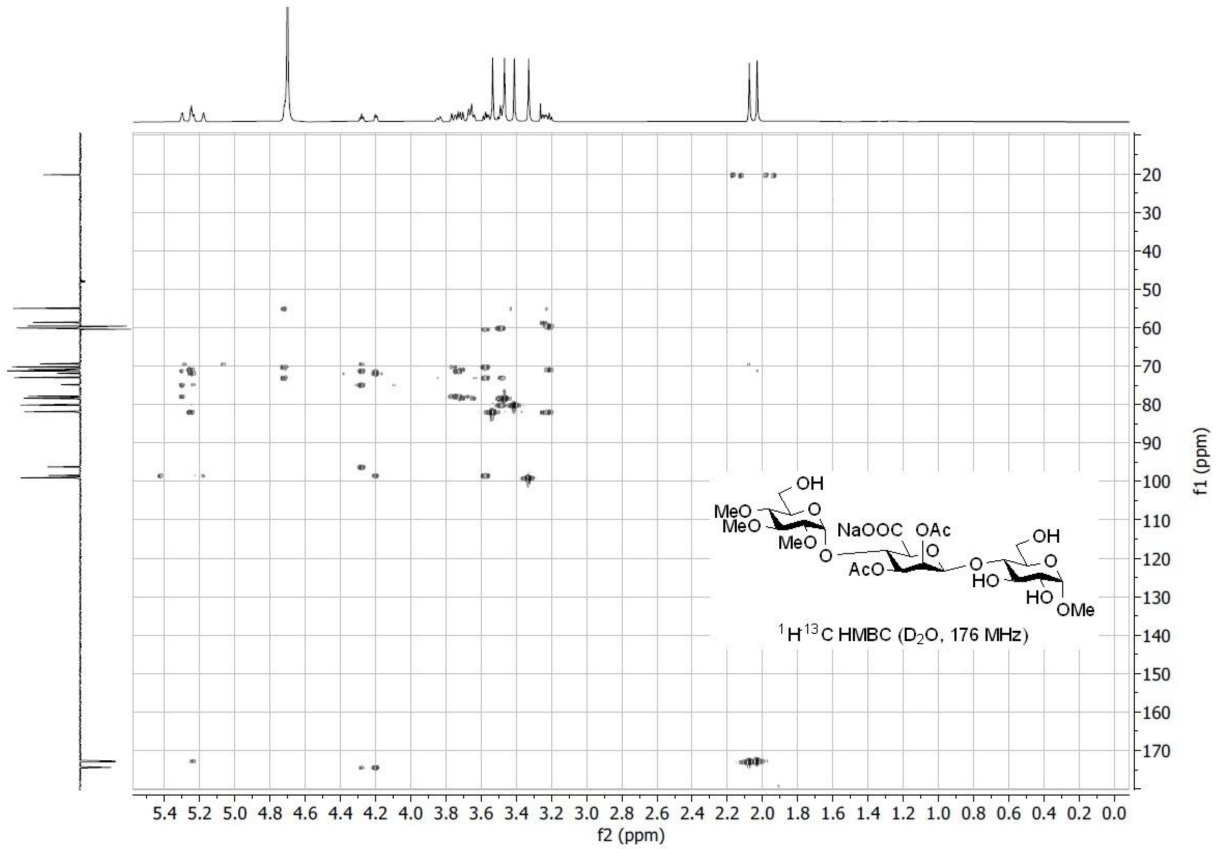
MeO
MeO
MeO
OH
O
O
NaOOC
OAc
AcO
O
O
OH
HO
HO
OMe
^{1}H-^{13}C HMBC (D_2O, 176 MHz)
f2 (ppm)
f1 (ppm)

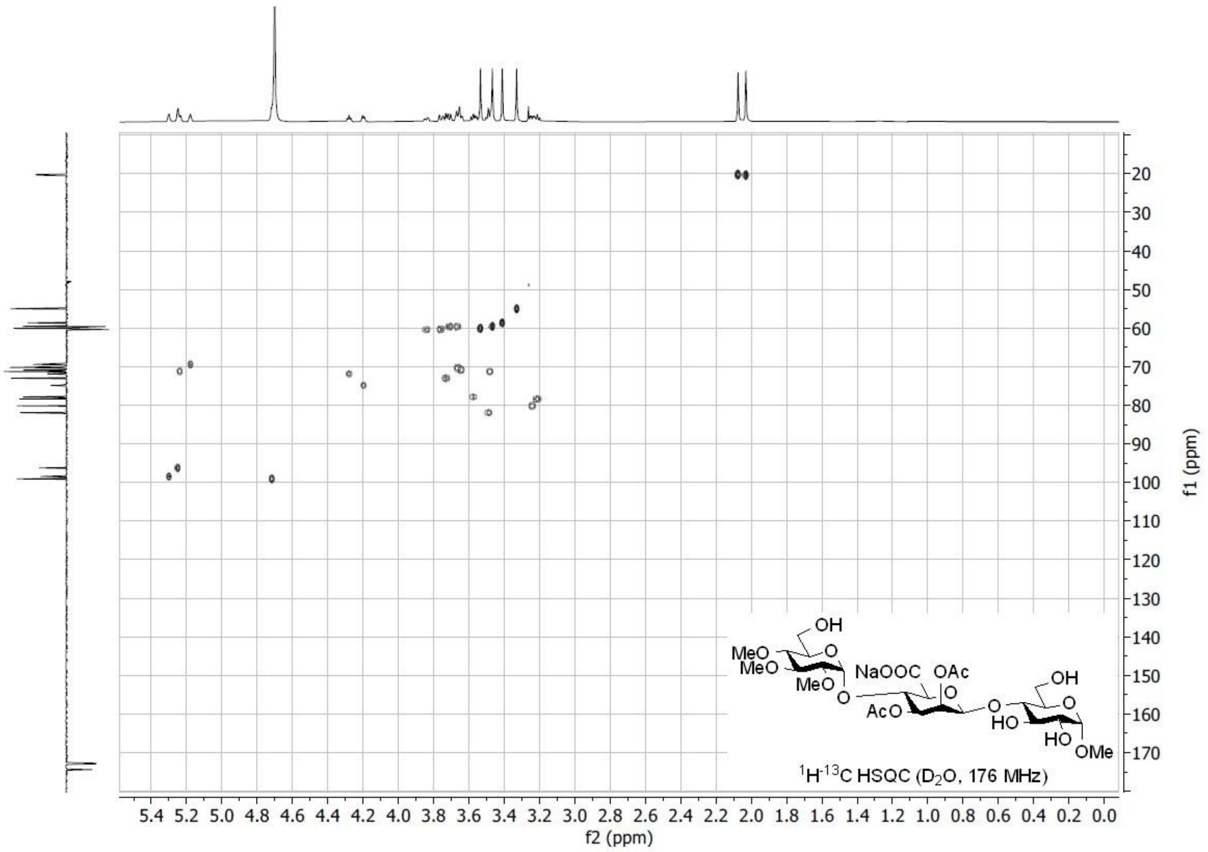

f2 (ppm)
f1 (ppm)
MeO
MeO
OH
MeO
NaOOC
OAc
AcO
OH
HO
HO
OMe
^{1}H-^{13}C HSQC (D_2O, 176 MHz)

^{1}H, ^{13}C, COSY and HSQC NMR spectra of compound **5**

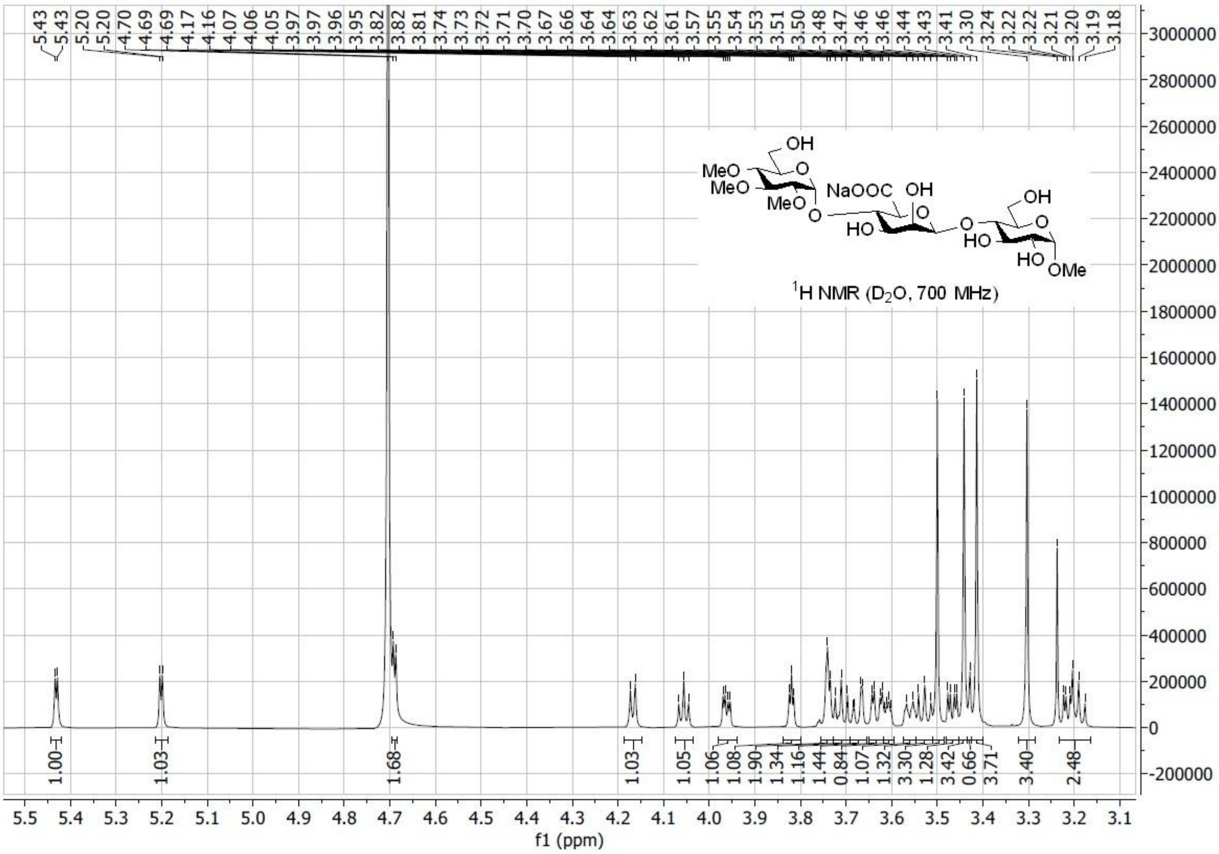

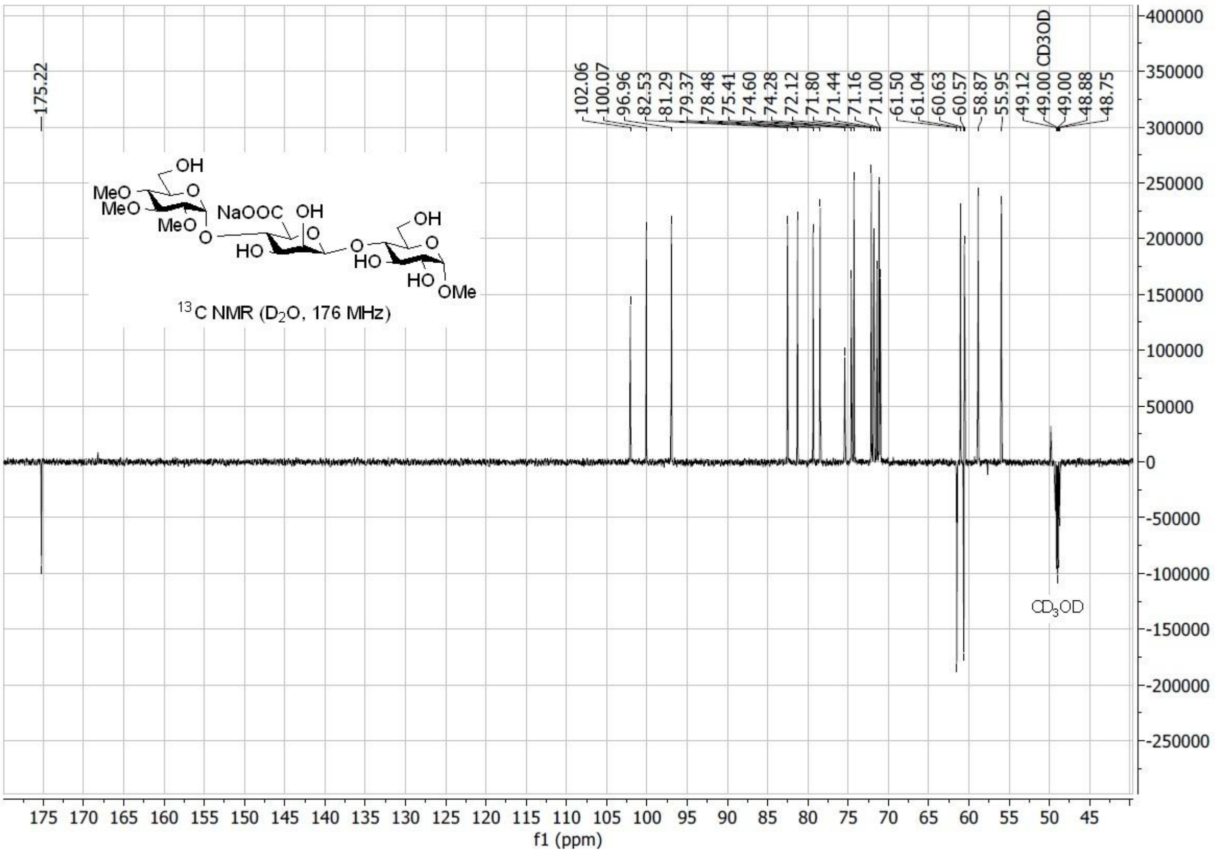

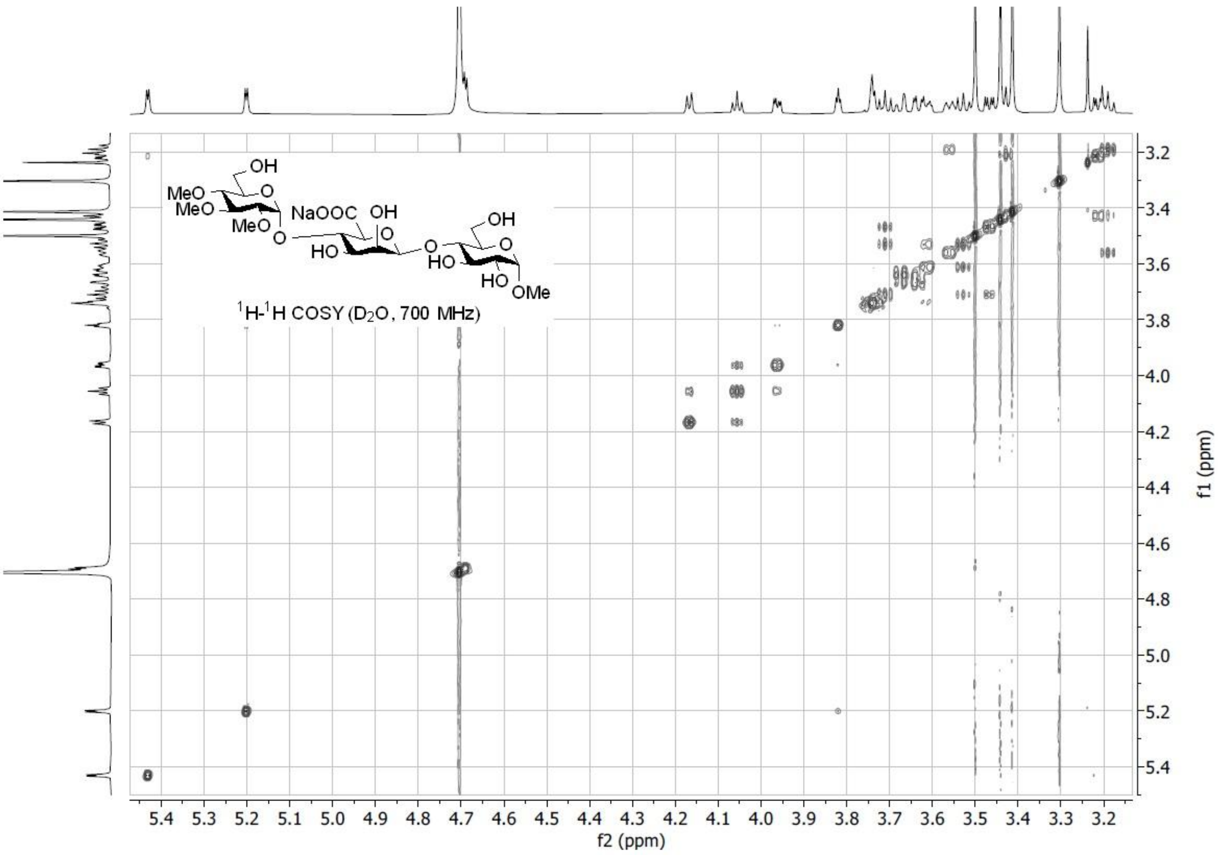

OH
MeO
MeO
O
MeO
NaOOC OH
O
HO
O
O
OH
O
HO
HO
OMe
^{1}H-^{1}H COSY (D$_2$O, 700 MHz)
f2 (ppm)
f1 (ppm)

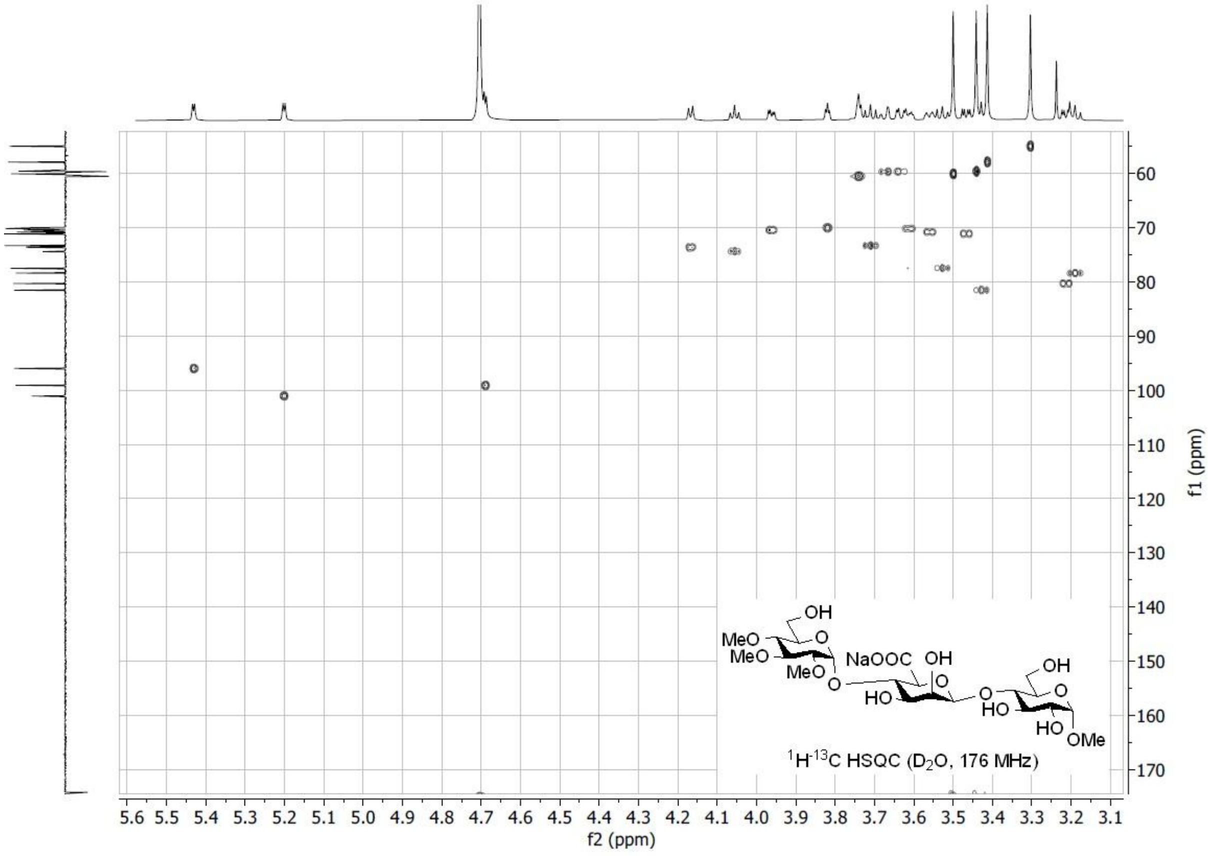

OH
MeO
MeO
O
MeO
NaOOC OH
O
HO
O
O
OH
O
HO
HO
OMe
^{1}H-^{13}C HSQC (D$_2$O, 176 MHz)
f2 (ppm)
f1 (ppm)

^{1}H, ^{13}C, COSY and HSQC NMR spectra of compound **6**

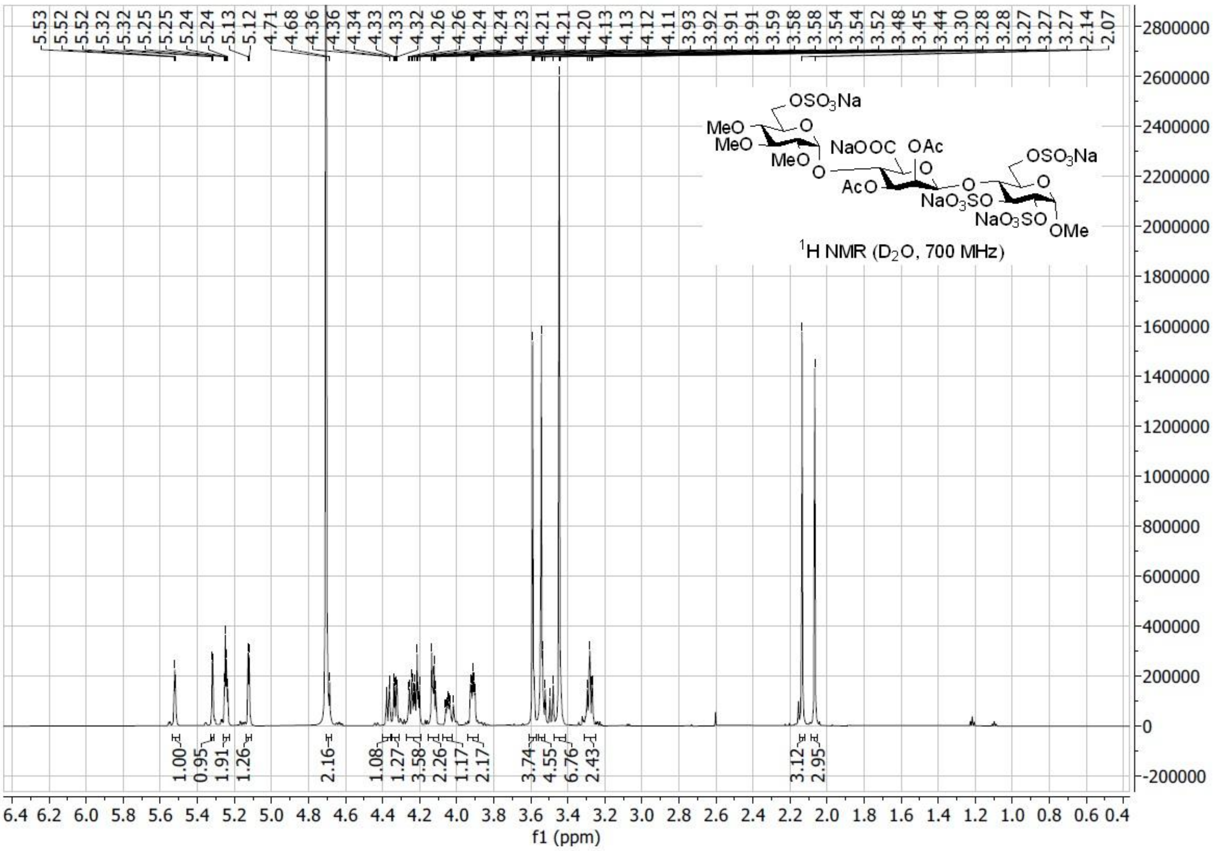

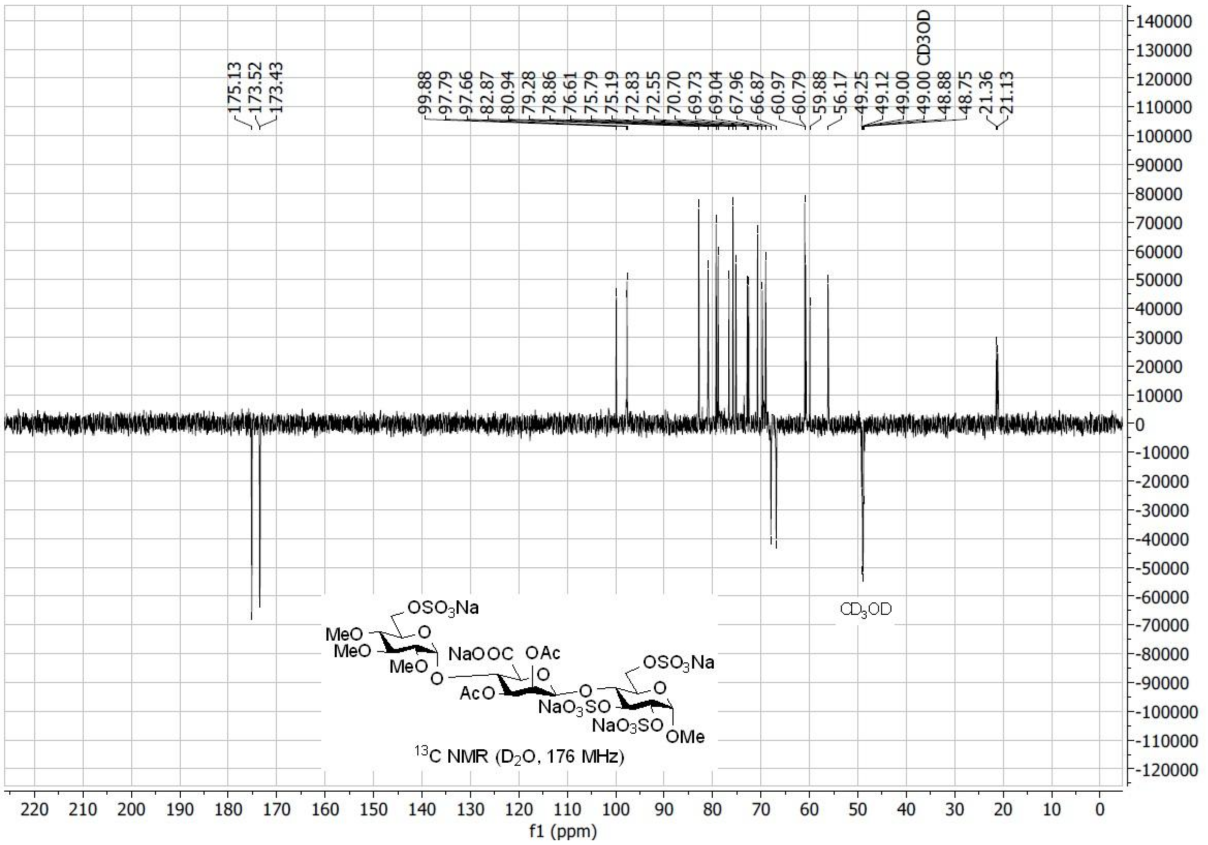

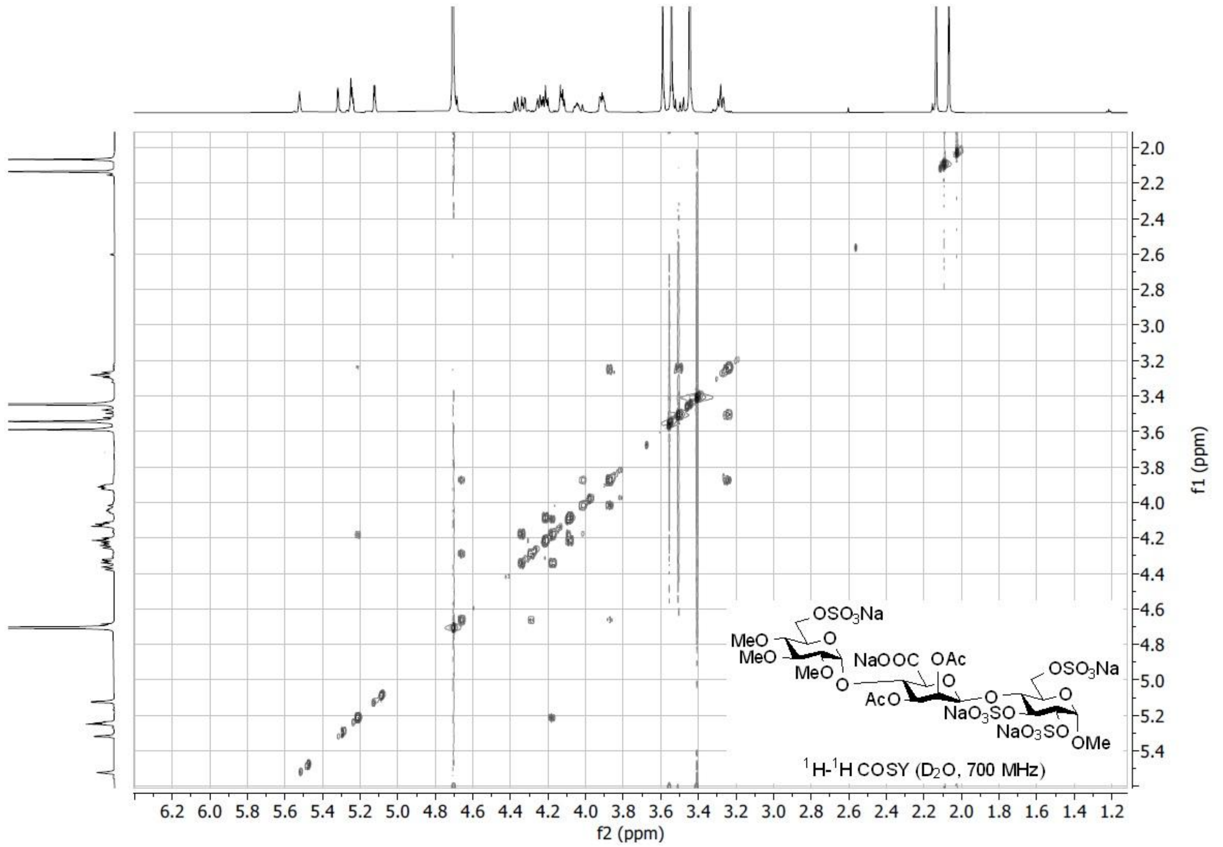
OSO3Na
MeO
MeO
MeO
NaOOC
OAc
AcO
NaO3SO
OSO3Na
NaO3SO
OMe
1H-1H COSY (D2O, 700 MHz)
f2 (ppm)
f1 (ppm)

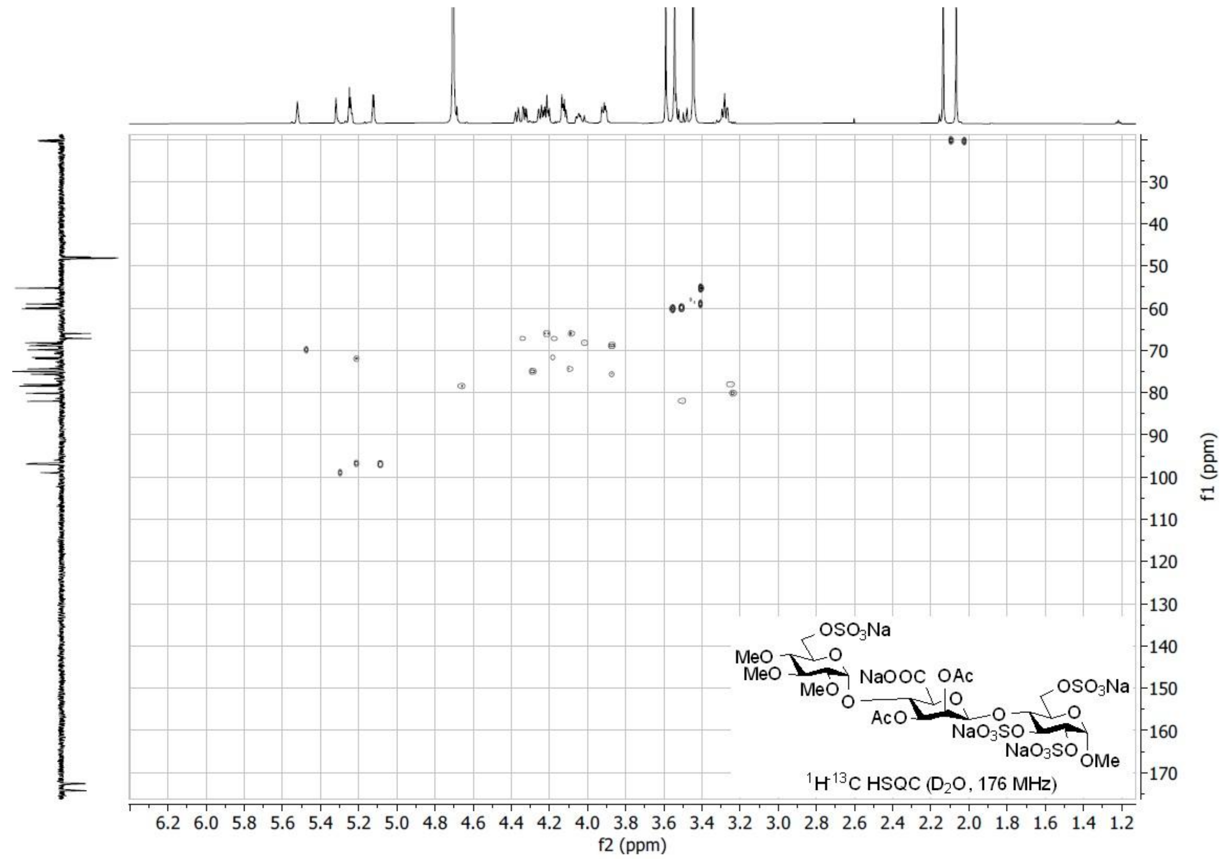
OSO3Na
MeO
MeO
MeO
NaOOC
OAc
AcO
NaO3SO
OSO3Na
NaO3SO
OMe
1H-13C HSQC (D2O, 176 MHz)
f2 (ppm)
f1 (ppm)

^{1}H, ^{13}C, COSY and HSQC NMR spectra of compound **7**

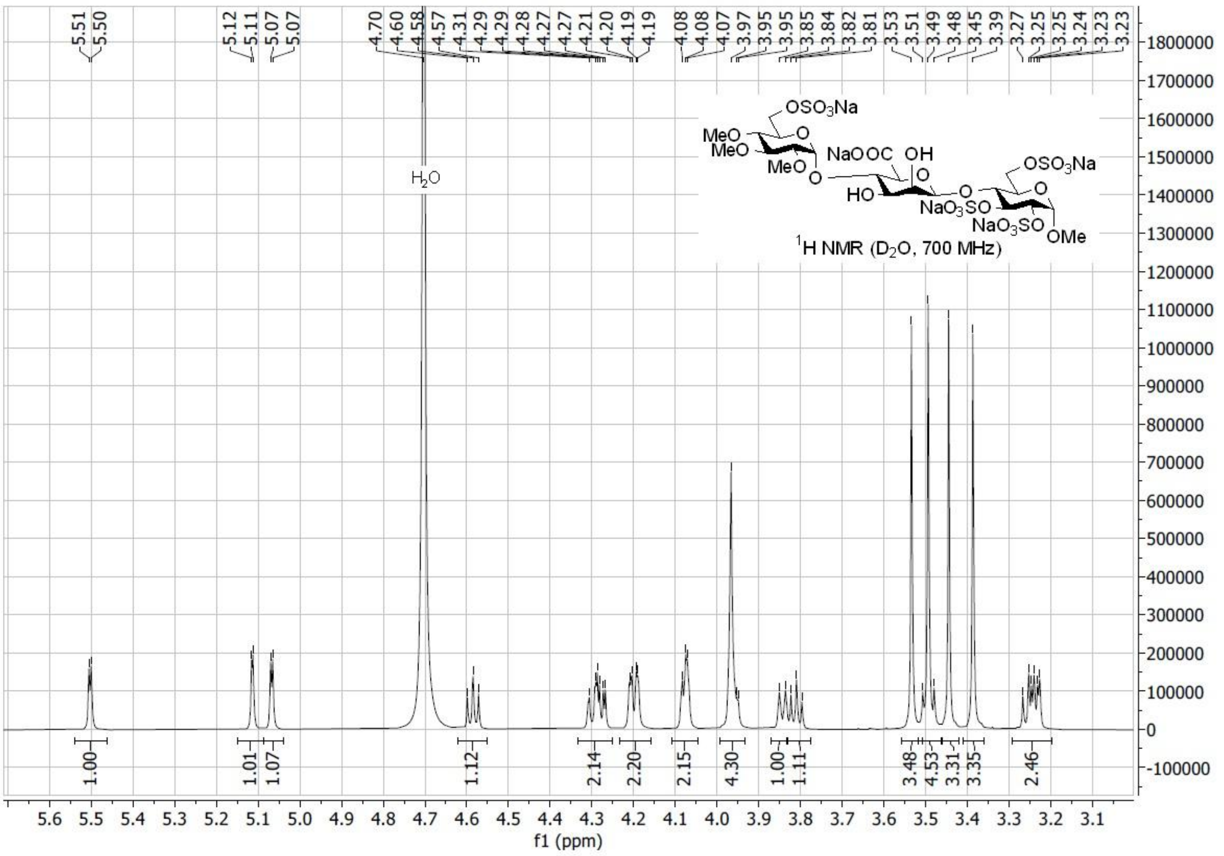

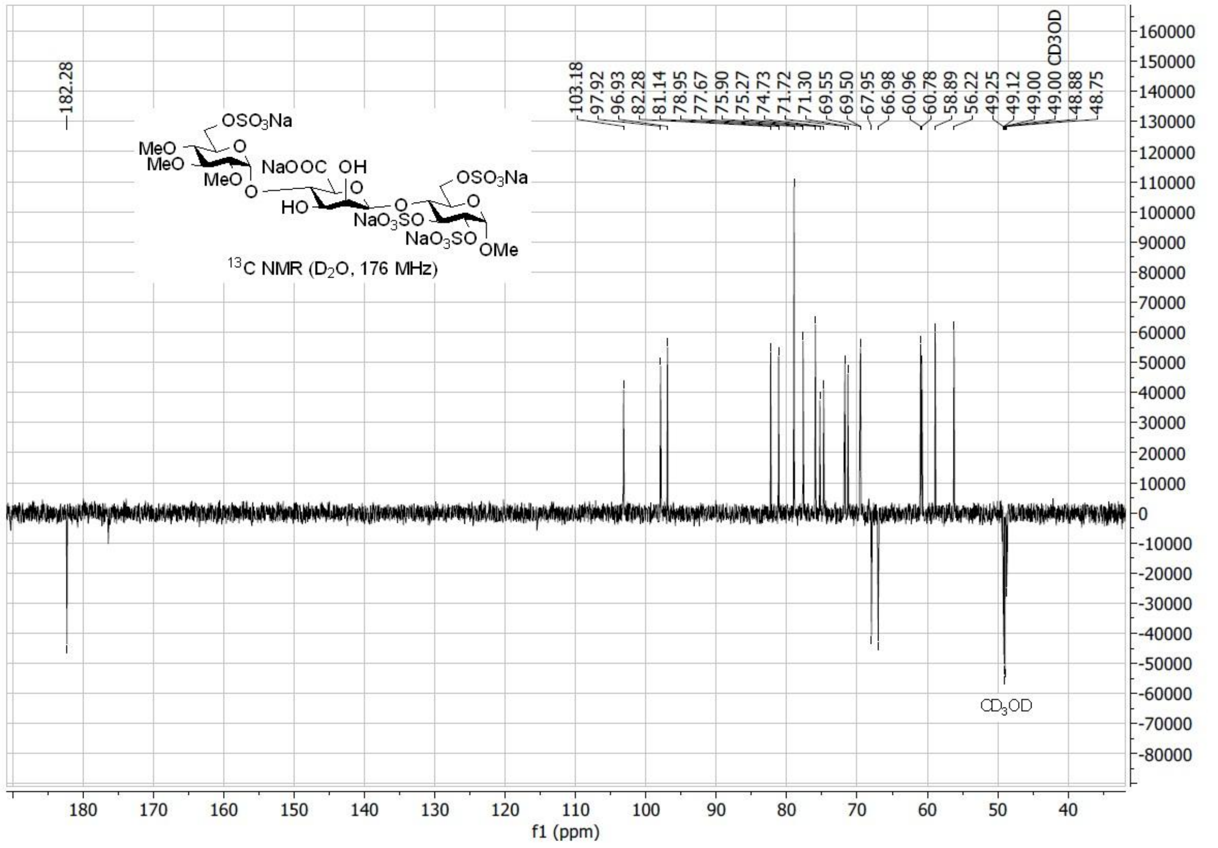

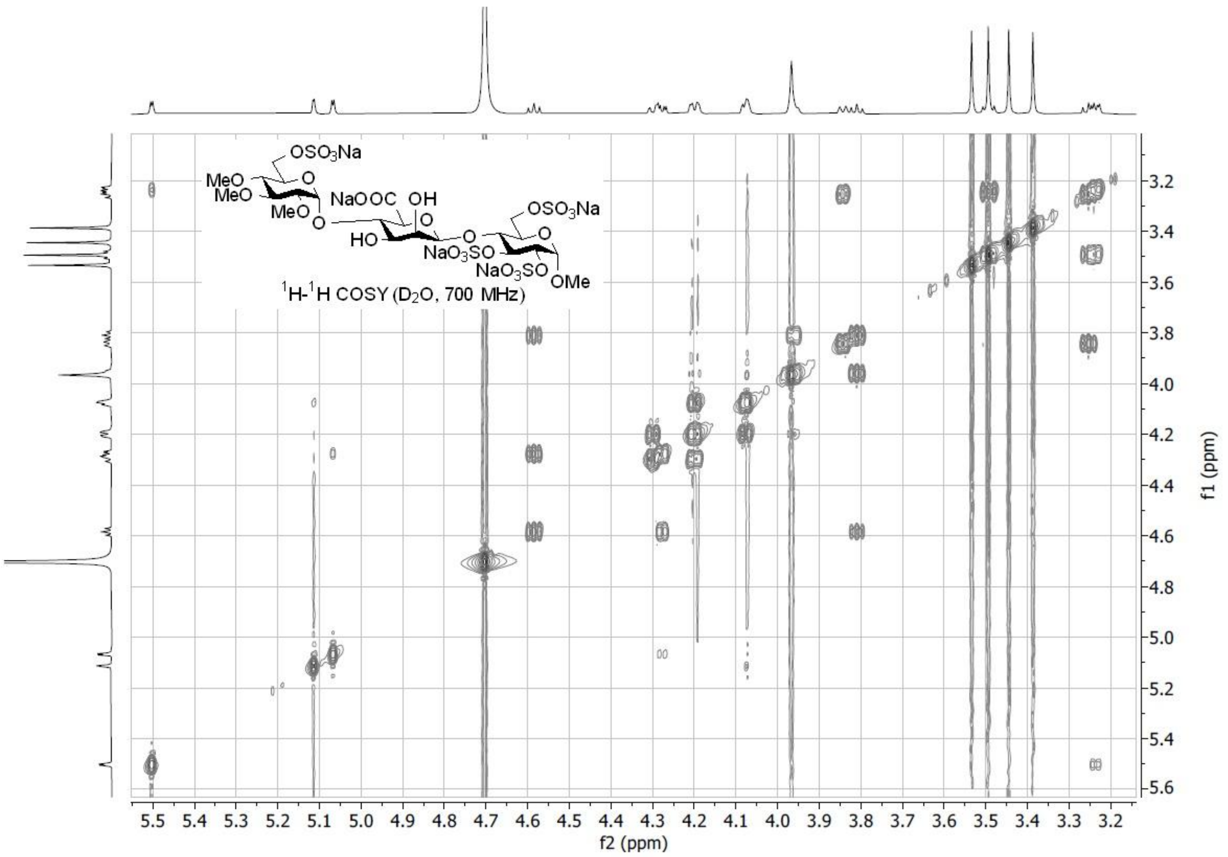

OSO3Na
MeO
MeO
MeO
NaOOC OH
HO
NaO3SO
OSO3Na
NaO3SO
OMe
1H-1H COSY (D2O, 700 MHz)
f2 (ppm)
f1 (ppm)

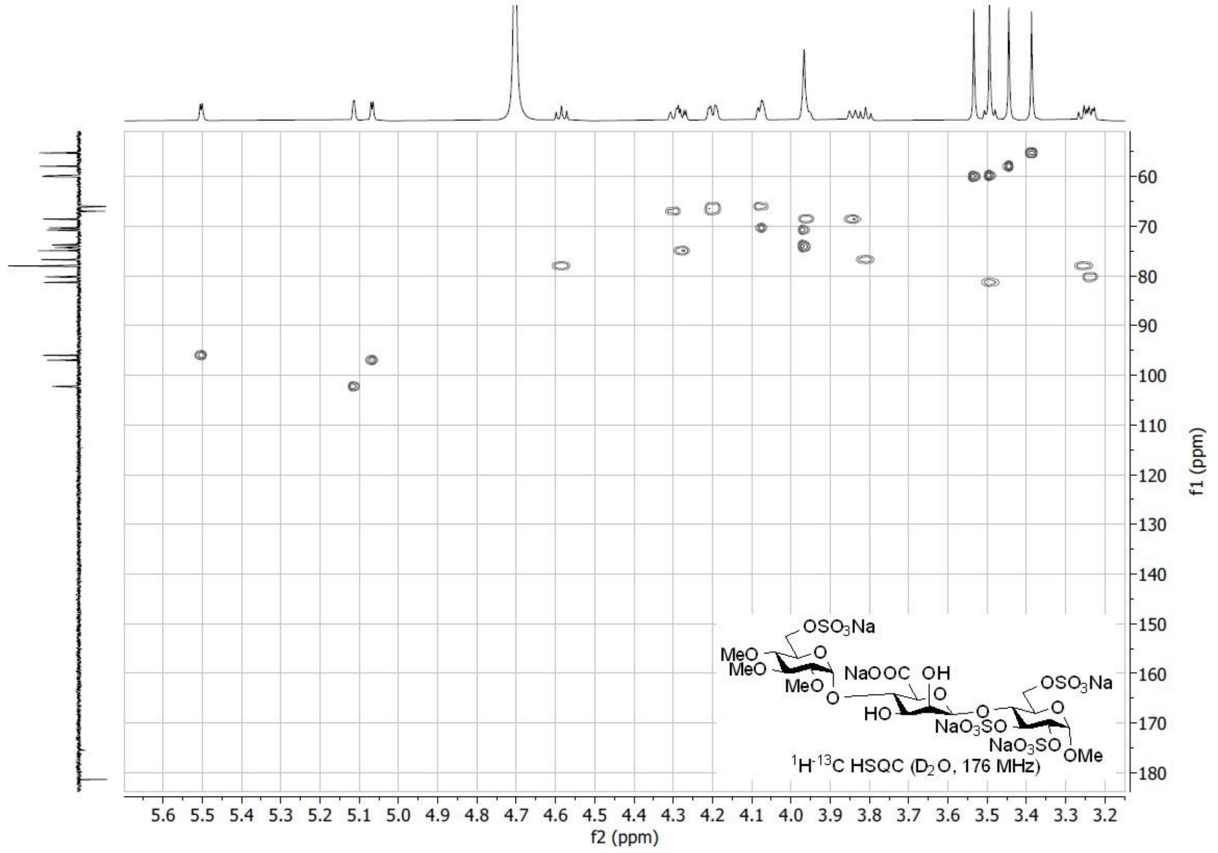

OSO3Na
MeO
MeO
MeO
NaOOC OH
HO
NaO3SO
OSO3Na
NaO3SO
OMe
1H-13C HSQC (D2O, 176 MHz)
f2 (ppm)
f1 (ppm)

^{1}H, ^{13}C, COSY and HSQC NMR spectra of compound **8**

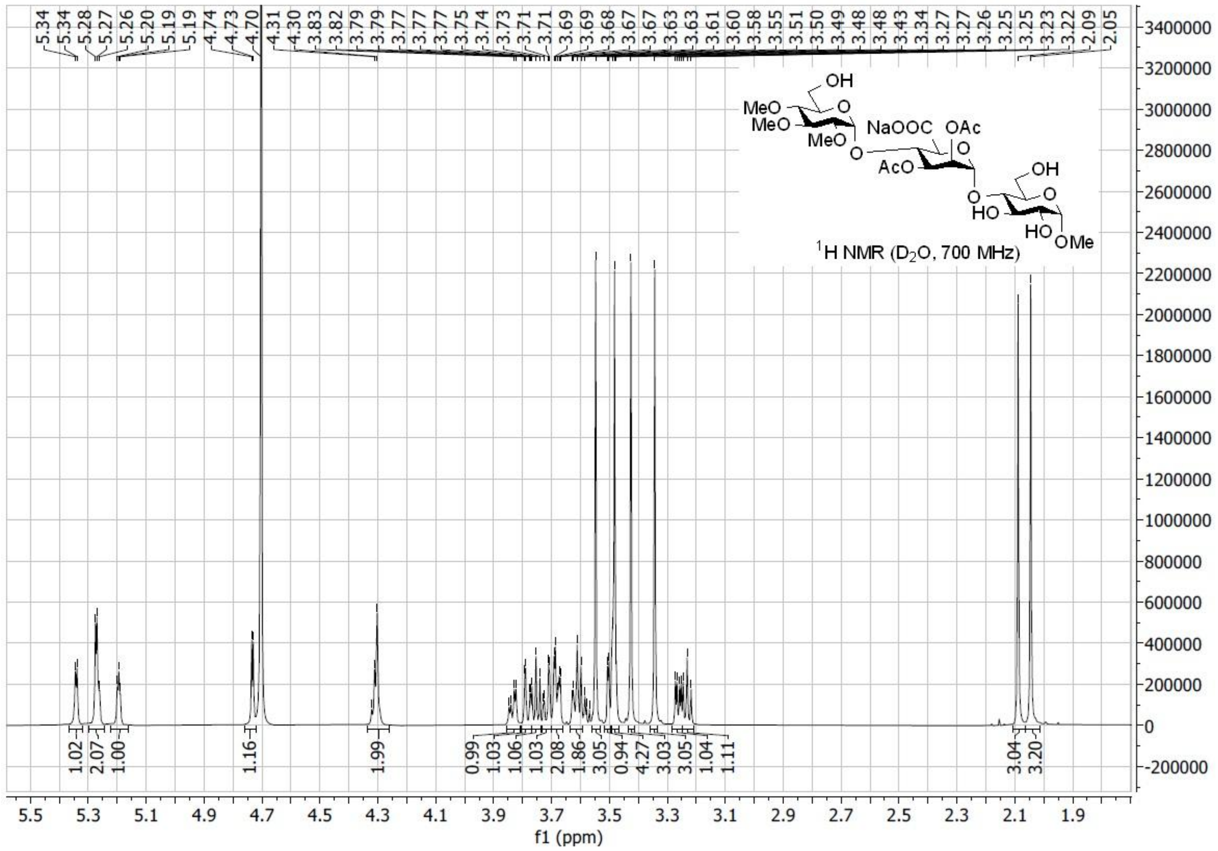

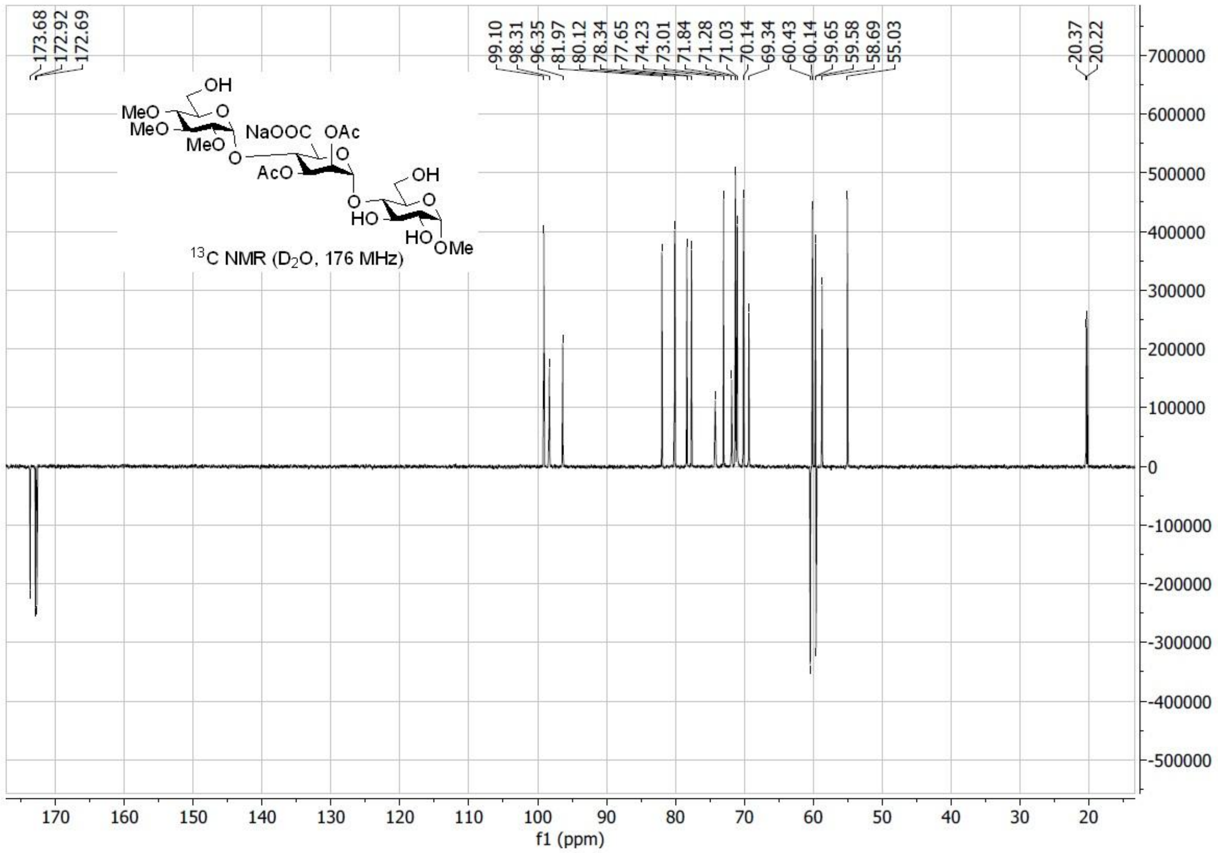

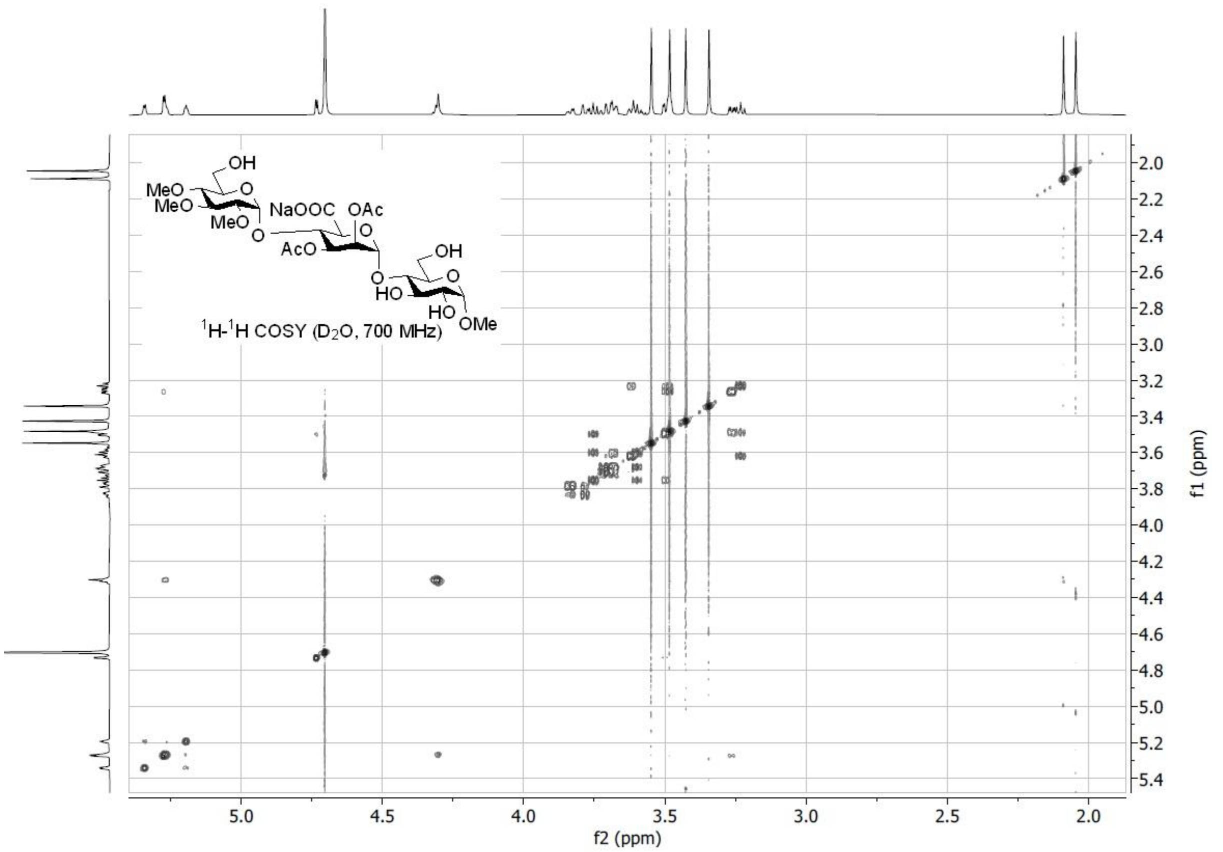
OH
MeO
MeO
MeO
O
NaOOC
OAc
O
AcO
O
OH
O
HO
HO
OMe
1H-1H COSY (D2O, 700 MHz)
f1 (ppm)
f2 (ppm)

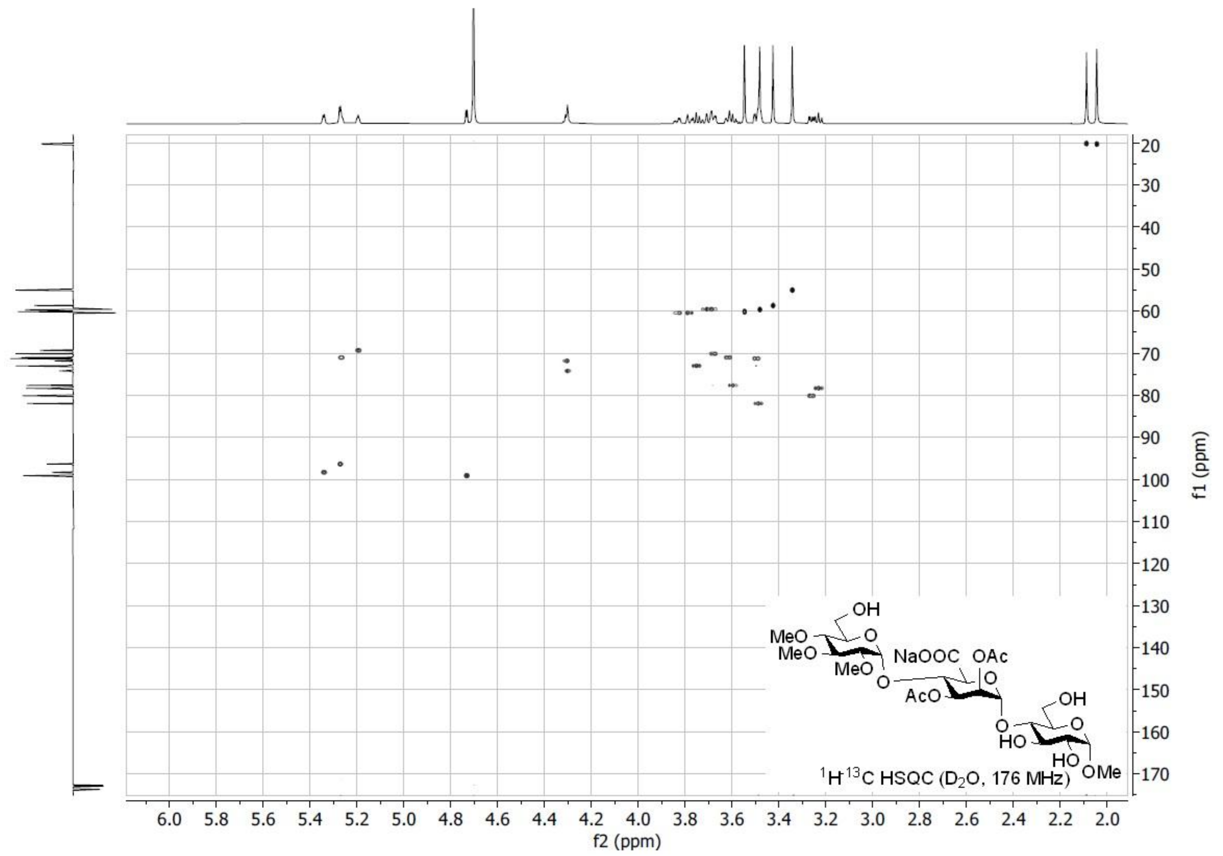
OH
MeO
MeO
MeO
O
NaOOC
OAc
O
AcO
O
OH
O
HO
HO
OMe
1H-13C HSQC (D2O, 176 MHz)
f1 (ppm)
f2 (ppm)

^{1}H, ^{13}C, COSY and HSQC NMR spectra of compound **9**

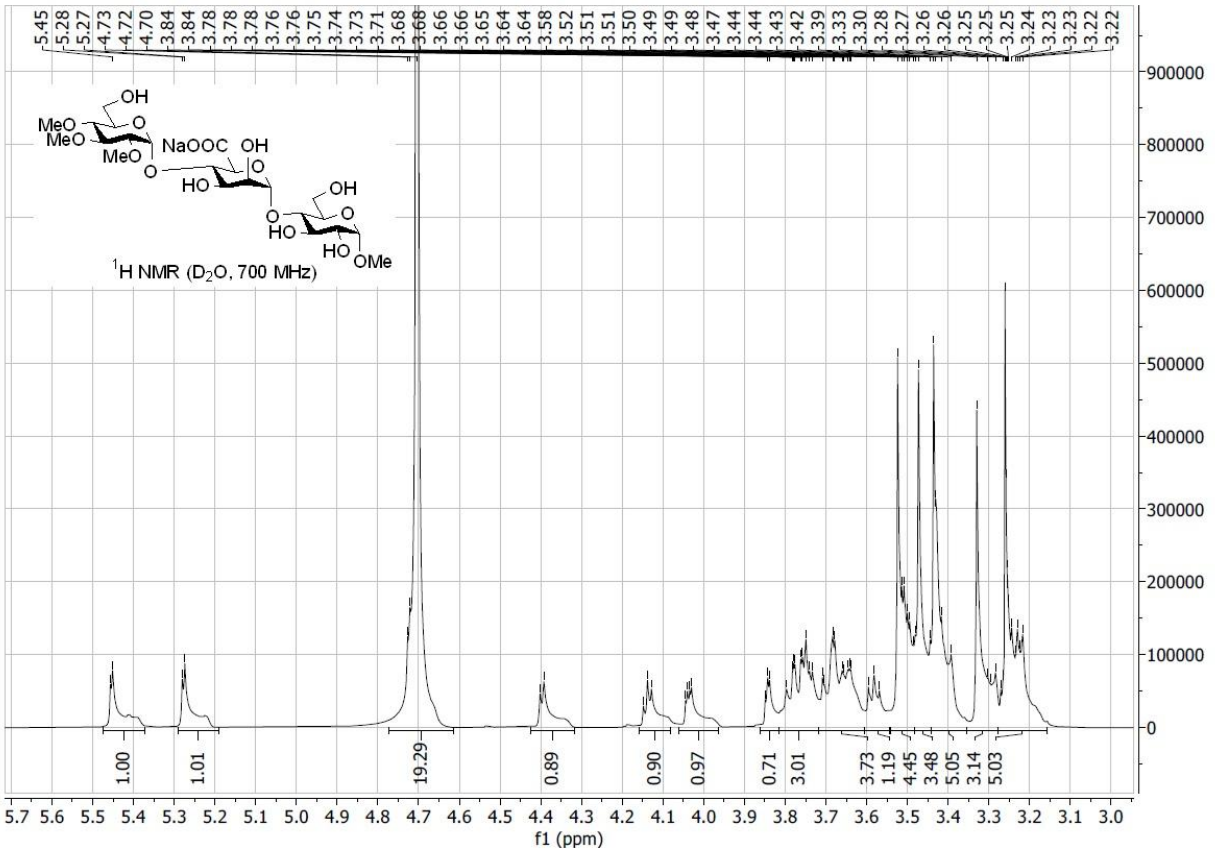

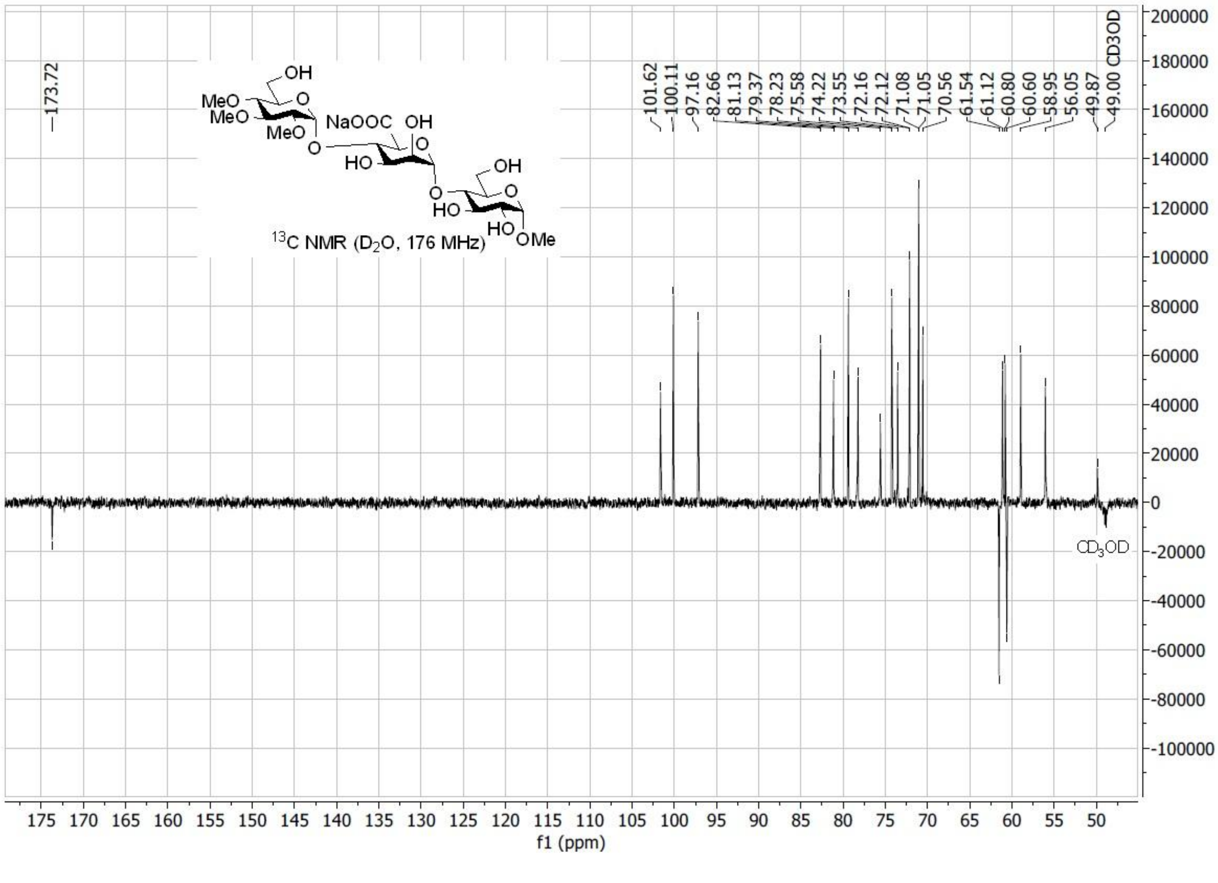

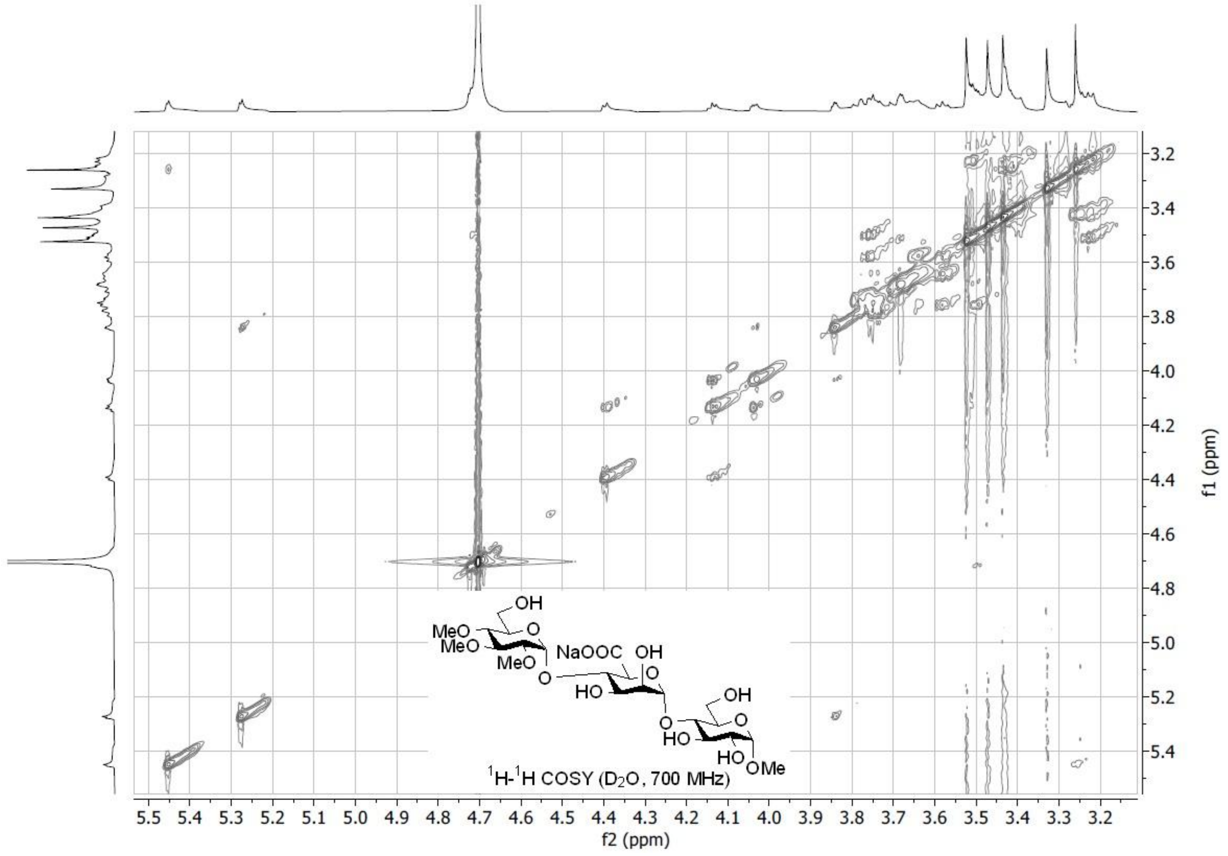

OH
MeO
MeO
O
MeO
NaOOC OH
O
HO
O
OH
O
HO
O
HO
OMe
^1H-^1H COSY (D_2O, 700 MHz)
f1 (ppm)
f2 (ppm)

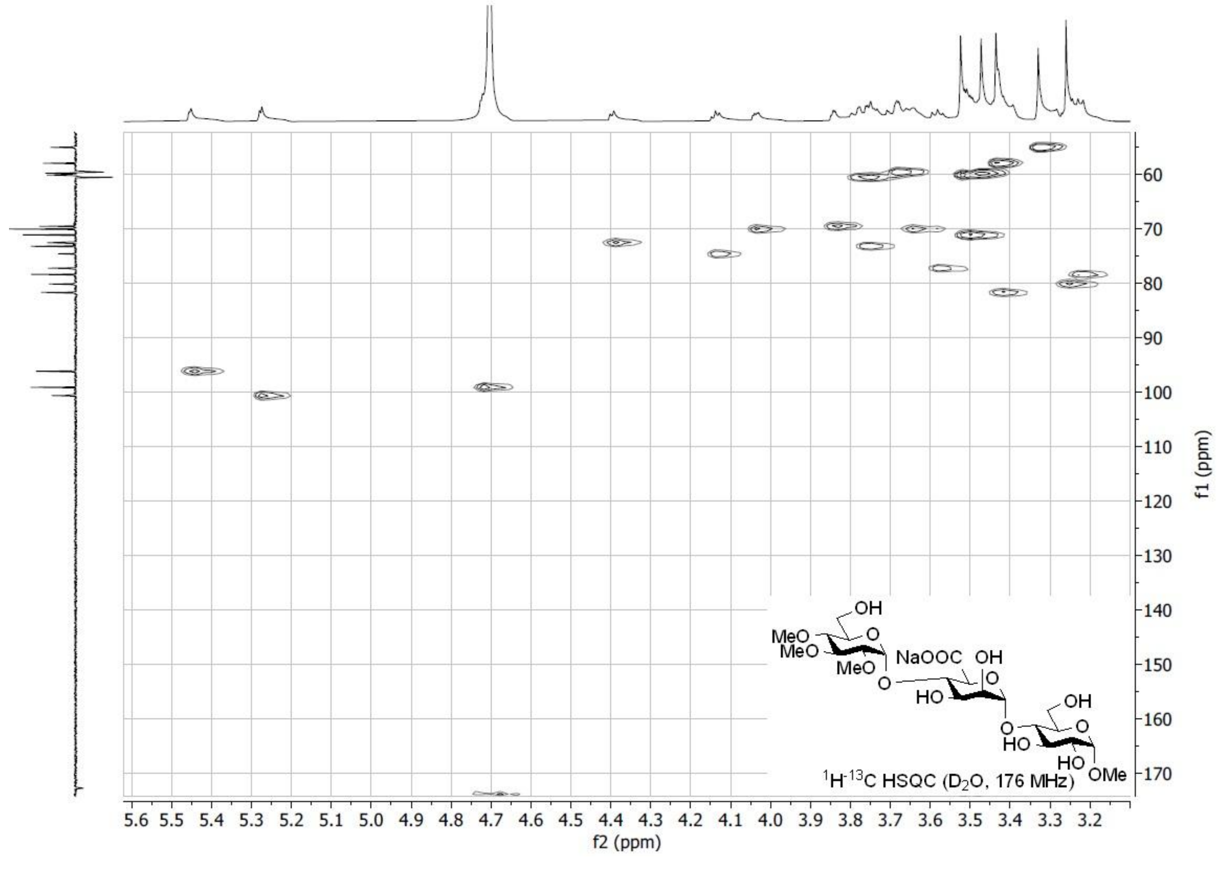

OH
MeO
MeO
O
MeO
NaOOC OH
O
HO
O
OH
O
HO
O
HO
OMe
^1H-^{13}C HSQC (D_2O, 176 MHz)
f1 (ppm)
f2 (ppm)

^{1}H, ^{13}C, COSY and HSQC NMR spectra of compound **11**

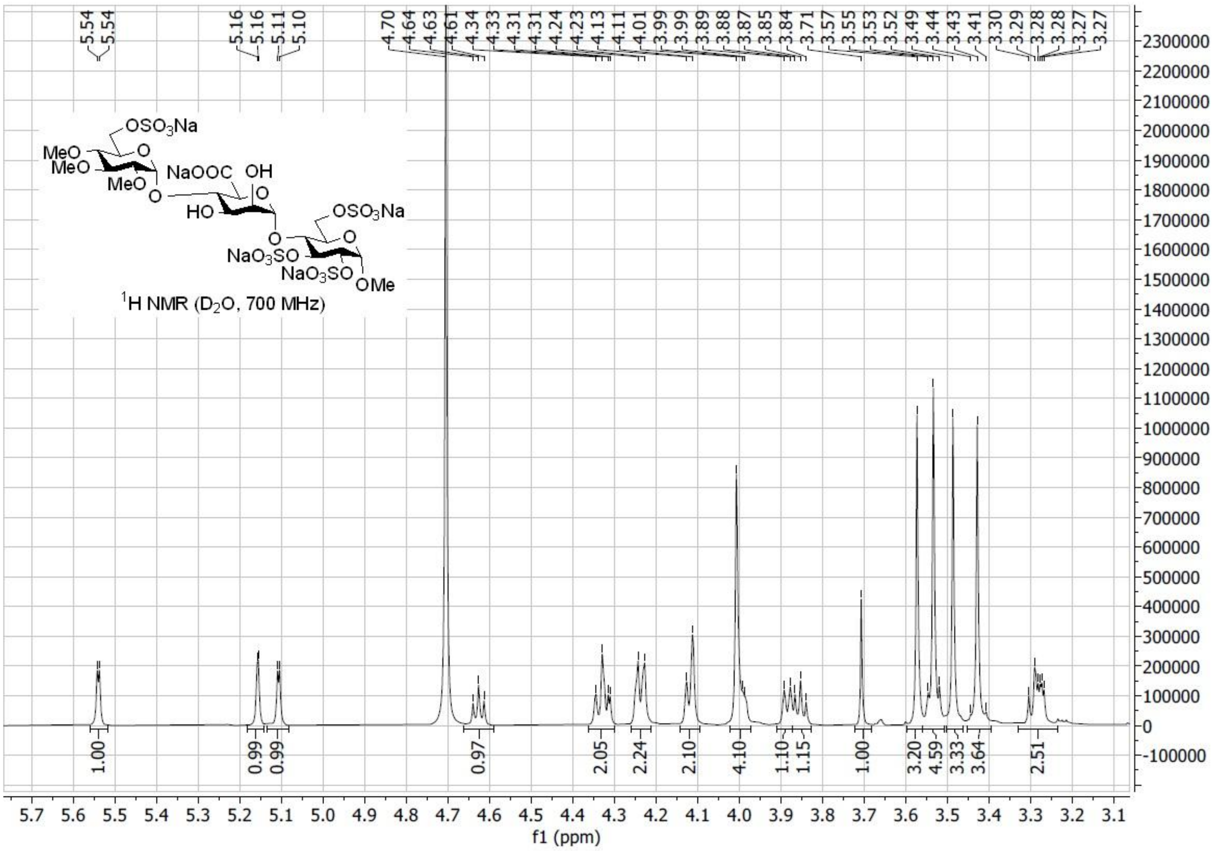

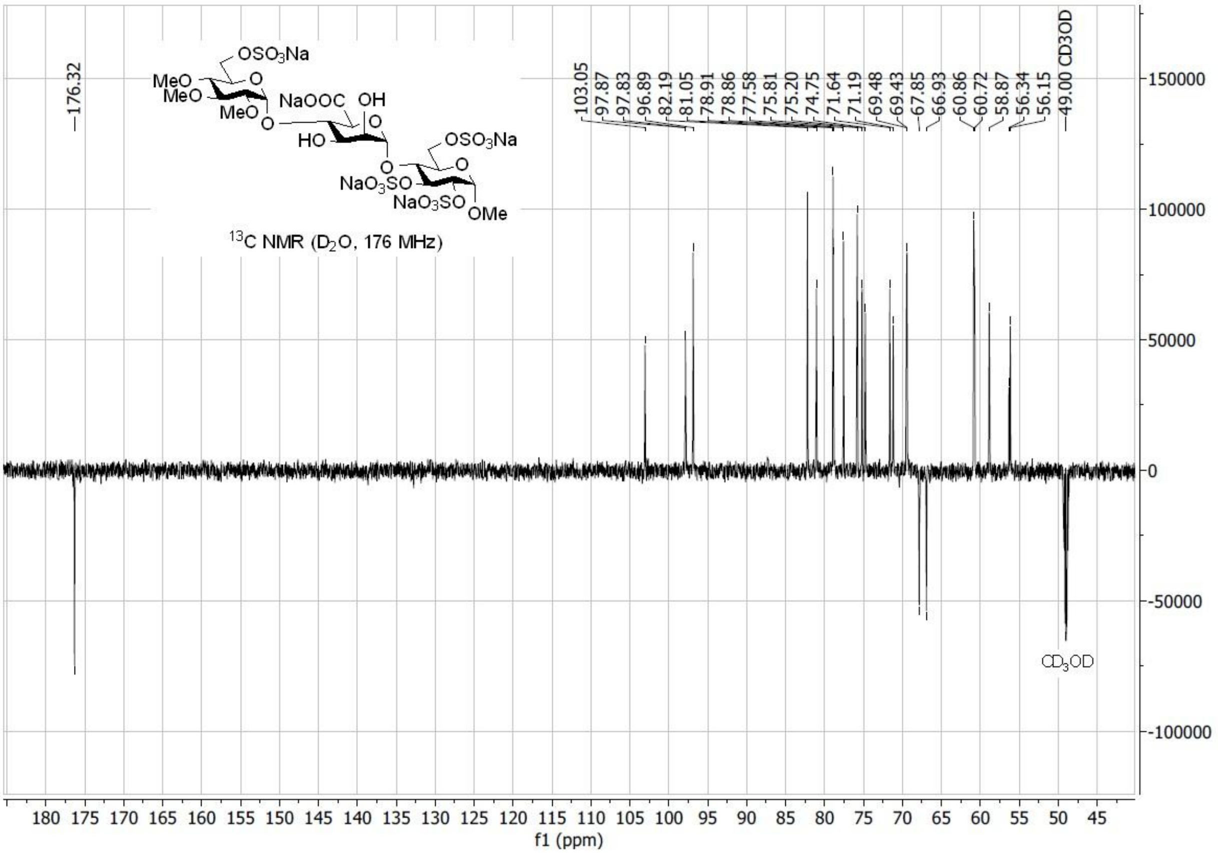

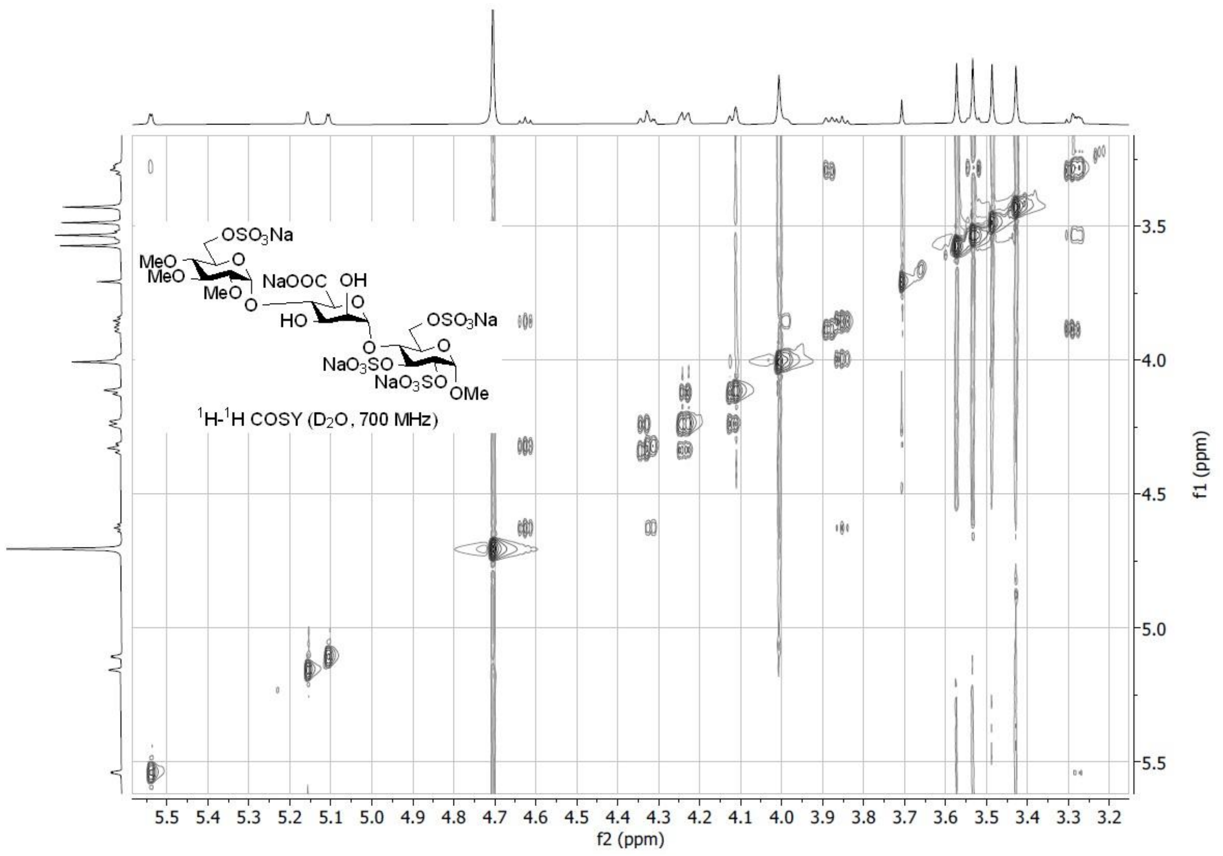

OSO3Na
MeO
MeO
MeO
O
NaOOC OH
HO
OSO3Na
NaO3SO
NaO3SO
OMe
1H-1H COSY (D2O, 700 MHz)
f2 (ppm)
f1 (ppm)

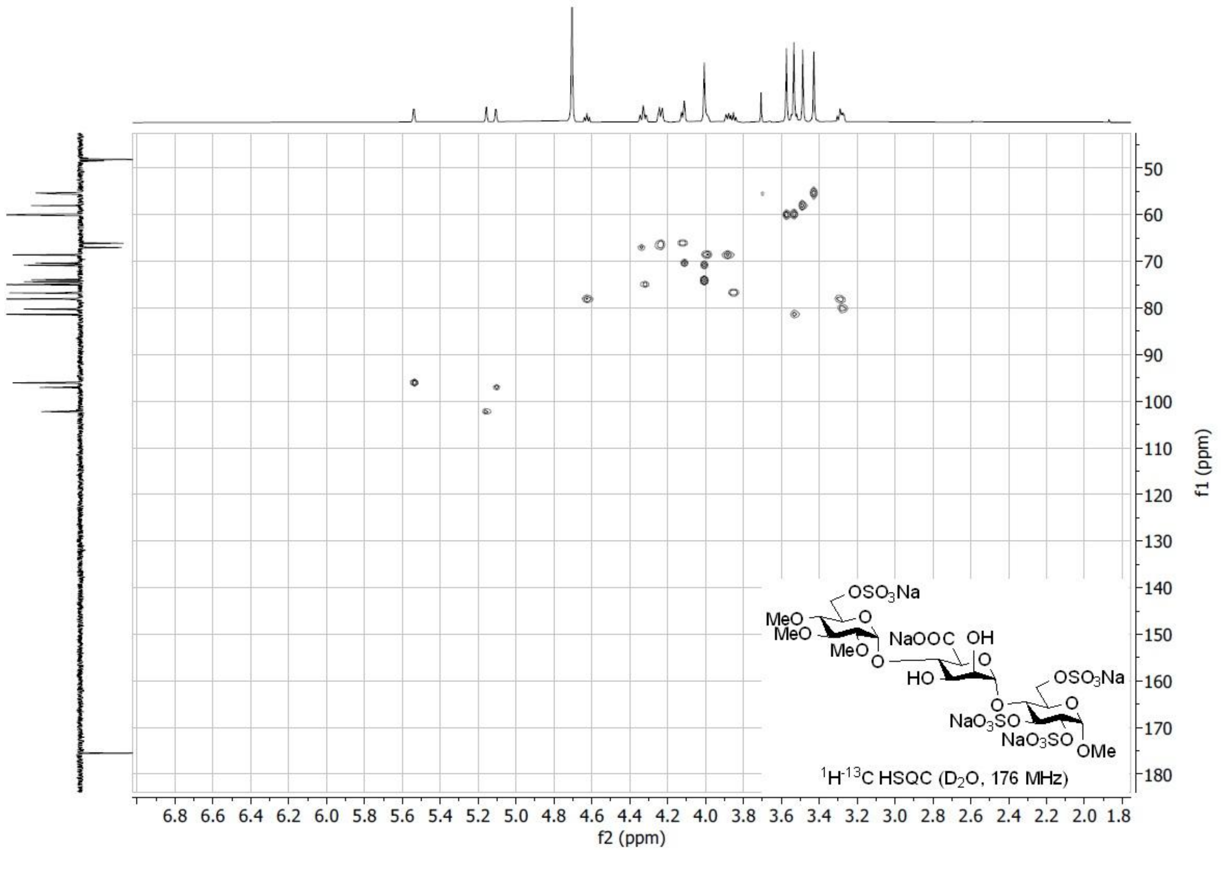

OSO3Na
MeO
MeO
MeO
NaOOC OH
HO
OSO3Na
NaO3SO
NaO3SO
OMe
1H-13C HSQC (D2O, 176 MHz)
f2 (ppm)
f1 (ppm)

^{1}H, ^{13}C, COSY and HSQC NMR spectra of compound **12**

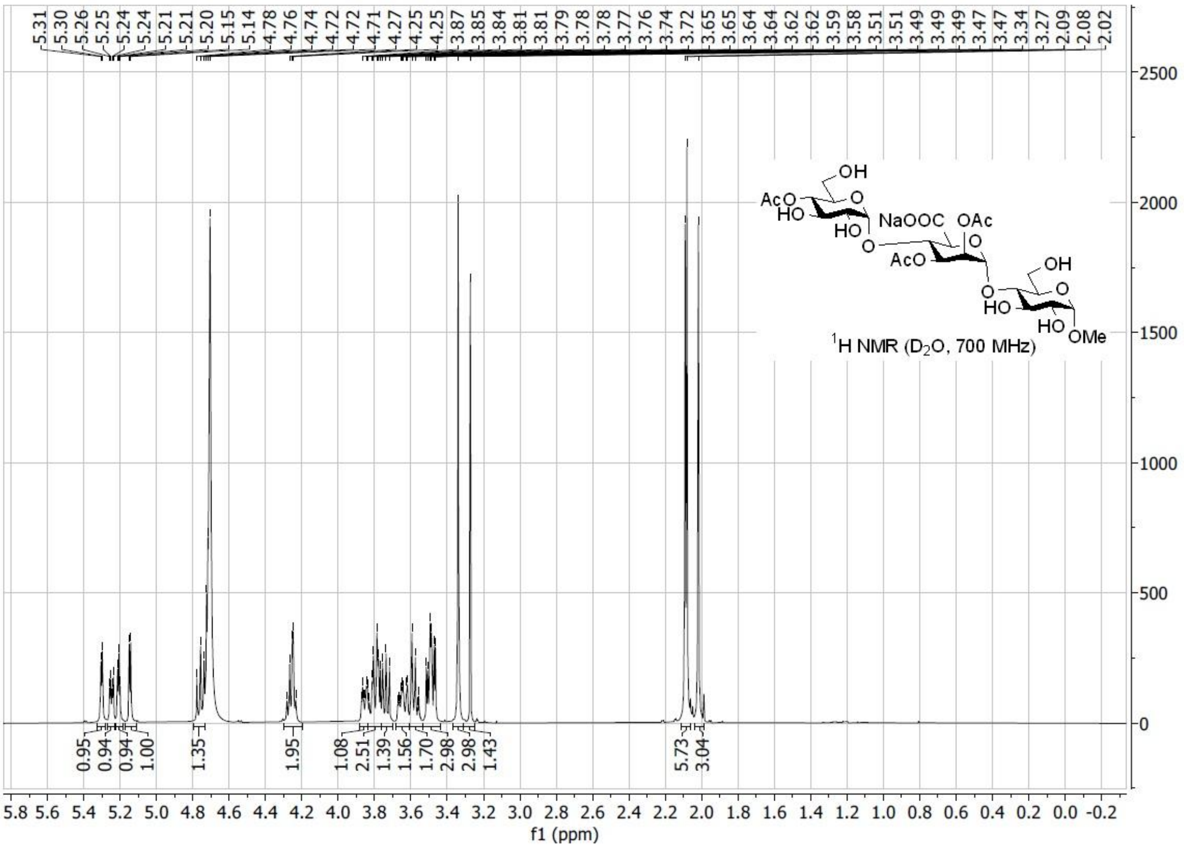

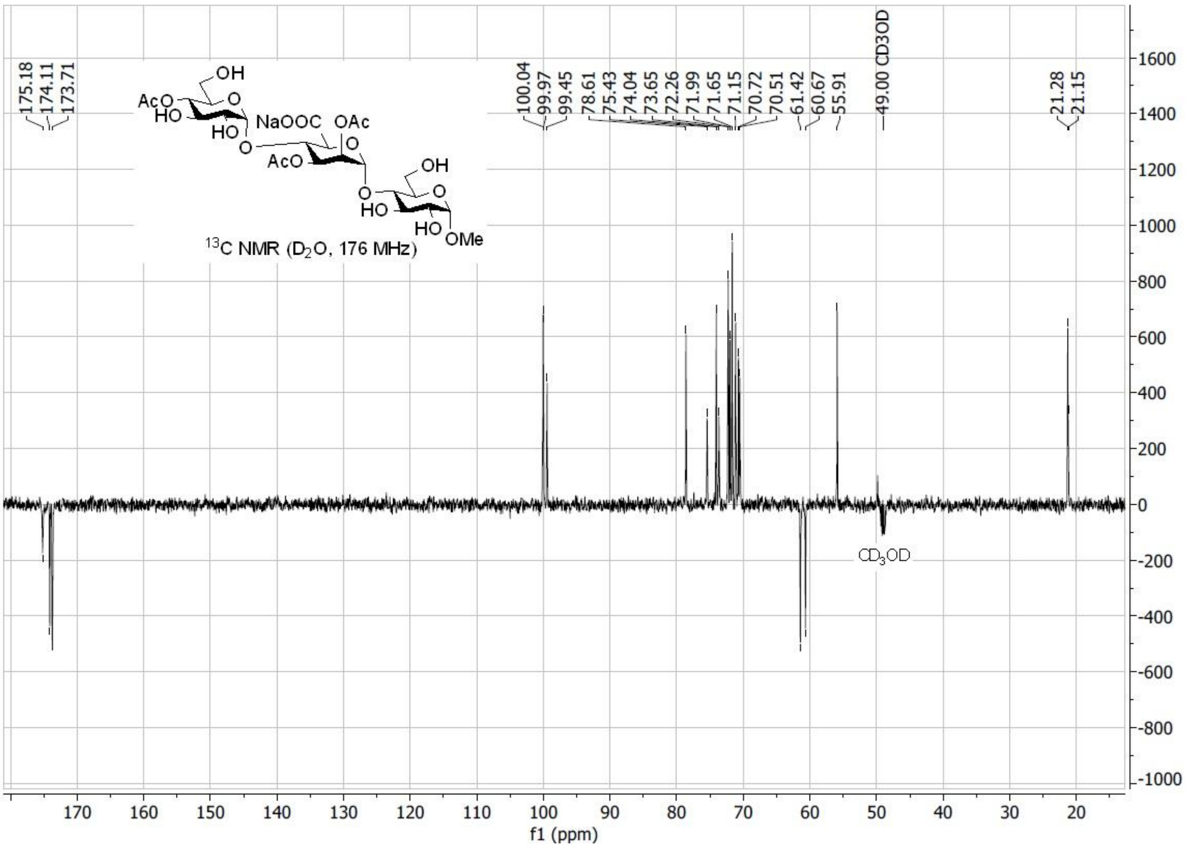

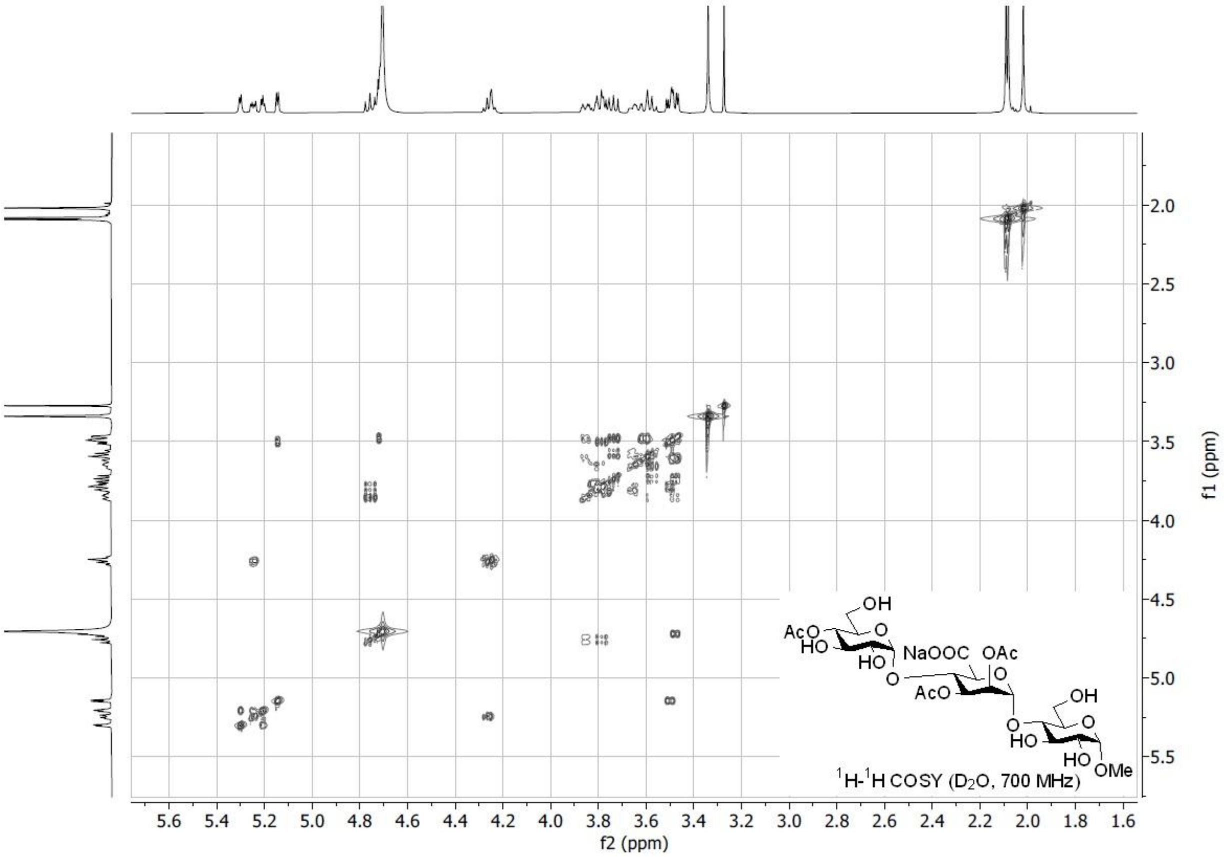
AcO
HO
OH
NaOOC
OAc
HO
AcO
OH
HO
HO
OMe
^{1}H-^{1}H COSY (D$_2$O, 700 MHz)
f1 (ppm)
f2 (ppm)

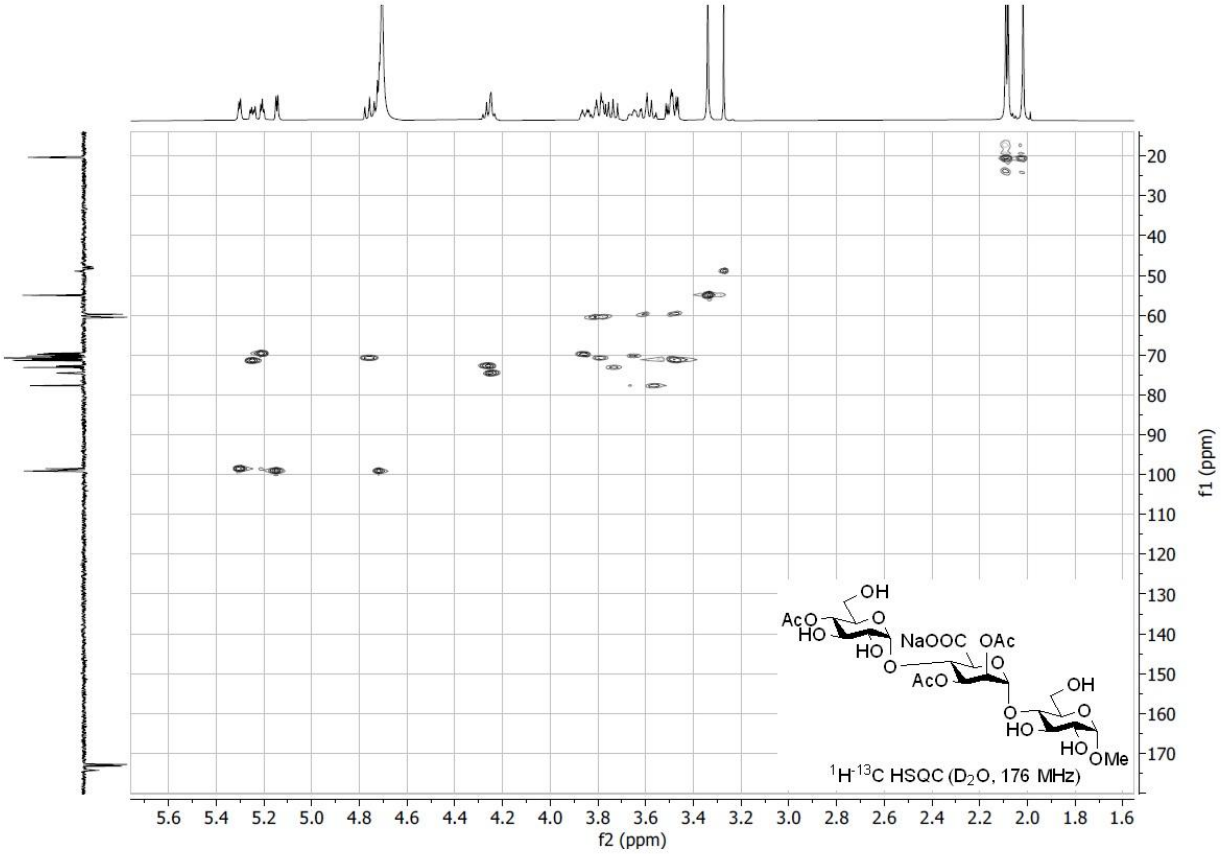
AcO
HO
OH
NaOOC
OAc
HO
AcO
OH
HO
HO
OMe
^{1}H-^{13}C HSQC (D$_2$O, 176 MHz)
f1 (ppm)
f2 (ppm)

^{1}H, ^{13}C, COSY and HSQC NMR spectra of compound **13**

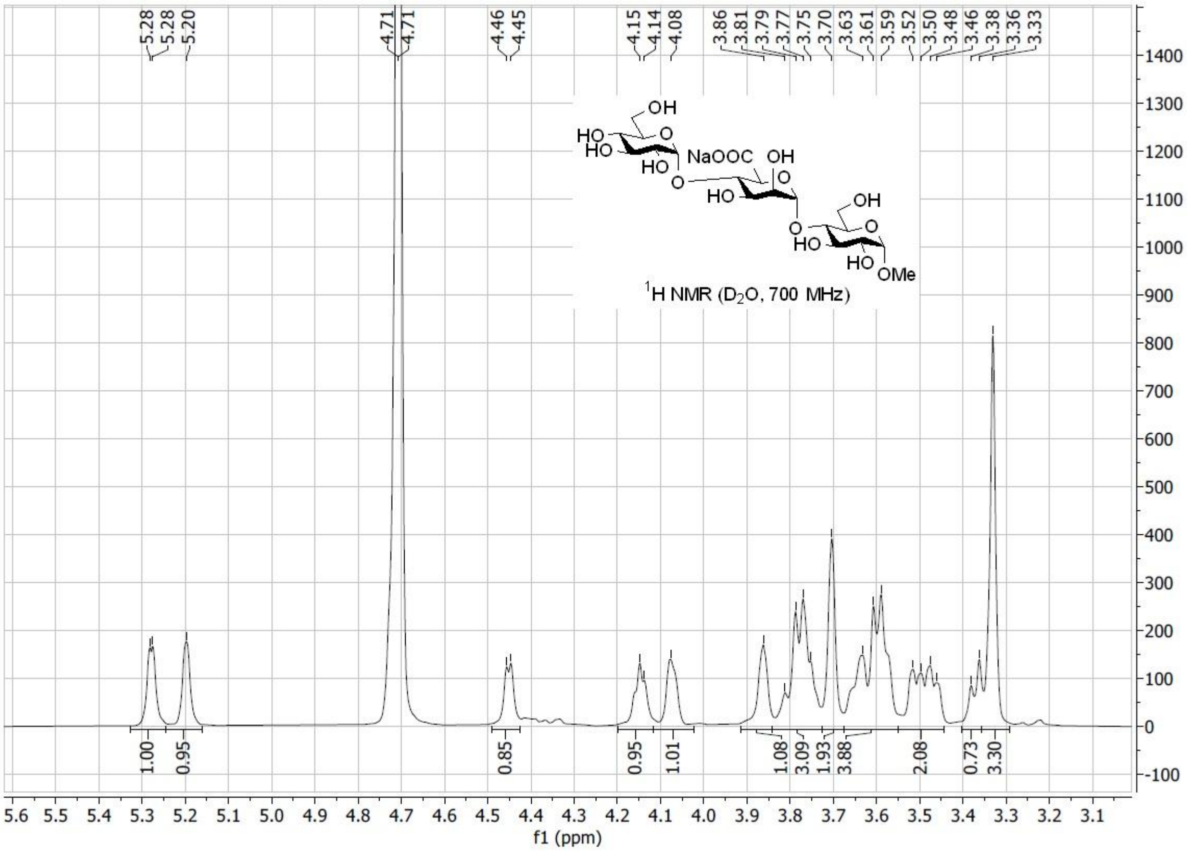

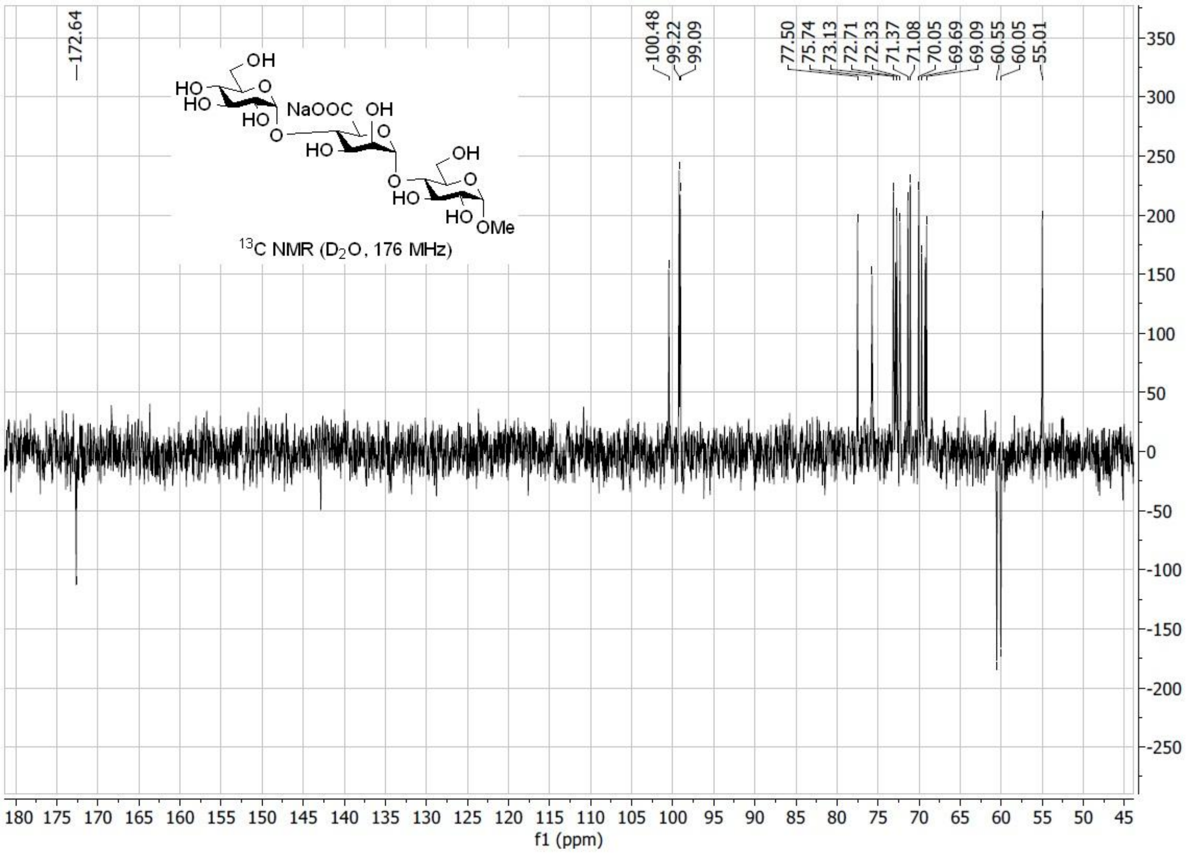

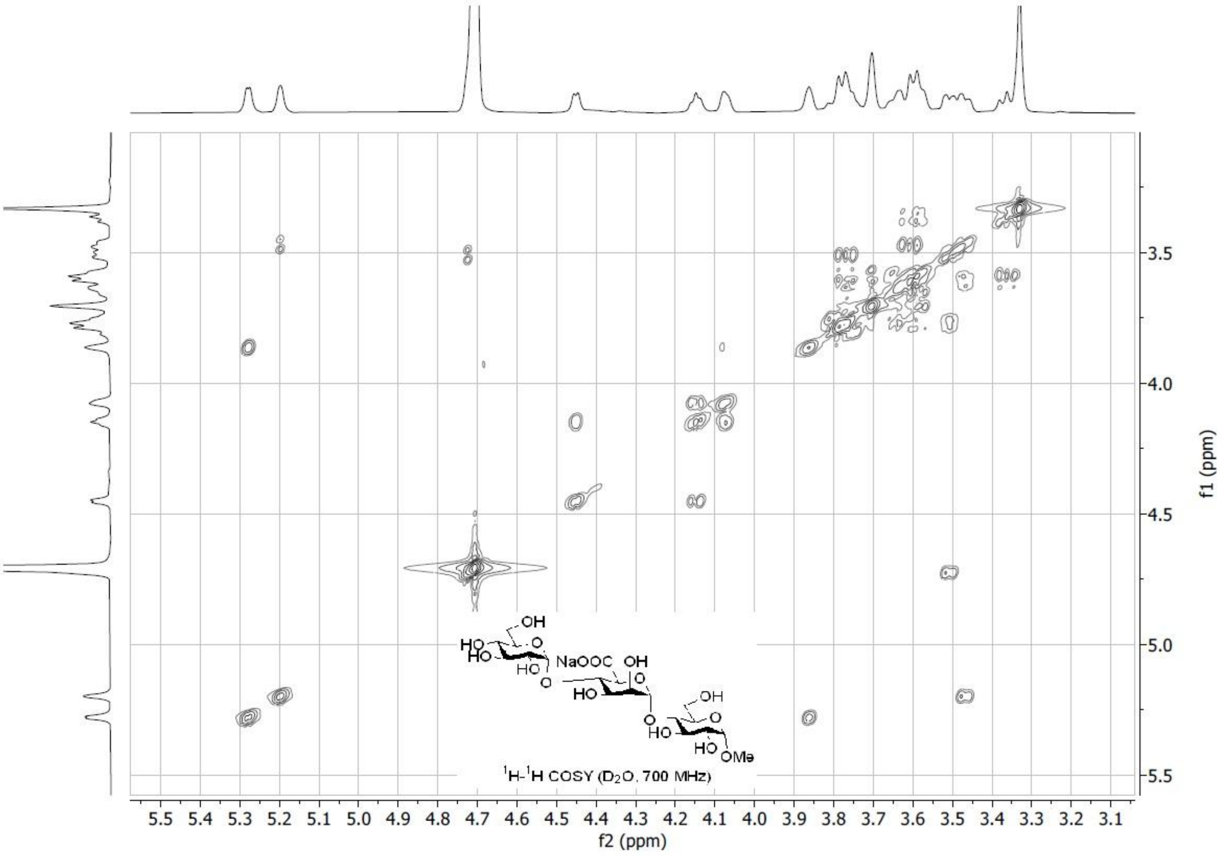
OH
HO
HO
HO
O
NaOOC
OH
HO
OH
HO
HO
OMe
1H-1H COSY (D2O, 700 MHz)
f1 (ppm)
f2 (ppm)

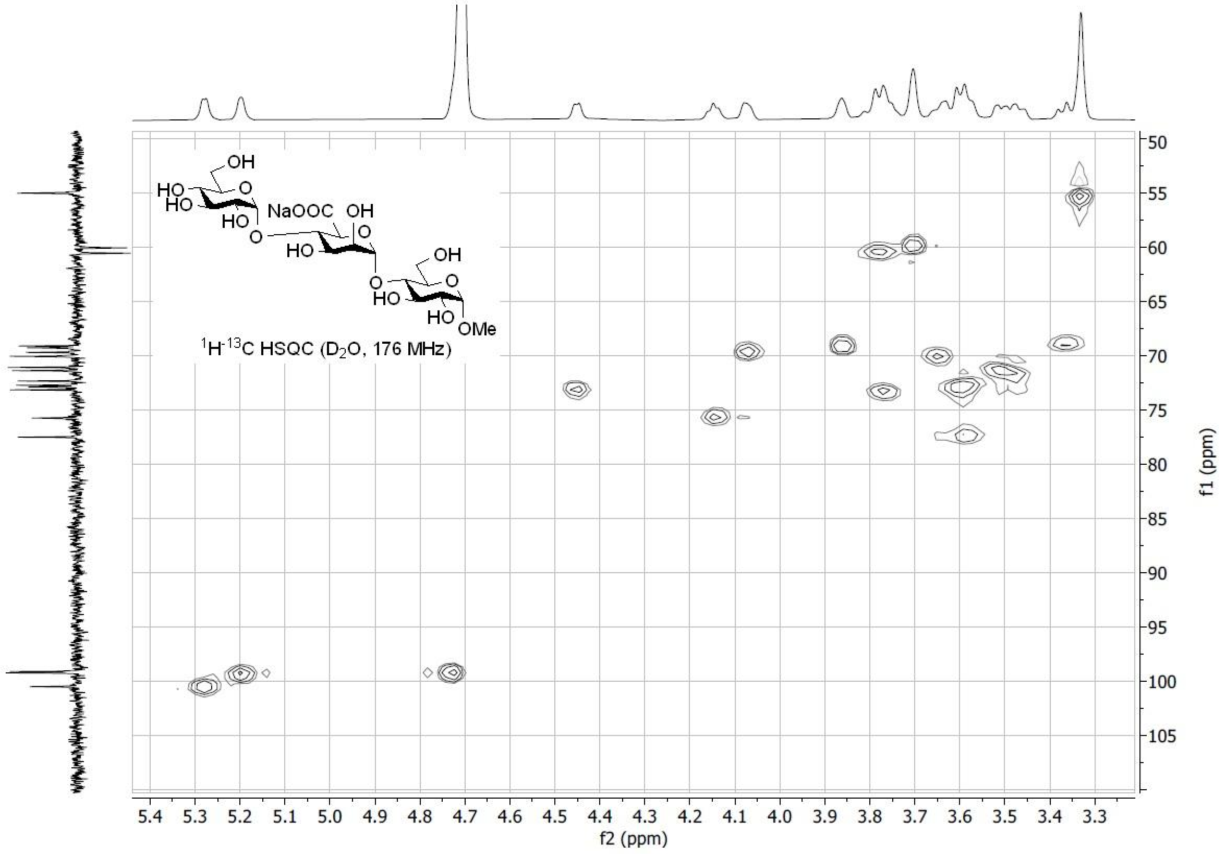
OH
HO
HO
HO
O
NaOOC
OH
HO
OH
HO
HO
OMe
1H-13C HSQC (D2O, 176 MHz)
f1 (ppm)
f2 (ppm)

^{1}H, ^{13}C, COSY and HSQC NMR spectra of compound **14**

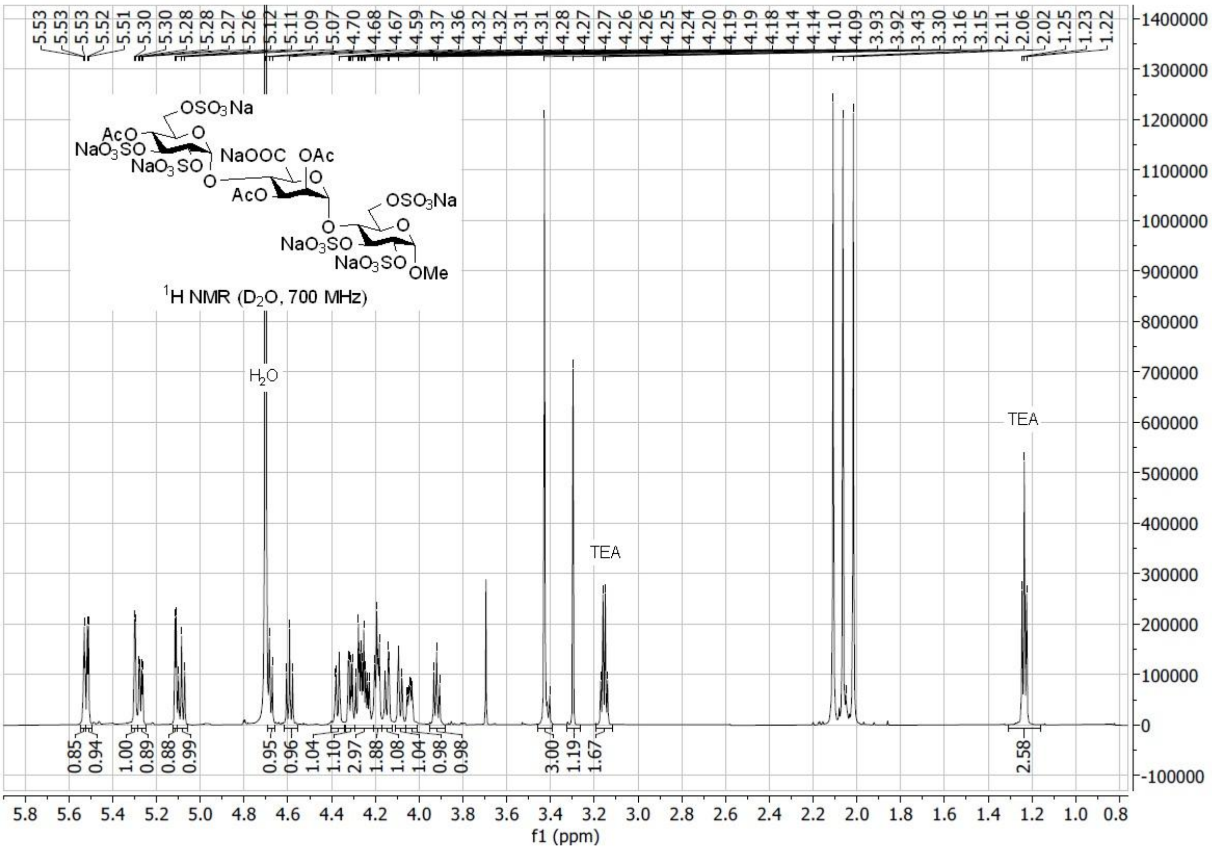

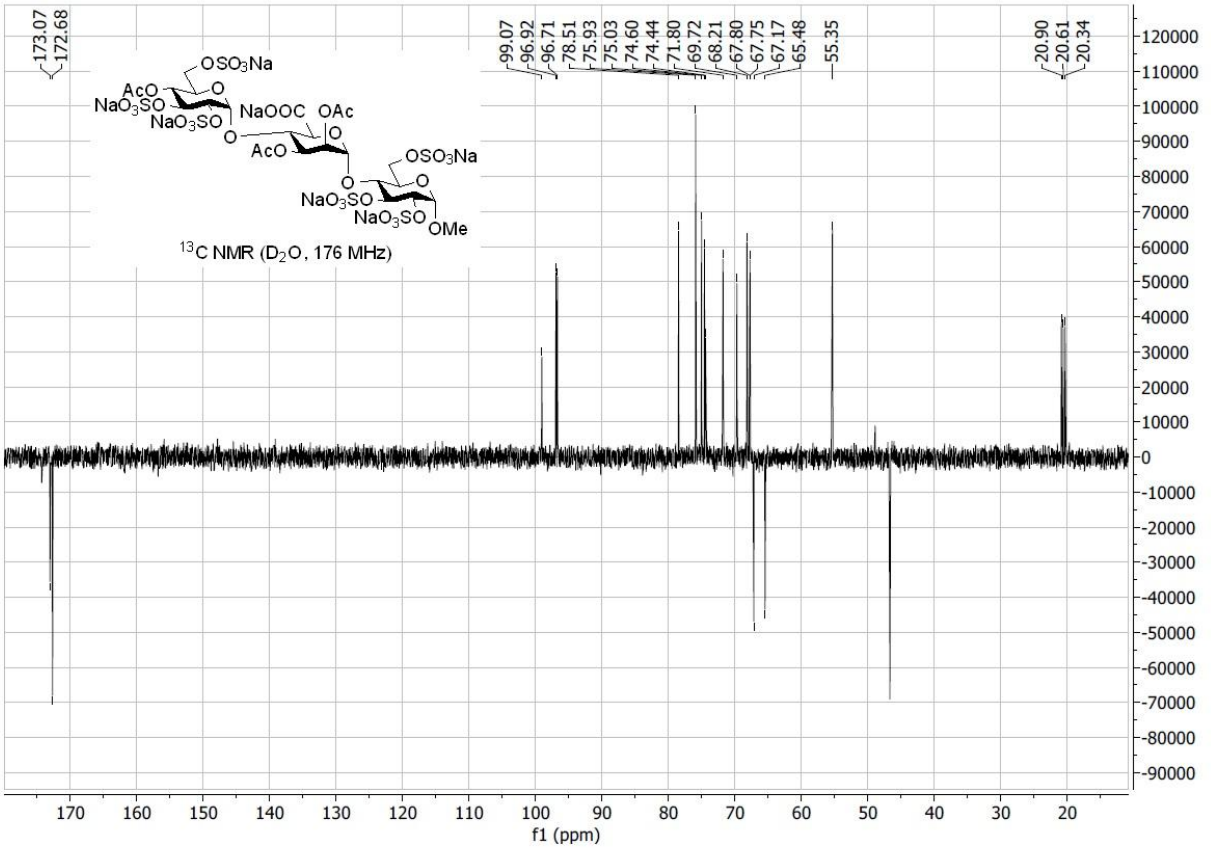

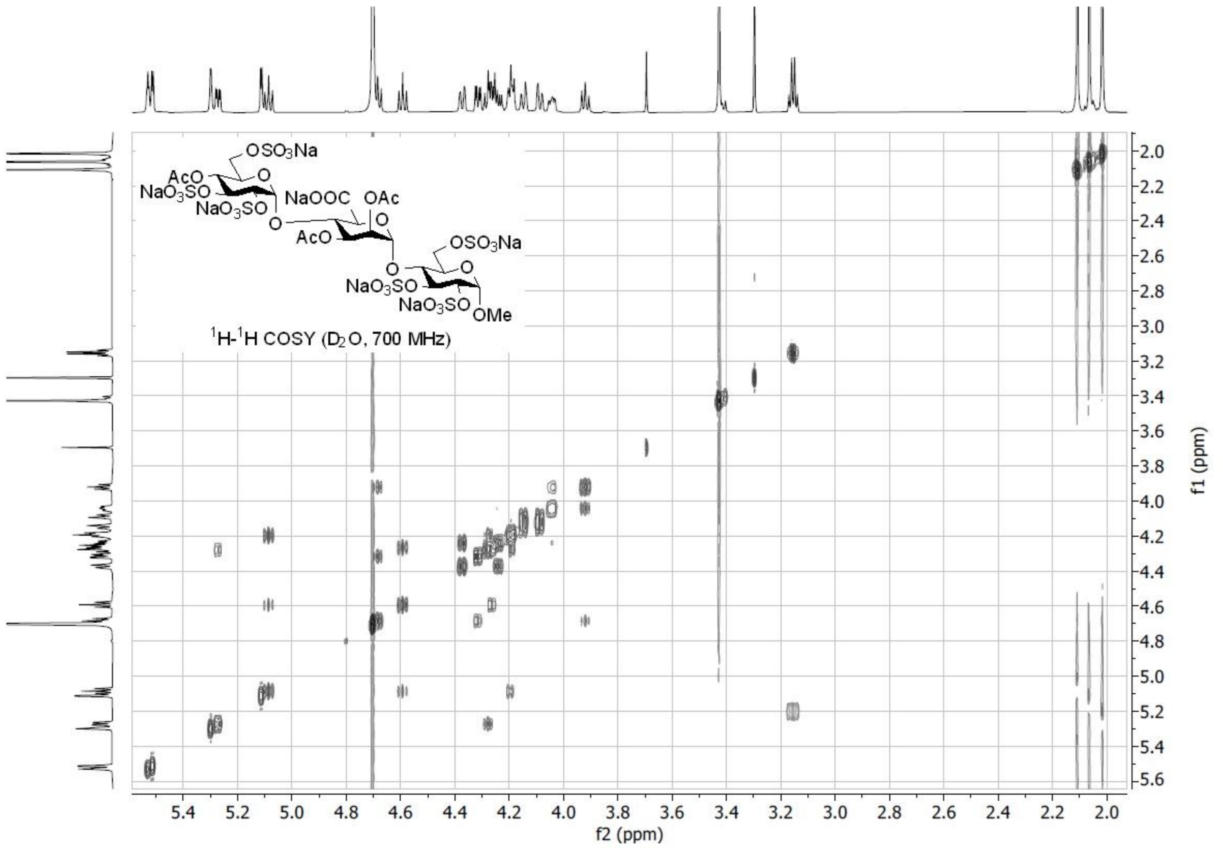
OSO3Na
AcO
NaO3SO
NaO3SO
NaOOC
OAc
AcO
OSO3Na
NaO3SO
NaO3SO
OMe
1H-1H COSY (D2O, 700 MHz)
f2 (ppm)
f1 (ppm)

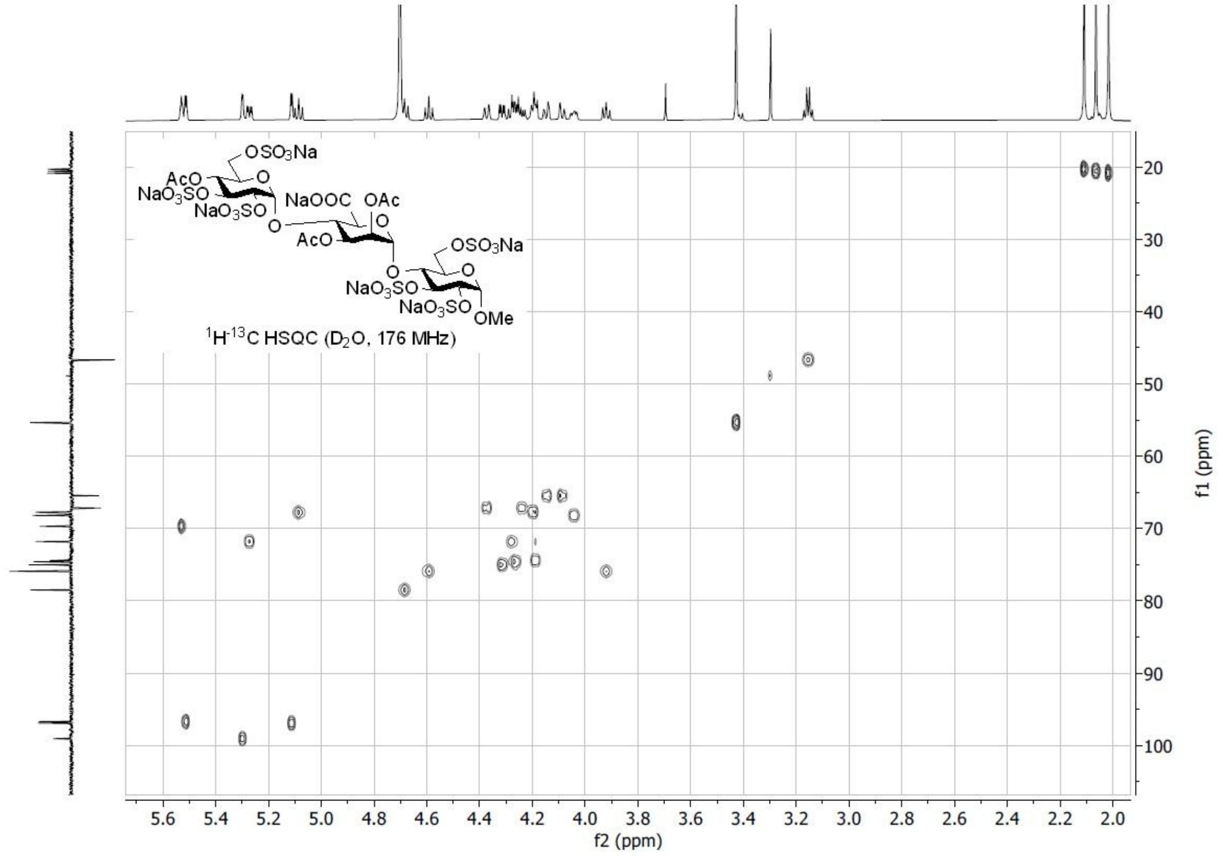
OSO3Na
AcO
NaO3SO
NaO3SO
NaOOC
OAc
AcO
OSO3Na
NaO3SO
NaO3SO
OMe
1H-13C HSQC (D2O, 176 MHz)
f2 (ppm)
f1 (ppm)

^1H, ^{13}C, COSY and HSQC NMR spectra of compound **15**

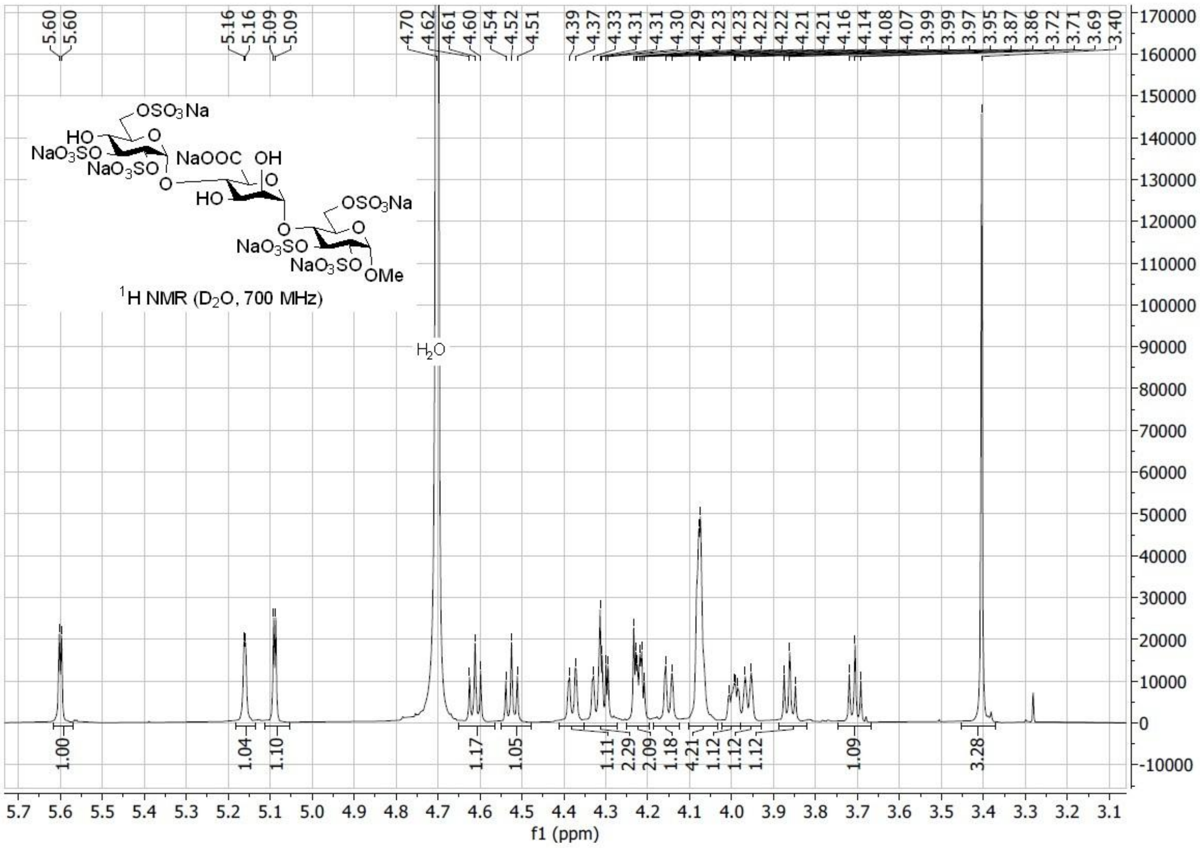

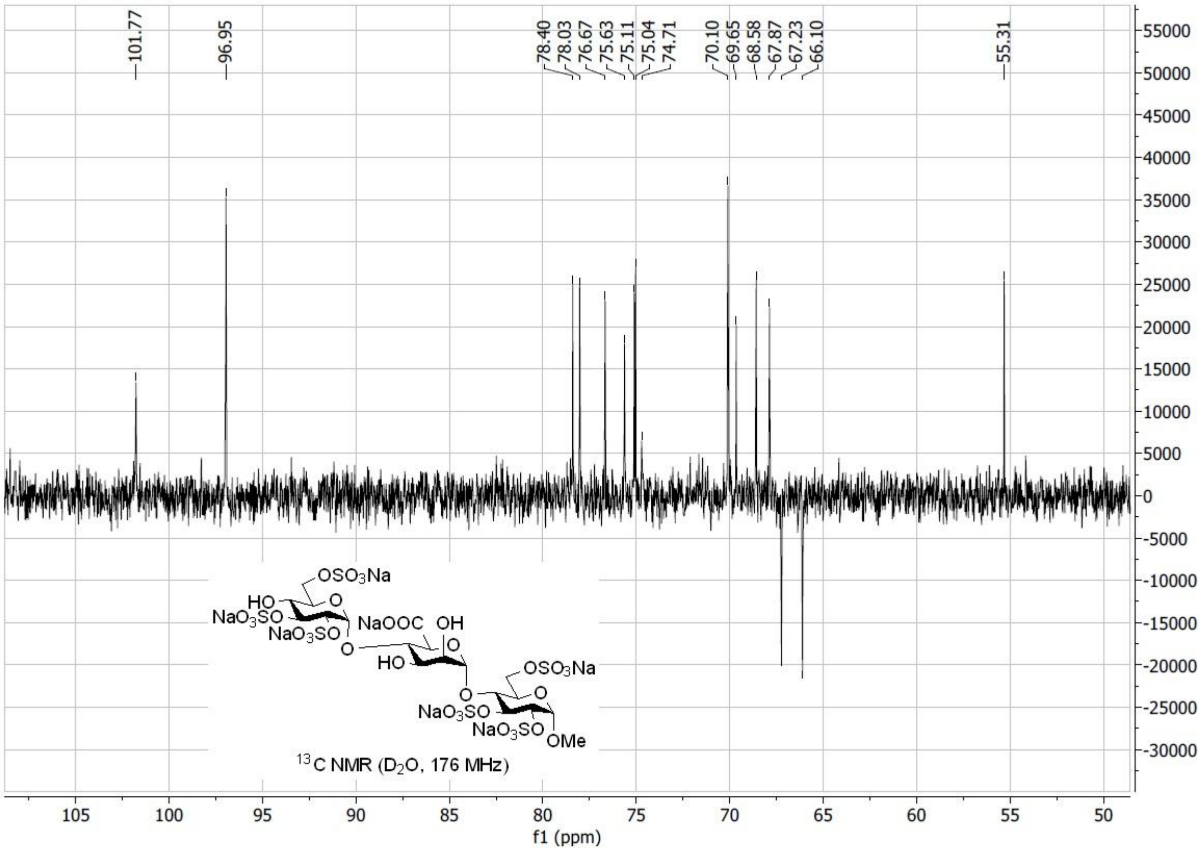

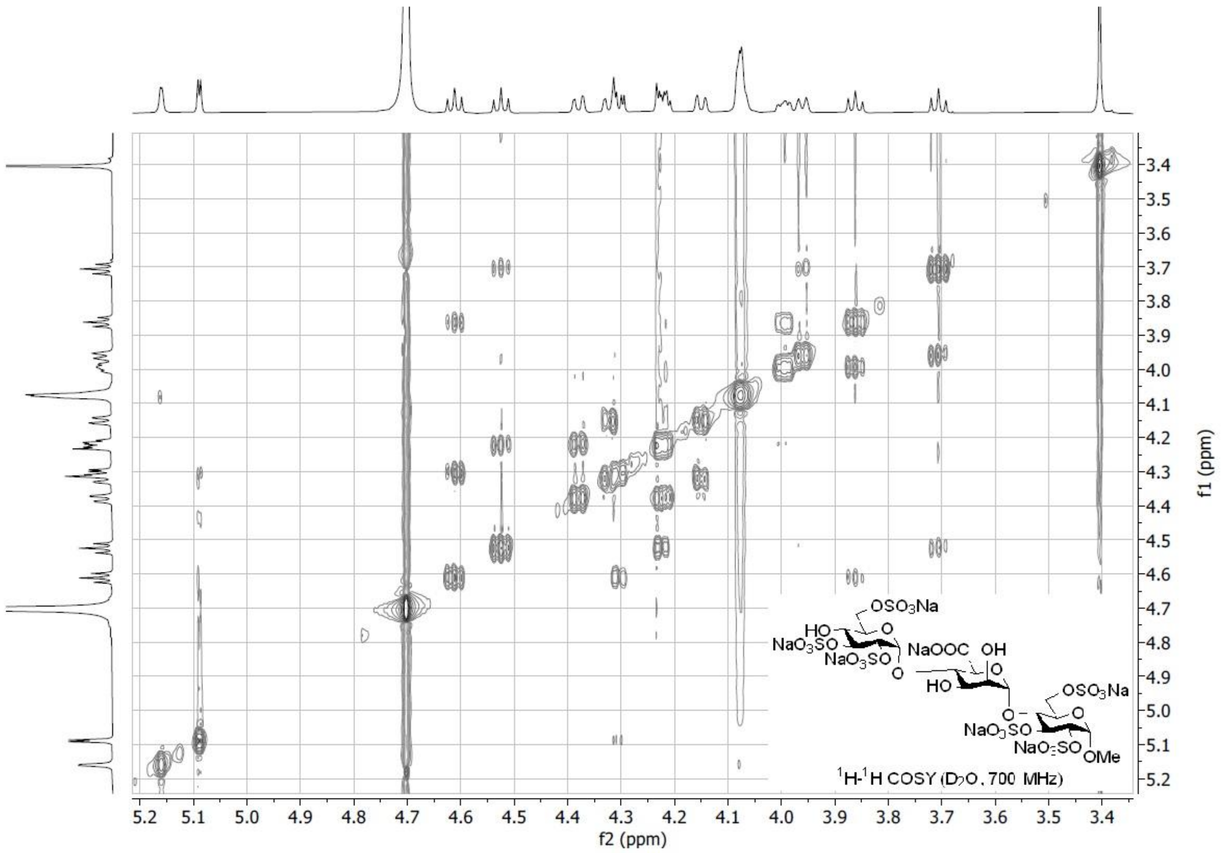

OSO3Na
HO
NaO3SO
NaO3SO
NaOOC OH
HO
OSO3Na
NaO3SO
NaO3SO
OMe
1H-1H COSY (D2O, 700 MHz)
f2 (ppm)
f1 (ppm)

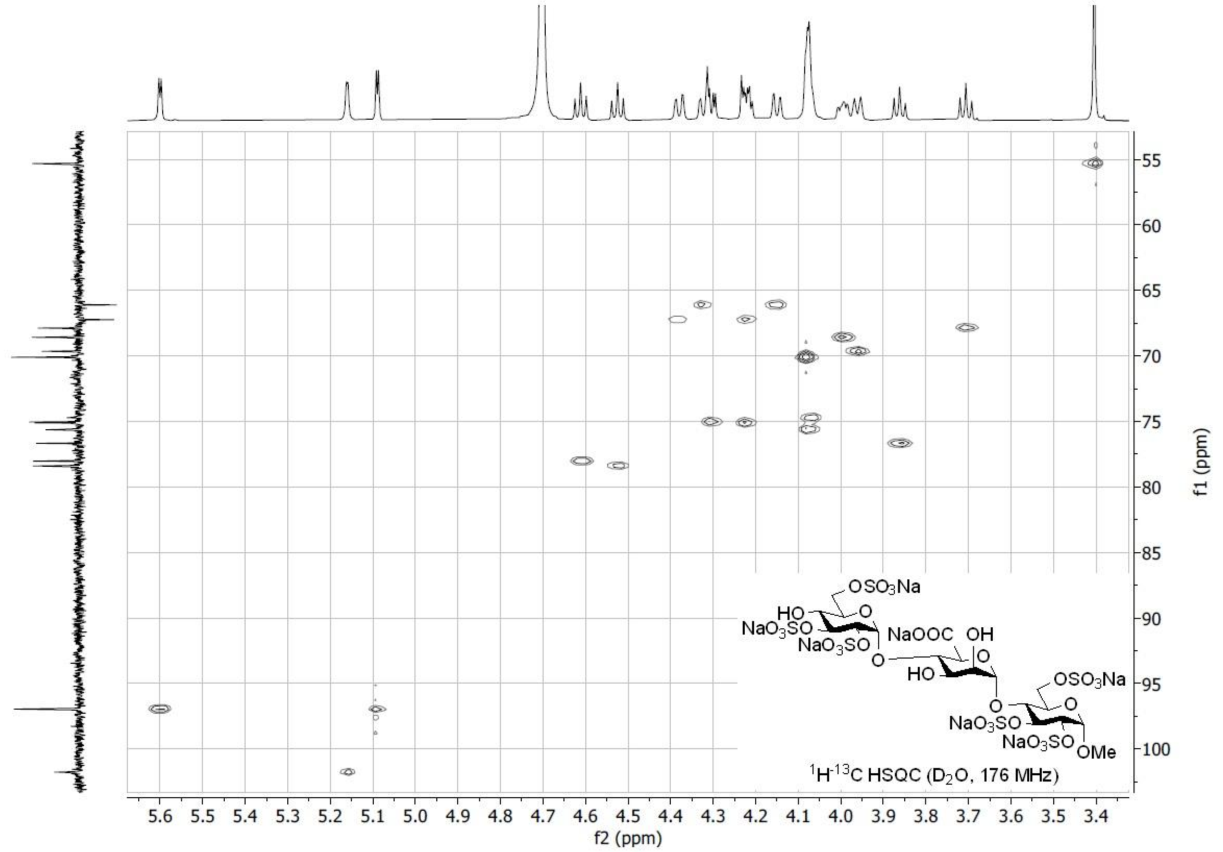

OSO3Na
HO
NaO3SO
NaO3SO
NaOOC OH
HO
OSO3Na
NaO3SO
NaO3SO
OMe
1H-13C HSQC (D2O, 176 MHz)
f2 (ppm)
f1 (ppm)

^{1}H, ^{13}C, COSY and HSQC NMR spectra of compound **17**

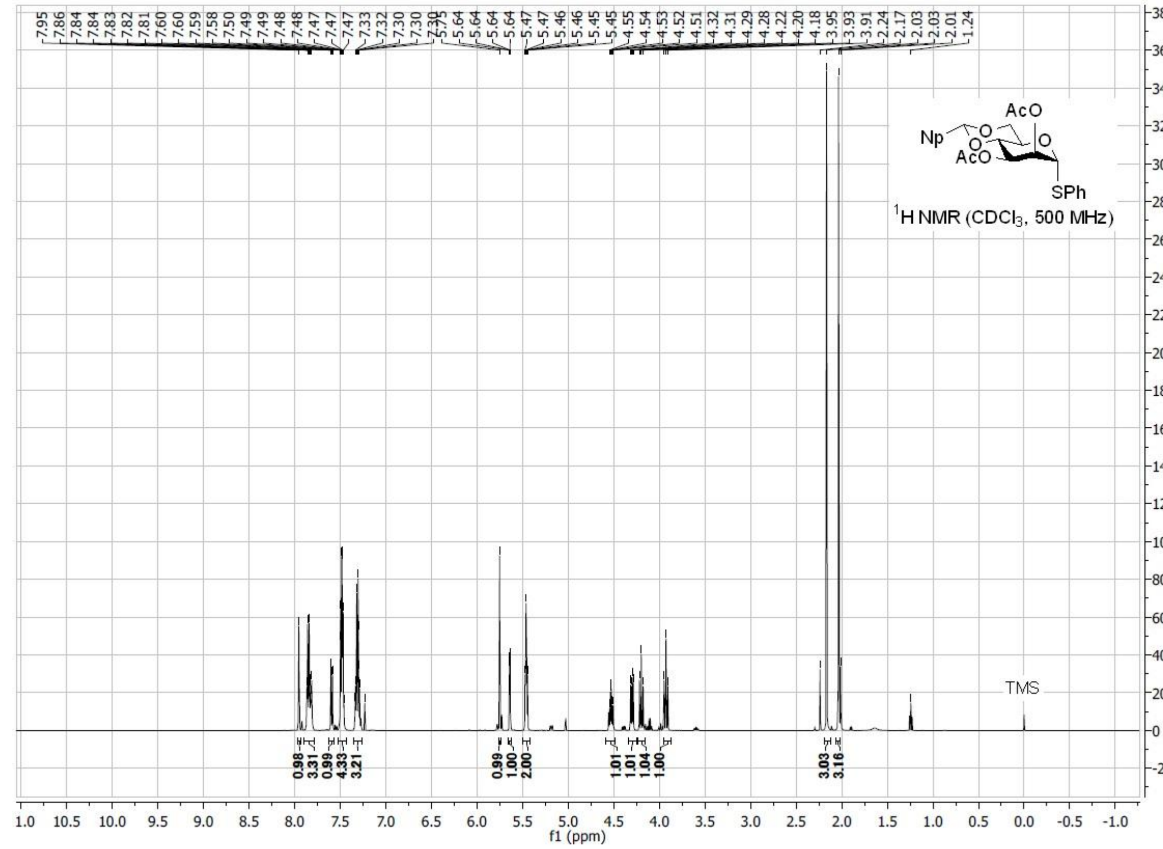

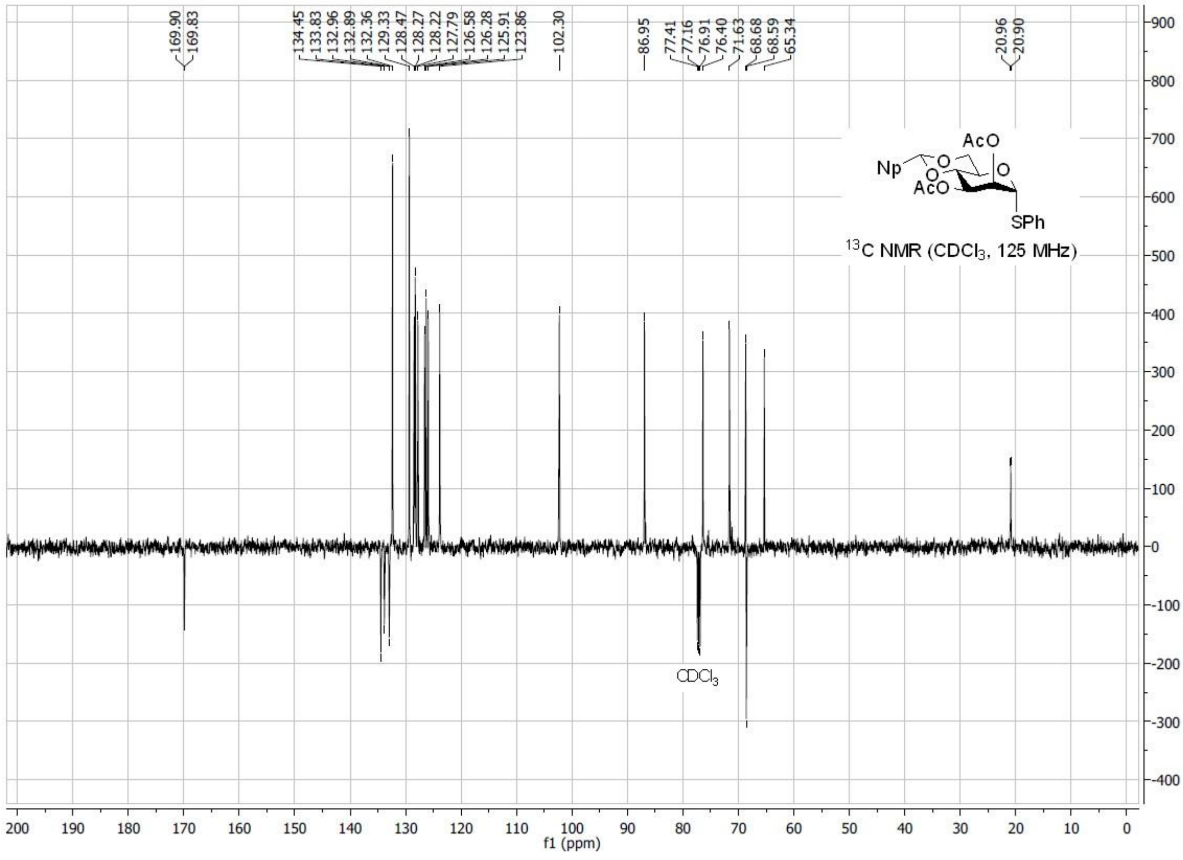

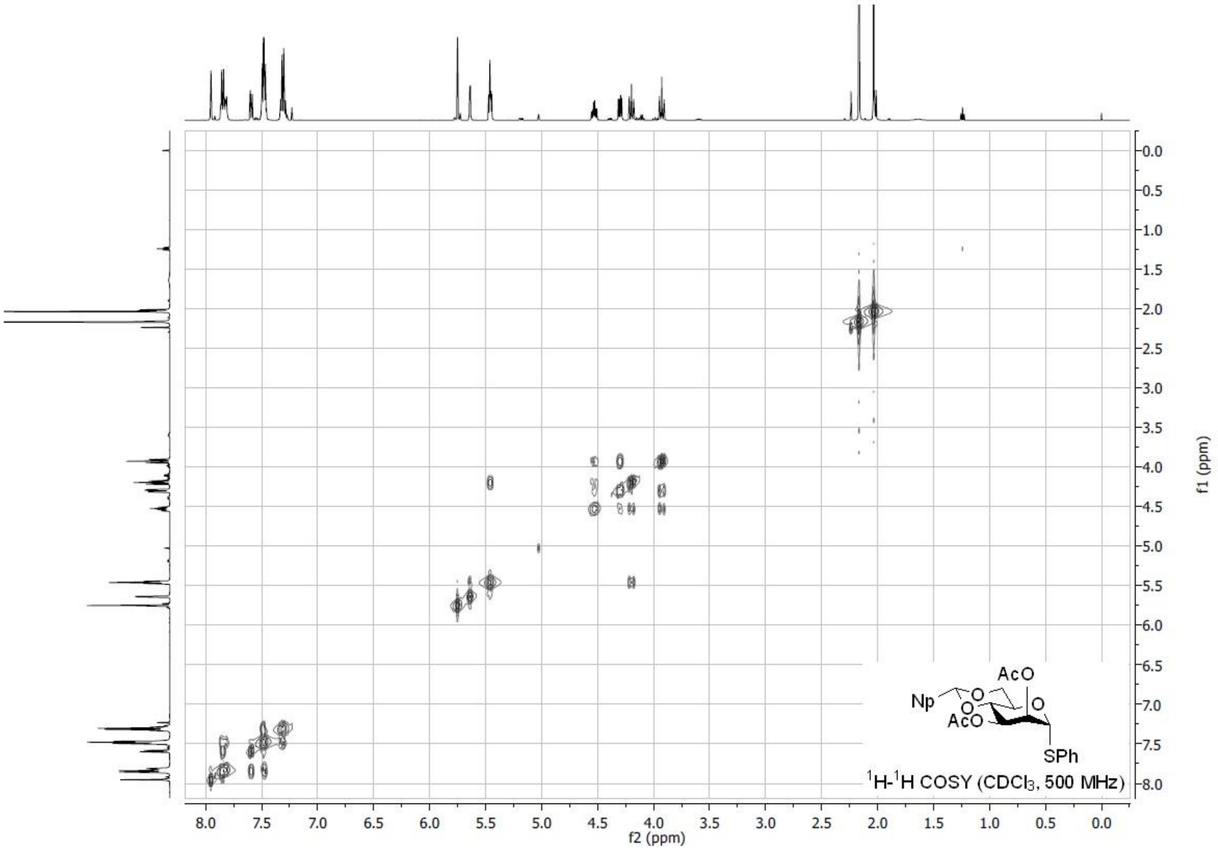
AcO
Np
O
O
AcO
O
SPh
1H-1H COSY (CDCl3, 500 MHz)
f2 (ppm)
f1 (ppm)

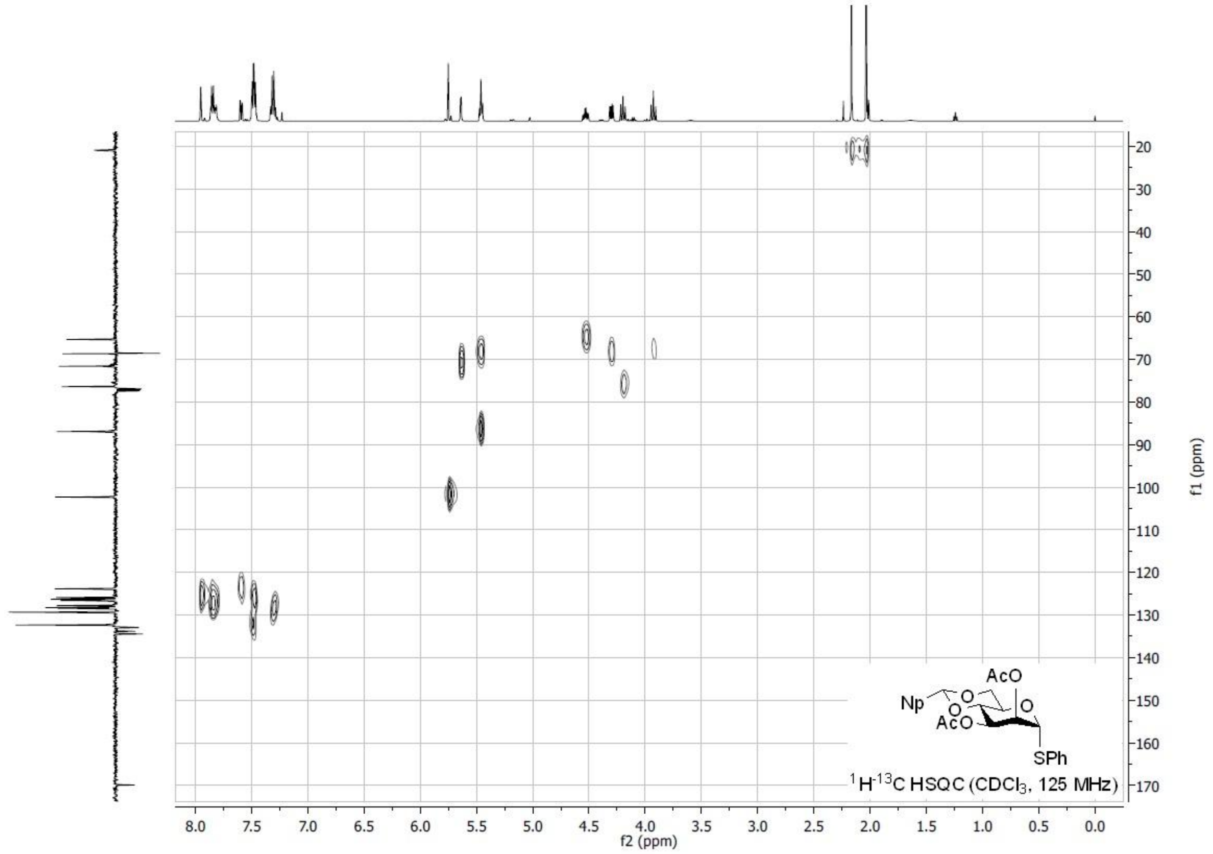
AcO
Np
O
O
AcO
O
SPh
1H-13C HSQC (CDCl3, 125 MHz)
f2 (ppm)
f1 (ppm)

^{1}H, ^{13}C, COSY and HSQC NMR spectra of compound **18**

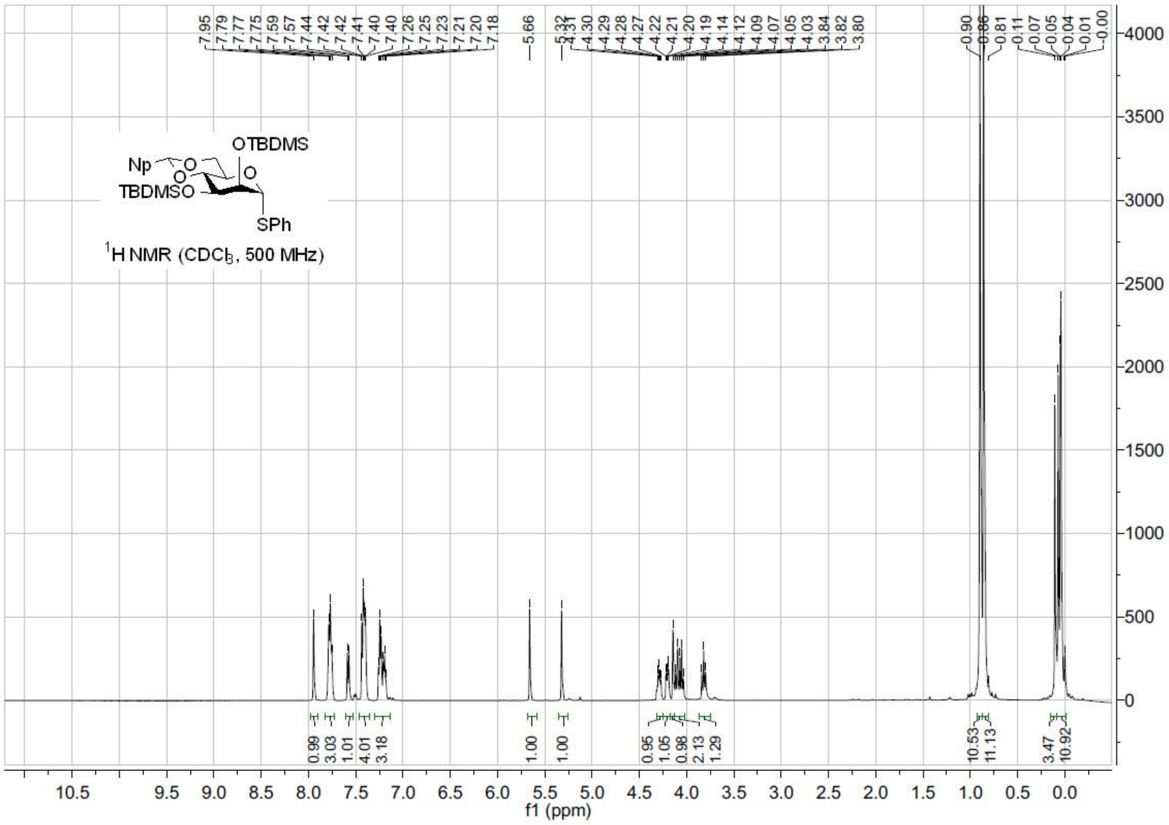

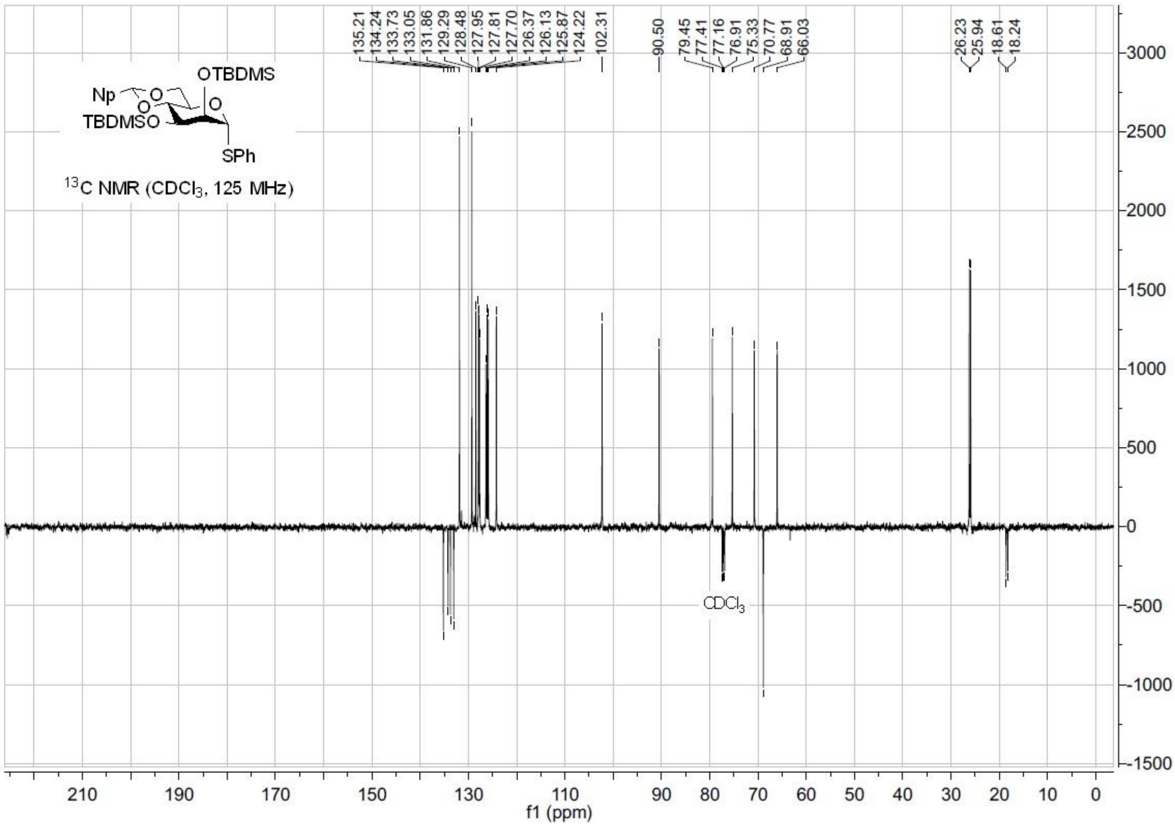

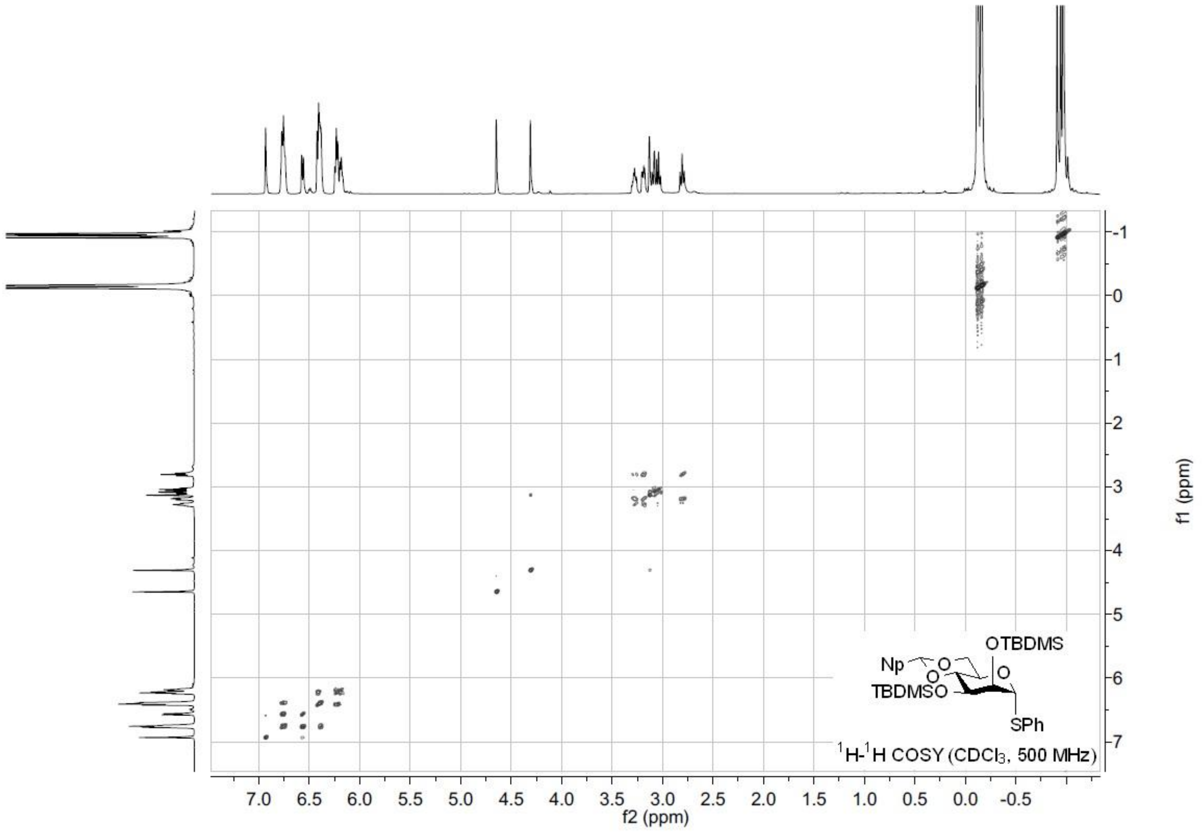
OTBDMS
Np
O
O
O
TBDMSO
SPh
^{1}H-^{1}H COSY (CDCl$_3$, 500 MHz)
f2 (ppm)
f1 (ppm)

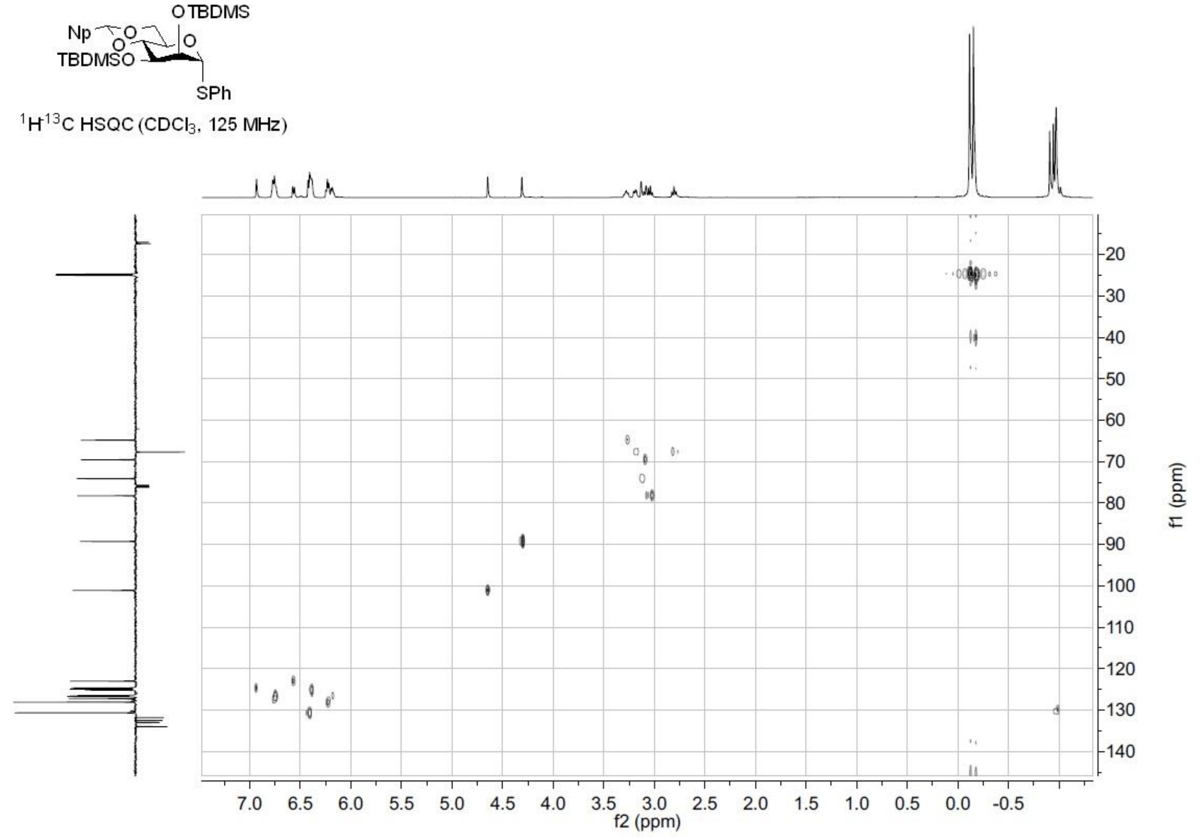
OTBDMS
Np
O
O
O
TBDMSO
SPh
^{1}H-^{13}C HSQC (CDCl$_3$, 125 MHz)
f2 (ppm)
f1 (ppm)

^{1}H, ^{13}C, COSY and HSQC NMR spectra of compound **20**

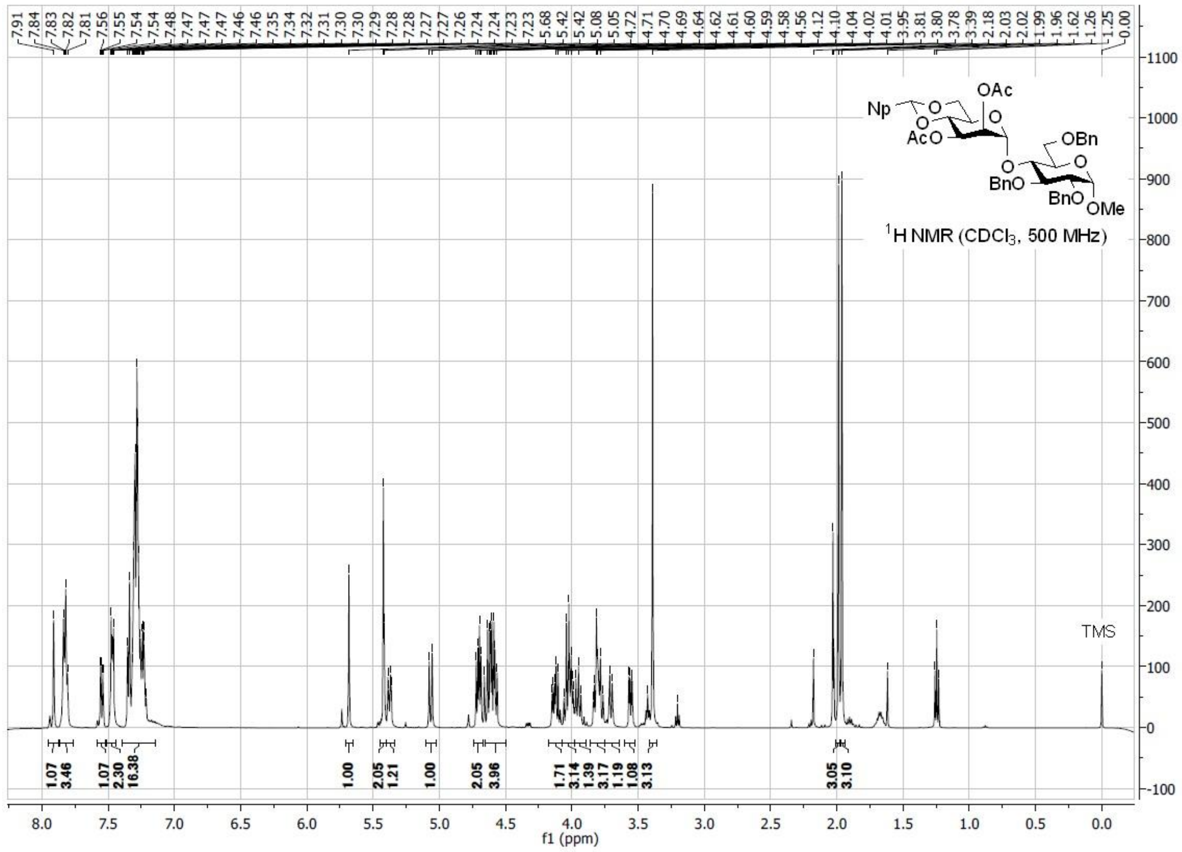

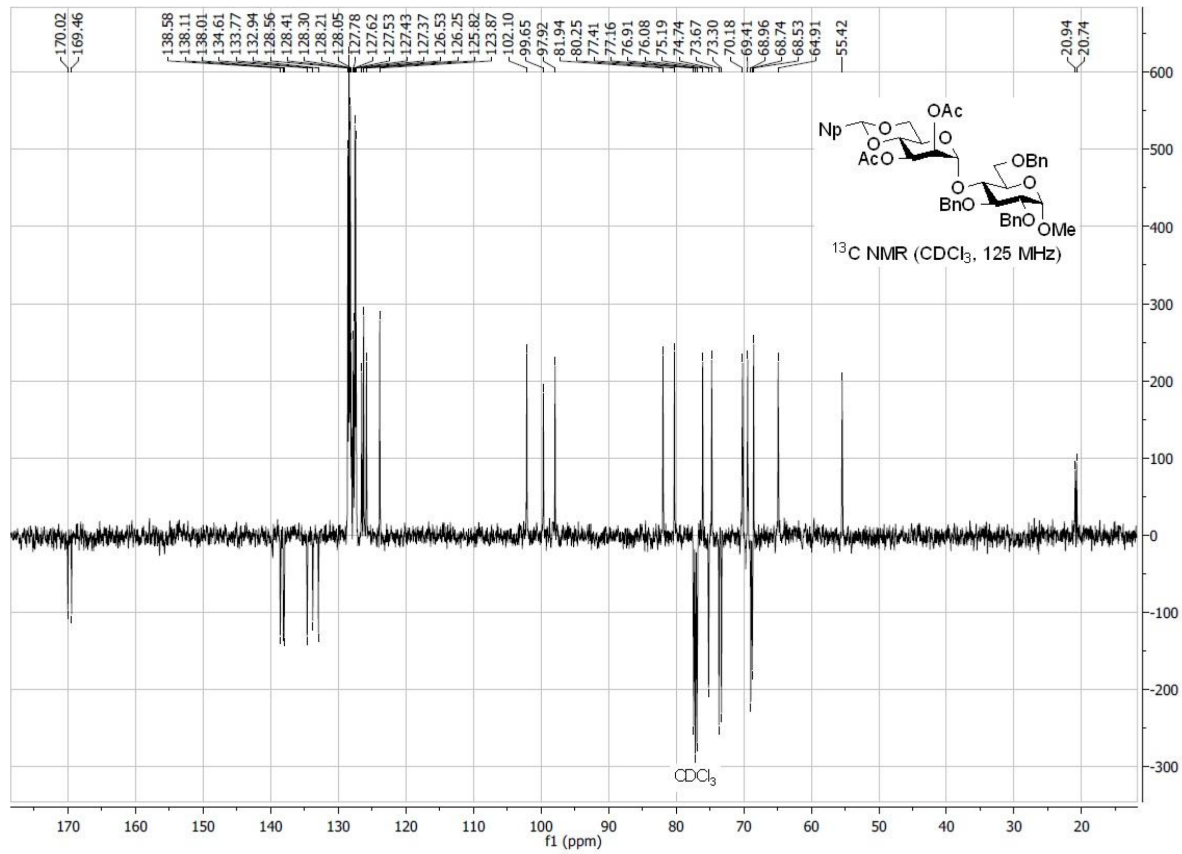

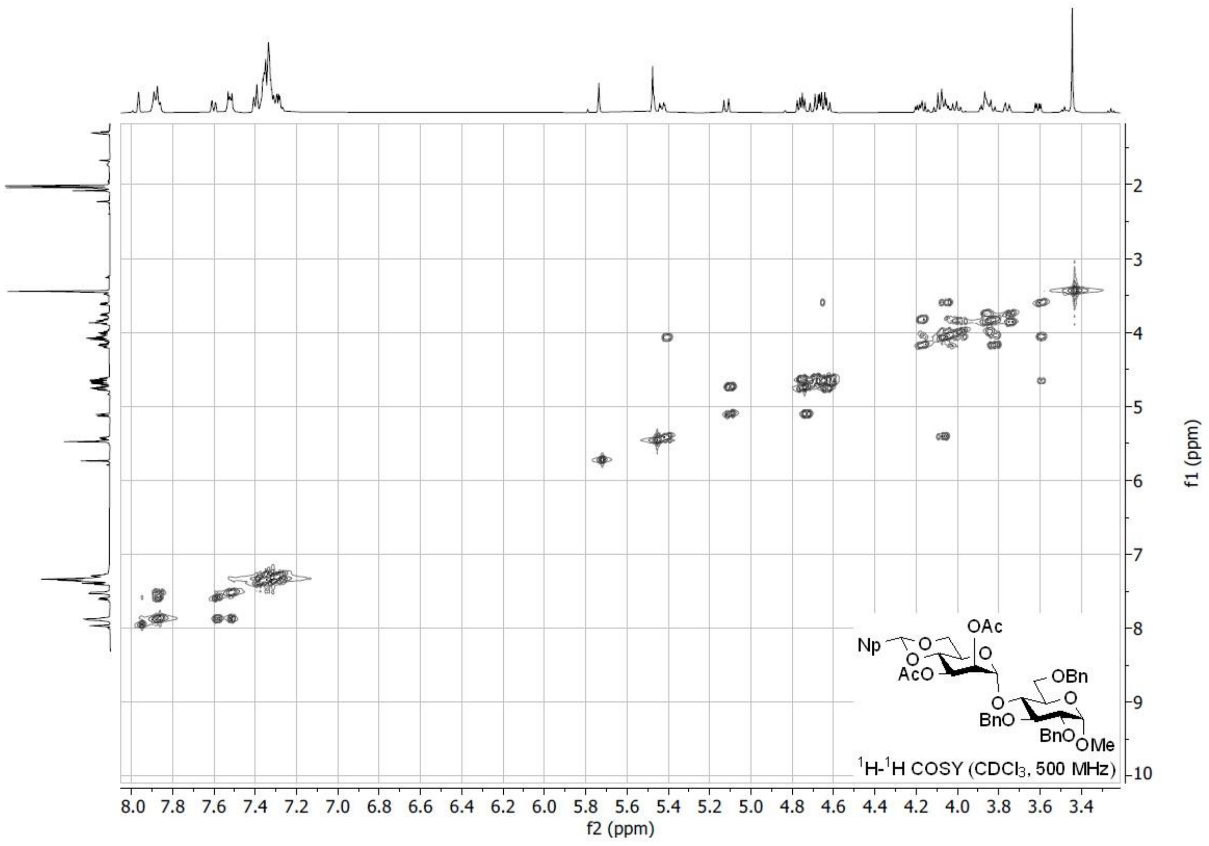
Np
OAc
AcO
OBn
BnO
BnO
OMe
^{1}H-^{1}H COSY (CDCl$_3$, 500 MHz)
f2 (ppm)
f1 (ppm)

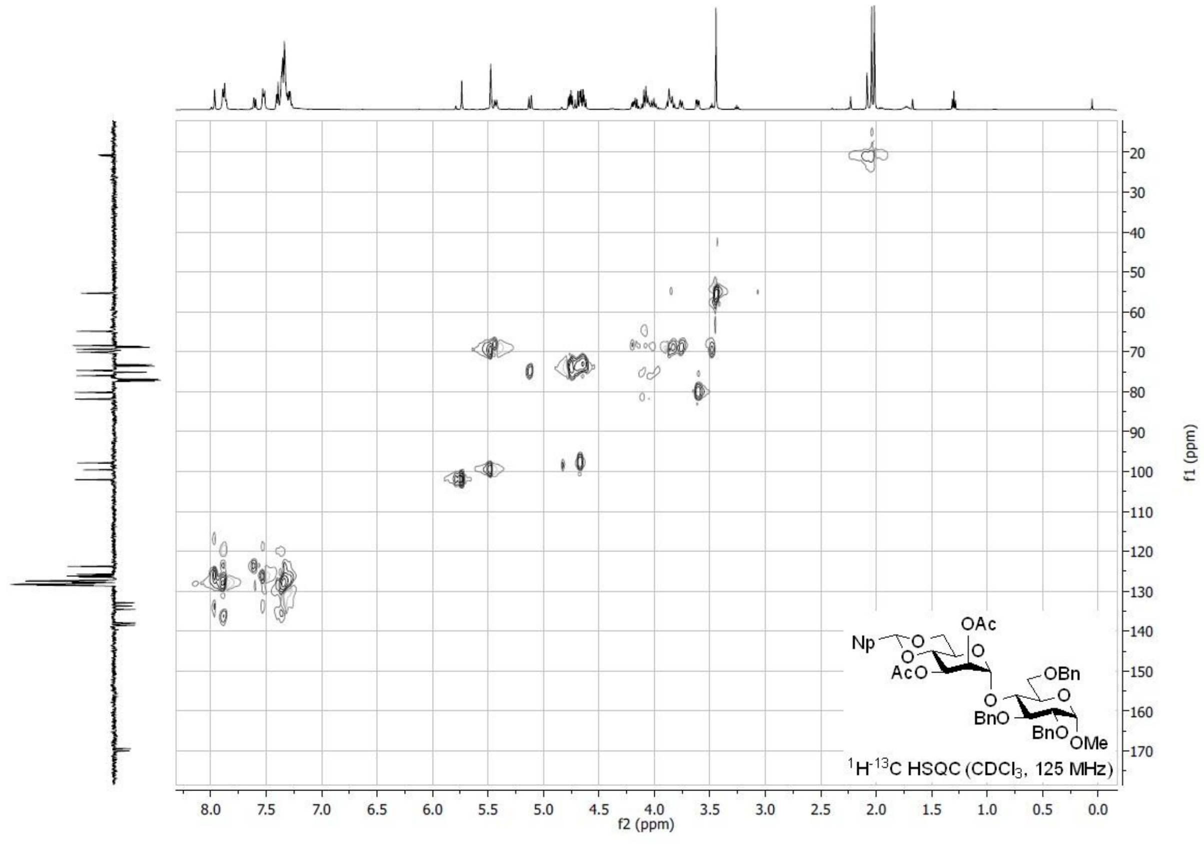
Np
OAc
AcO
OBn
BnO
BnO
OMe
^{1}H-^{13}C HSQC (CDCl$_3$, 125 MHz)
f2 (ppm)
f1 (ppm)

^{1}H, ^{13}C, COSY and HSQC NMR spectra of compound **21**

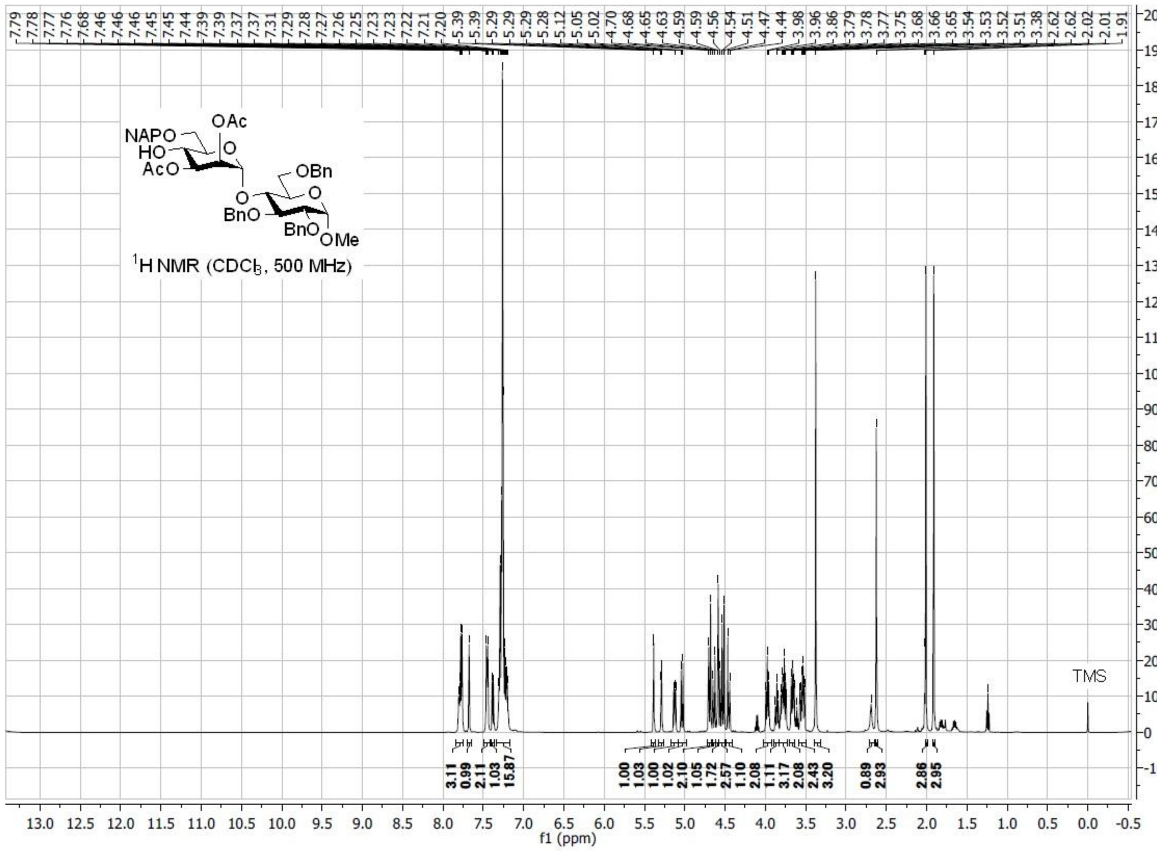

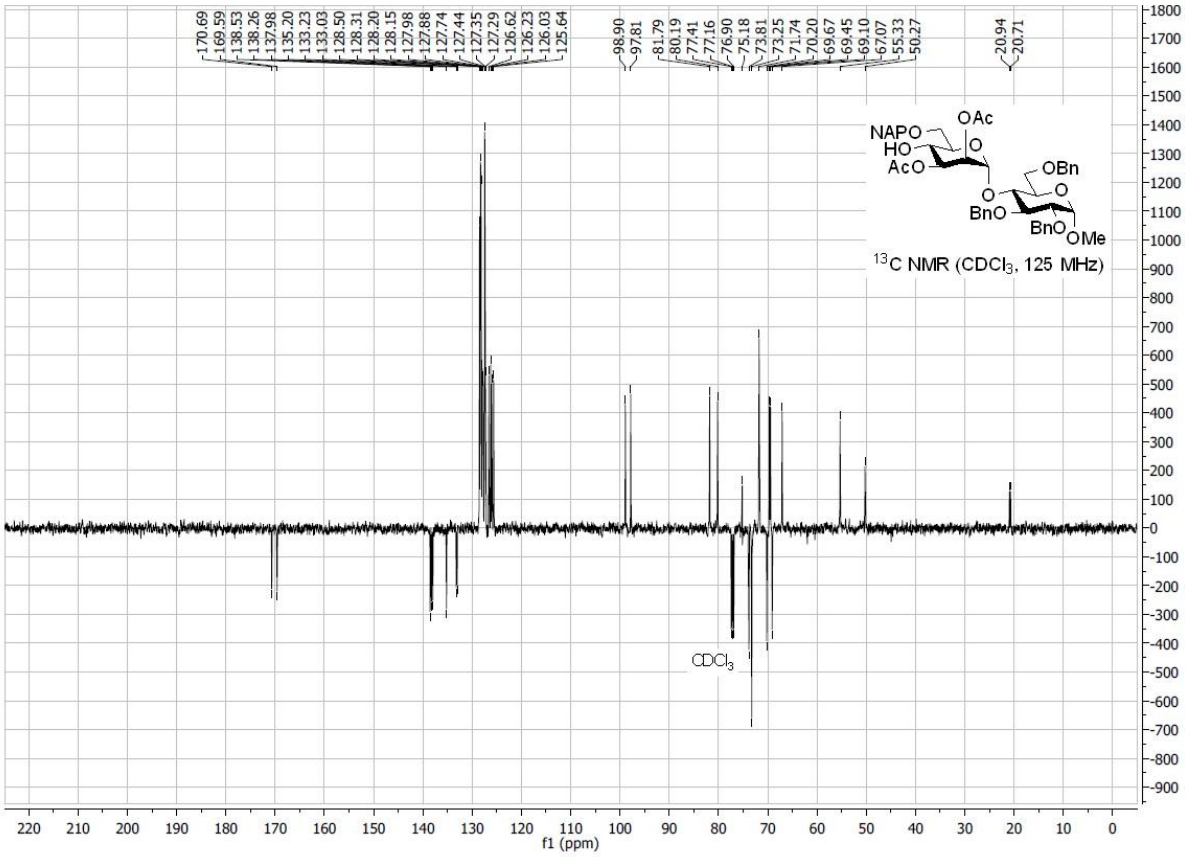

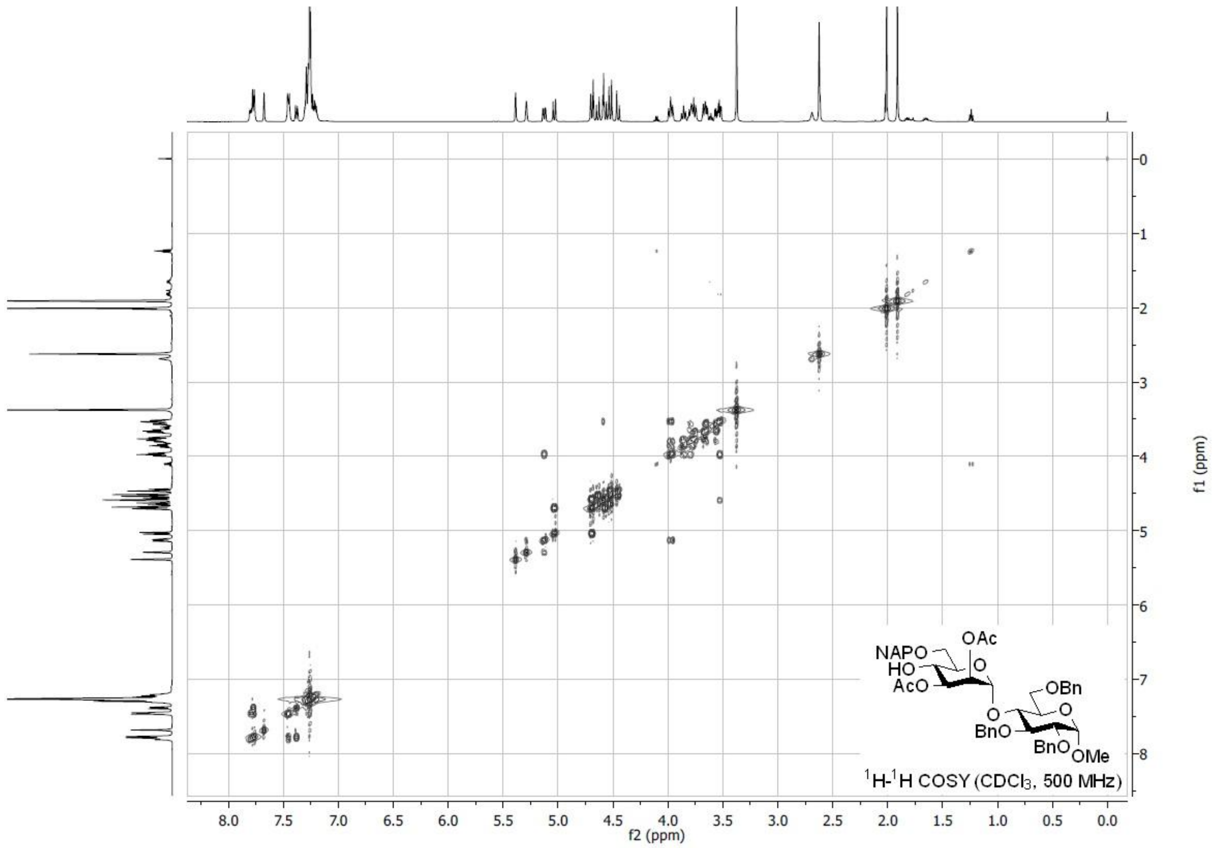
NAPO
HO
AcO
OAc
O
O
OBn
O
BnO
BnO
OMe
^{1}H-^{1}H COSY (CDCl$_3$, 500 MHz)
f2 (ppm)
f1 (ppm)

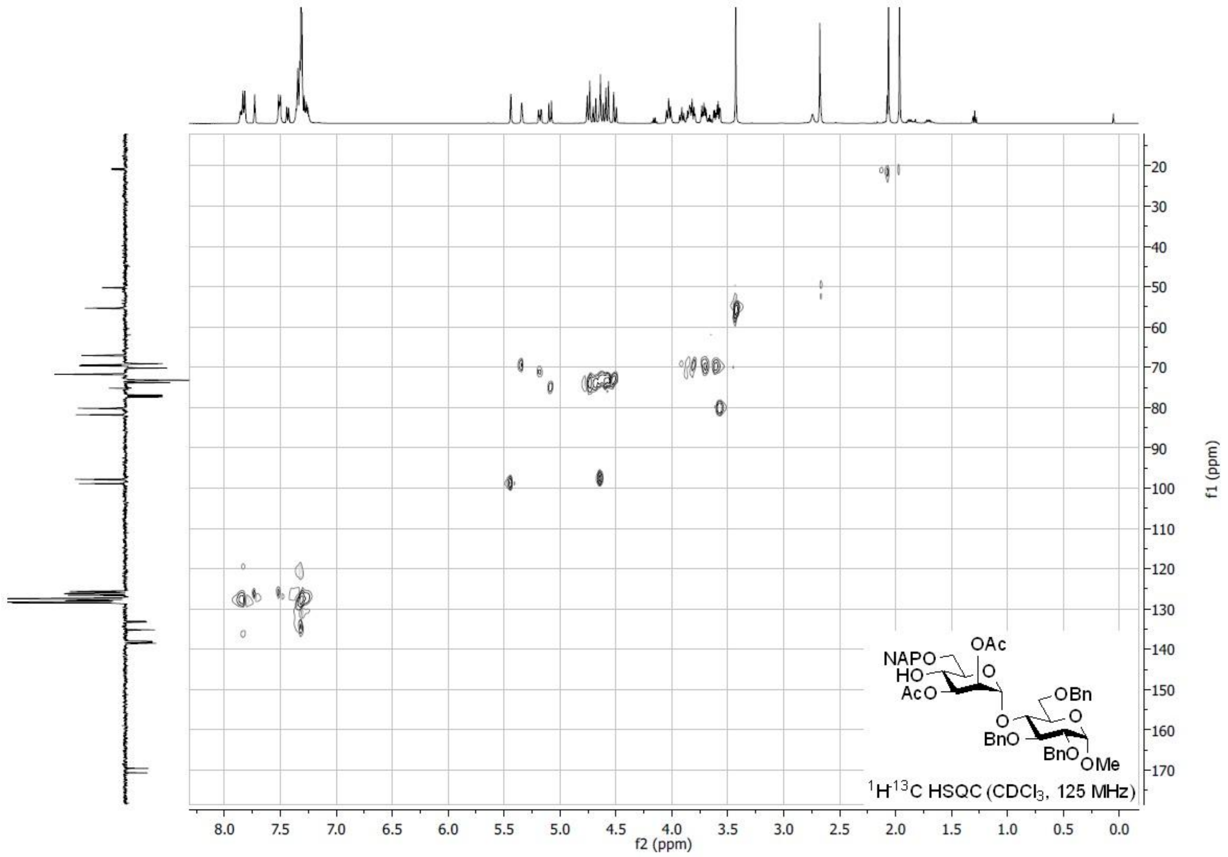
NAPO
HO
AcO
OAc
O
O
OBn
O
BnO
BnO
OMe
^{1}H-^{13}C HSQC (CDCl$_3$, 125 MHz)
f2 (ppm)
f1 (ppm)

^1H, ^{13}C, COSY and HSQC NMR spectra of compound **22**

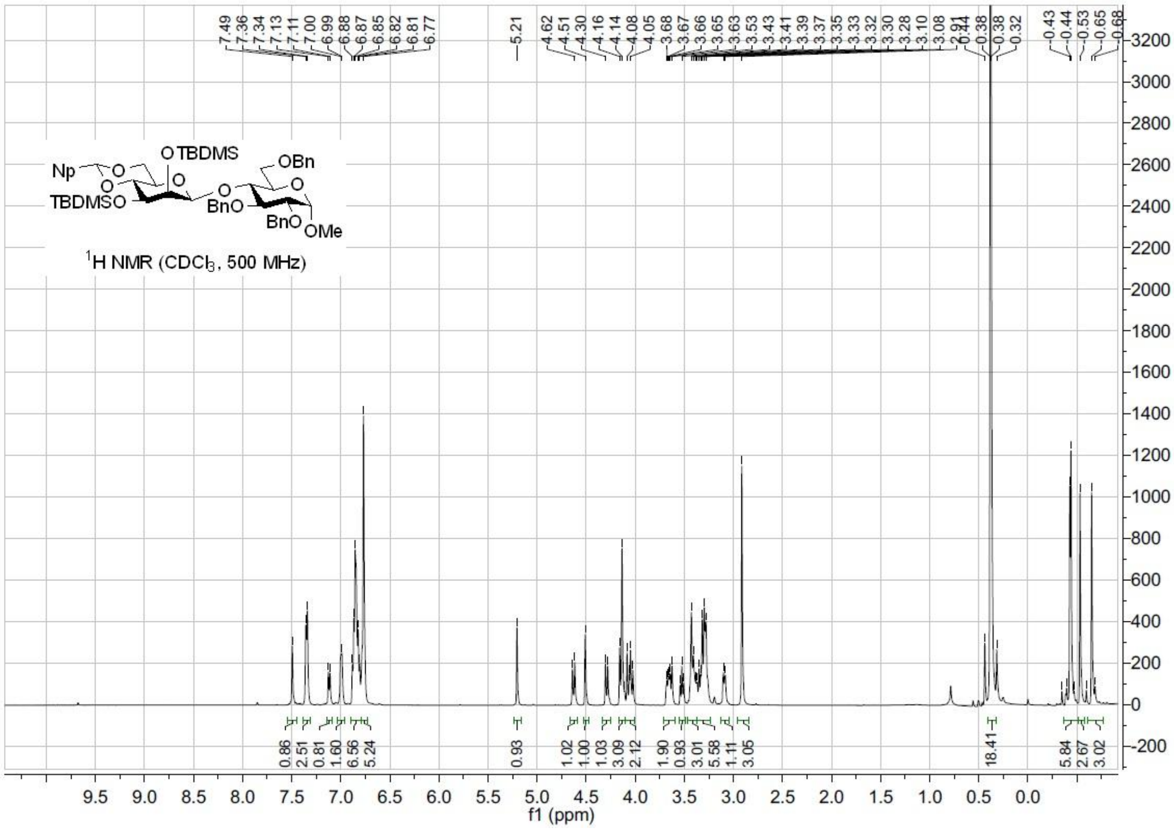

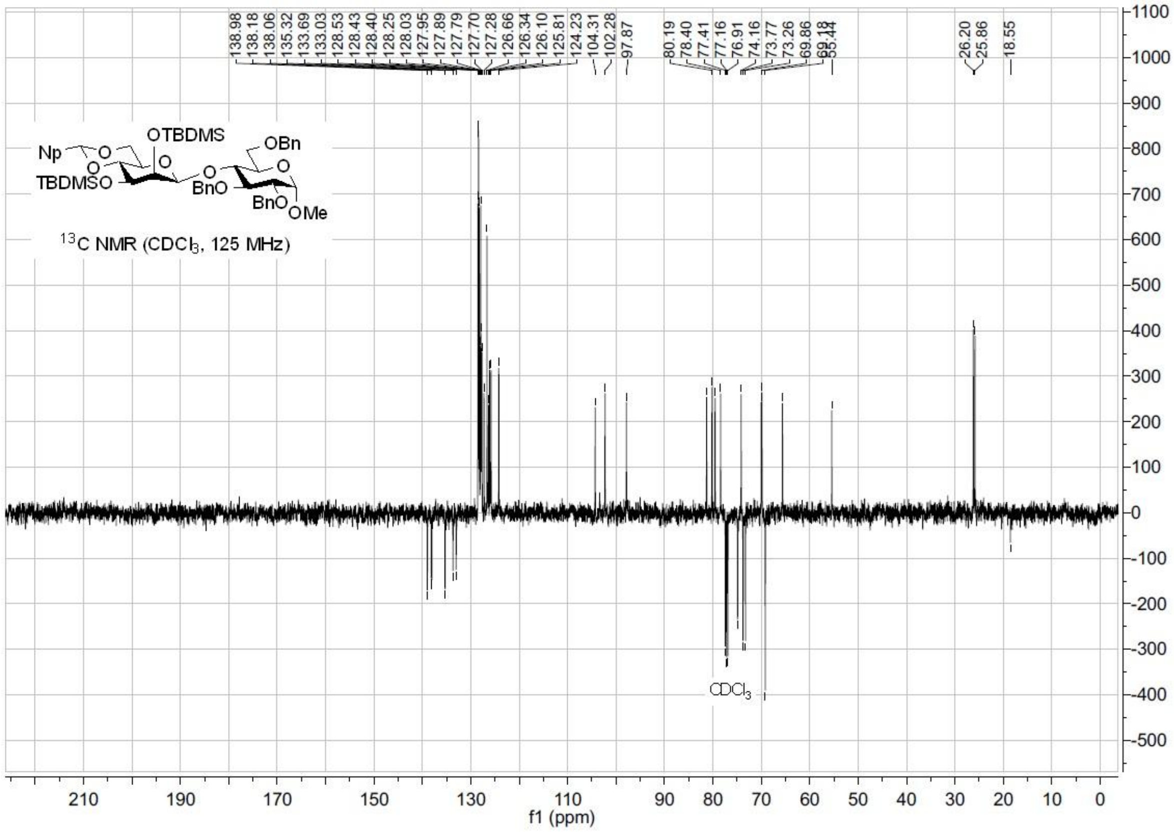

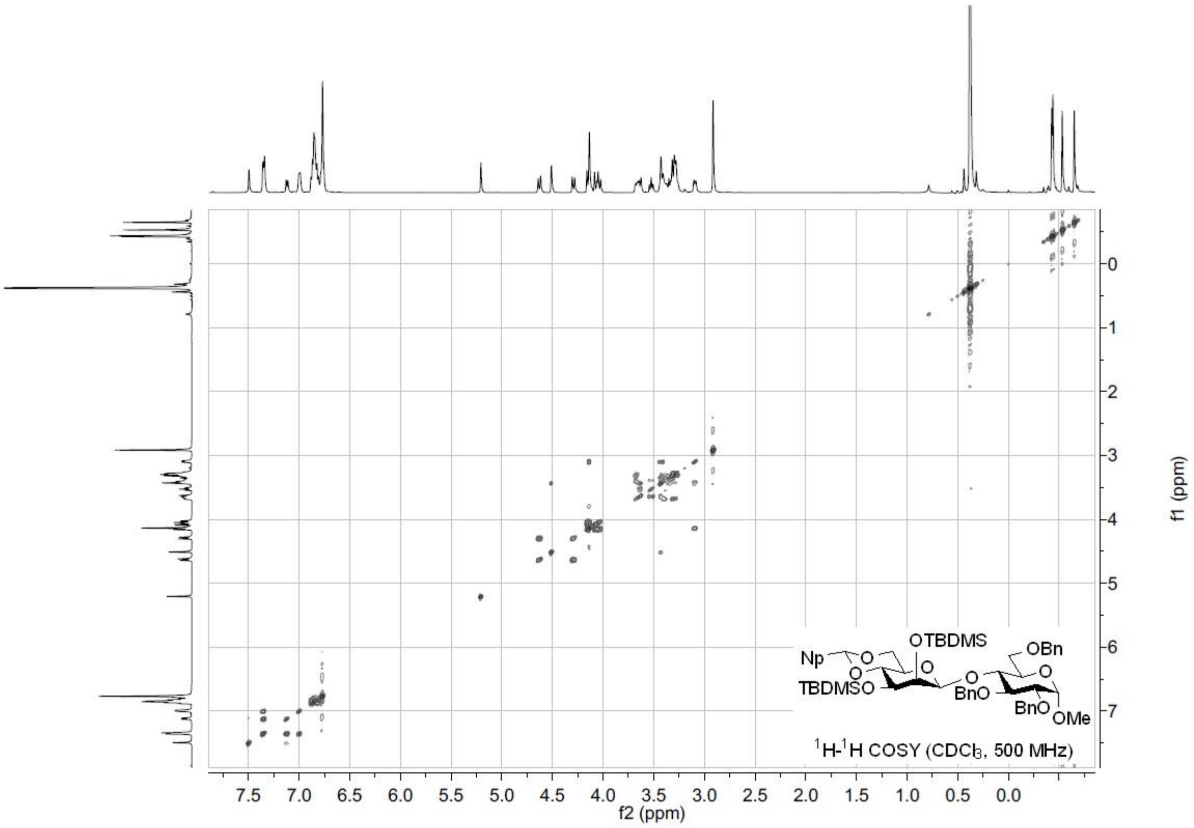
OTBDMS
Np
O
O
O
TBDMSO
O
BnO
OBn
O
BnO
OMe
1H-1H COSY (CDCl3, 500 MHz)
f1 (ppm)
f2 (ppm)

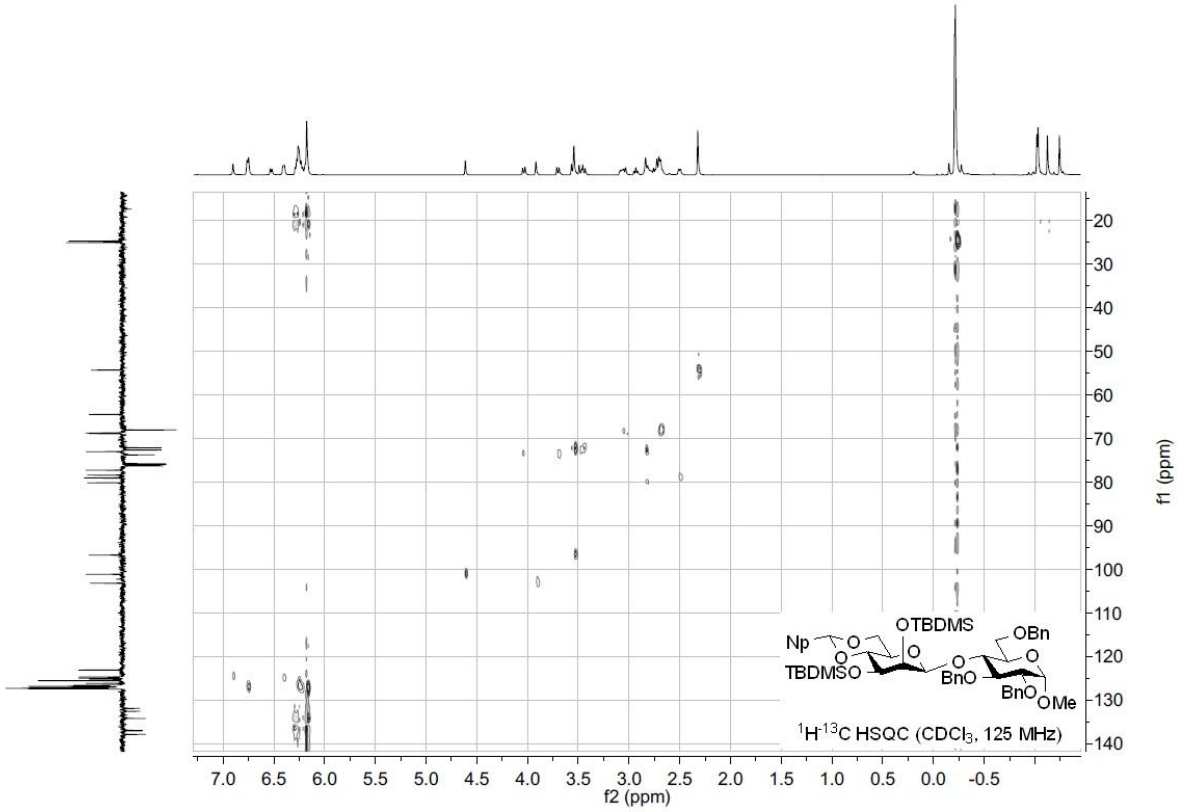
OTBDMS
Np
O
O
O
TBDMSO
O
BnO
OBn
O
BnO
OMe
1H-13C HSQC (CDCl3, 125 MHz)
f1 (ppm)
f2 (ppm)

^{1}H, ^{13}C, COSY, HMBC and HSQC NMR spectra of compound **23**

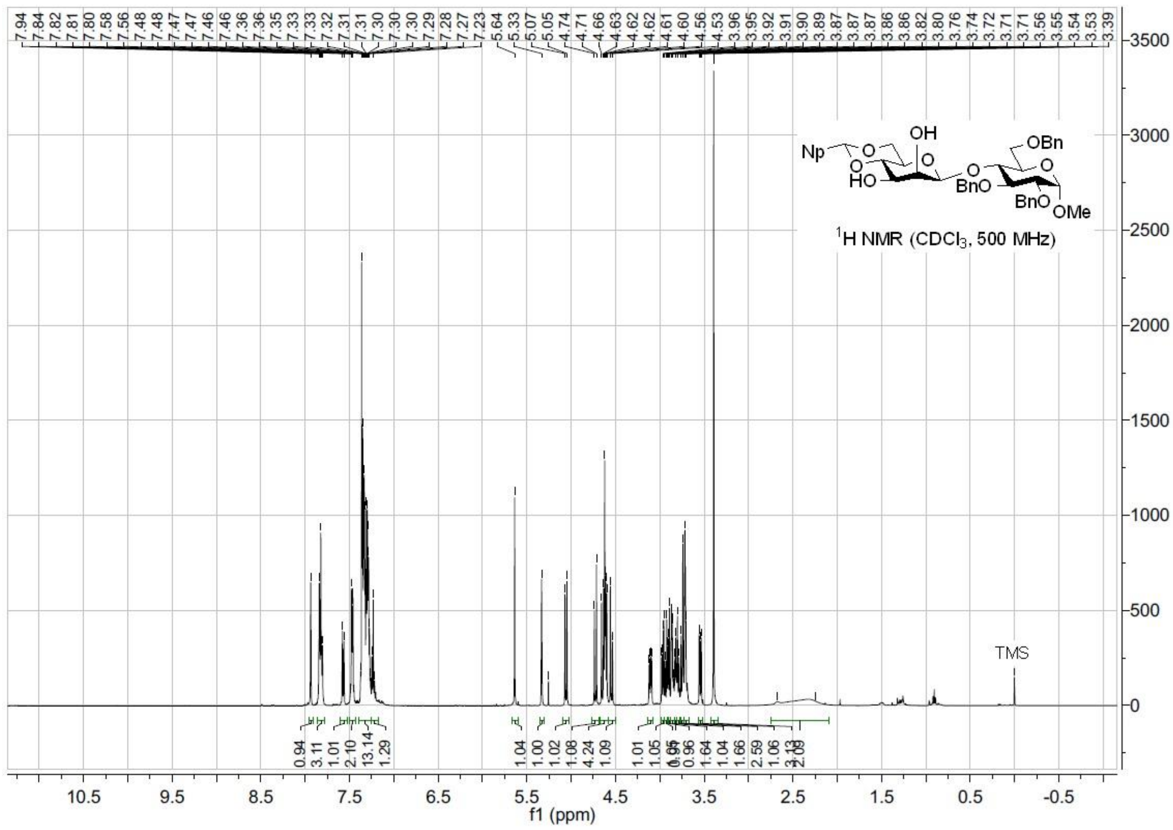

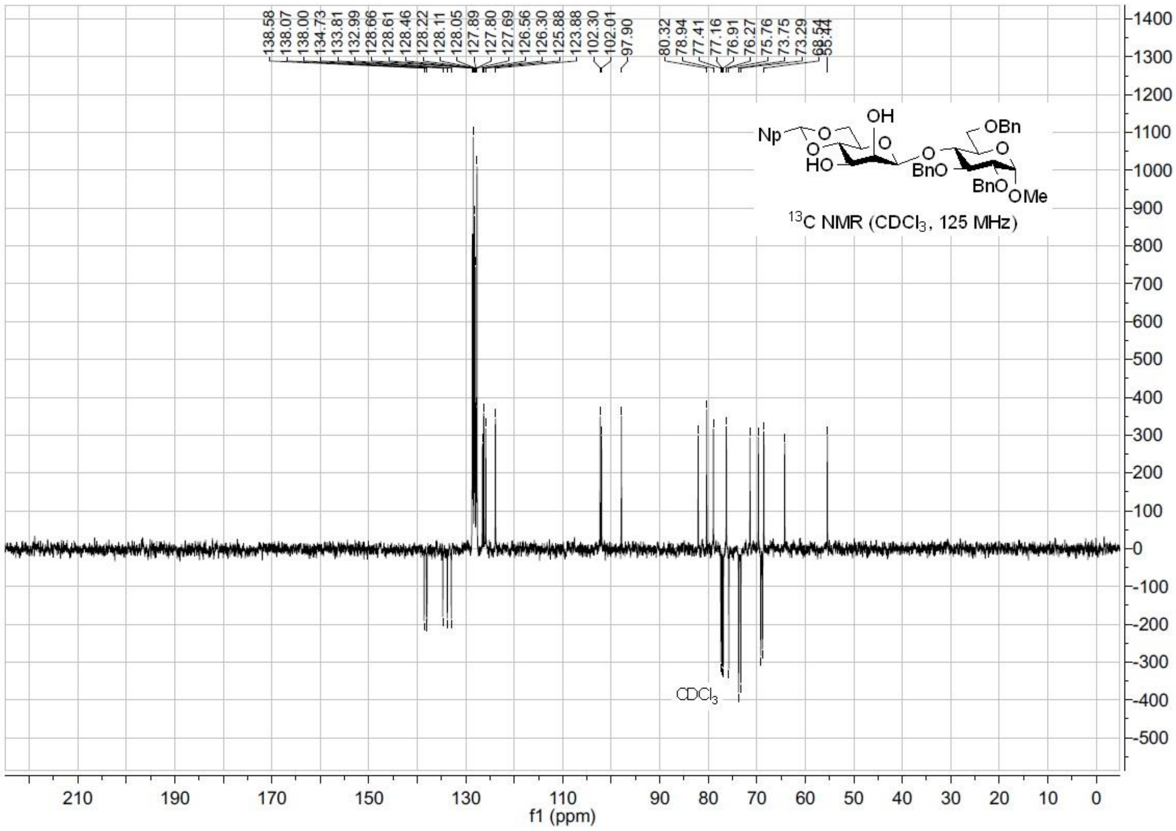

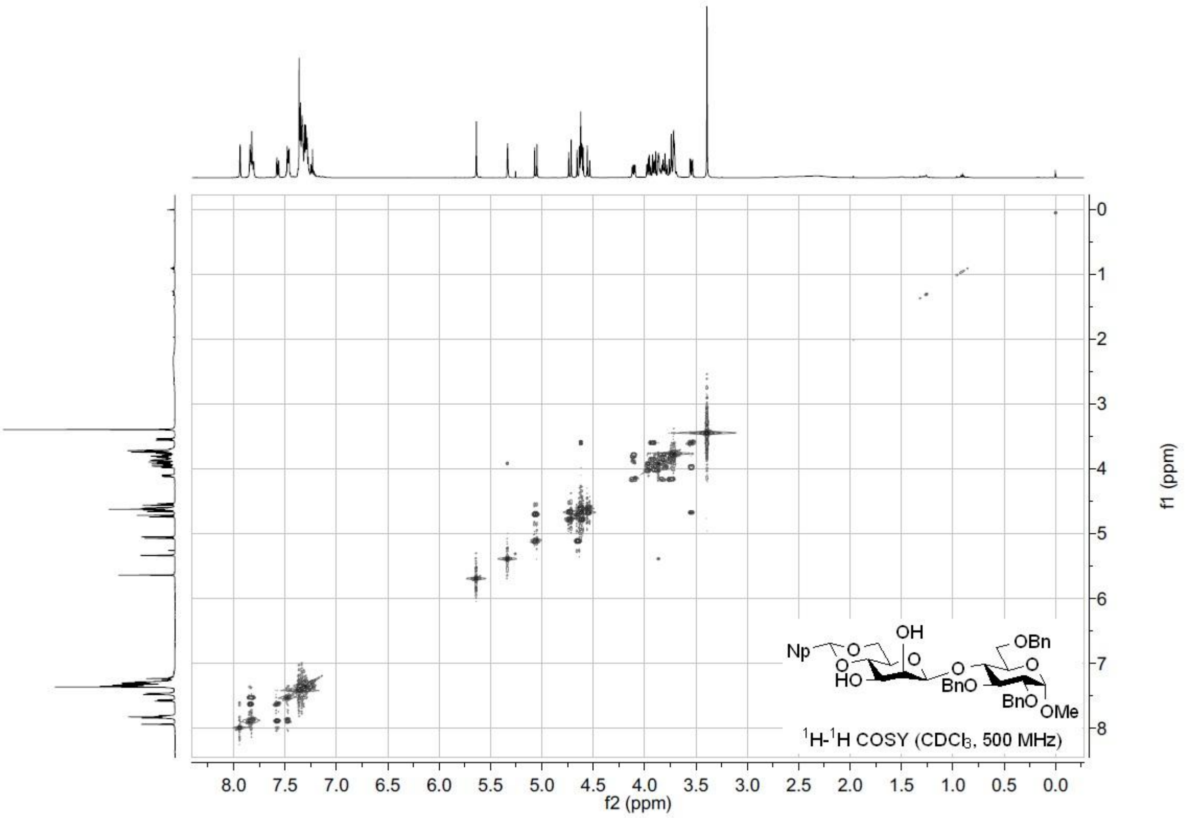
OH
Np
O
O
HO
O
O
BnO
OBn
O
BnO
OMe
^1H-^1H COSY ($CDCl_3$, 500 MHz)
f1 (ppm)
f2 (ppm)

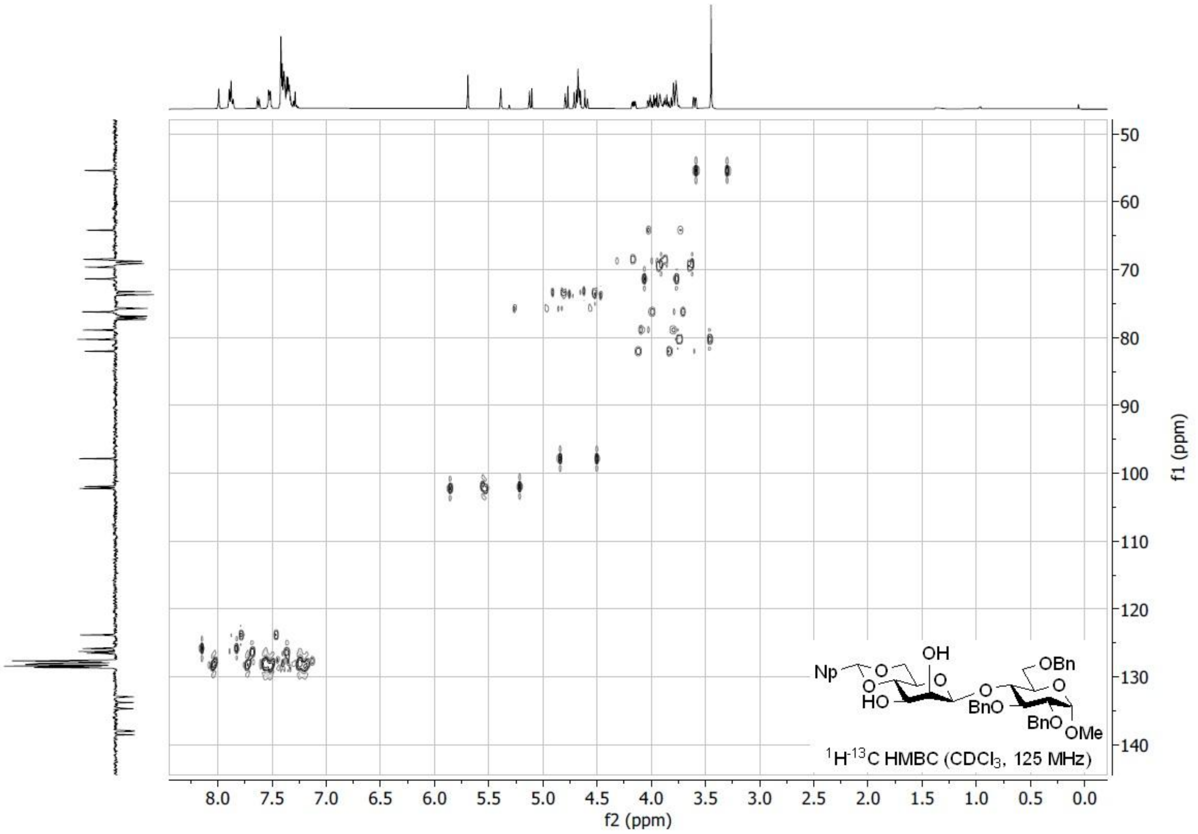
OH
Np
O
O
HO
O
O
BnO
OBn
O
BnO
OMe
^1H-^{13}C HMBC ($CDCl_3$, 125 MHz)
f1 (ppm)
f2 (ppm)

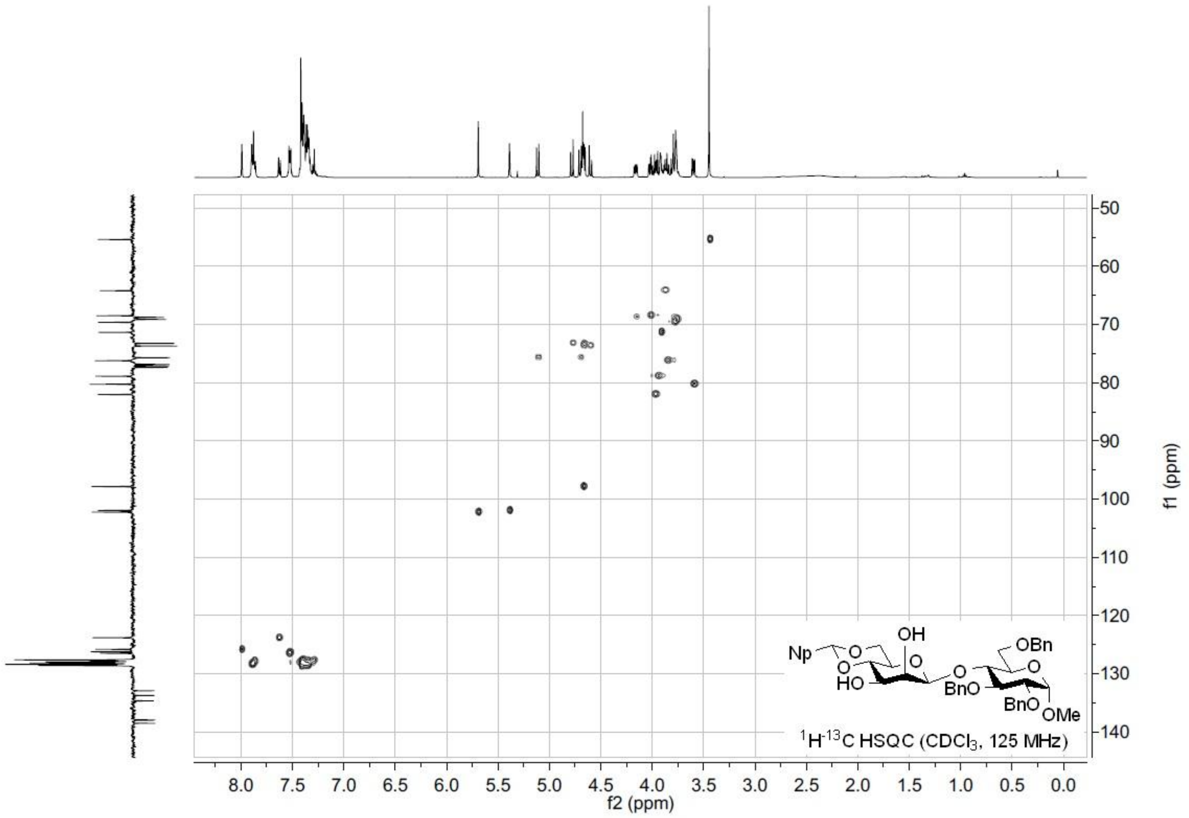

f1 (ppm)
f2 (ppm)
Np
OH
OBn
HO
BnO
BnO
OMe
^{1}H-^{13}C HSQC (CDCl$_3$, 125 MHz)

^{1}H, ^{13}C, COSY and HSQC NMR spectra of compound **24**

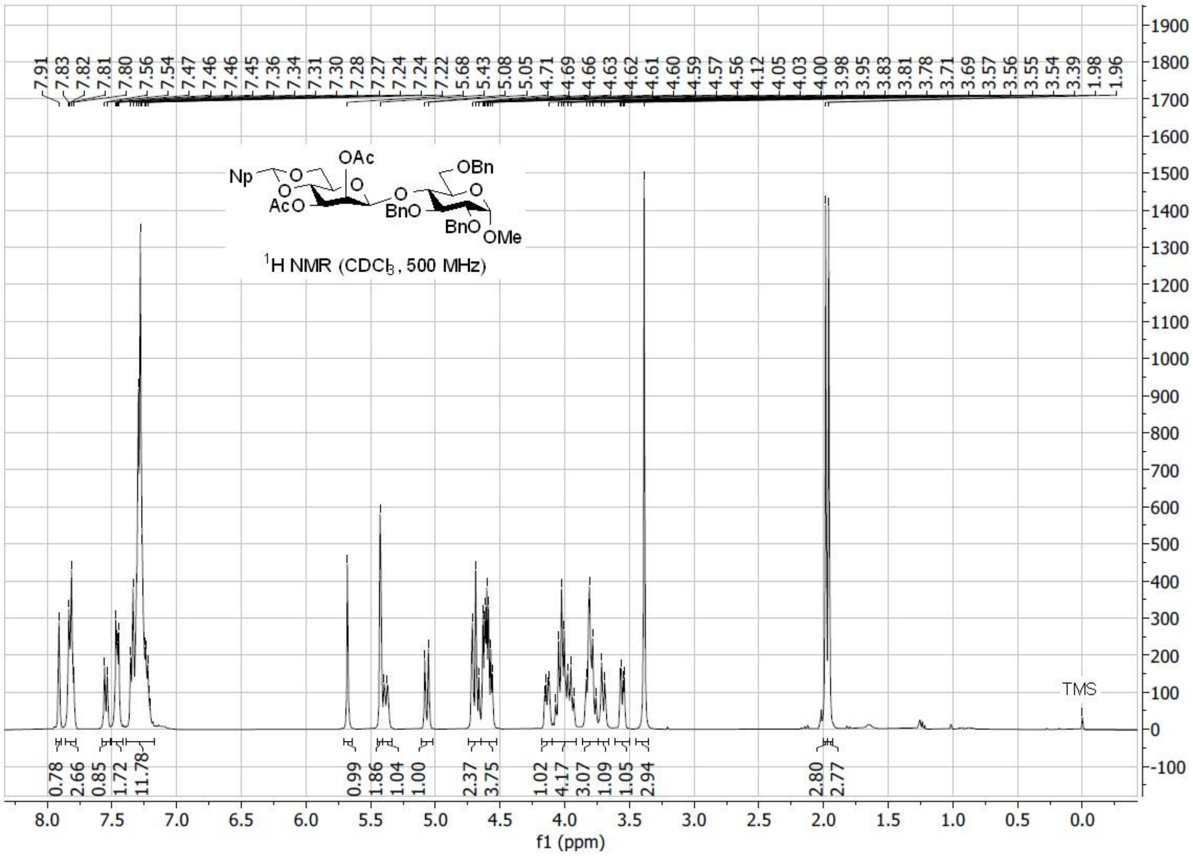

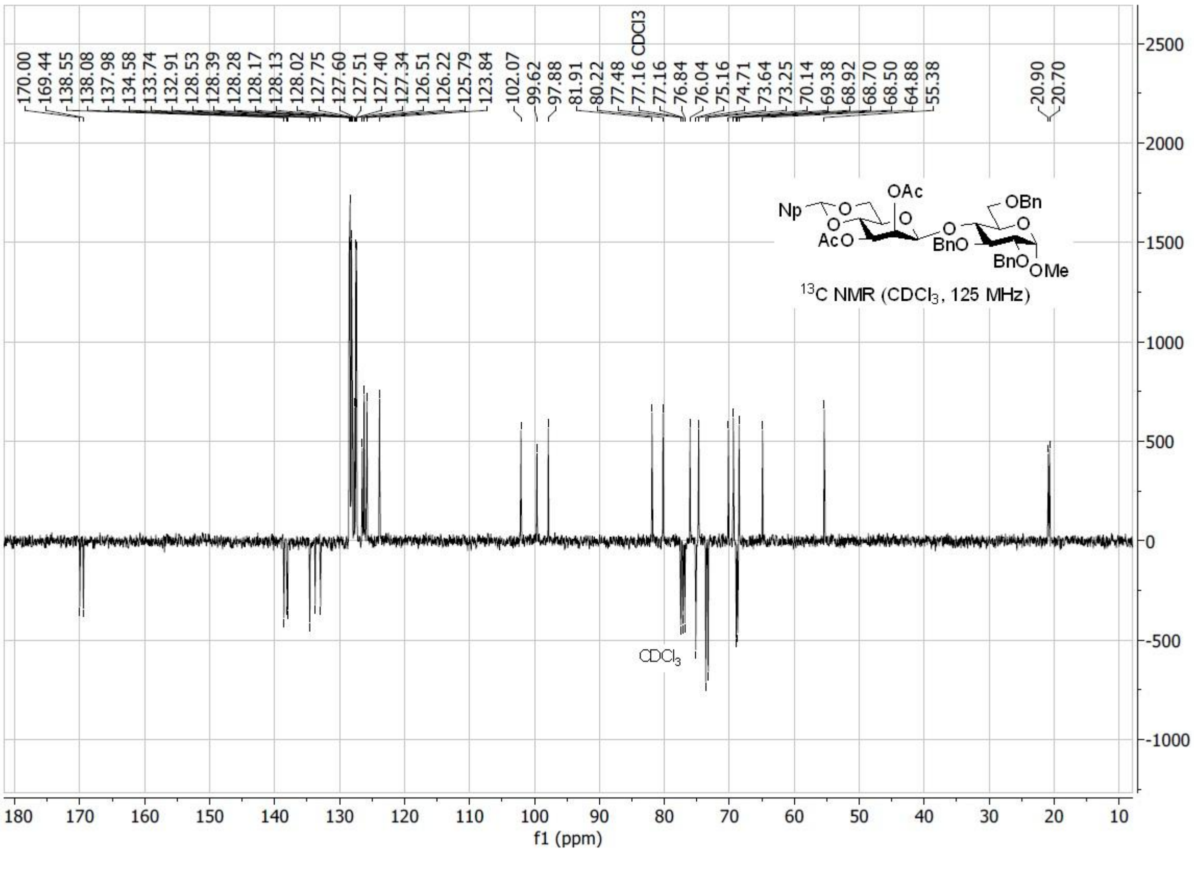

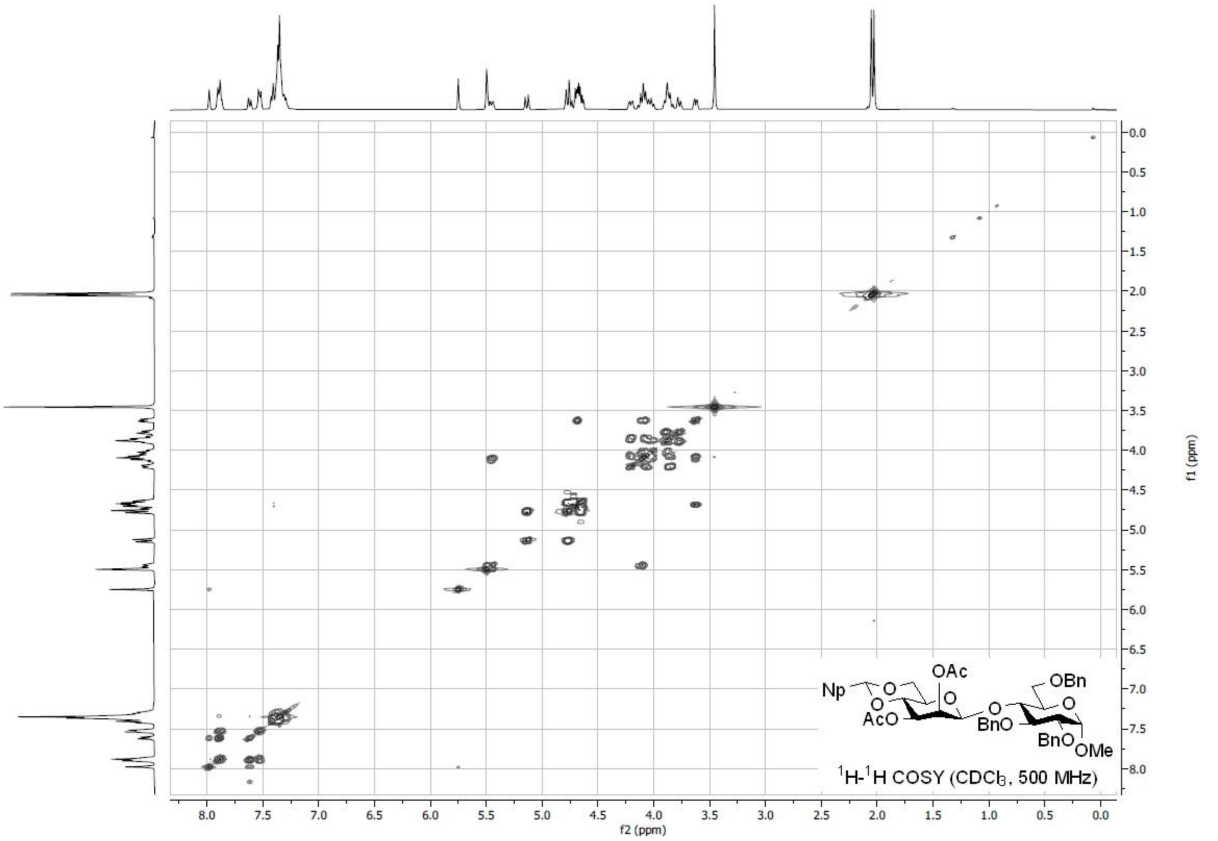

Np
OAc
OBn
AcO
BnO
BnO
OMe
^{1}H-^{1}H COSY (CDCl$_3$, 500 MHz)
f2 (ppm)
f1 (ppm)

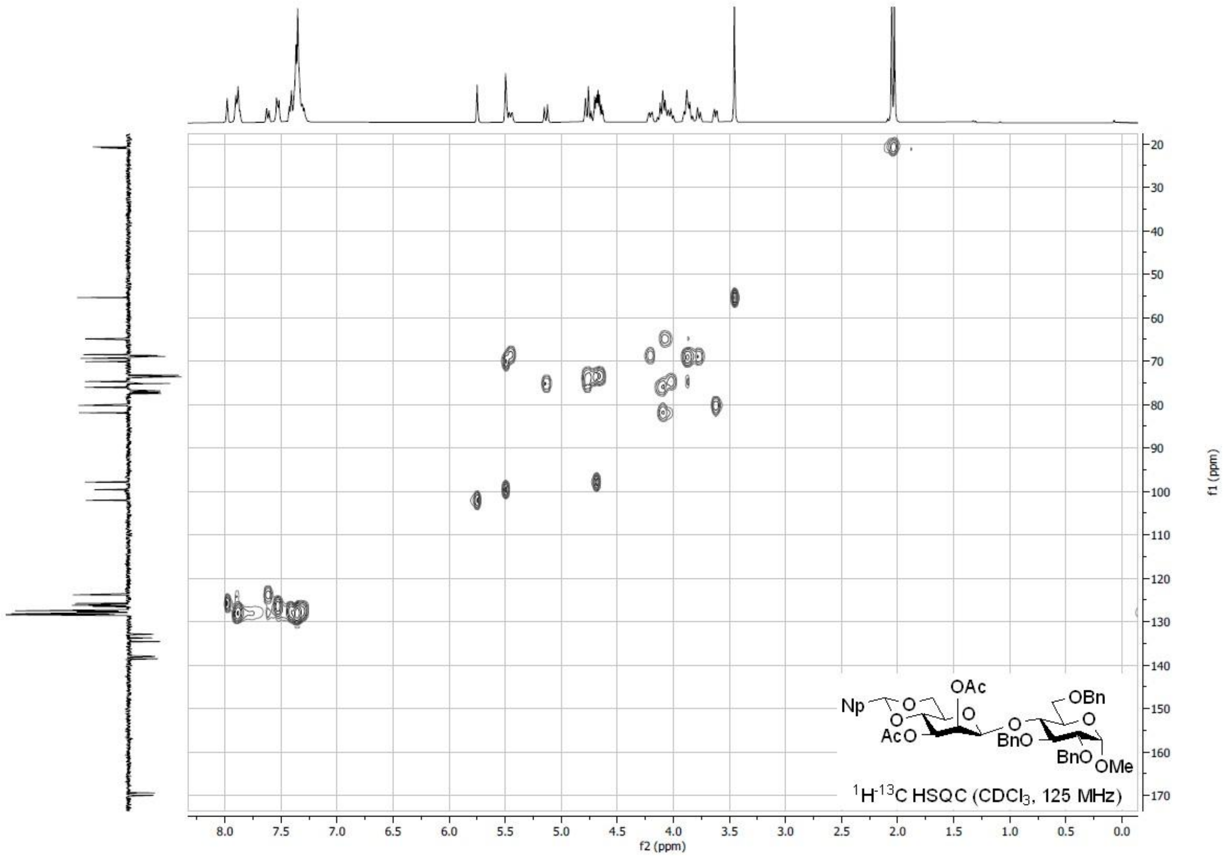

Np
OAc
OBn
AcO
BnO
BnO
OMe
^{1}H-^{13}C HSQC (CDCl$_3$, 125 MHz)
f2 (ppm)
f1 (ppm)

^{1}H, ^{13}C, COSY and HSQC NMR spectra of compound **25**

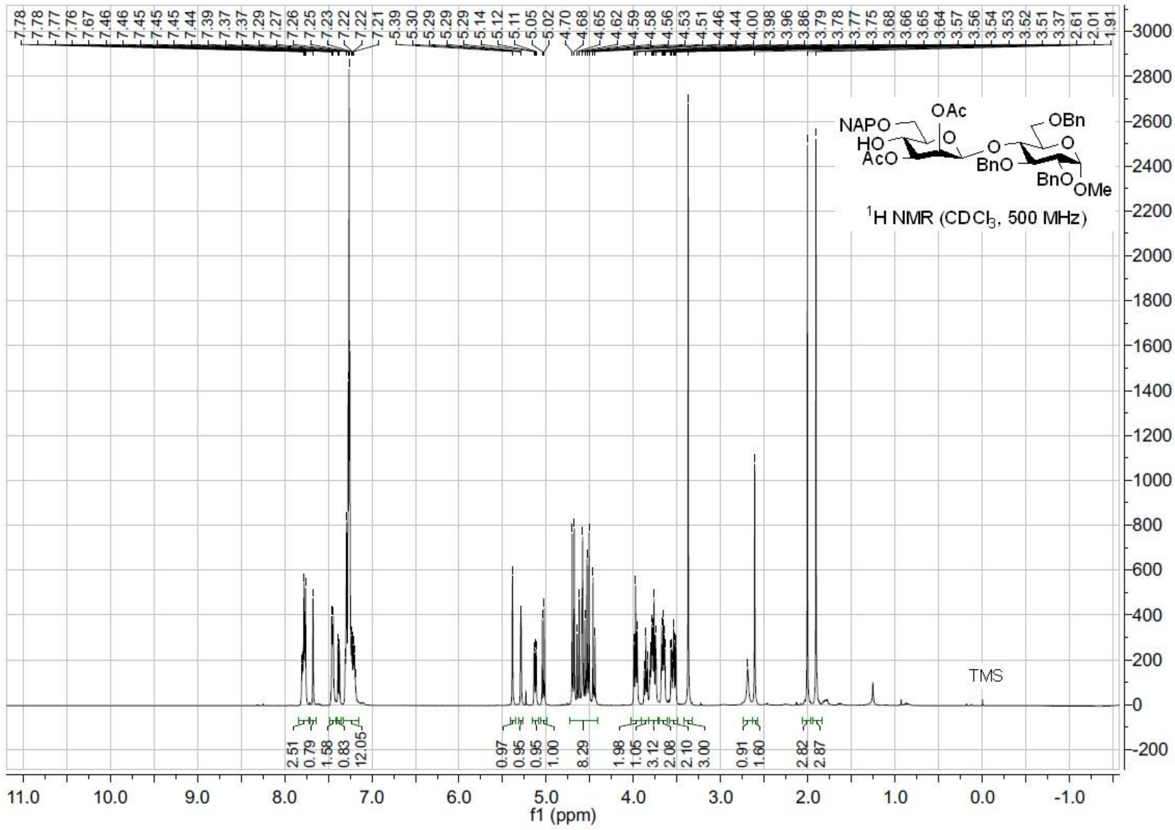

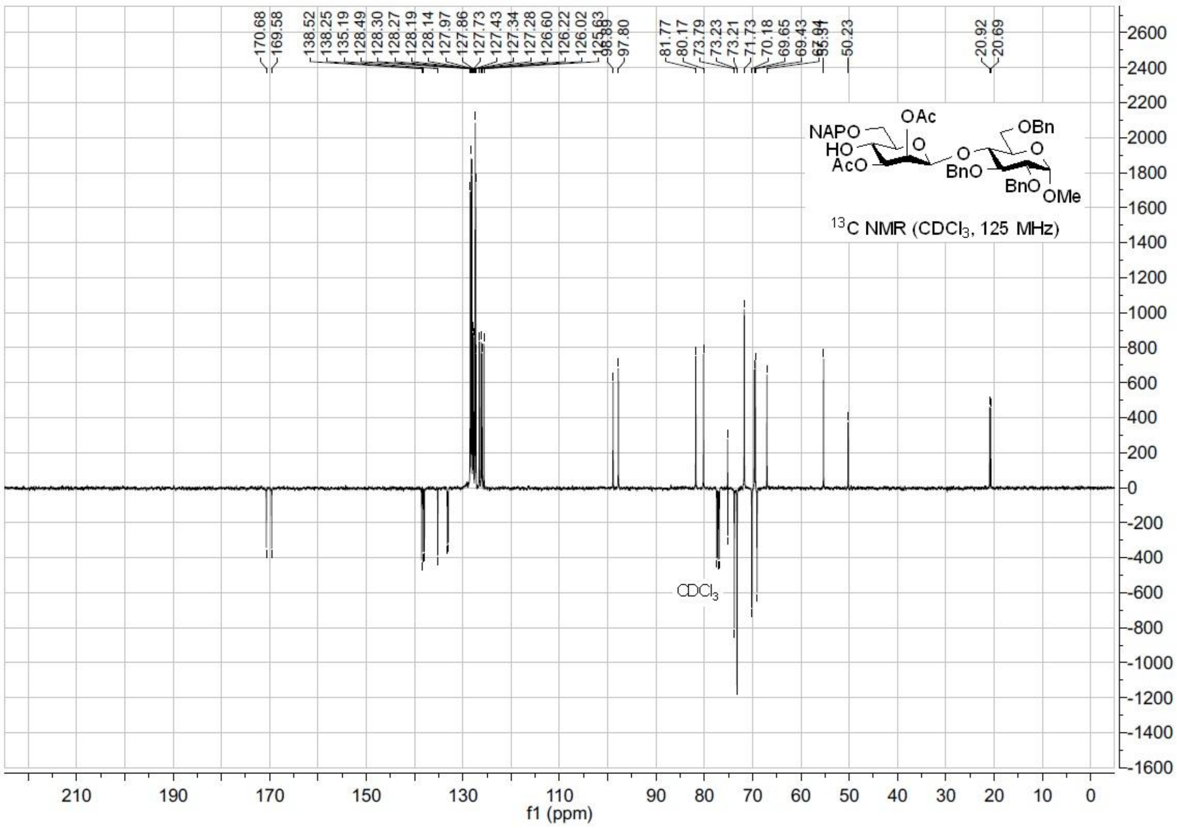

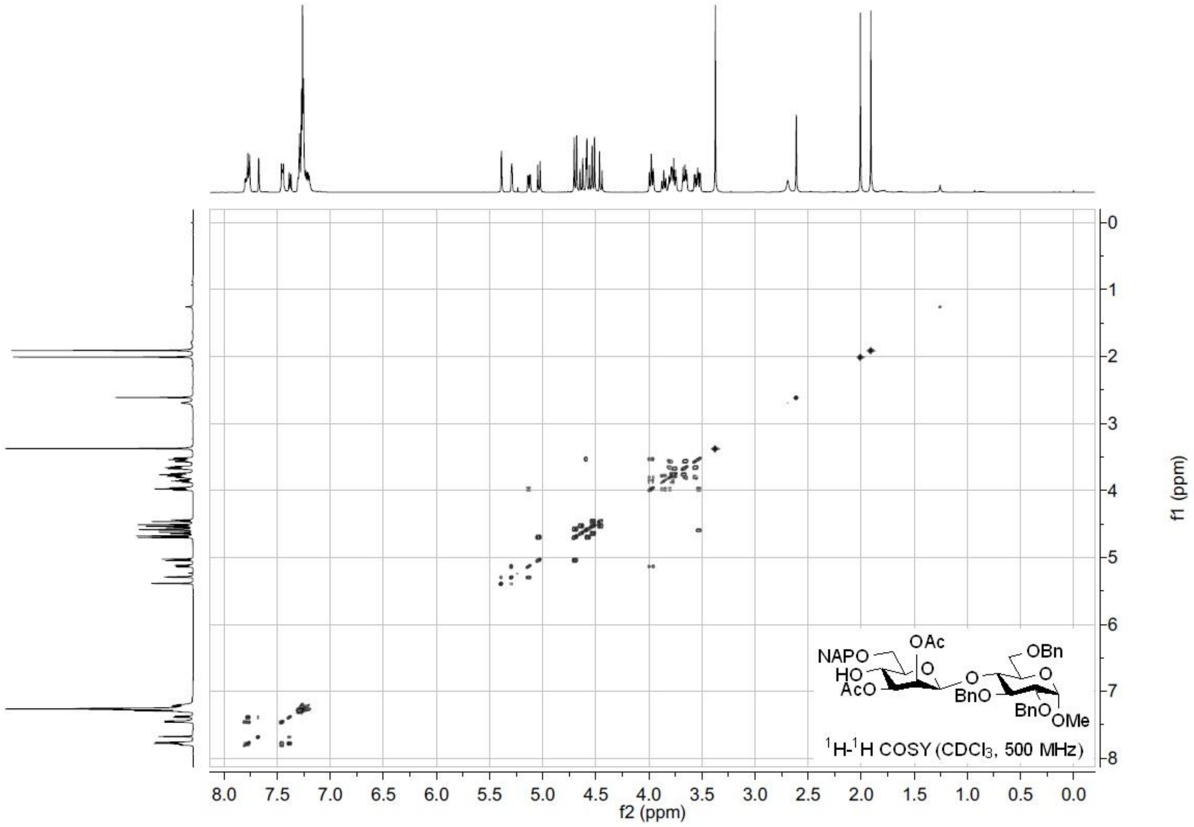
NAPO
OAc
O
HO
AcO
O
OBn
O
BnO
BnO
OMe
^{1}H-^{1}H COSY (CDCl$_3$, 500 MHz)
f2 (ppm)
f1 (ppm)

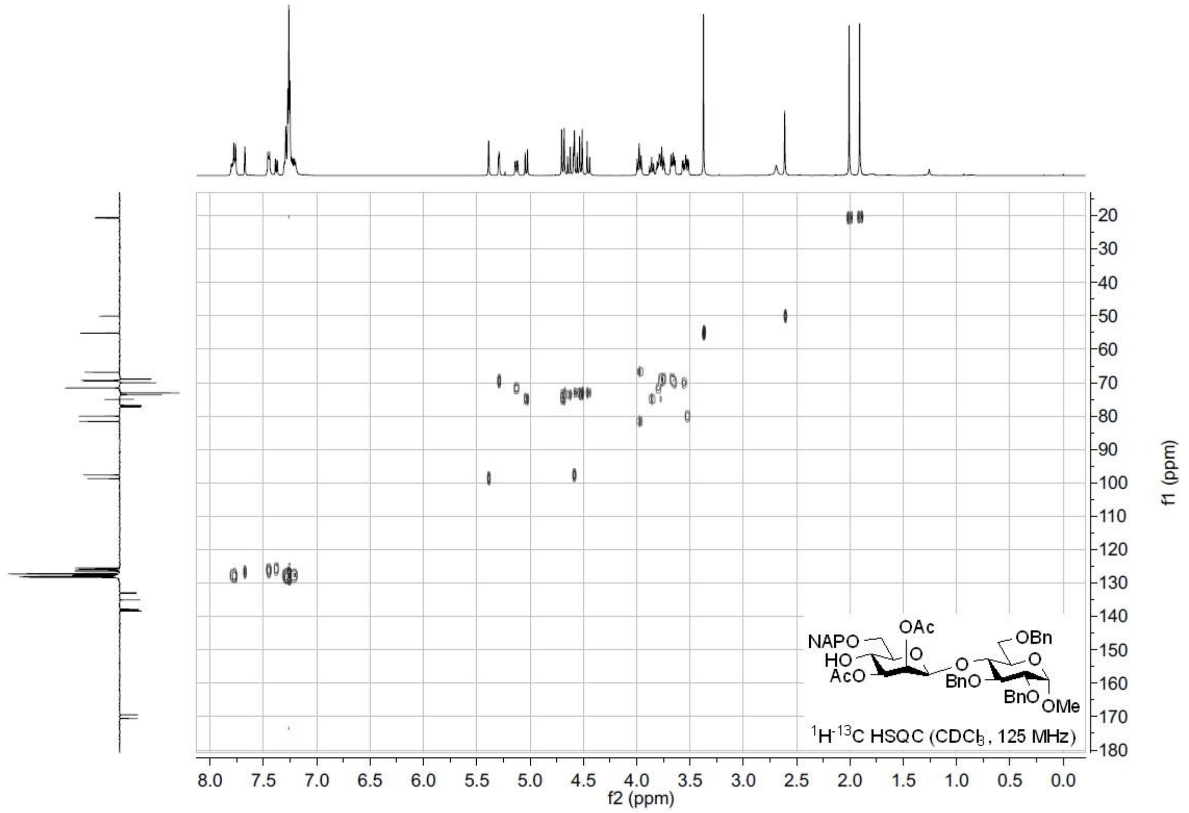
NAPO
OAc
O
HO
AcO
O
OBn
O
BnO
BnO
OMe
^{1}H-^{13}C HSQC (CDCl$_3$, 125 MHz)
f2 (ppm)
f1 (ppm)

^{1}H, ^{13}C, COSY and HSQC NMR spectra of compound **27**

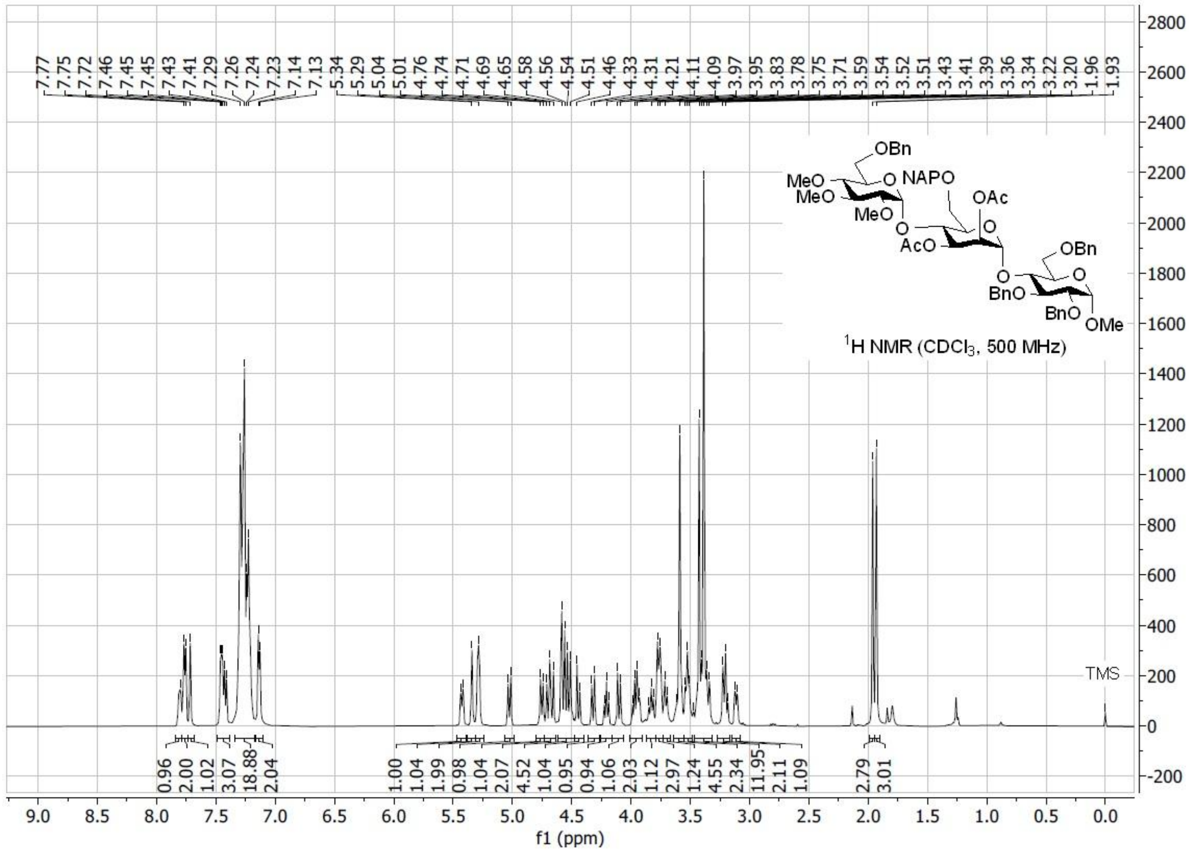

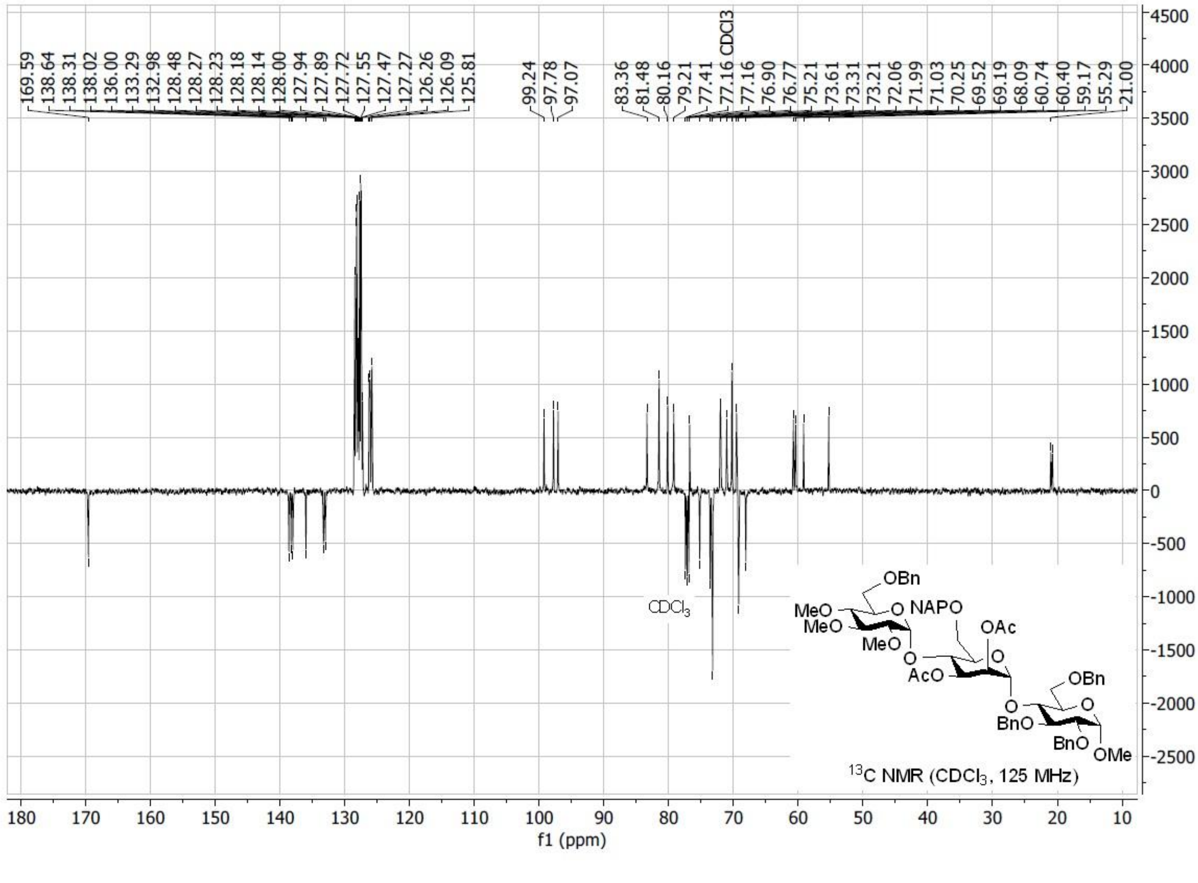

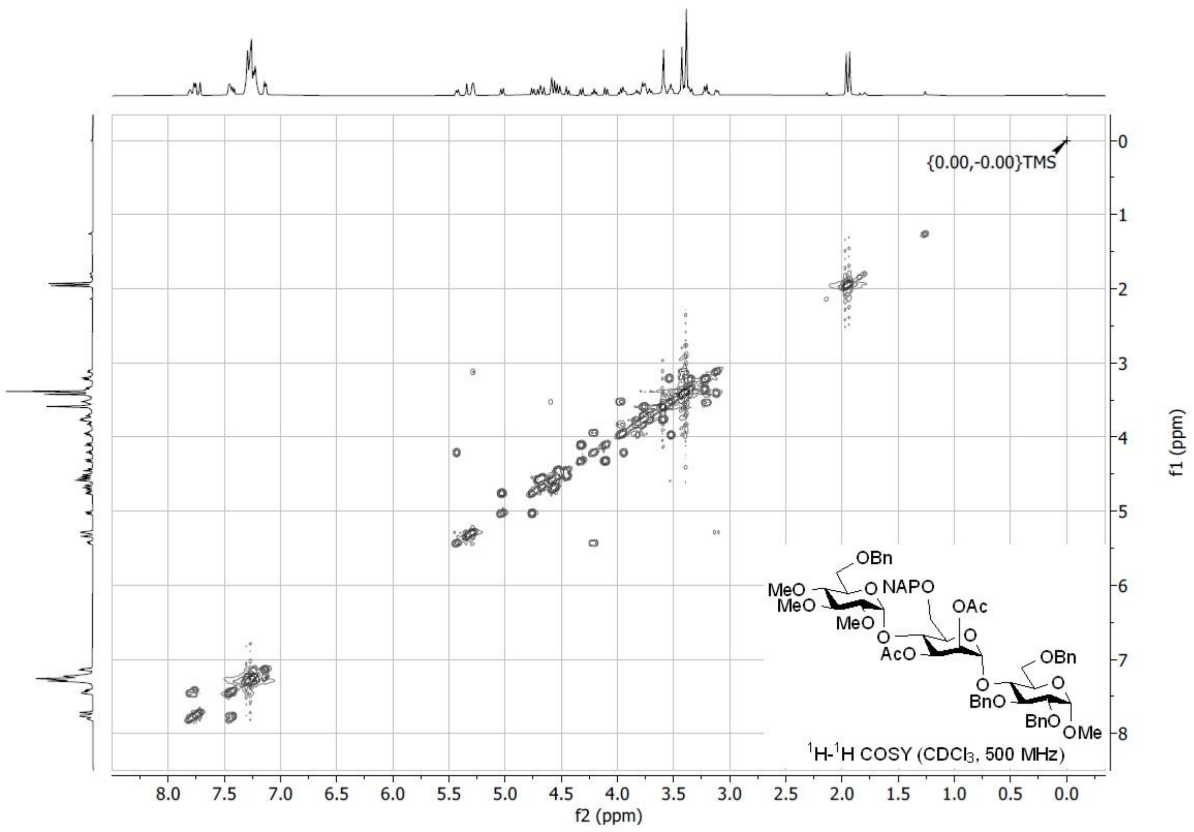

{0.00,-0.00}TMS
OBn
MeO
MeO
O
NAPO
OAc
MeO
O
O
AcO
O
OBn
O
BnO
O
BnO
OMe
^{1}H-^{1}H COSY (CDCl$_3$, 500 MHz)
f1 (ppm)
f2 (ppm)

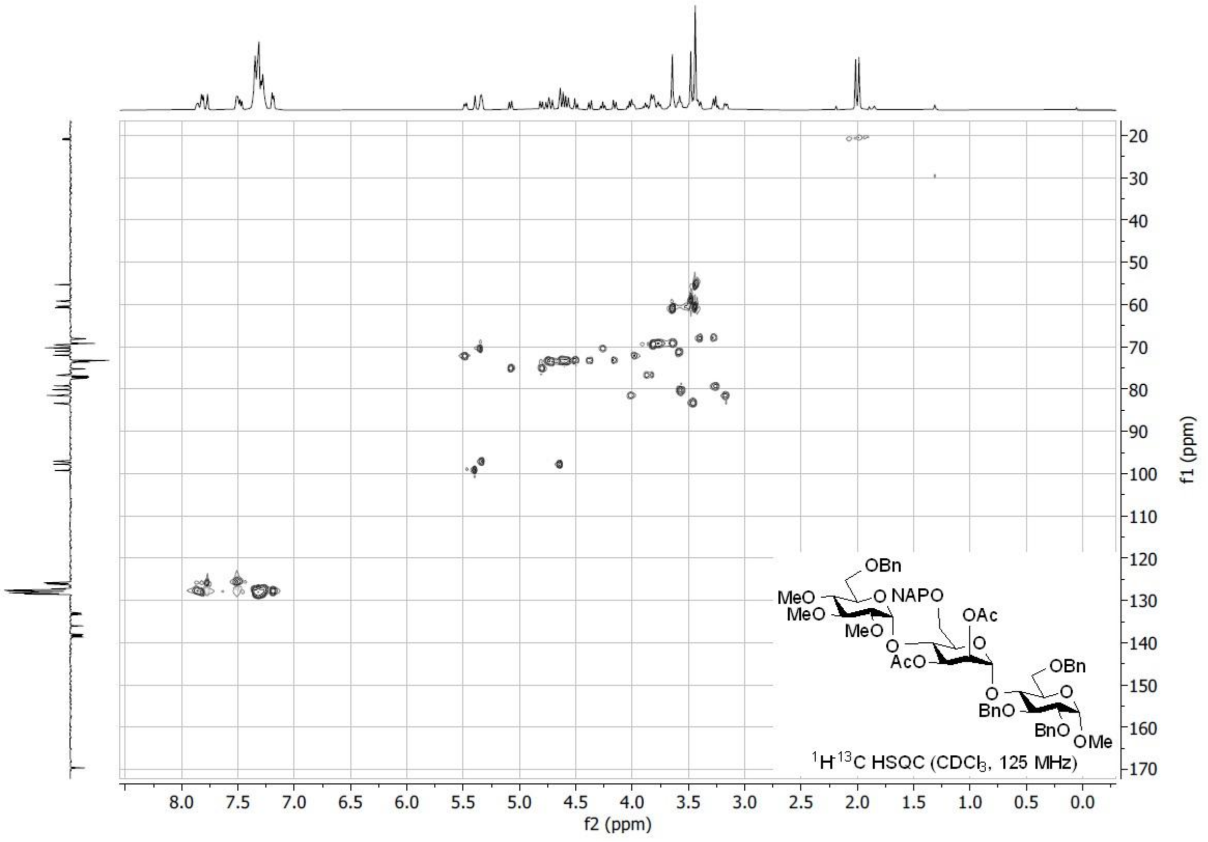

OBn
MeO
MeO
O
NAPO
OAc
MeO
O
O
AcO
O
OBn
O
BnO
O
BnO
OMe
^{1}H-^{13}C HSQC (CDCl$_3$, 125 MHz)
f1 (ppm)
f2 (ppm)

^{1}H, ^{13}C, COSY, HMBC and HSQC NMR spectra of compound **28**

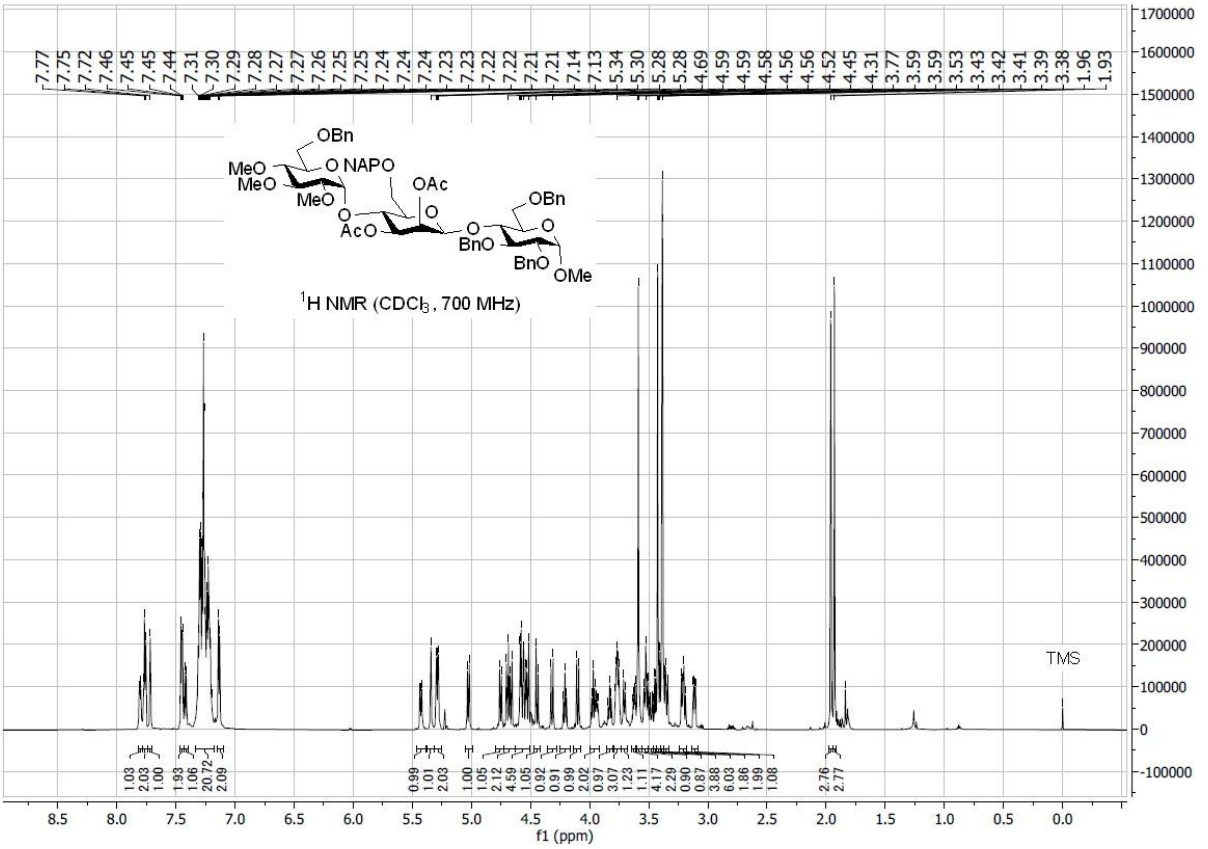

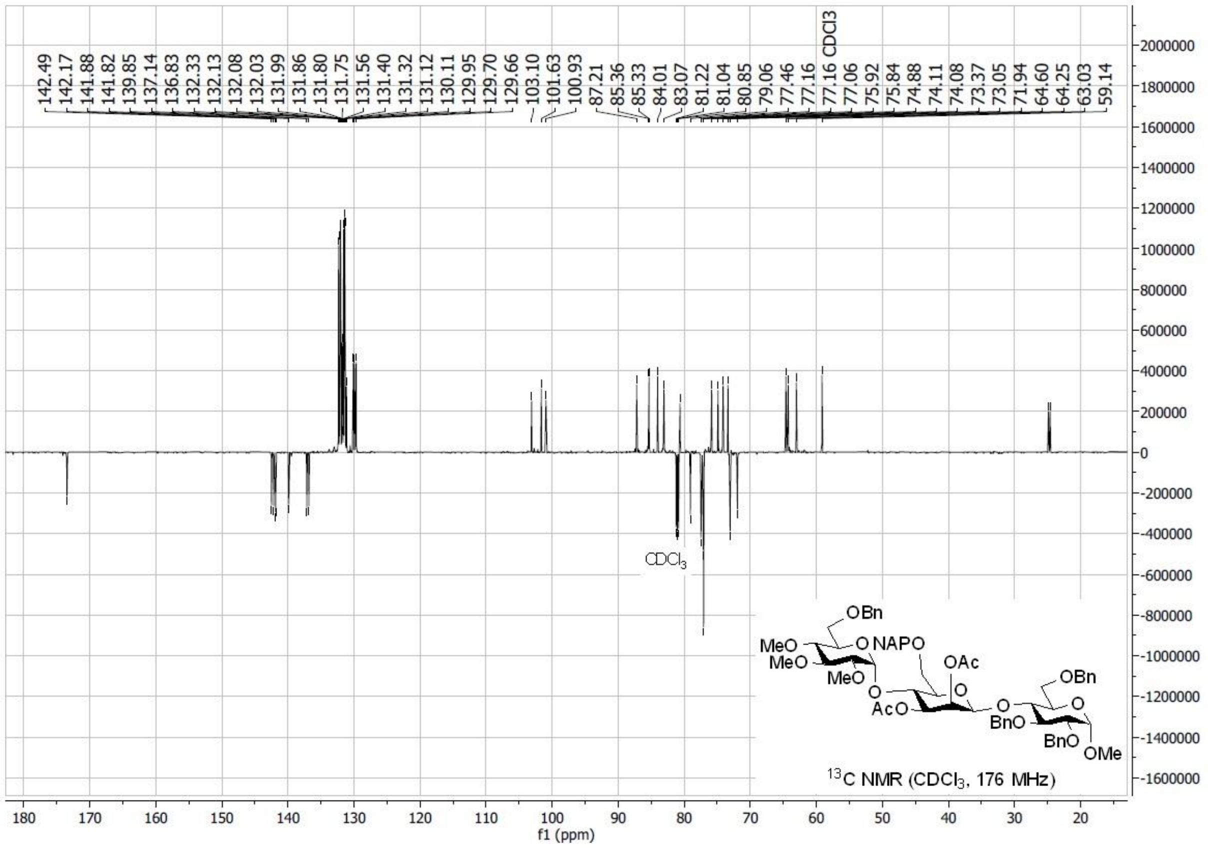

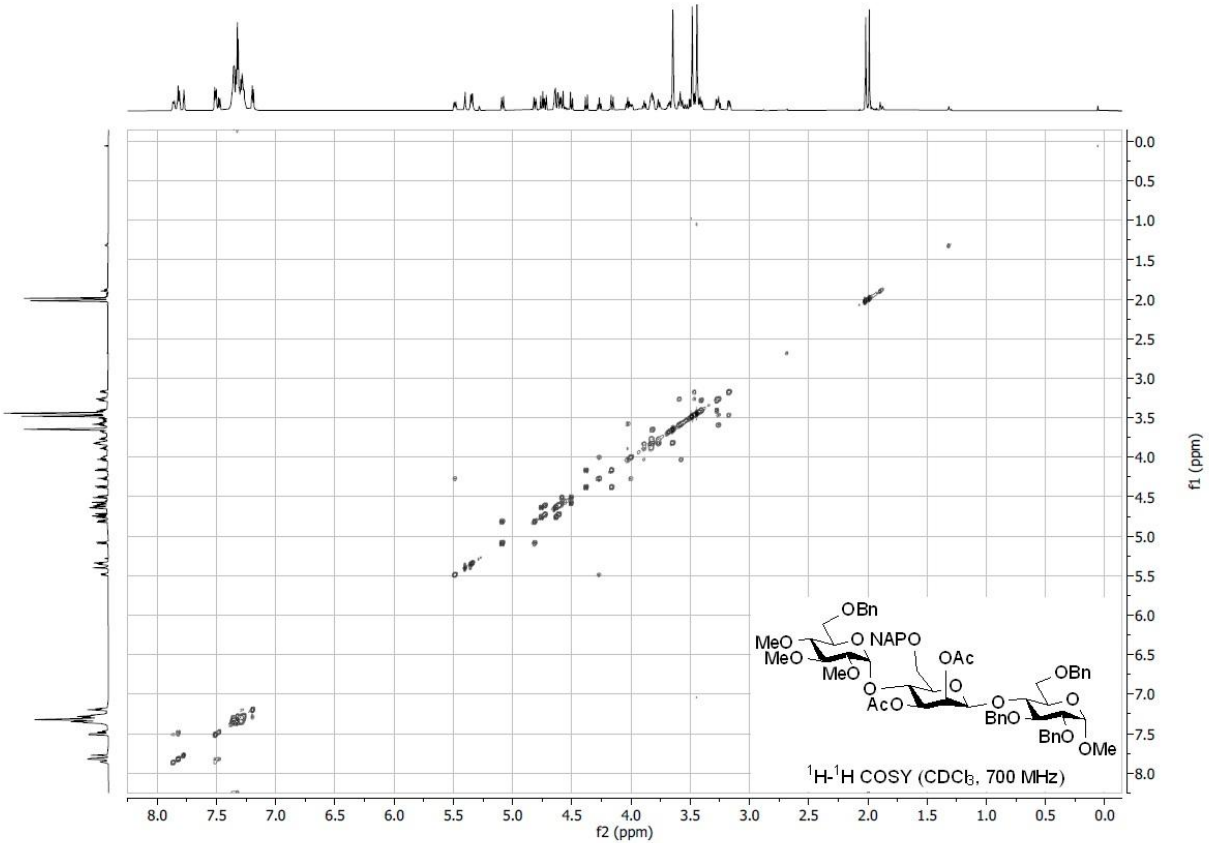
OBn
MeO
MeO
O NAPO
OAc
MeO
O
AcO
O
O
OBn
O
BnO
BnO
OMe
^{1}H-^{1}H COSY (CDCl$_3$, 700 MHz)
f1 (ppm)
f2 (ppm)

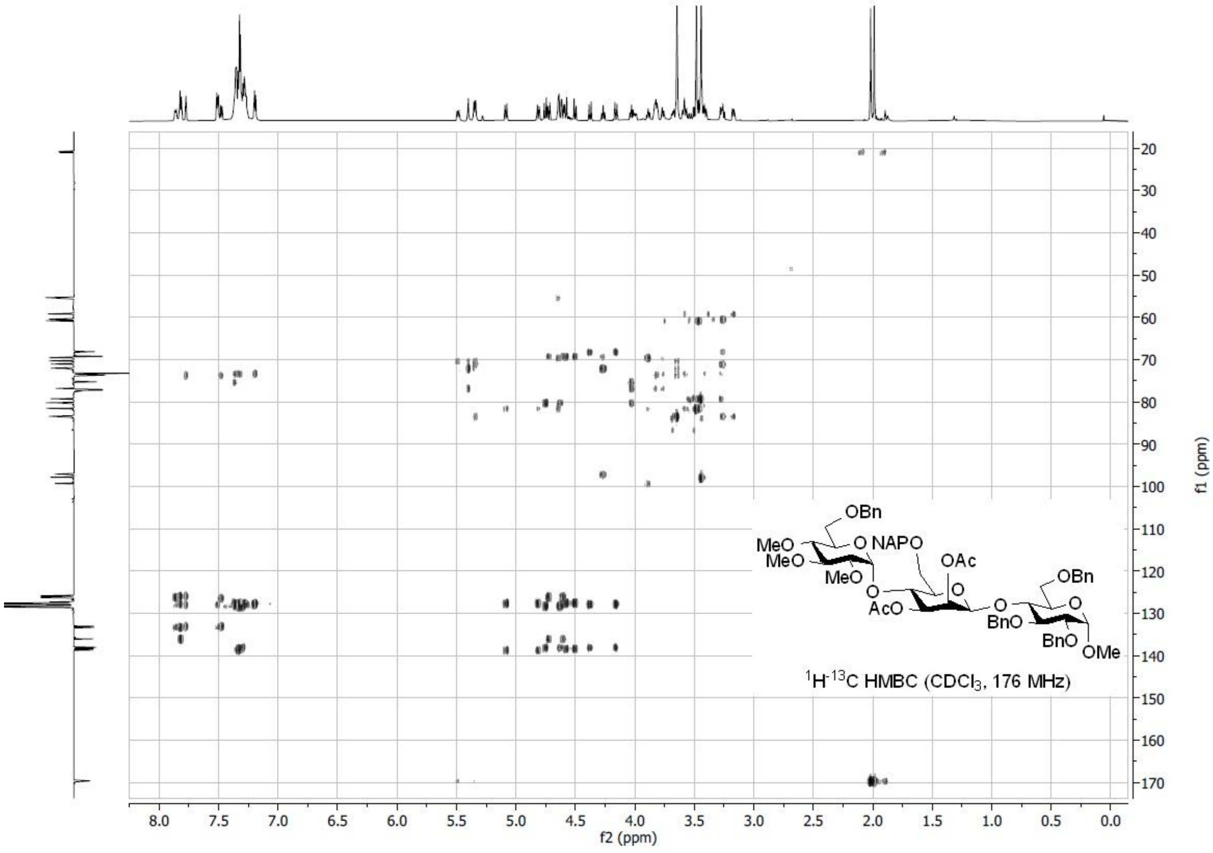
OBn
MeO
MeO
O NAPO
OAc
MeO
O
AcO
O
O
OBn
O
BnO
BnO
OMe
^{1}H-^{13}C HMBC (CDCl$_3$, 176 MHz)
f1 (ppm)
f2 (ppm)

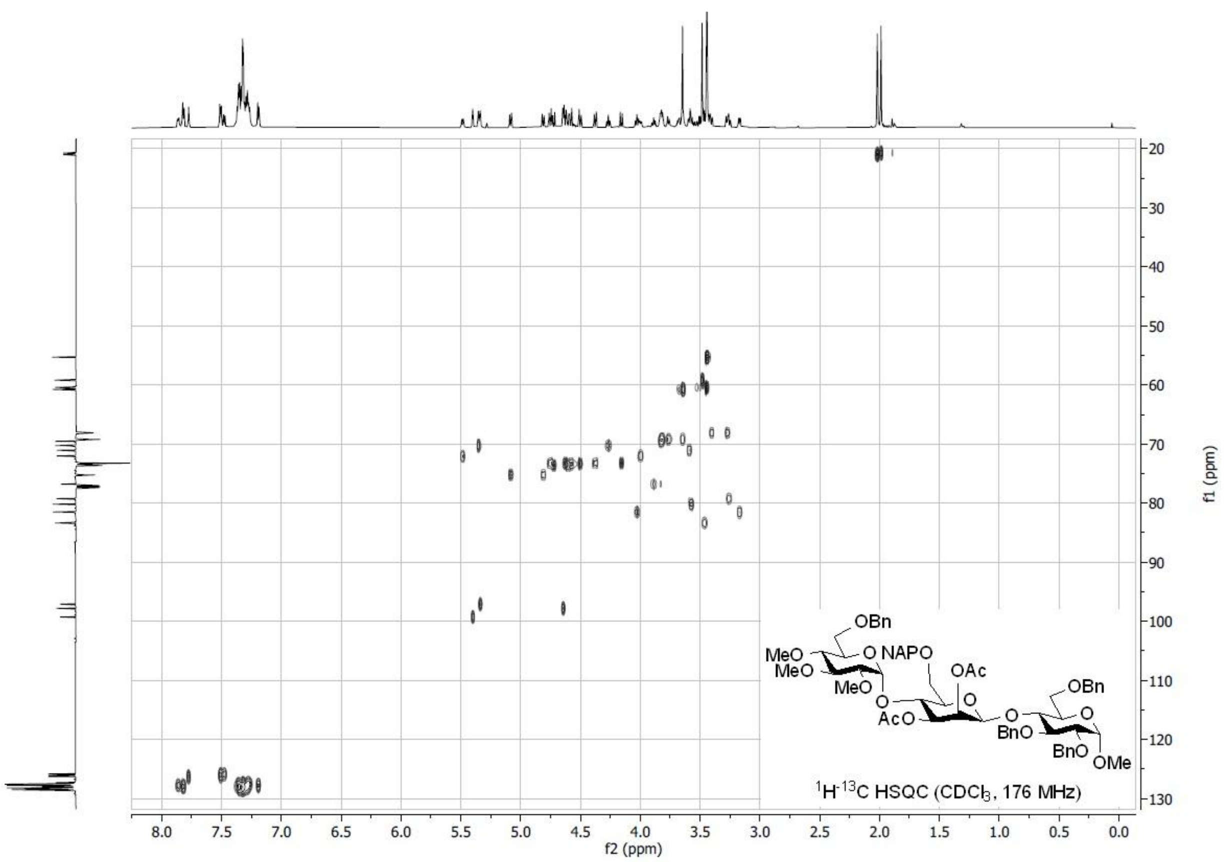
OBn
MeO
MeO
O
NAPO
OAc
MeO
O
AcO
O
O
OBn
O
BnO
BnO
OMe
^{1}H-^{13}C HSQC (CDCl$_3$, 176 MHz)
f2 (ppm)
f1 (ppm)

^{1}H, ^{13}C, COSY and HSQC NMR spectra of compound **30**

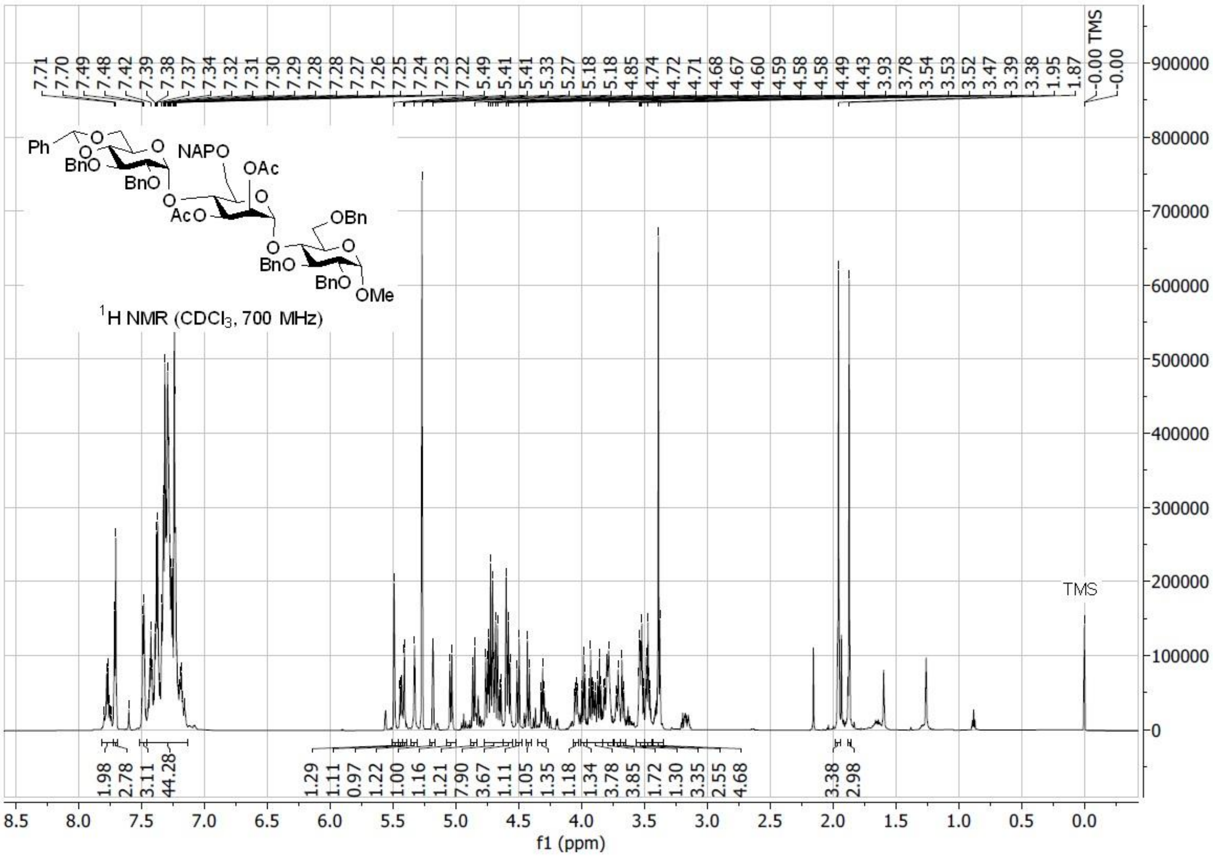

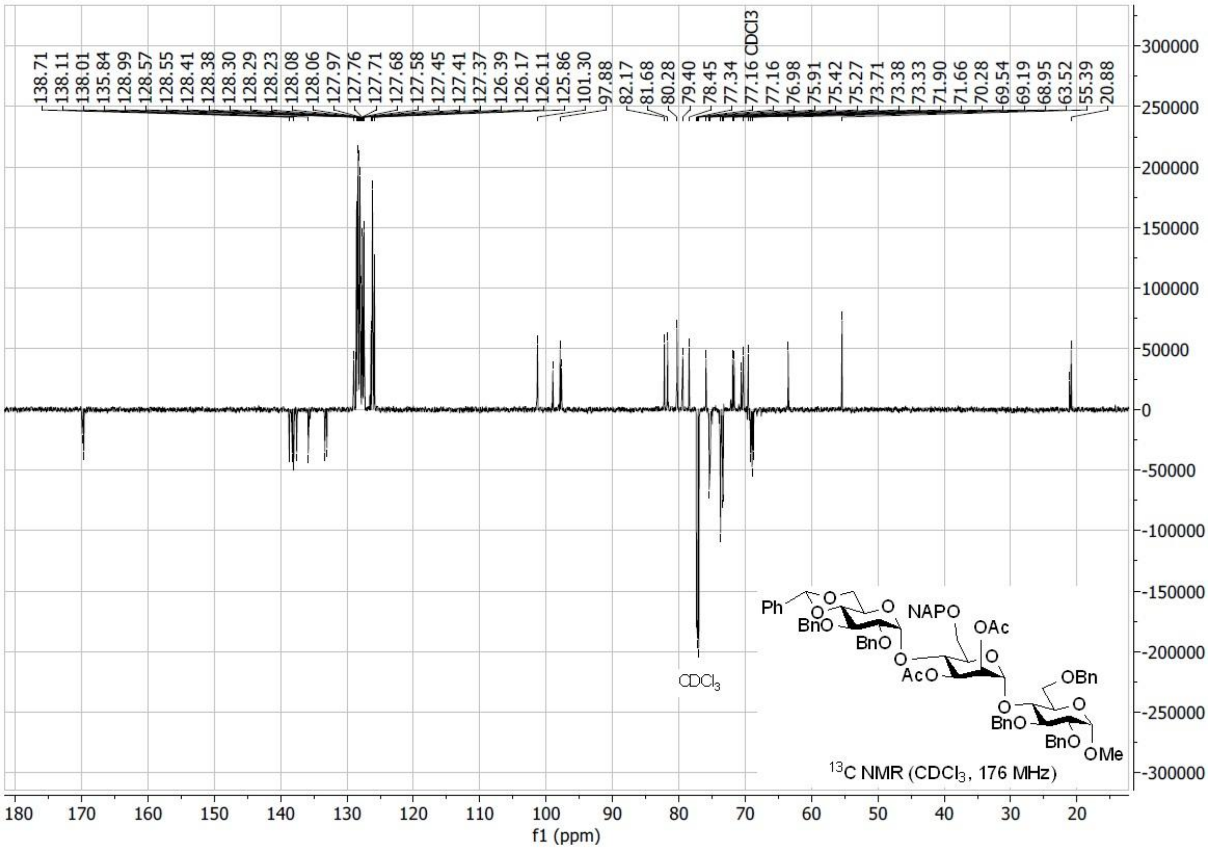

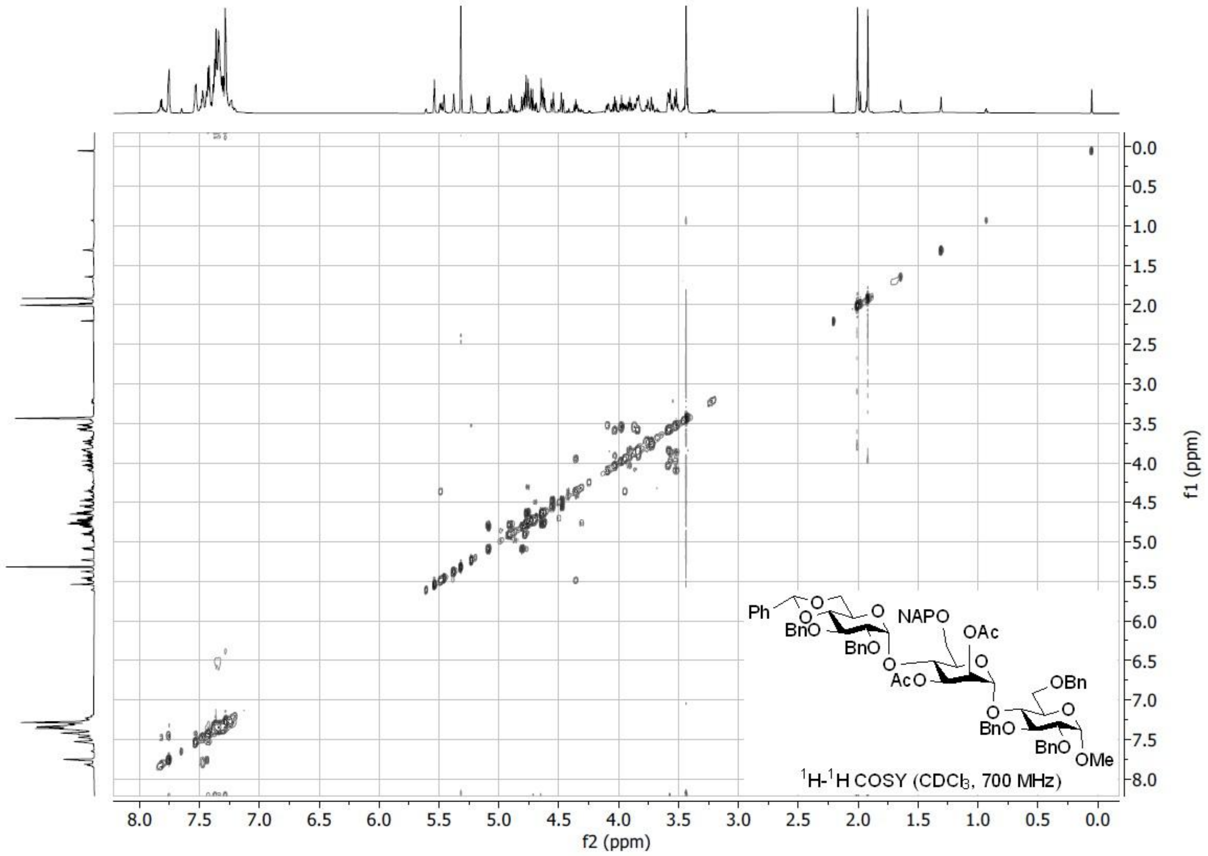
Ph
O
O
O
BnO
BnO
NAPO
OAc
O
O
AcO
O
OBn
O
O
BnO
BnO
OMe
1H-1H COSY (CDCl3, 700 MHz)
f1 (ppm)
f2 (ppm)

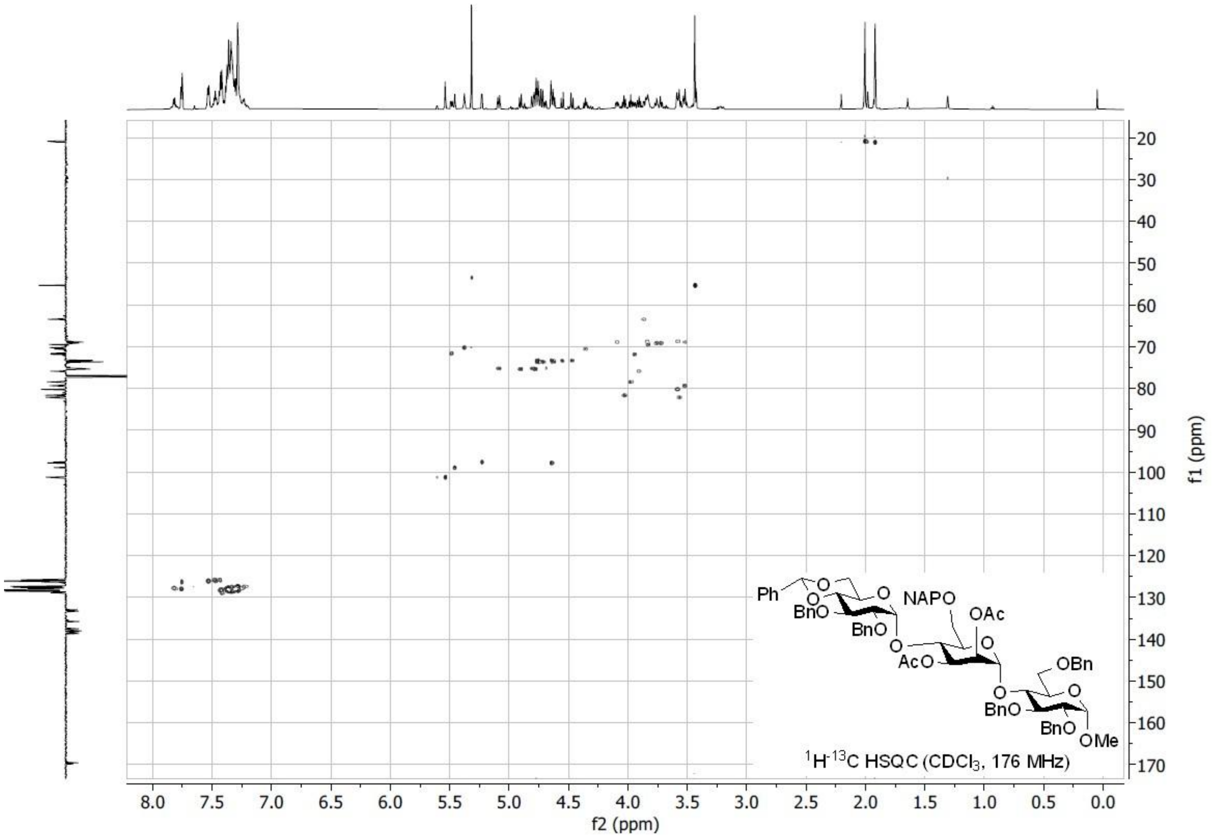
Ph
O
O
O
BnO
BnO
NAPO
OAc
O
O
AcO
O
OBn
O
O
BnO
BnO
OMe
1H-13C HSQC (CDCl3, 176 MHz)
f1 (ppm)
f2 (ppm)

^{1}H, ^{13}C, COSY and HSQC NMR spectra of compound **32**

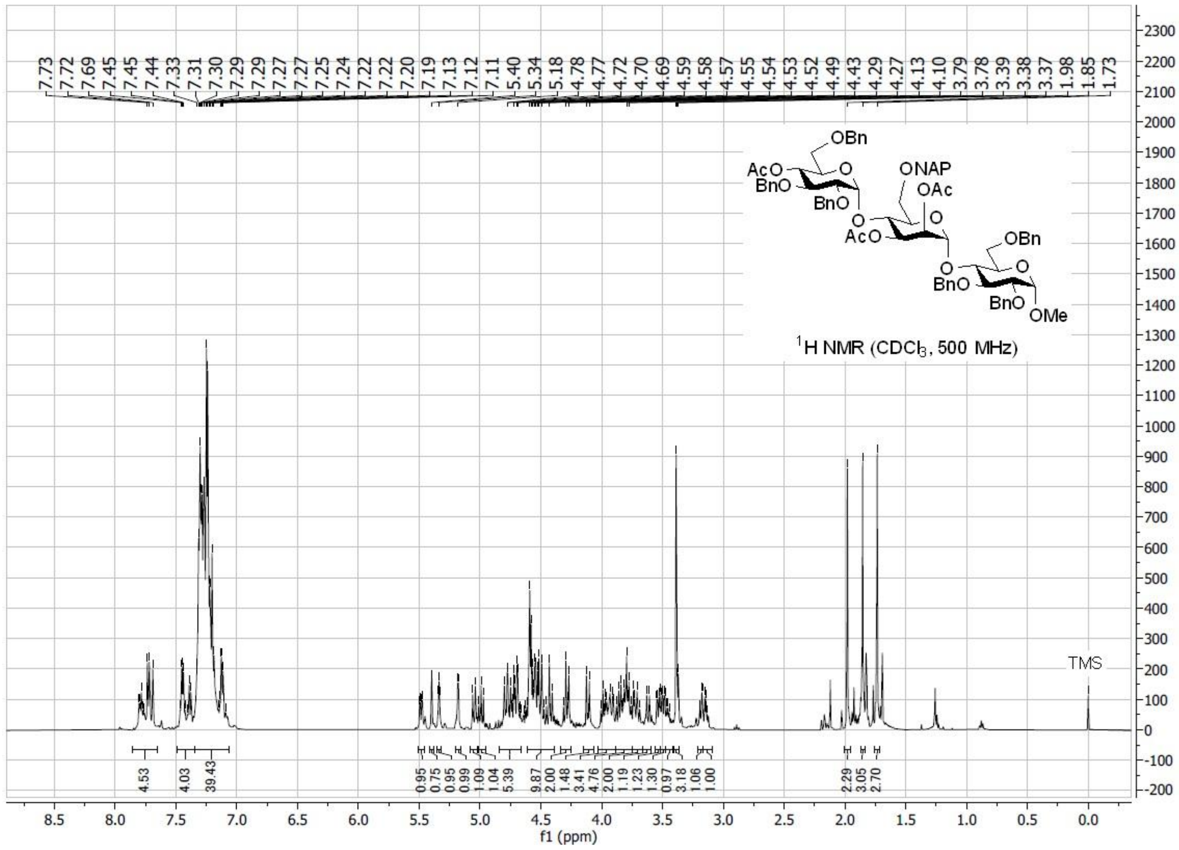

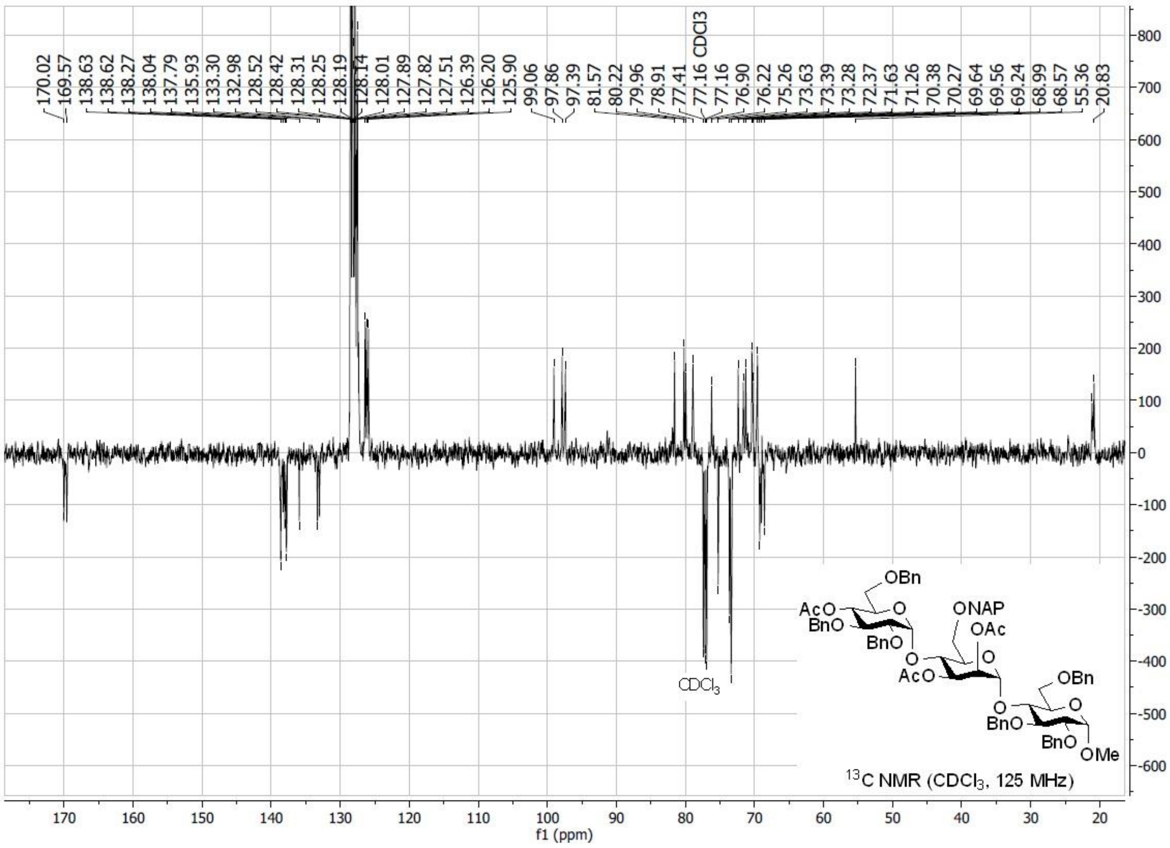

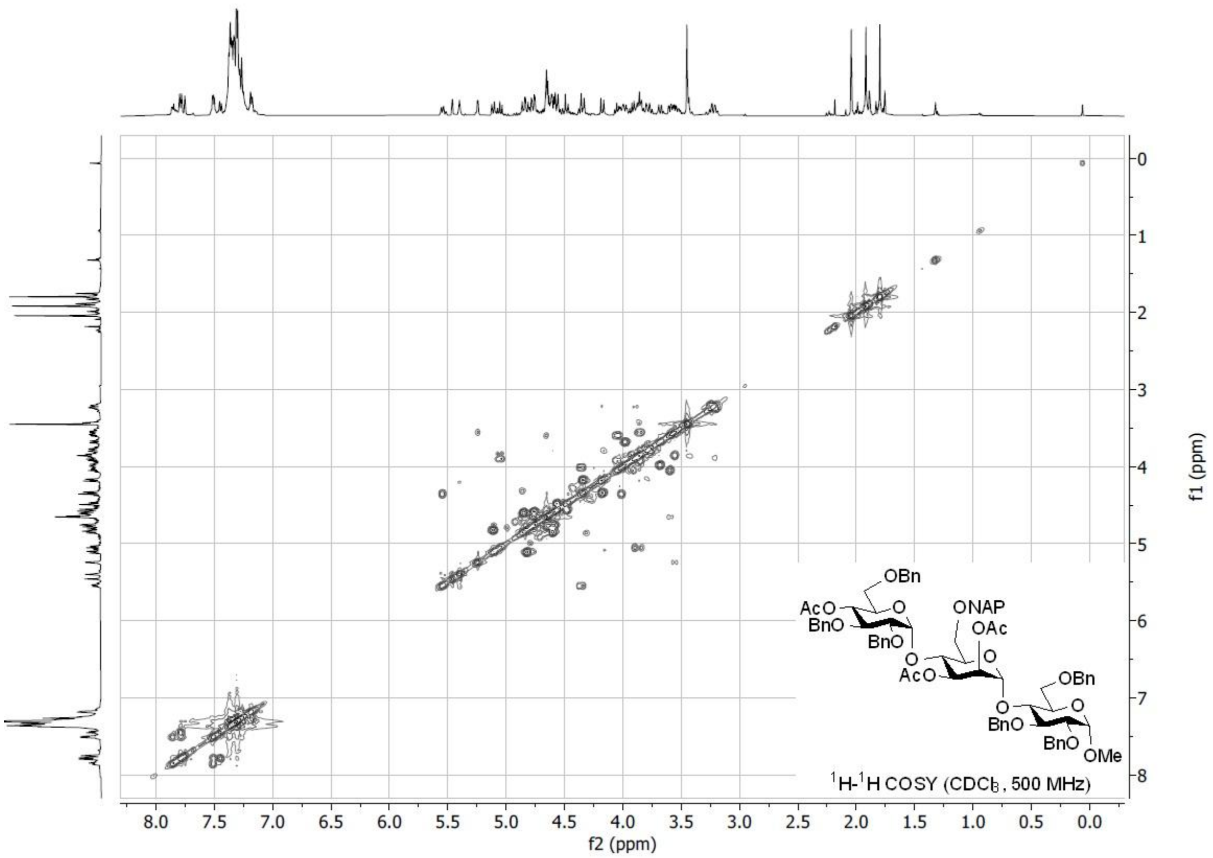

OBn
AcO
BnO
BnO
O
ONAP
OAc
AcO
OBn
BnO
BnO
OMe
^{1}H-^{1}H COSY ($CDCl_3$, 500 MHz)
f2 (ppm)
f1 (ppm)

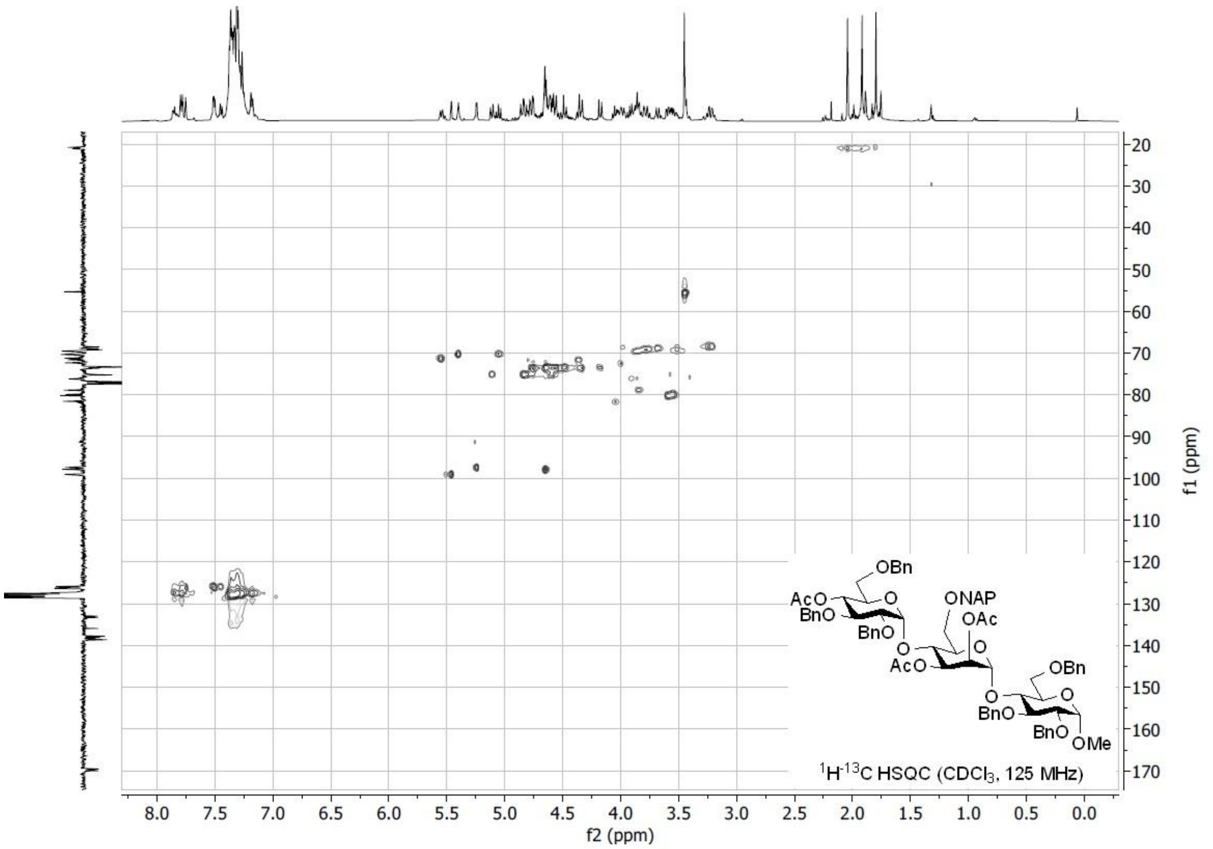

OBn
AcO
BnO
BnO
O
ONAP
OAc
AcO
OBn
BnO
BnO
OMe
^{1}H-^{13}C HSQC ($CDCl_3$, 125 MHz)
f2 (ppm)
f1 (ppm)

^{1}H, ^{13}C, COSY, HMBC and HSQC NMR spectra of compound **34**

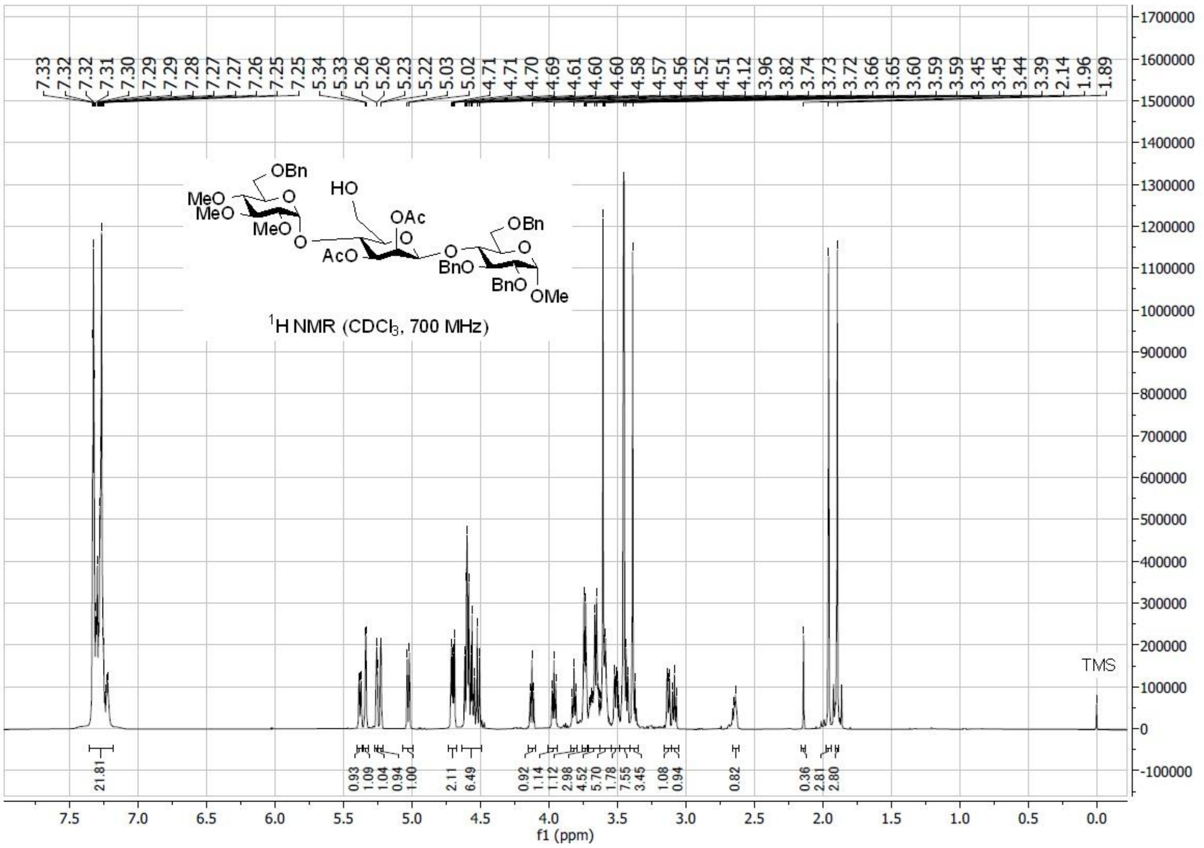

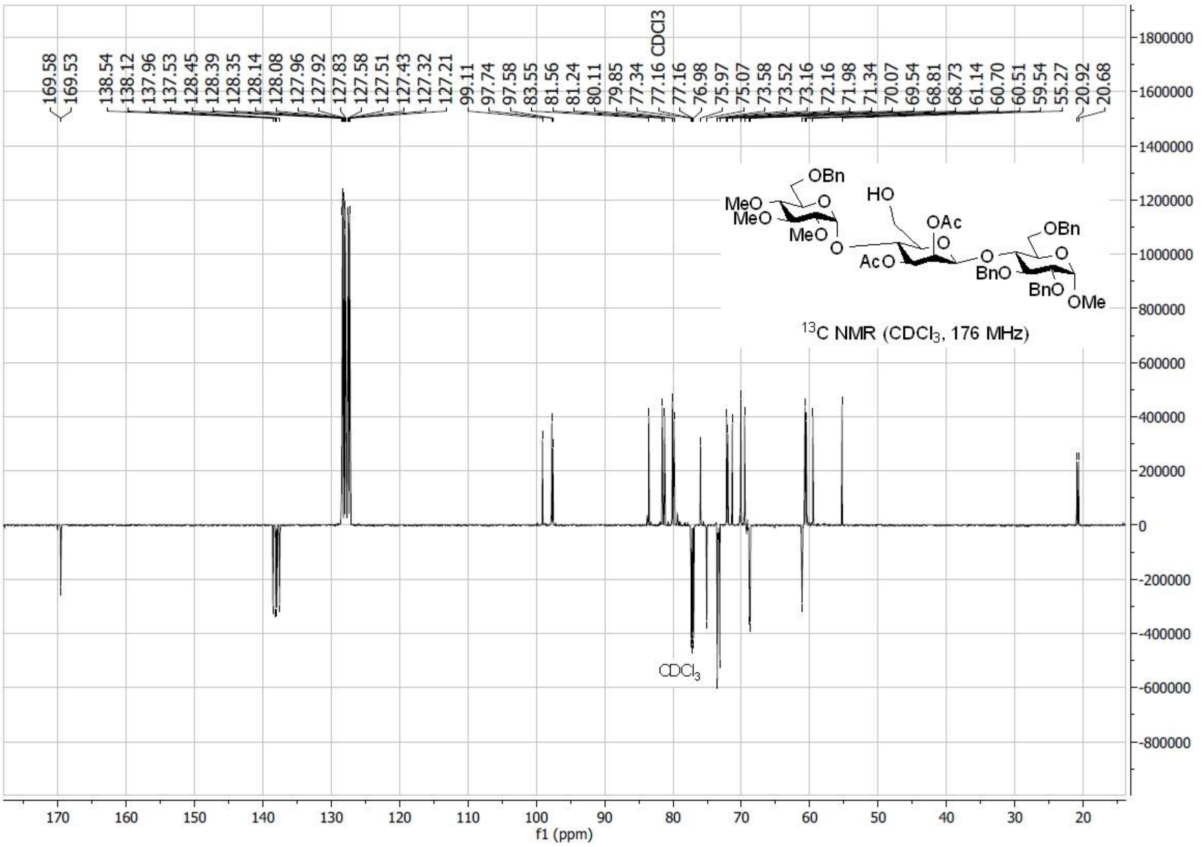

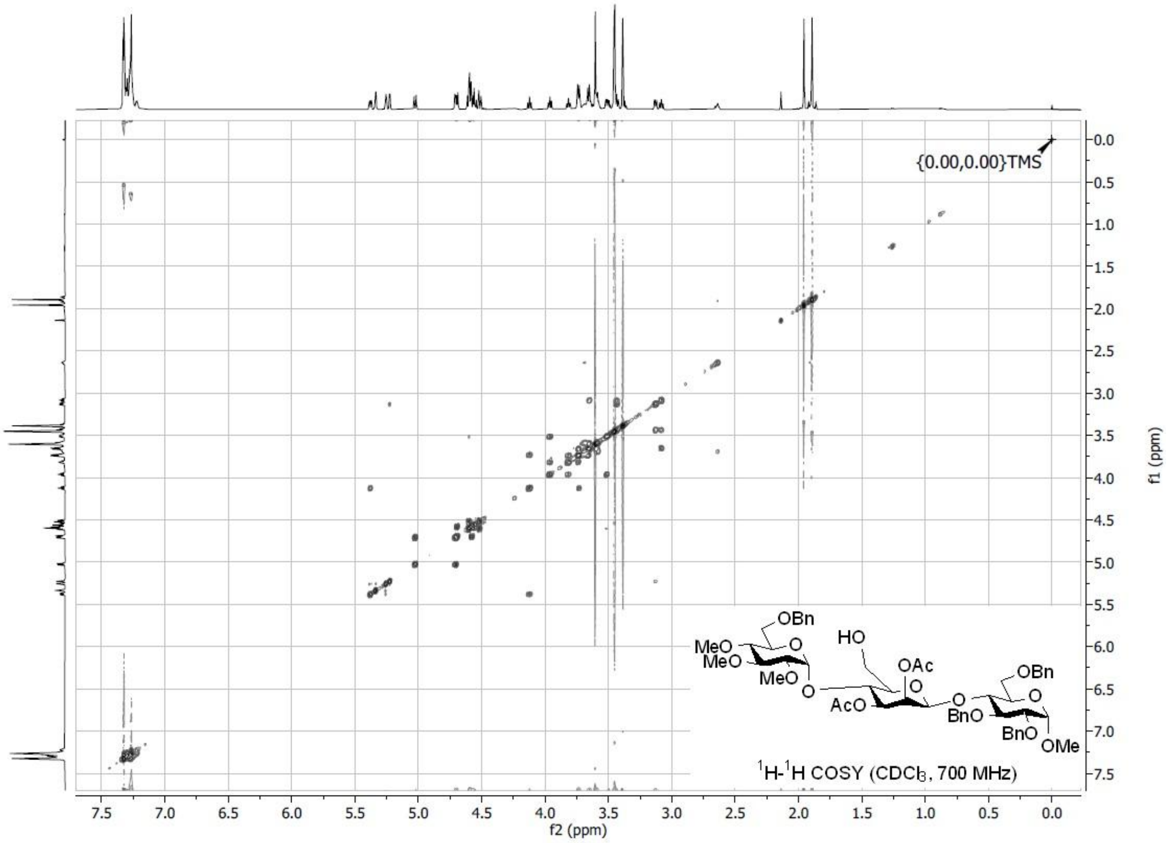
{0.00,0.00}TMS
OBn
MeO
MeO
MeO
O
HO
OAc
AcO
BnO
BnO
OMe
^{1}H-^{1}H COSY (CDCl$_3$, 700 MHz)
f2 (ppm)
f1 (ppm)

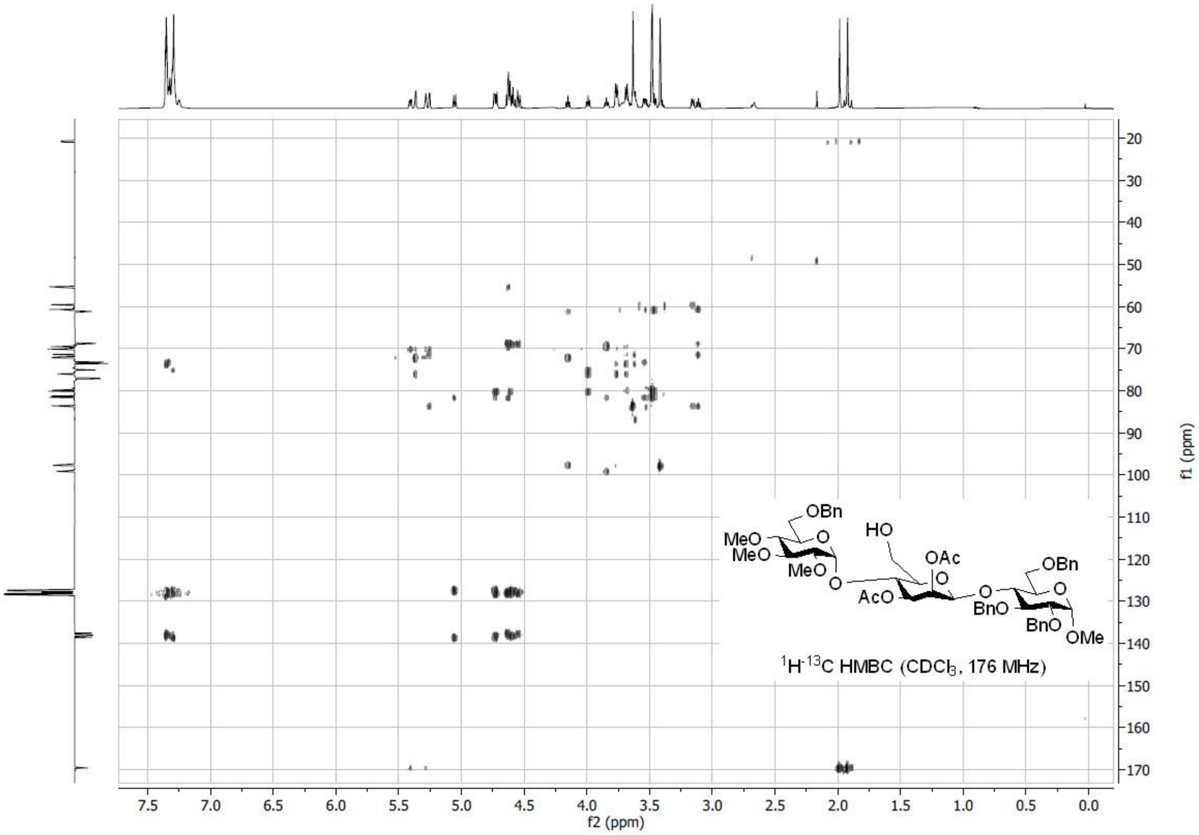
OBn
MeO
MeO
MeO
O
HO
OAc
AcO
BnO
BnO
OMe
^{1}H-^{13}C HMBC (CDCl$_3$, 176 MHz)
f2 (ppm)
f1 (ppm)

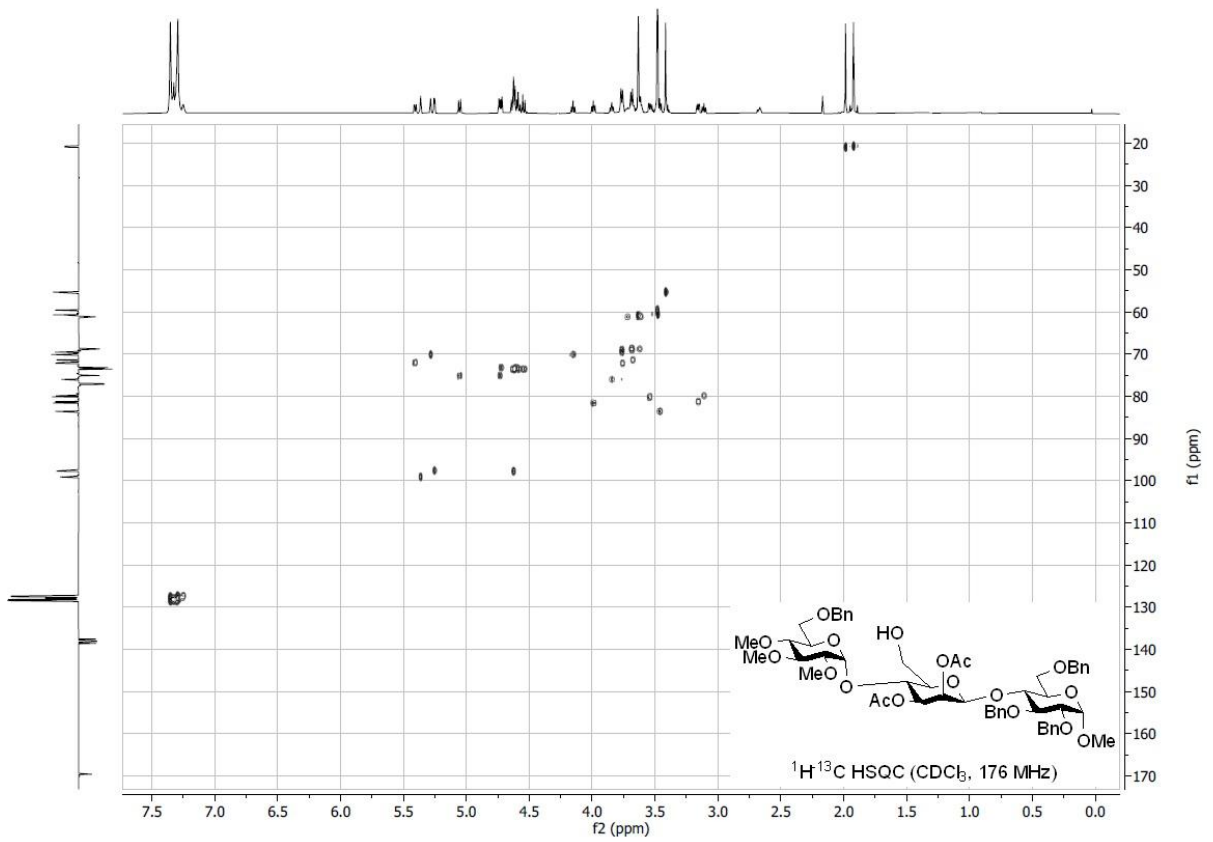

f1 (ppm)
f2 (ppm)
OBn
MeO
MeO
MeO
O
HO
OAc
AcO
BnO
BnO
OMe
1H-13C HSQC (CDCl3, 176 MHz)

^1H, ^{13}C, COSY and HSQC NMR spectra of compound **35**

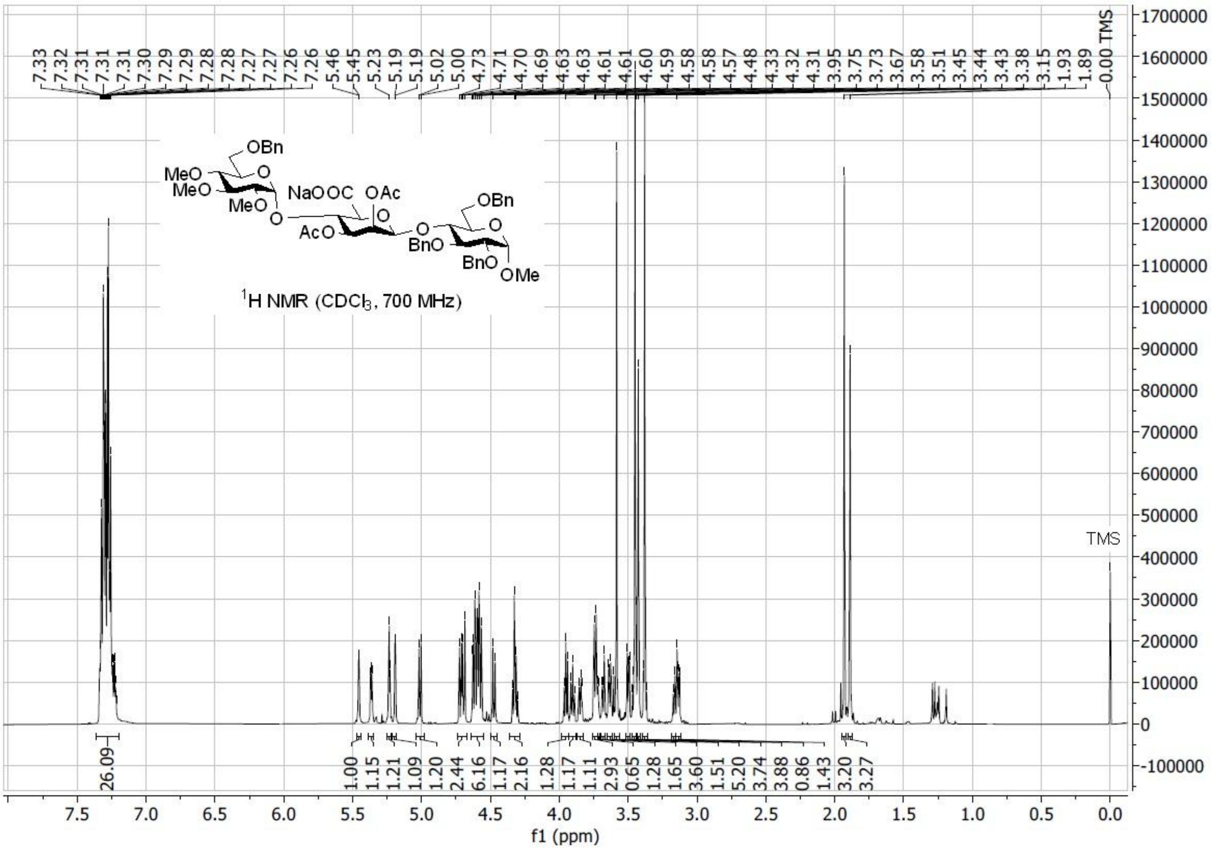

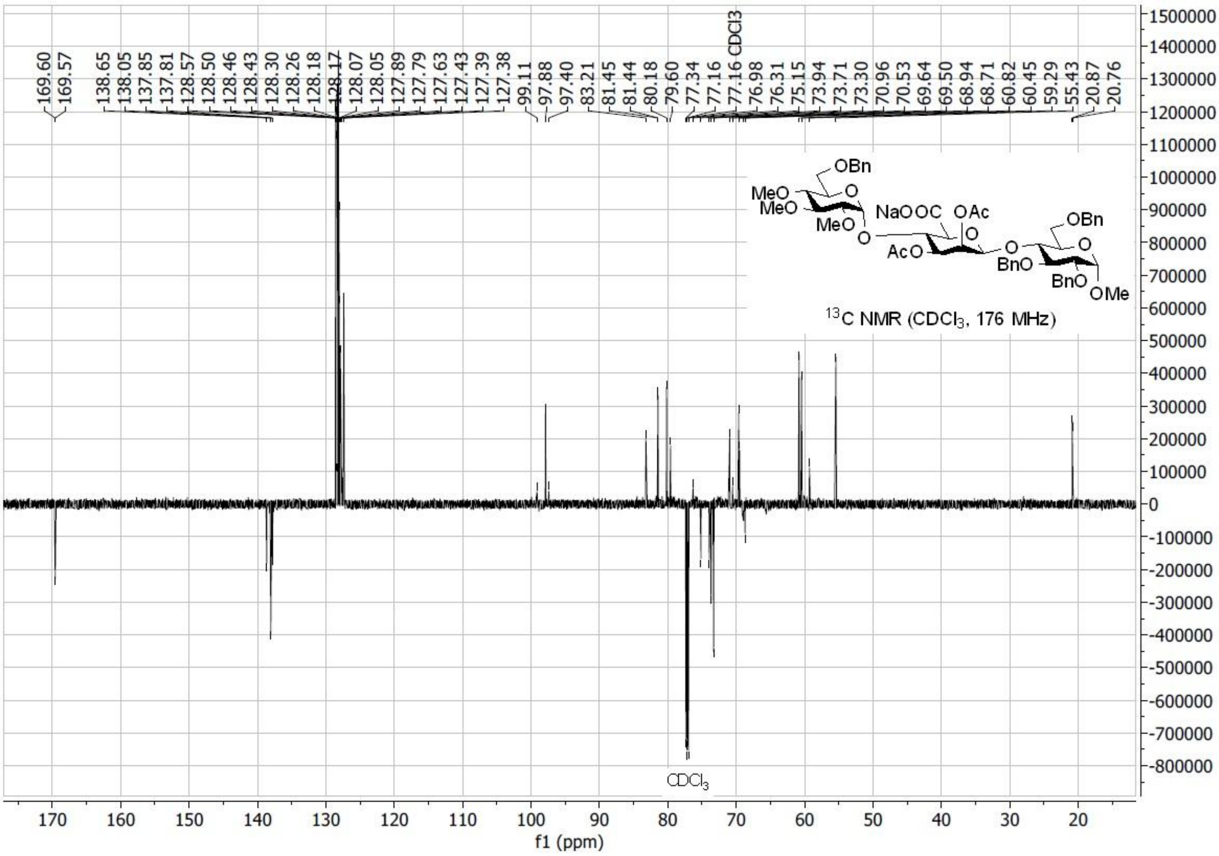

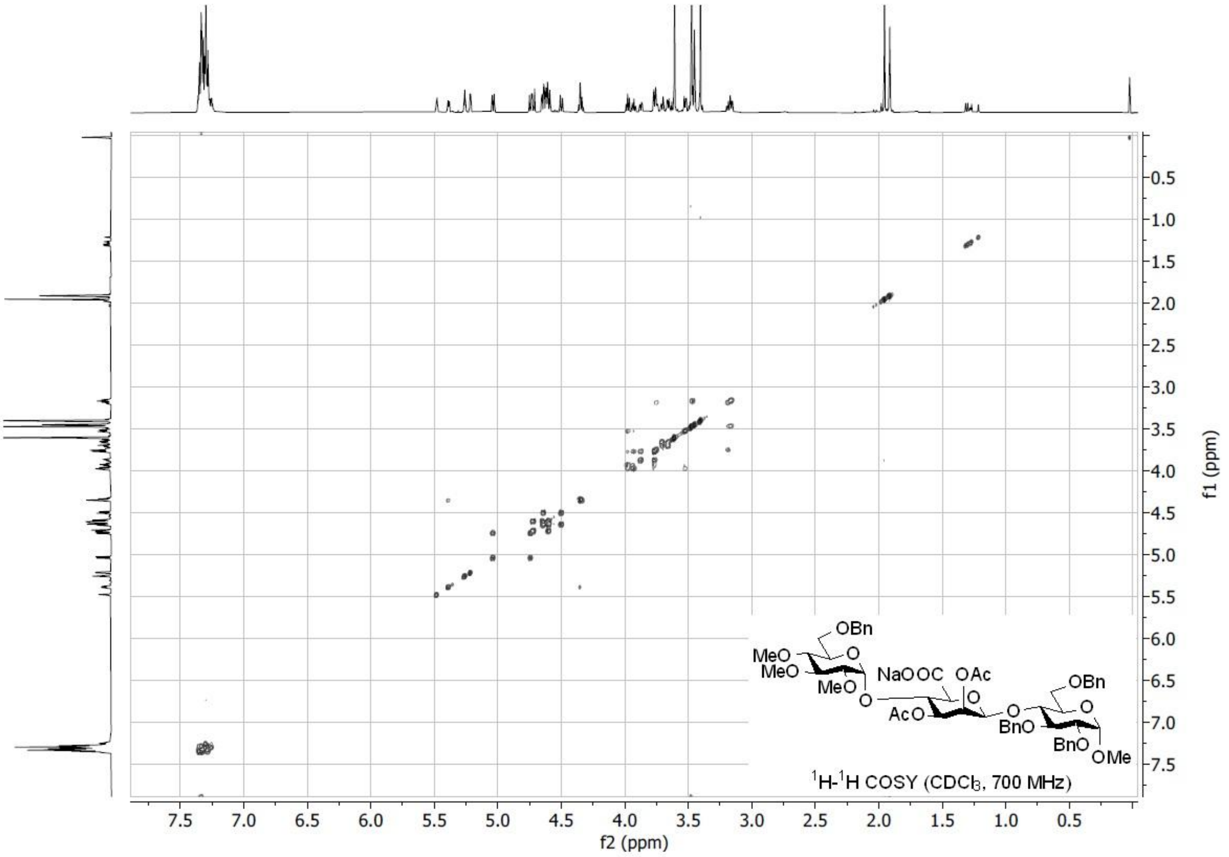

OBn
MeO
MeO
MeO
NaOOC
OAc
OBn
AcO
BnO
BnO
OMe
^{1}H-^{1}H COSY (CDCl$_3$, 700 MHz)
f1 (ppm)
f2 (ppm)

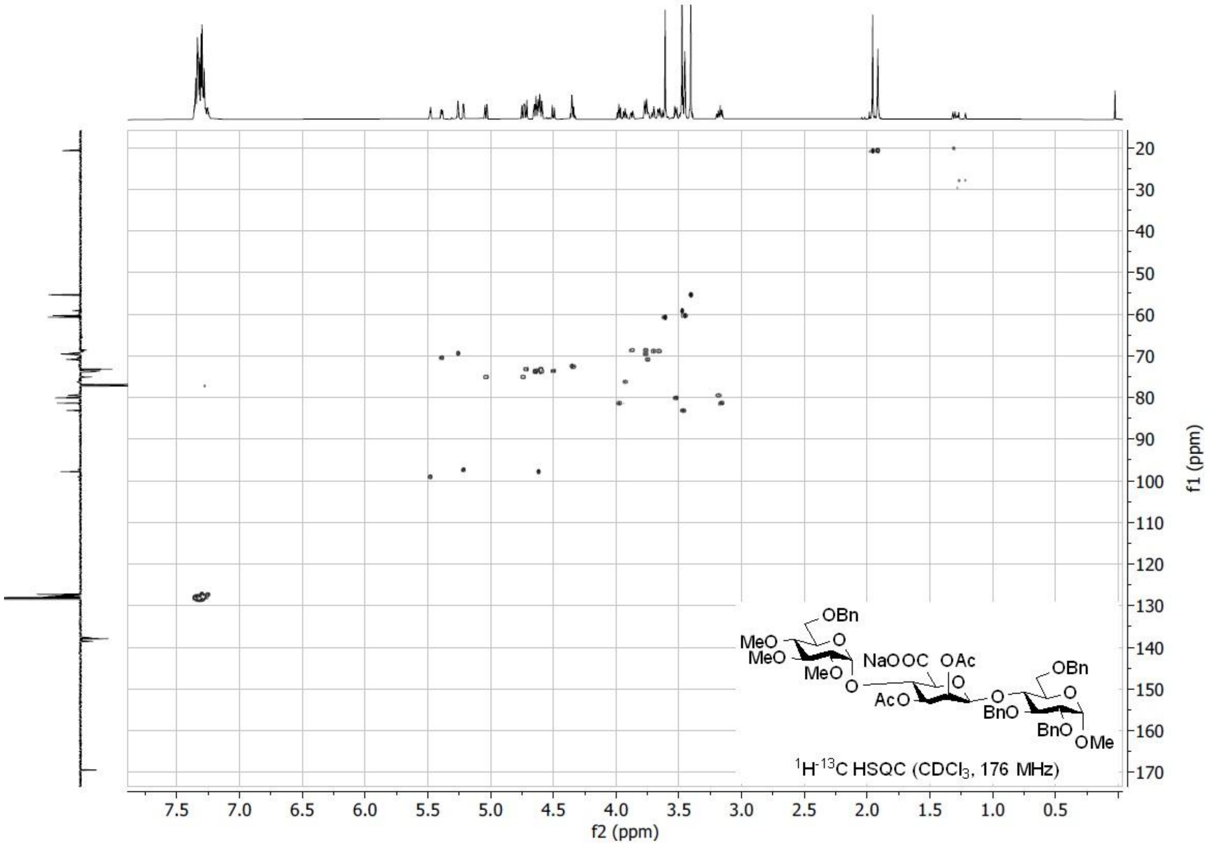

OBn
MeO
MeO
MeO
NaOOC
OAc
OBn
AcO
BnO
BnO
OMe
^{1}H-^{13}C HSQC (CDCl$_3$, 176 MHz)
f1 (ppm)
f2 (ppm)

^1H, ^{13}C, COSY and HSQC NMR spectra of compound **36**

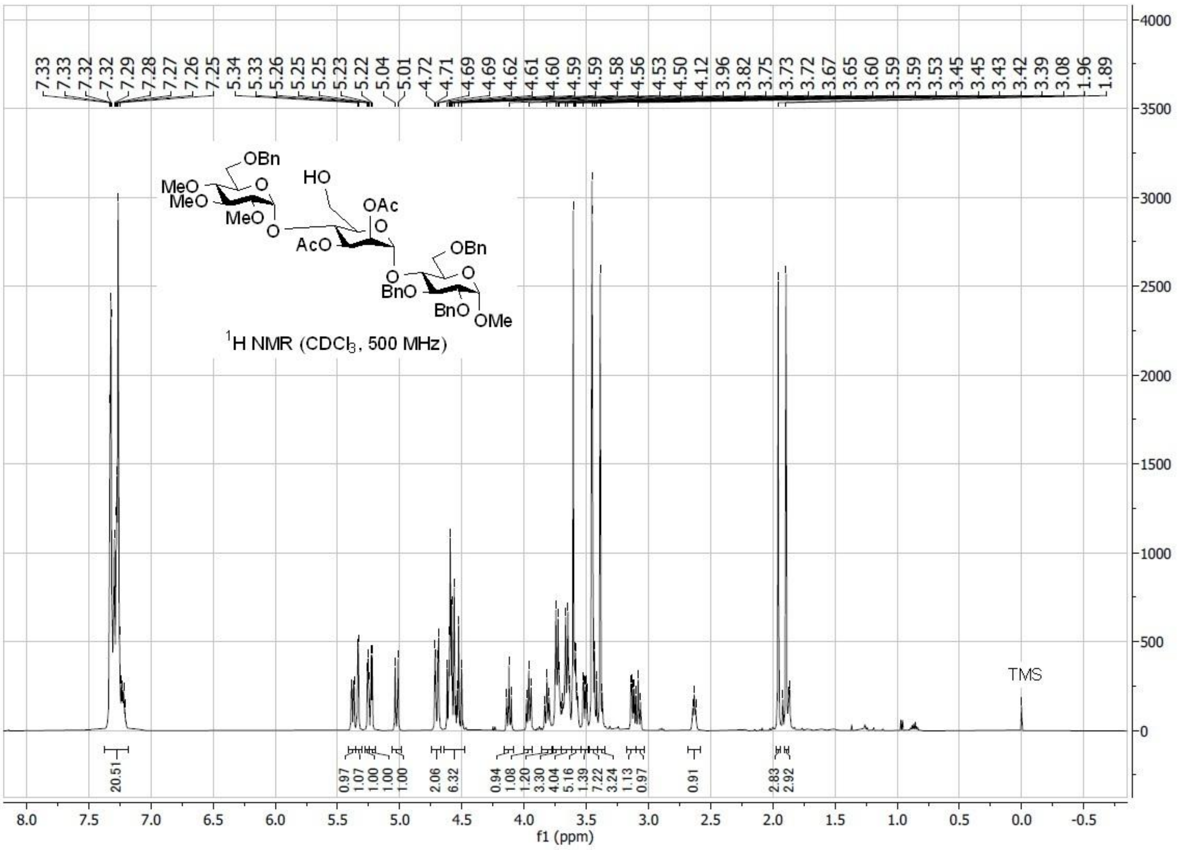

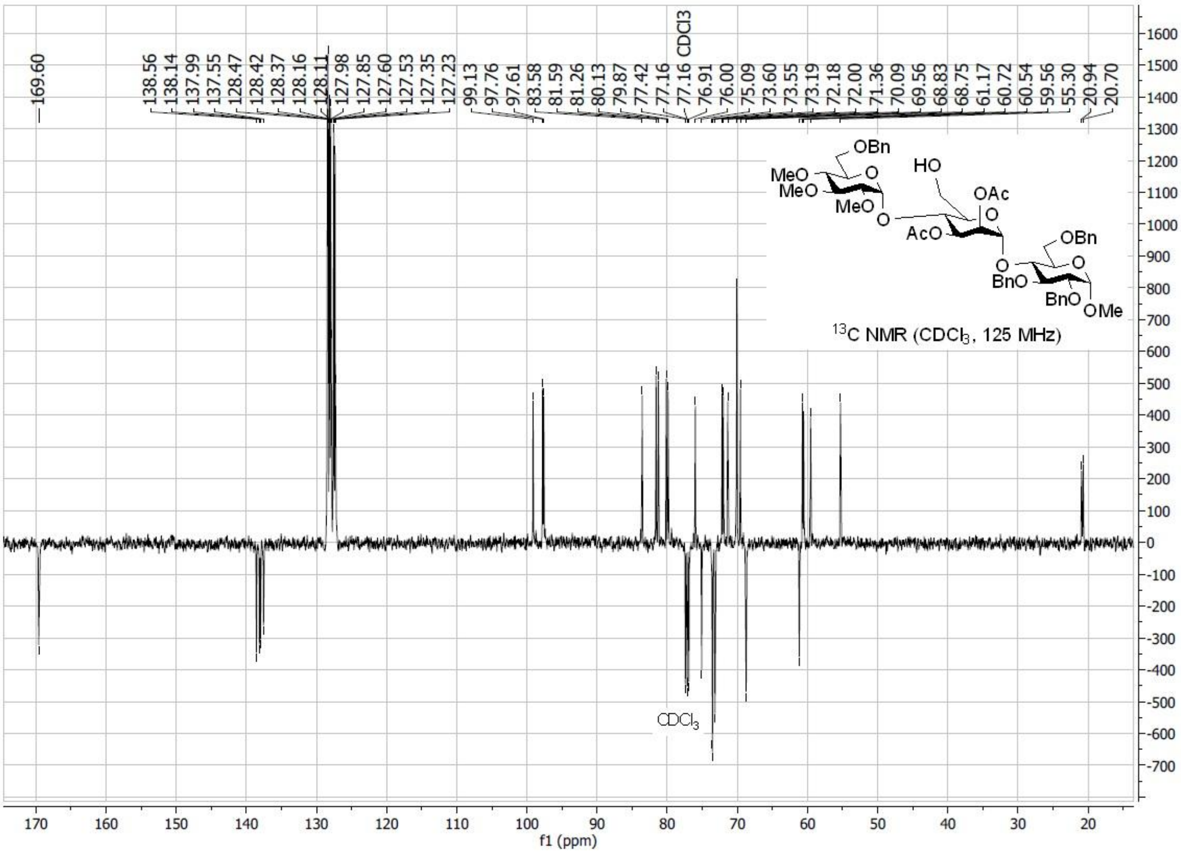

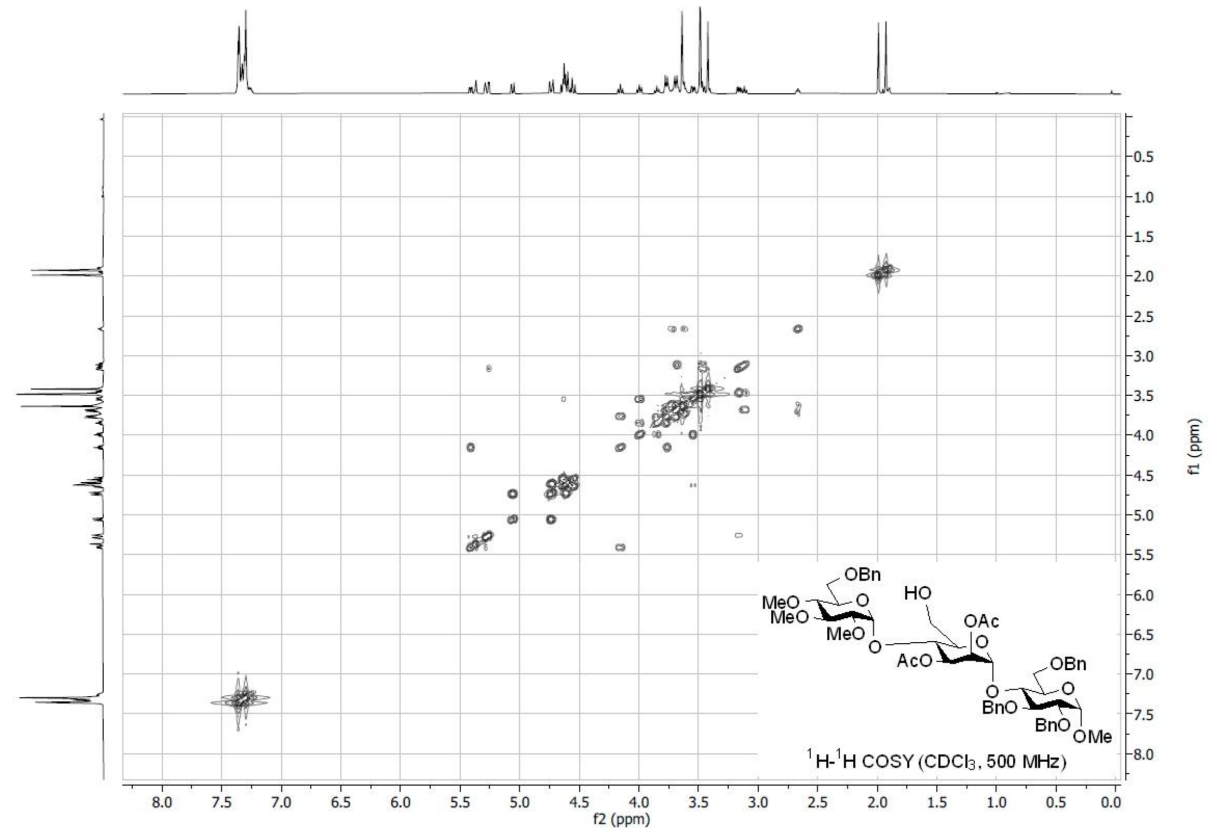

MeO
MeO
MeO
OBn
HO
OAc
AcO
OBn
BnO
BnO
OMe
^{1}H-^{1}H COSY (CDCl$_3$, 500 MHz)
f2 (ppm)
f1 (ppm)

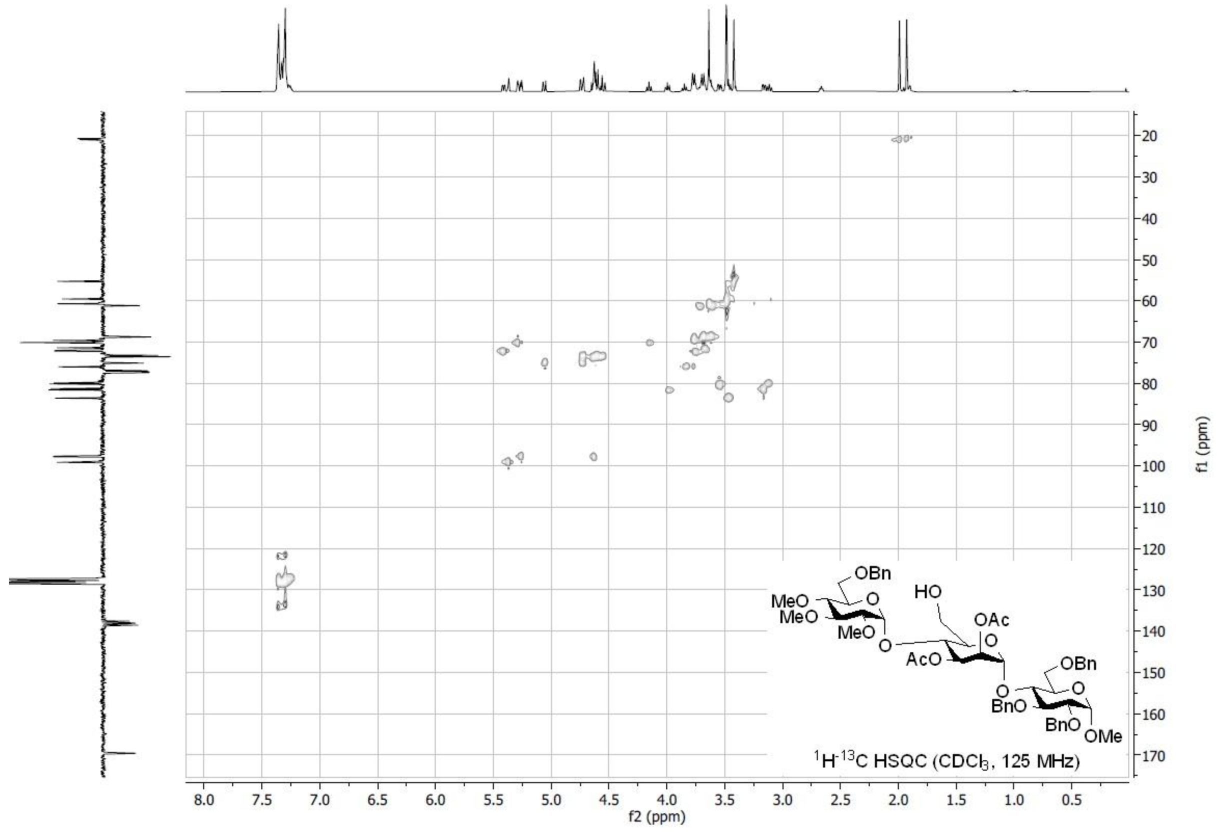

MeO
MeO
MeO
OBn
HO
OAc
AcO
OBn
BnO
BnO
OMe
^{1}H-^{13}C HSQC (CDCl$_3$, 125 MHz)
f2 (ppm)
f1 (ppm)

^{1}H, ^{13}C, COSY and HSQC NMR spectra of compound **37**

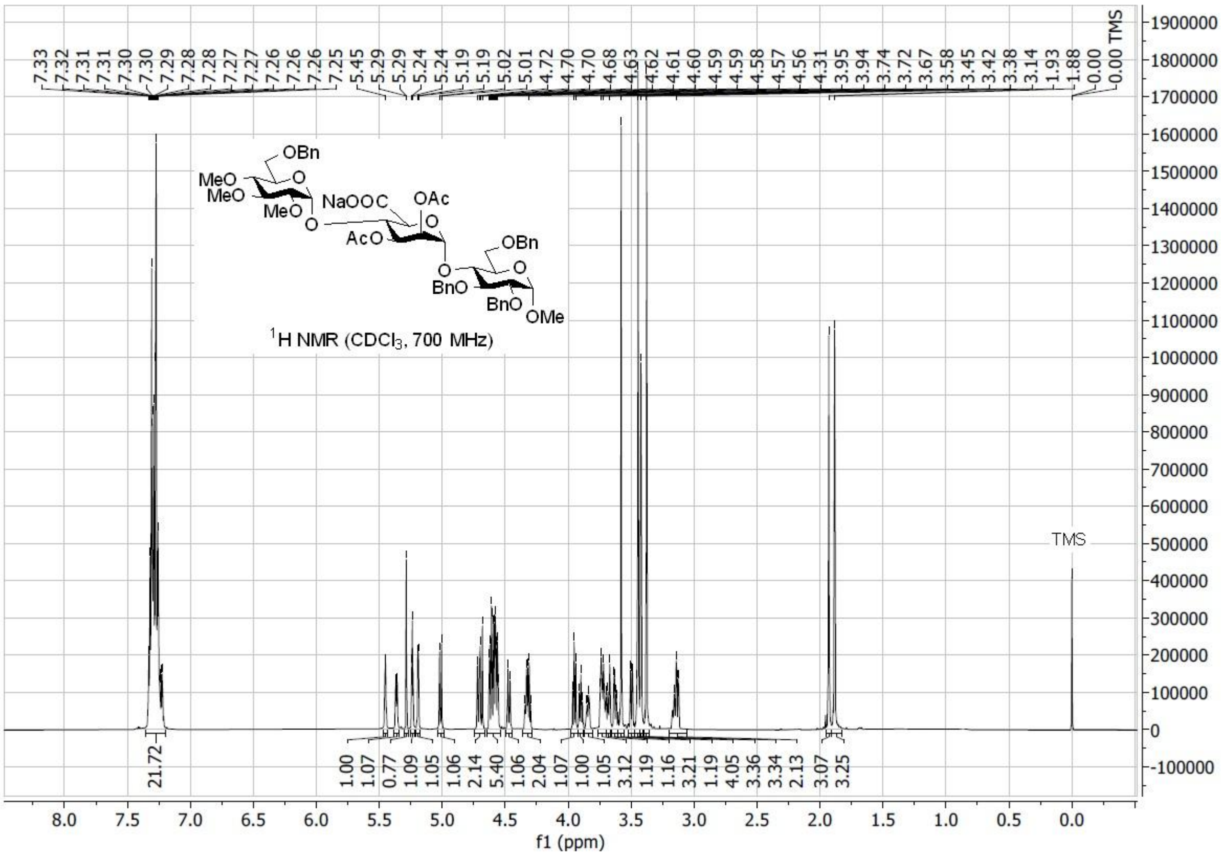

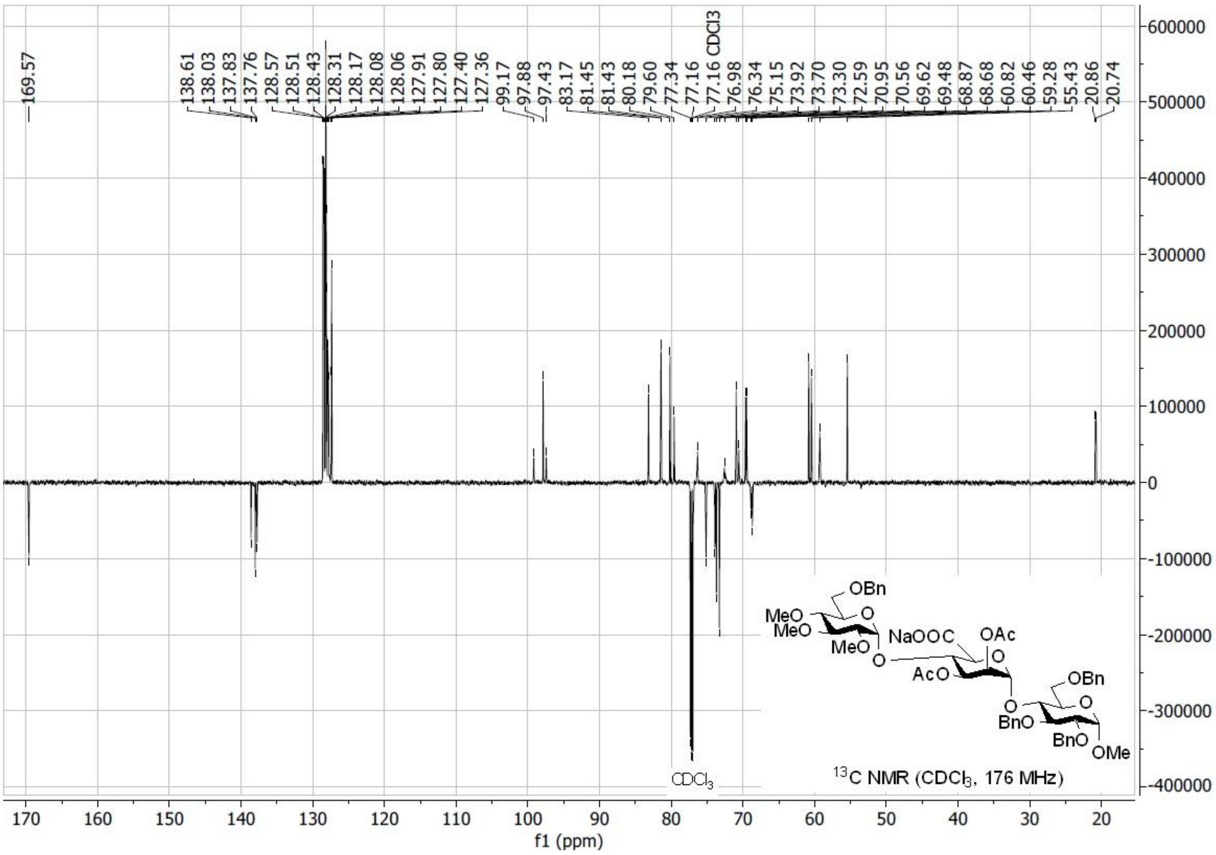

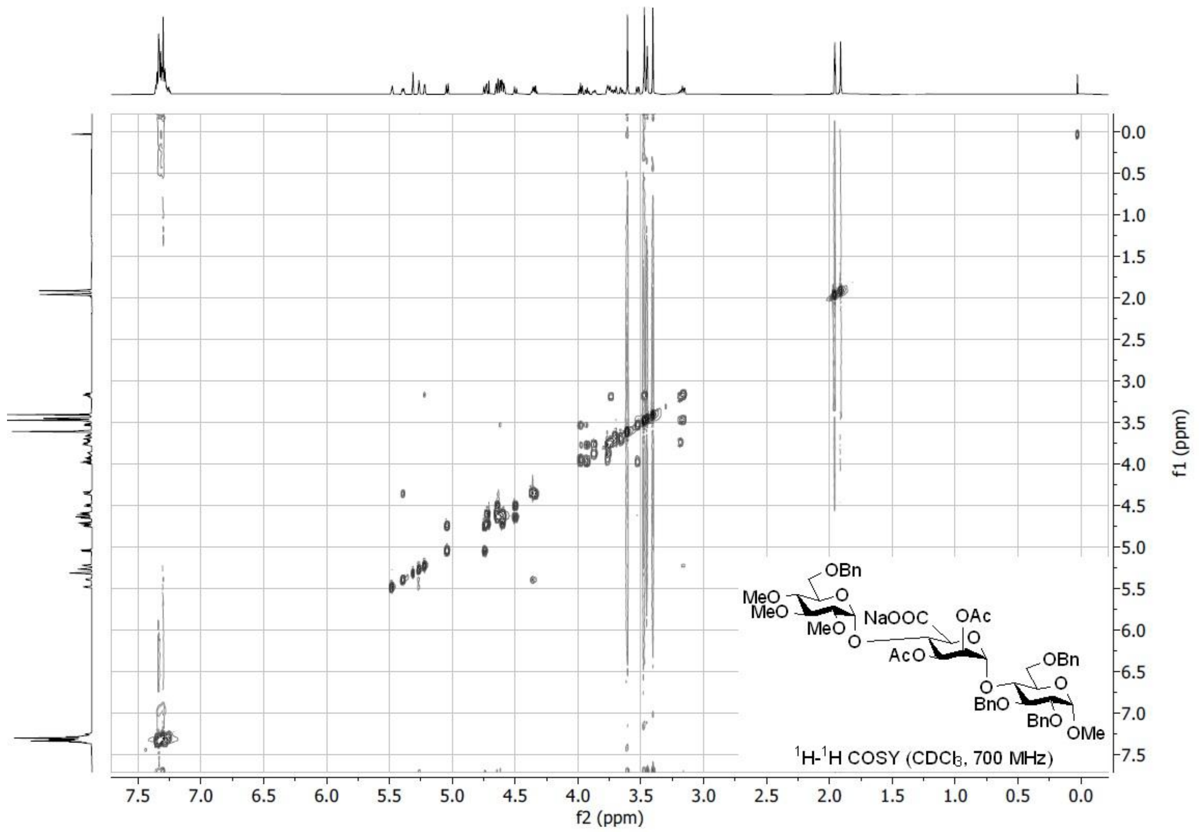

OBn
MeO
MeO
MeO
NaOOC
OAc
AcO
OBn
BnO
BnO
OMe
^{1}H-^{1}H COSY (CDCl$_3$, 700 MHz)
f1 (ppm)
f2 (ppm)

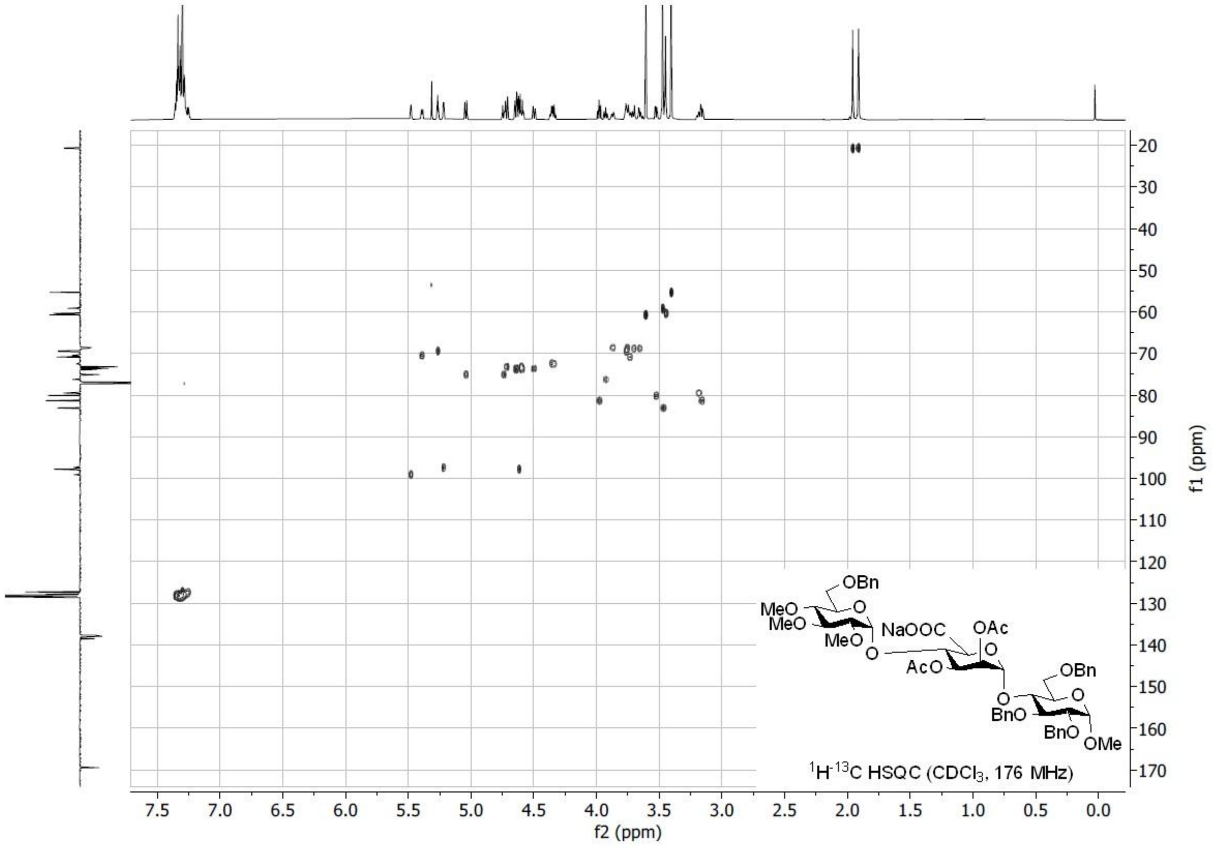

OBn
MeO
MeO
MeO
NaOOC
OAc
AcO
OBn
BnO
BnO
OMe
^{1}H-^{13}C HSQC (CDCl$_3$, 176 MHz)
f1 (ppm)
f2 (ppm)

^{1}H, ^{13}C, COSY and HSQC NMR spectra of compound **38**

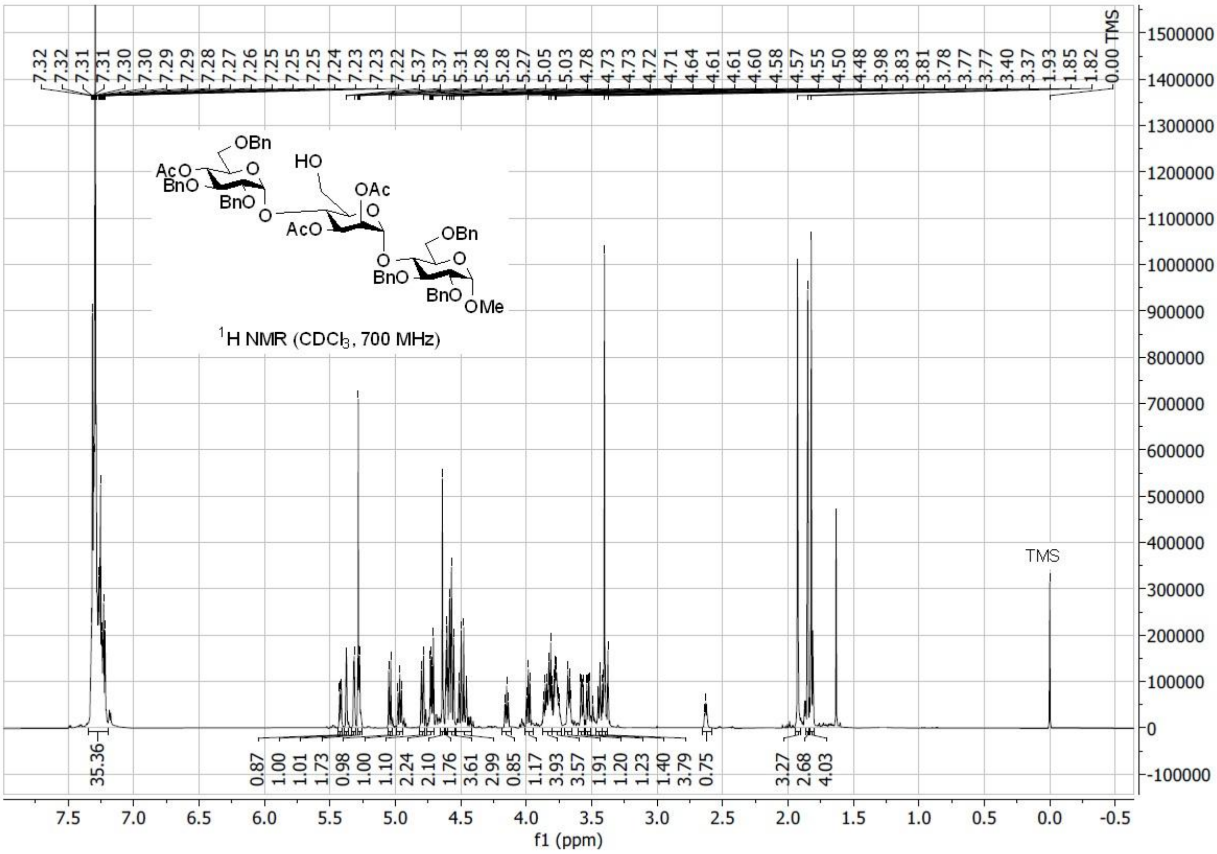

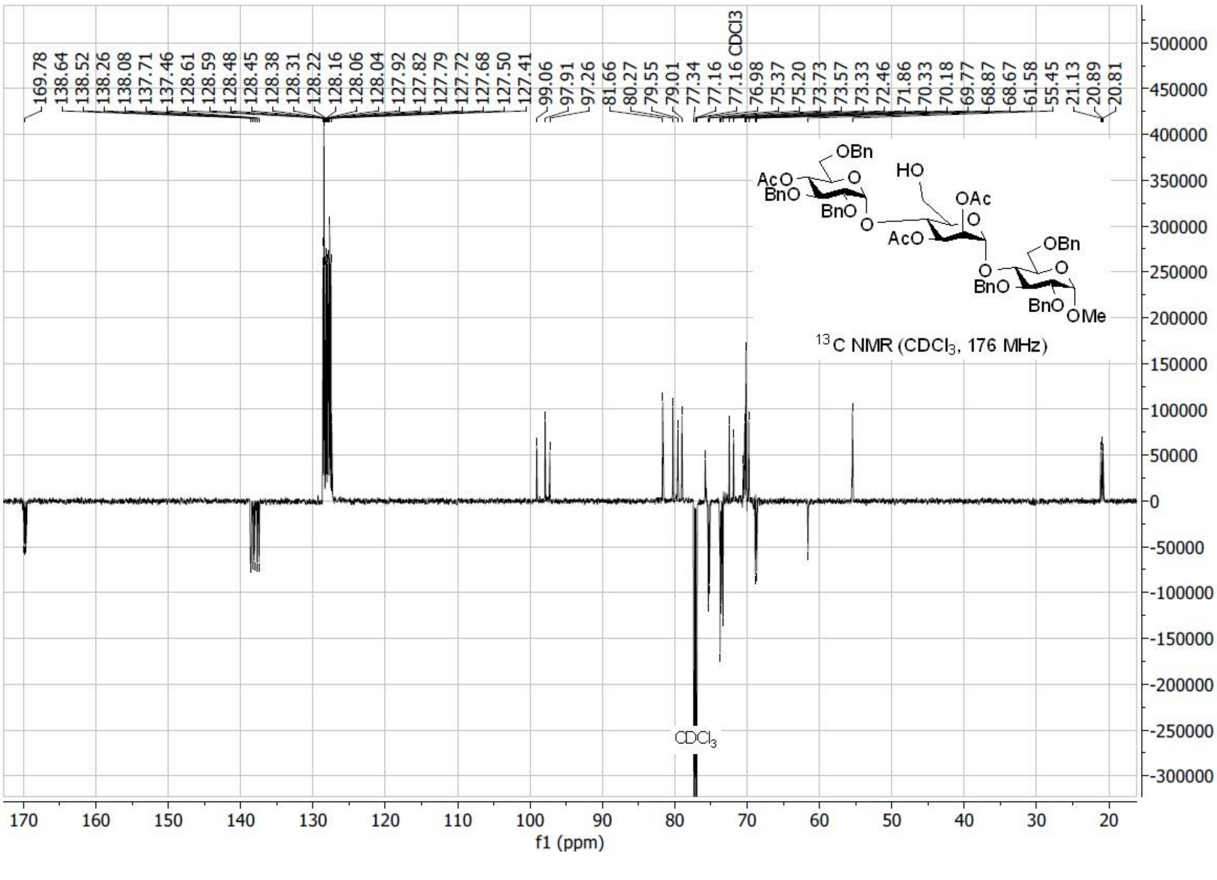

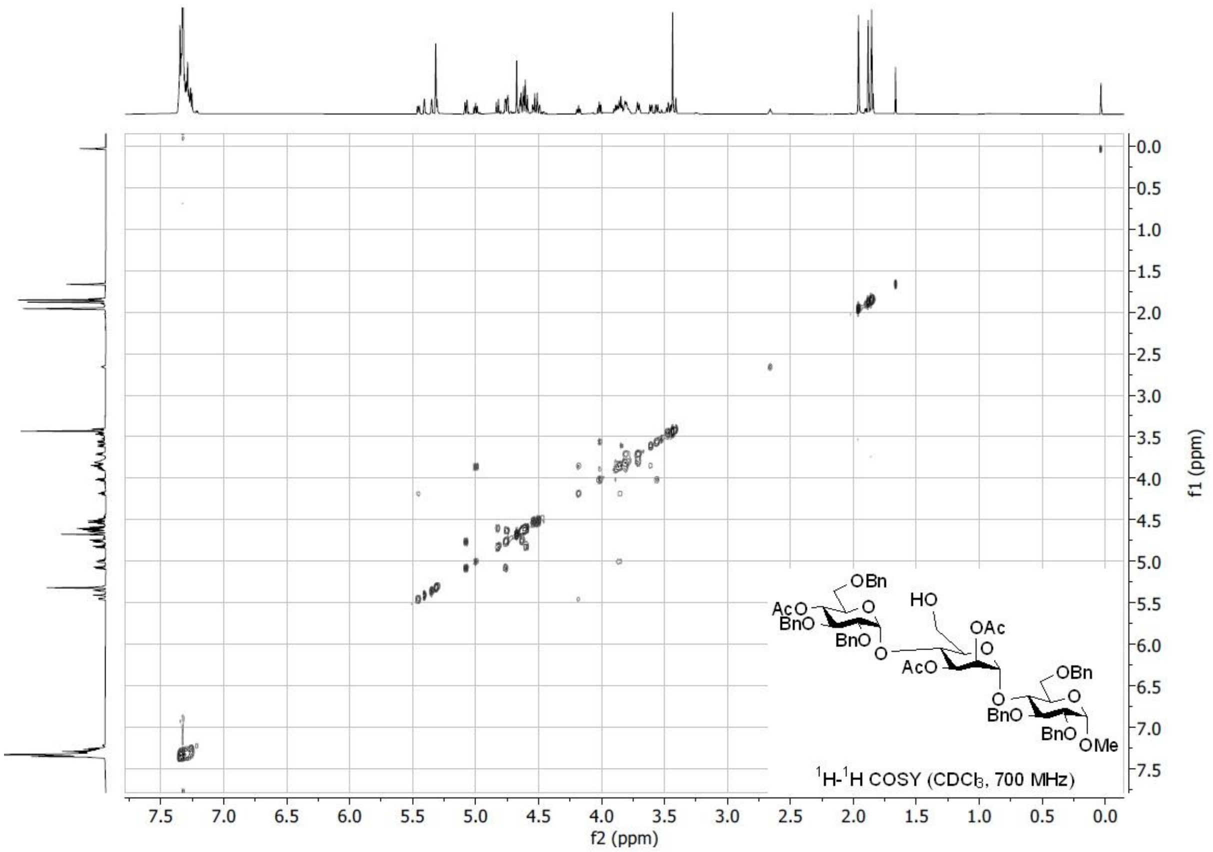
OBn
AcO
BnO
O
BnO
O
HO
OAc
O
AcO
O
OBn
O
BnO
BnO
OMe
^{1}H-^{1}H COSY (CDCl$_3$, 700 MHz)
f2 (ppm)
f1 (ppm)

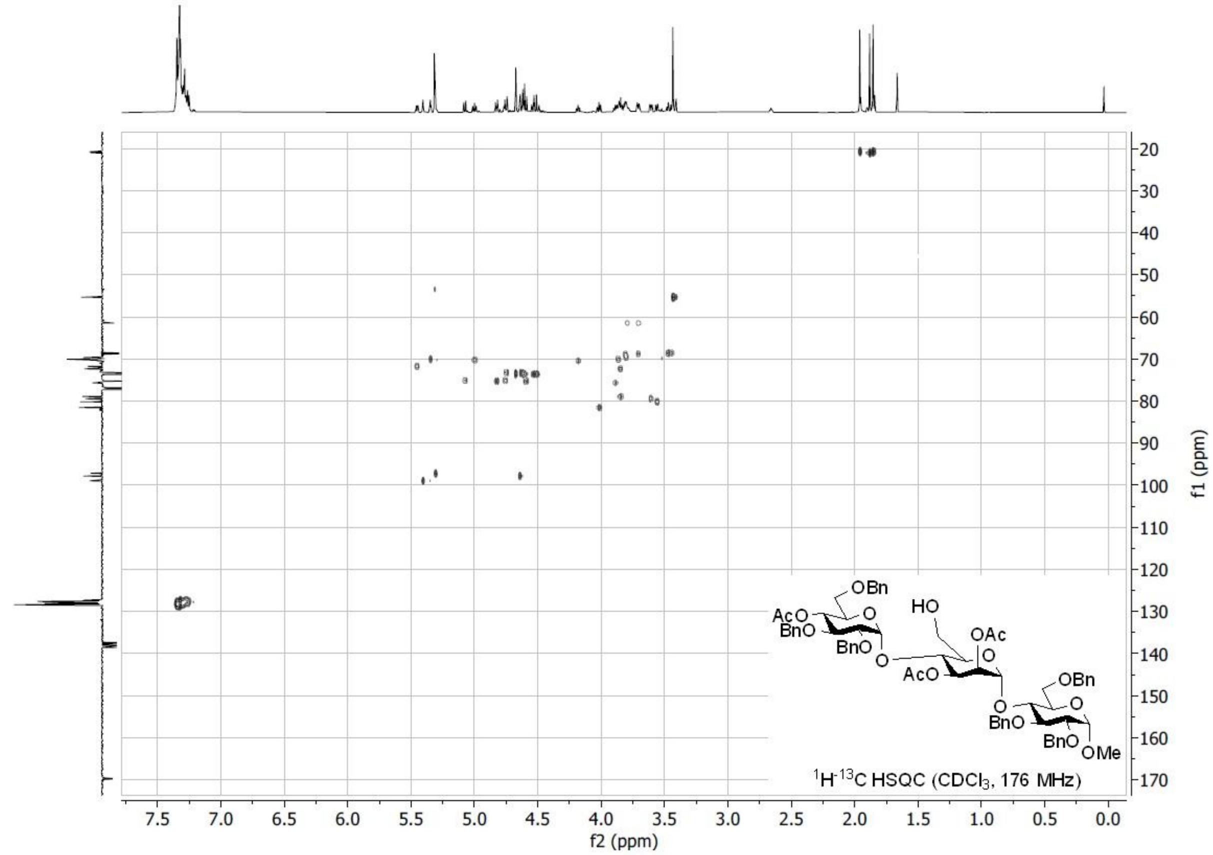
OBn
AcO
BnO
O
BnO
O
HO
OAc
O
AcO
O
OBn
O
BnO
BnO
OMe
^{1}H-^{13}C HSQC (CDCl$_3$, 176 MHz)
f2 (ppm)
f1 (ppm)

^1H, ^{13}C, COSY and HSQC NMR spectra of compound **39**

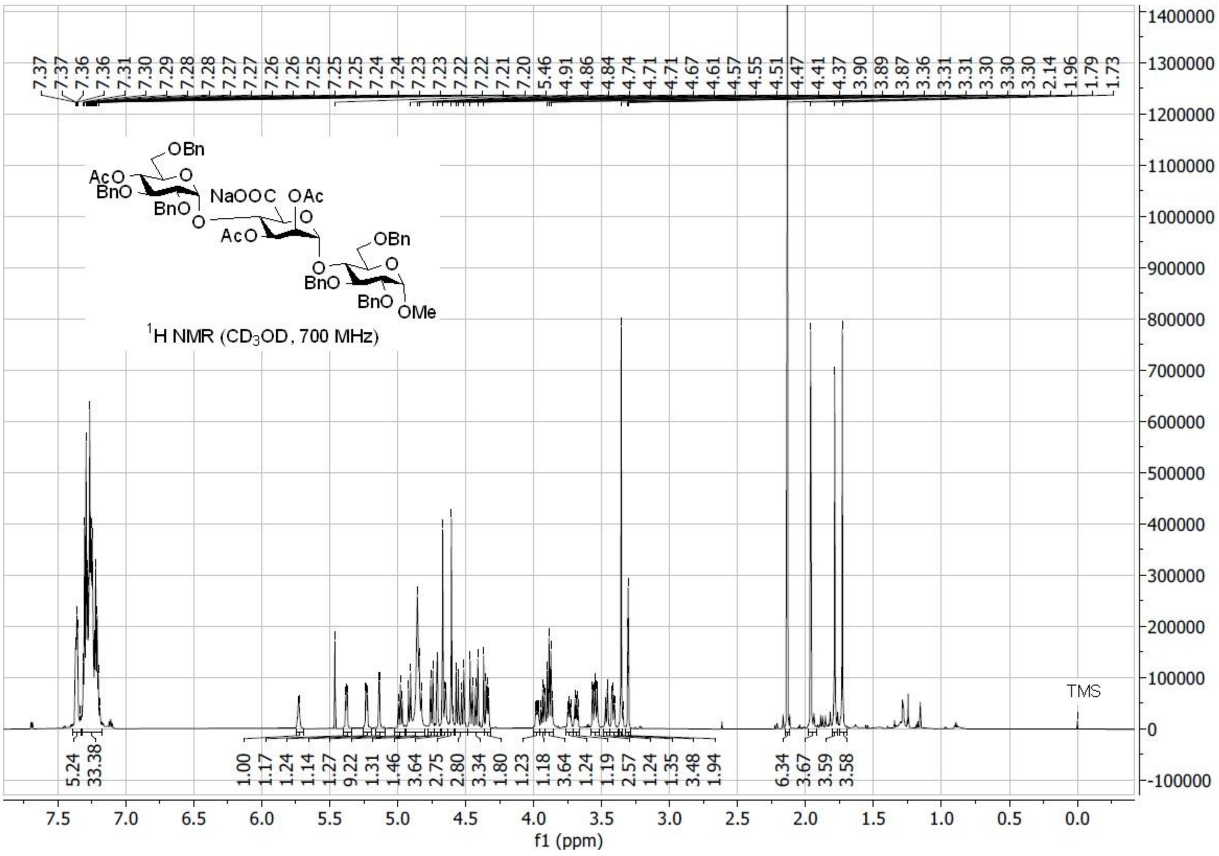

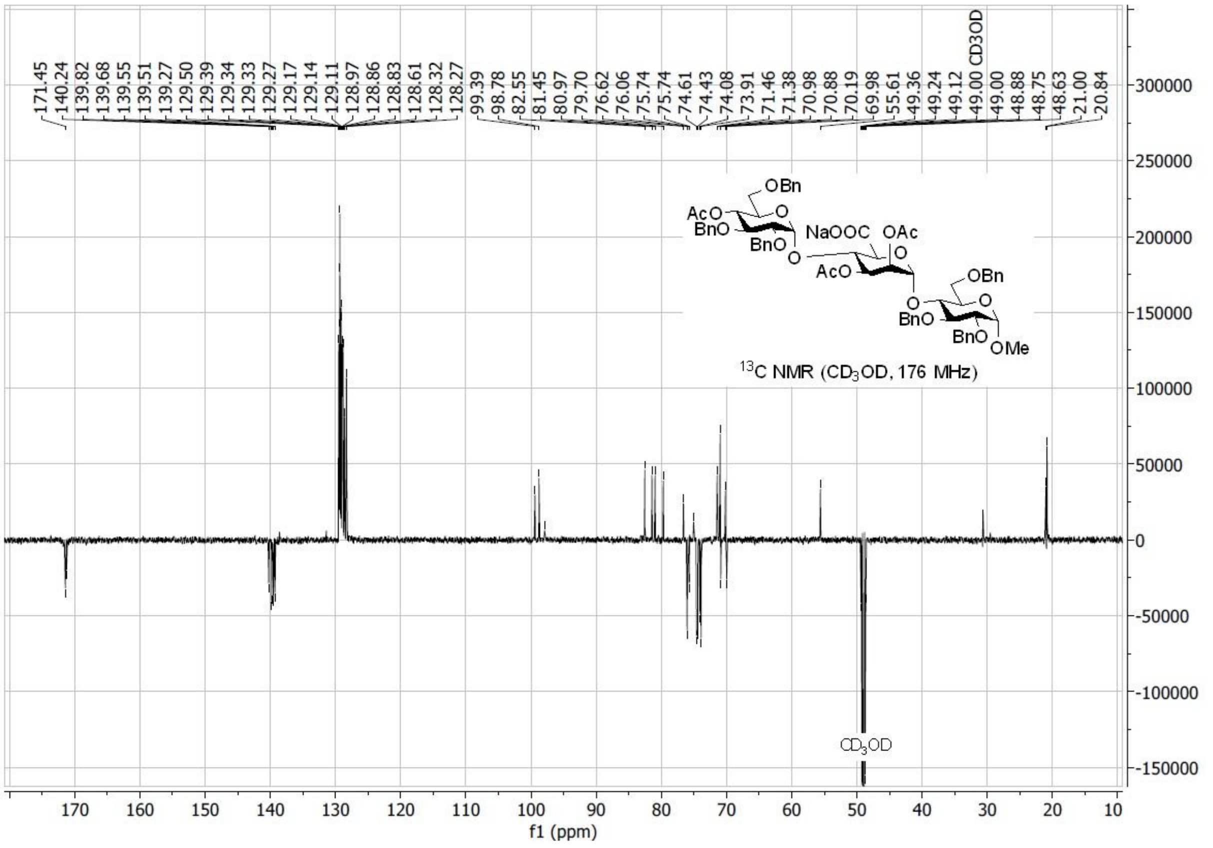

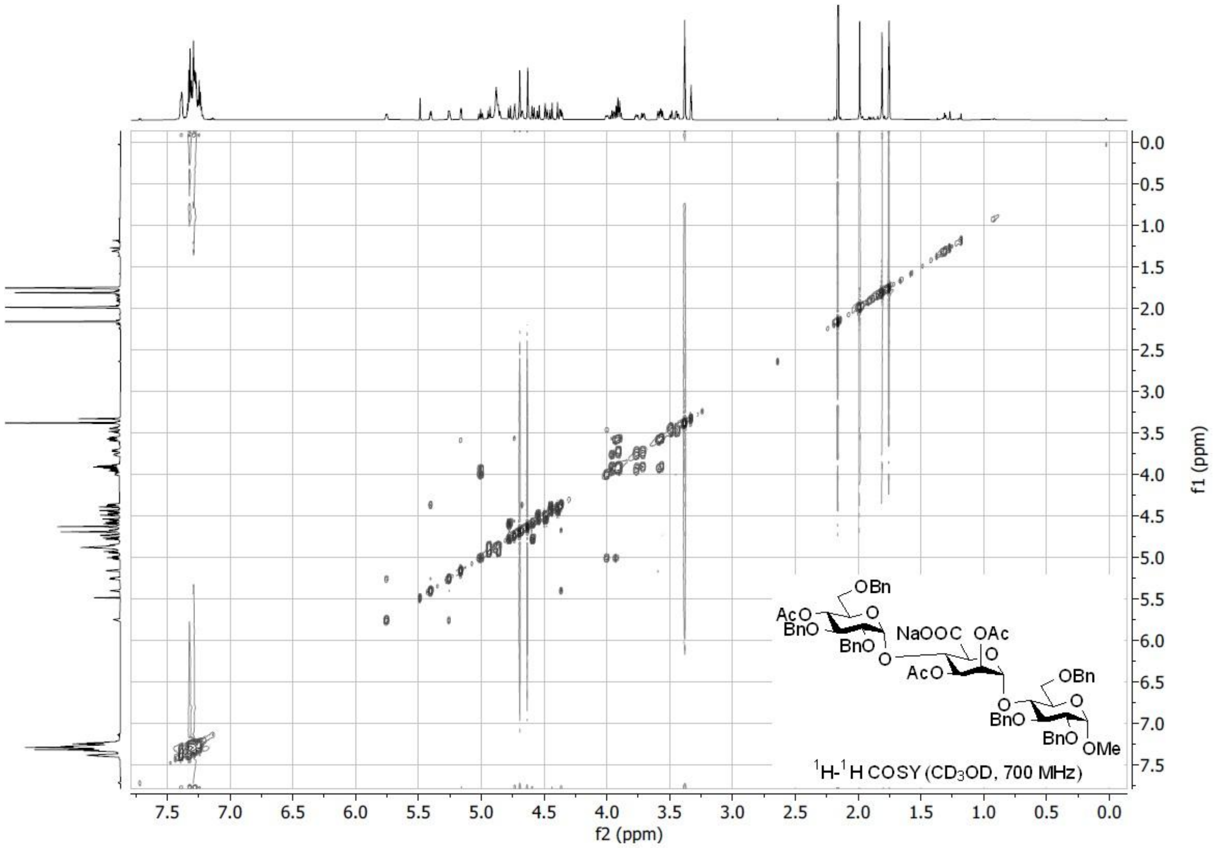
OBn
AcO
BnO
BnO
NaOOC OAc
O
AcO
OBn
BnO
BnO
OMe
^{1}H-^{1}H COSY (CD$_3$OD, 700 MHz)
f1 (ppm)
f2 (ppm)

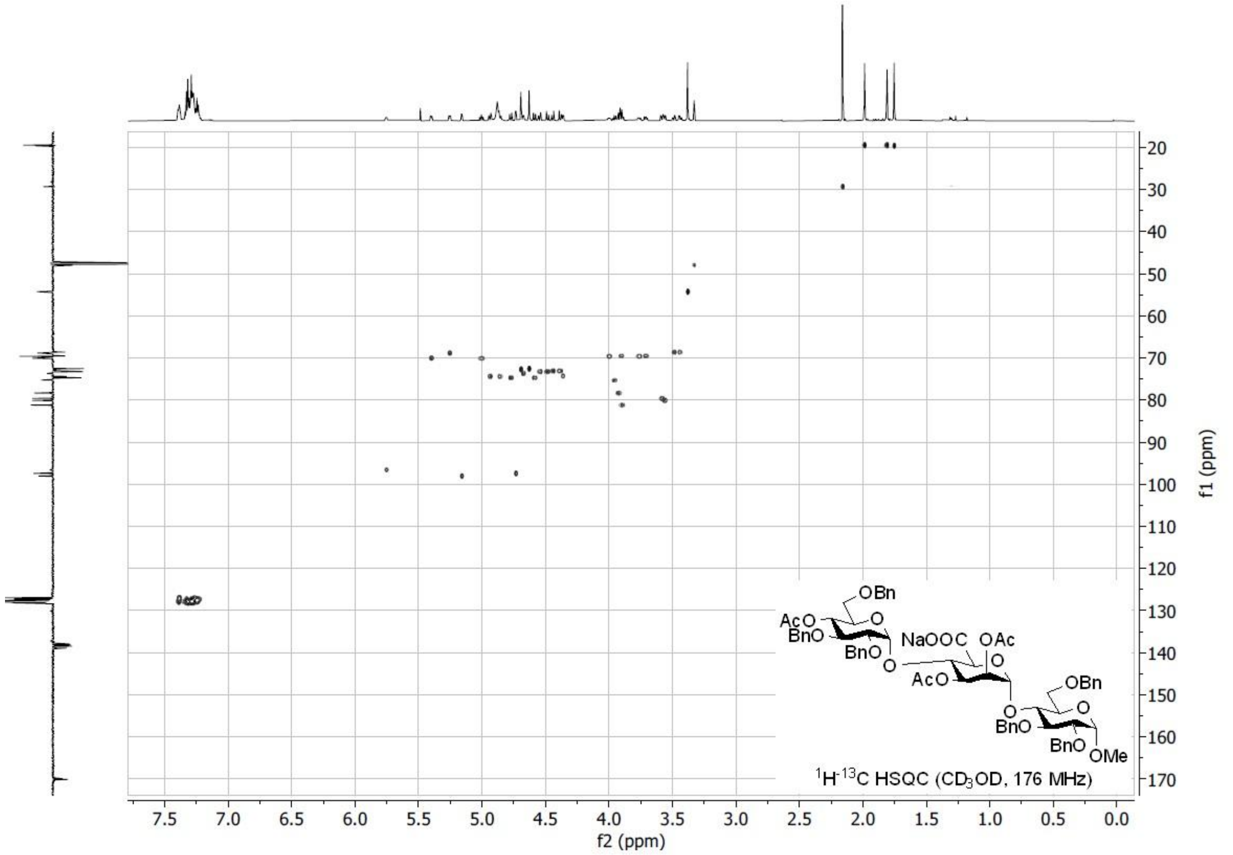
OBn
AcO
BnO
BnO
NaOOC OAc
O
AcO
OBn
BnO
BnO
OMe
^{1}H-^{13}C HSQC (CD$_3$OD, 176 MHz)
f1 (ppm)
f2 (ppm)
